# Private health insurance in Gulf Cooperation Council countries: A scoping review

**DOI:** 10.1016/j.hpopen.2025.100157

**Published:** 2025-12-05

**Authors:** Husein Reka, Robin van Kessel, Elias Mossialos, Wim Groot, Milena Pavlova

**Affiliations:** a Department of Health Services Research, CAPHRI, Maastricht University Medical Center, Faculty of Health, Medicine and Life Sciences, Maastricht University Universiteitssingel 40, 6229 ER Maastricht, the Netherlands; b Department of Health Policy, London School of Economics and Political Science Cowdray House, Houghton St, London WC2A 2AE, United Kingdom

**Keywords:** Private health insurance, Gulf cooperation council, Transformation, Scoping review, Healthcare finance

## Abstract

Private Health Insurance (PHI) in Gulf Cooperation Council (GCC) countries has experienced rapid growth over the past two decades, driven by demographic and economic changes. Although various analyses at the country level have been reported, no study has reviewed PHI systems in the GCC through a methodological approach. We provide a conceptual framework to review, describe and document the development of PHI in the GCC, based on literature from the scoping review. As of December 2023, all GCC countries have laws in place or have promulgated laws establishing mandatory PHI schemes. Most of these schemes are designed for expatriate populations residing in these countries, but there is a trend to extend them to nationals working in the private sector. The health system context plays a role in how PHI emerged and is designed in terms of role, eligibility, and coverage. PHI markets in the region are concentrated and dominated by local companies with performance levels that could be further improved. These markets are maturing and subject to more robust technical and prudential regulations as governments seek to enhance competition. Governments in the region must ensure the sustainable growth of these schemes and a more strategic alignment with health system objectives. Lessons learned from more mature markets are critical for future developments.

## Highlights

• Private Health Insurance (PHI) in GCC grew by CAGR of 23.4 % (2000–2022)
• Governments still dominate healthcare financing from 52 % in UAE up to 87 % in Kuwait.
• Health system context and path dependency shape distinctive features of PHI schemes.
• Governments’ focus should be on performance, equity, and sustainability of PHI also.
• GCC countries can learn from each other on the optimal PHI design and implementation.

## Graphical abstract

**Figure:**
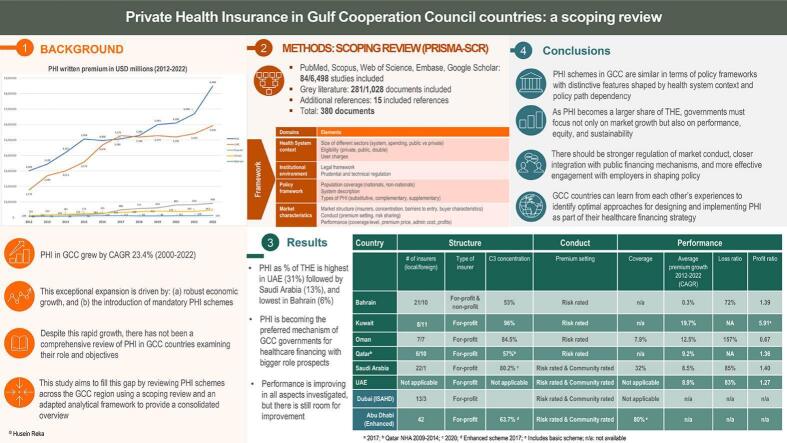
Private Health Insurance (PHI) in Gulf Cooperation Council (GCC) countries scoping review and appraisal graphical abstract

## 1 Introduction

Private health insurance (PHI) is increasingly becoming an essential component of healthcare financing worldwide. The global market for PHI has reached a significant milestone, with an estimated USD 2 trillion in written premiums (2021). This accounts for nearly one-third of all insurance written premiums, showcasing a substantial growth from 8.6 % of total underwritten premiums in 2000 to 31.8 % in 2021 and a compounded average growth rate (CAGR) of 11.1 % [1].

The political history of PHI development worldwide clearly demonstrates its growing role in healthcare financing, with some countries increasingly shifting towards a compulsory model. This has prompted more government intervention in regulating PHI, as its implications for health system performance are becoming even more emphasised [2]. While the growing importance of PHI is undisputed, debates continue over whether it improves access and quality or instead drives cost escalation, overconsumption and greater health system inequities [3], [4]. Nevertheless, as Chollet and Lewis (1997) argue, the priority for governments is to develop a deep understanding of the health insurance industry itself, as this knowledge underpins effective regulation and alignment with broader health system goals [5].

PHI can take on several roles depending on whether they are a primary healthcare financing source or not [2], [6]. In most countries, PHI primarily functions as a supplementary or complementary form of coverage rather than as the core mechanism for healthcare financing. Supplementary PHI typically provides faster access to services, a wider choice of providers and coverage for amenities or services that go beyond what is offered by publicly financed schemes. In contrast, complementary PHI is designed to cover costs not fully reimbursed by public schemes, such as user charges or services excluded from the basic benefit package. Alternatively, PHI can also play a substitutive role, where certain population groups are allowed to opt out of the mandatory public scheme and purchase private coverage instead, as is the case in Germany and Chile [7]. These roles can be beneficial for those who can afford them or who are part of group schemes offered by employers. However, they also risk entrenching inequities if they create a two-tiered system where access and quality depend heavily on the ability to pay.

Only a few countries, such as the Netherlands and Switzerland, have shifted PHI into a compulsory role, heavily regulated by the government, as the primary financing mechanism for universal coverage. These models demonstrate how the structure and regulation of PHI can significantly influence the performance of health systems. In the Gulf Cooperation Council (GCC) countries, where PHI is expanding rapidly, understanding these distinctions is critical. Whether PHI evolves into a more compulsory function under strong or weak regulatory oversight will ultimately determine its long-term impact on access, equity and cost control.

The GCC region has experienced even faster growth in PHI than global trends, with CAGR of 23.4 % from 2000 to 2022. This growth rate is three times higher than life insurance growth and twice as high as non-life insurance growth in the GCC region [1]. This exceptional expansion is driven primarily by two factors: a) robust economic growth, which has increased demand for insurance coverage and b) the introduction of mandatory PHI schemes, which has further accelerated uptake. These developments reflect both a government preference for this financing model and a clear policy direction [8], [9], [10], [11], [12], [13], [14]. Given this rapid and policy-driven expansion, there is a pressing need to examine how PHI schemes are structured and evolving across the GCC and identify common patterns, challenges and opportunities for strengthening their alignment with broader health system goals.

Despite this rapid growth, there has been no comprehensive review of PHI in GCC countries examining its role and objectives. Most of the existing literature focuses on individual countries, while more GCC countries are either introducing or considering mandatory PHI schemes. This study aims to fill this gap by reviewing PHI schemes across the GCC region and documenting their structure, role and objectives. Using a scoping review and an adapted analytical framework, we provide a consolidated overview that sets the scene for more detailed studies on the impact of PHI on access, equity and broader health system goals.

## 2 Methods

### 2.1 Methodology framework

A review of PHI in GCC countries was performed using a scoping review as an underlying methodological approach. The collection of information and data pertinent to the topic of this research augmented this. To frame the review, we utilised key PHI studies to establish the methodological framework for data collection and analysis (Table 1). Specifically, to describe PHI schemes in the GCC region, we used Preker et al.’s framework with further adaptations [3]. Based on previous literature [5], [15], [16], [17], types of coverage, roles of PHI and regulatory approaches were incorporated as additional elements. Thus, the analytical framework has four domains: (a) health system context, (b) institutional environment, (c) PHI policy framework and (d) market characteristics.

**Table 1:** Private Health Insurance descriptive and review framework.

**Framework domains**	**Domain elements**
**Health system context**	· Size of different sectors (system, spending, public vs. private) · Eligibility (private, public, double) · User charges

**Institutional environment**	· Legal framework · Prudential and technical regulation

**Policy framework**	· Population coverage (nationals, non-nationals) · System description · Types of PHI (substitutive, complementary, supplementary)

**Market characteristics**	· Market structure (insurers, concentration, barriers to entry, buyer characteristics) · Conduct (premium setting, risk-sharing) · Performance (coverage level, premium price, admin cost, profits)

Sources: Preker et al (2006), Foubister et al (2006), OECD (2004); Mossialos et al (2004). Schieber et al (1997); Mossialos and Thomson (2002).

### 2.2 Search strategy

A search strategy was developed to capture data and information from five electronic databases (PubMed, Scopus, Web of Science, Embase and Google Scholar). We used a three-step search strategy to locate both published and unpublished studies. The first step was an initial limited search in the databases indicated above. This was done to understand the existing literature and inform the development of the search strategy. The second step involved a comprehensive search using all identified search criteria across all databases. The syntax for this main search was developed together with a librarian. As this was done through a scoping review, we have identified the participants, concept and context of the search (Table 2). As a third step, the search strategy, including all identified keywords and index terms, was adapted for each database. The keywords for each database are provided in Appendix A.

**Table 2:** Search strategy keywords by participants, concept, and context of study.

Category	Keywords
Participants: Private Health Insurance	“Insurance”[MeSH] OR insurance*OR ((health* AND (expenditure* OR financ* OR payment* OR cost* OR regulat* OR budget*))
Concept: Transformation terms	reform* OR transform* OR chang* OR strateg* OR shift* OR impact*
Context: Country health systems terms	Bahrain OR Kuwait OR Oman OR Qatar OR “Saudi Arabia” OR KSA OR “United Arab Emirates” OR UAE OR “Gulf States” OR Dubai OR “Abu Dhabi”

The grey literature search was conducted systematically on the websites of national regulators in GCC countries and other relevant portals, i.e. searching defined relevant entities’ websites and document repositories using defined keyword(s). A list of websites and portals for the entities searched is provided in Appendix B. The search was conducted through a saturation approach, starting with the latest available year of legislation, policies and other documents. While each entity’s databases required a unique approach to retrieve documentation, keywords used to retrieve documentation were ‘insurance’ and ‘health insurance’ where a search engine was available. Where not, all records were reviewed manually on the entity's website by looking at the above keywords. A search methodology is provided in Appendix C.

### 2.3 Screening process

References that cover the participants, concept, and context of the study were included. The following limitations were applied to the search: studies published in English and Arabic from 1999. Following the search, all identified citations were collated and uploaded into EndNote version 21 for deduplication, followed by a screening process. A Google Scholar search was conducted separately, including only the first 300 records for each country. These records were then imported into EndNote, following established guidance documents, and included in the deduplication exercise [18]. Following a pilot test, all titles and abstracts were screened by a single reviewer (HR). A second reviewer (RVK) screened 20 % of the titles and abstracts for validation and quality purposes. Disagreements were resolved by discussion with other researchers. Potentially relevant sources were retrieved in full, and their citation details were imported into EndNote version 21 [19].

The full text of selected citations was assessed in detail against the inclusion criteria by two reviewers (HR and RVK). Any disagreements that arose between the reviewers at each stage of the selection process were resolved through discussion or with an additional reviewer. Search results and study inclusion process are reported in full in the results section and presented in a Preferred Reporting Items for Systematic Reviews and Meta-analyses extension for scoping review (PRISMA-ScR) flow diagram [20]. The final step was to screen the reference list for additional references.

### 2.1 Data extraction and analysis

Data were extracted from papers included in the scoping review by one reviewer (HR). The extracted data includes specific details about the participants, concept, context, study methods and key findings relevant to the domains of the review framework. Data extraction was further refined by checking the first five papers by (RVK) and (MP). A draft extraction form is provided in Appendix D. In case of any uncertainties, a discussion with the research team was held. The extracted data were synthesized using a framework analysis approach, a structured form of thematic analysis [21]. This involved charting data from the included documents against a pre-specified analytical framework adapted from Preker et al. (2006), as described above, and related literature. Within each domain of this framework, we identified and refined themes iteratively, ensuring that data from all sources (academic and grey literature) were systematically compared and contrasted. This framework analysis allowed us to move beyond a simple descriptive summary of country-level findings and to highlight cross-cutting patterns and variations across GCC countries [21], [22]. Details of each extracted record are available in the supplement material.

## 3 Results

### 3.1 Literature search

**Fig. 1:**
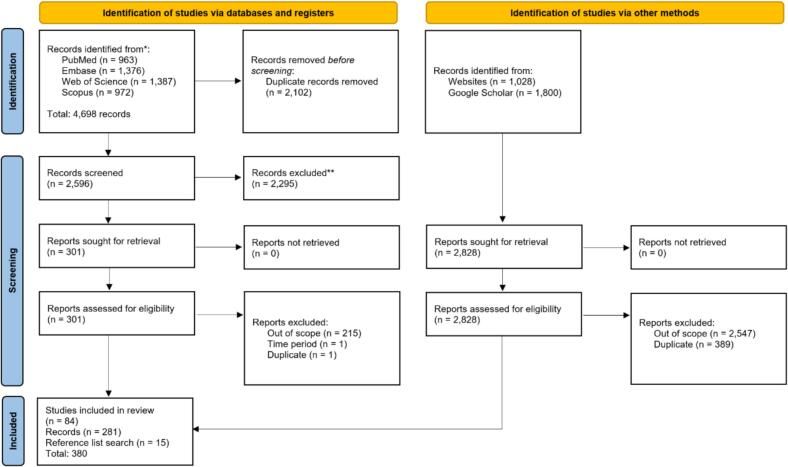
PRISMA 2020 flow diagram for search and screening process.

The database search returned 6,498 references, which, after deduplication, were reduced to 4,077 references. After screening for eligibility, we ended up with 301 references, of which 84 were selected for review. A grey literature search initially identified 1,028 records, out of which 281 were deemed eligible for the study objectives and were retrieved accordingly. Additionally, a reference list search was conducted to enrich the information required for study objectives, yielding 15 additional records (Fig. 1). All databases were merged, and information was extracted to populate our descriptive and review framework. The extraction matrix containing data per included record is included in Appendix D.

Most eligible papers originated from Saudi Arabia (49/84; 58.3 %) [12], [23], [24], [25], [26], [27], [28], [29], [30], [31], [32], [33], [34], [35], [36], [37], [38], [39], [40], [41], [42], [43], [44], [45], [46], [47], [48], [49], [50], [51], [52], [53], [54], [55], [56], [57], [58], [59], [60], [61], [62], [63], [64], [65], [66], [67], [68], [69], [70], followed by the United Arab Emirates (UAE) (14/84; 16.7 %) [10], [11], [13], [71], [72], [73], [74], [75], [76], [77], [78], [79], [80], [81], Qatar (7/84; 8.3 %) [8], [82], [83], [84], [85], [86], [87], Kuwait (5/84; 5.9 %) [9], [88], [89], [90], [91], Oman (3/84; 3.6 %) [92], [93], [94] and Bahrain (2/84; 2.4 %) [95], [96]. One-third of the included studies are descriptive (28/84), looking at the effect of health insurance on out-of-pocket payments, access to health care services and satisfaction with PHI. Several country reports focused on the health systems of GCC countries and discussed health insurance. Quantitative studies, in general, have applied some form of regression analysis (multivariate, logistic, quantile, linear) looking at possible associations between private health insurance and satisfaction, out-of-pocket expenditure, health outcomes and knowledge of products. Similarly, 245 out of the 281 (87 %) eligible grey literature comes from Dubai (108/281; 38.4 %), Saudi Arabia (72/281; 25.6 %) and Abu Dhabi (65/281; 23.1 %). Most of the identified documents issued by different regulators in GCC countries come in the form of circulars, followed by policies and decisions issued by governments, such as laws, regulations and ministerial decisions.

The findings are organised around the four domains of our analytical framework: (1) health system context, (2) institutional environment, (3) PHI policy framework and (4) market characteristics. For each domain, we summarise cross-cutting findings across the GCC countries and then highlight essential differences by country where relevant. Key similarities and divergences are also presented in the following sections to aid interpretation for readers less familiar with the region.

### 3.2 Health system context

Across the GCC countries, the role of PHI is closely shaped by the broader health system arrangements, particularly government-financed schemes for nationals and employer-mandated schemes for non-nationals (Table 3). While publicly funded health services cover most nationals, non-nationals typically rely on mandatory PHI schemes as their primary form of coverage. These differing arrangements influence the relative size of PHI markets, the scope of benefit packages and the use of user charges. This subsection describes how PHI interacts with existing public health system functions, revenue collection, pooling and purchase and highlights variations in population eligibility and financial protection across countries.

**Table 3:** Overview of healthcare financing functions in GCC countries.

Country	Revenue Collection	Pooling	Purchasing	Benefit package
Bahrain	Government budget	Single pool (SCH)	Block budget (SCH)	Implicit (SCH)
Kuwait	Government budget PHI premium	Single pool (MOH) Single pool (Afya)	Block budget (MOH) Fee for service (PHI)	Implicit (MOH) Explicit (Afya)
Oman	Government budget	Single pool (MOH)	Block budget (MOH)	Implicit (MOH)
Qatar	Government budget	Single pool (MOPH)	Block budget (MOPH)	Implicit (MOH)
Saudi Arabia	Government budget PHI premium	Single pools (MOH, NG, MOD, other) Multiple pools (CHIS)	Block budget (MOH, NG, MOD, other) FFS/DRG (CHIS)	Implicit (MOH, NG, MOD, other) Explicit (CHIS)
United Arab Emirates	Government budget PHI premium	Single pools (MOHAP, Thiqa, Basic, SAADA) Multiple pools (ISAHD, Enhanced)	Block budget (MOHAP) Fee for service/DRG (Thiqa, ISAHD, Basic, Enhanced)	Implicit (MOHAP) Explicit (Thiqa, ISAHD, SAADA, Basic, Enhanced)

SCH: Supreme Council of Health; MOH: Ministry of Health; FFS: Fee-for-service; MOPH: Ministry of Public Health; NG: National Guard; MOD: Ministry of Defence; CHIS: Cooperative Health Insurance Scheme; DRG: Diagnoses Related Groups; MOHAP: Ministry of Health and Prevention; ISAHD: Insurance System for Advancing Health in Dubai.

Currently, only two countries in the GCC have operational mandatory PHI schemes: Saudi Arabia and the UAE. In all cases where the government is the primary funder, we have single pooling, operating implicit packages that are mainly funded by block budgets. Conversely, PHIs operate in multiple pools with explicit benefit packages, primarily using fee-for-service as a purchasing tool. The only exceptions are the UAE and Saudi Arabia, where PHI schemes have been utilising case-based payment models, such as diagnosis-related groups.

#### 3.2.1 Sector size

Regarding size, the share of PHI in total health expenditure (THE) varies among GCC countries. The highest penetration of PHI is seen in the UAE, constituting 30.9 % of healthcare financing revenues, followed by Saudi Arabia with 13.3 %. The lowest shares of PHI are registered in Bahrain, at only 6.4 %, while in Kuwait and Oman, PHI contributes relatively higher, at 8.5 % and 12.3 %, respectively, with expectations that these two markets will increase manifold once PHI becomes mandatory [97].

This breakdown illustrates variations resulting from the modus operandi of PHI, i.e. whether it is a mandatory or voluntary role, while also reflecting the distinct characteristics of health systems. Dubai is a case in point where PHI contributes with 47 % of the THE due to its mandatory role and significant non-national population covered by PHI [98]. Conversely, in Saudi Arabia, although PHI is mandatory for the private sector, the contribution to the THE is just 13.3 % as the government still covers the majority of the local population.

Despite the varying extent of PHI size in the region, PHI in the GCC is witnessing an upwards trend in growth between 2012 and 2022, with Kuwait experiencing the highest growth (CAGR 22.6 %), compared to the lowest registered growth in Bahrain (CAGR 6.4 %). Saudi Arabia, Oman and the UAE experienced stable double-digit growth during this period, with CAGRs of 11 %, 16 % and 13 %, respectively (Table 4).

**Table 4:** Healthcare spending in GCC countries (2019).

	Bahrain	Kuwait	Oman	Qatar	Saudi Arabia	United Arab Emirates
Written premiums (USD Millions)	97.60	618.14	411.84	NA	5,993.31	5,727.27
Insurance penetration (%of GDP)	0.25	0.44	0.47	NA	0.71	1.26
Total healthcare spending as % of GDP	4.01	5.50	4.39	2.91	5.69	4.3
Public healthcare spending as a % of total healthcare spending	59.20	86.96	87.55	72.76	69.17	52.13
Private health insurance spending as % of total healthcare spending	6.4	8.5	12.3	NA	13.3	30.9

Source: AXCO country reports and World Bank database. NA: Not available

#### 3.2.2 Eligibility

Analysis of eligibility provides insights into additional complexities (Table 5). While the non-national population is almost exclusively covered by PHI through employers (except in rare cases when employed by the government in Dubai and Saudi Arabia), the situation with nationals is more fluid, where the possibility of dual or even multiple eligibilities exists in practice. In general, GCC countries show dichotomy in terms of eligibility, i.e. separate schemes for non-nationals and nationals, except the Cooperative Health Insurance Scheme in Saudi Arabia, which has evolved into more of a hybrid model covering both groups (one-third of 11.5 million beneficiaries in 2022 were nationals) [99]. The scheme initially covered only the non-national population; however, in 2002, it was decided to extend coverage to nationals employed in the private sector [100].

**Table 5:** Eligibility for health insurance coverage of nationals and non-nationals in the GCC countries.

		Abu Dhabi	Bahrain	Dubai	Kuwait	Oman	Qatar	Saudi Arabia
	Applicable Law	Law No. 23 of 2005	Law No. 23 of 2018	Law No. 11 of 2013	Decision No. 586 of 2010 Law No. 1 of 1999 − Amended by Law No. 15 of 2019	Decision No. 34/19 of 2019	Law no.22 of 2021	Decree No. M/10 of 1999

Coverage	Nationals	Covered under the *Thiqa* program, exclusively managed by Daman* * Note the coverage may differ based on the legals status of the Emirati within Abu Dhabi	Covered under The Health Insurance Fund − HIF (SHIFA) of Bahrain in the public sector Optional Private Package is available for Bahraini nationals for coverage in private sector	Covered under the *Enaya* as long as not receiving other Government insurance	Covered under public health system & Afya (for Retirees)* *Kuwaiti Cabinet approved a one-year suspension of the program (via Decree-Law No. 105 of 2024), effectively pausing the implementation of Law No. 114/2014 for retirees	Covered under the public health system Optional insurance through Dhamani that may be provided by the employer in private sector	Covered under public health system through HMC and PHCC facilities exclusively	Covered under PHI if employed in private sector
	Non-nationals	Employers are mandated to provide health insurance. Three key segments exist - Employer funded Basic Policy for persons with limited income (exclusively offered by Daman) or - Employer based Enhanced Health Insurance Policy for high income individual - Expatriate Resident cover for those who not covered by the Employer’s health insurance (i.e. Parents)	Employers are mandated to provide health insurance; dependents are covered as stipulated in employment contract	Employers are mandated to provide health insurance to employees and dependents	Employers are mandated to provide health insurance through Dhaman (for expatriate workers on Article 18 Visas and their families) i.e. domestic workers of Kuwaiti nationals who covered under Article 20 and therefore need not be covered under the health insurance scheme	Employers are mandated to provide health insurance through Dhamani; dependents are covered if sponsored by employer; not enforced/ practiced	Employers are mandated to provide health insurance inclusive of dependents; not enforced/ regulated	Employers are mandated to provide health insurance

Funding	Nationals	Automatically enrolled in Thiqa; that is fully state-funded by Abu Dhabi Government	Government through HIF covering medical treatment at public facilities. Optional Private Package is available with subsidized co-payments (40 %) for treatment at private healthcare facilities Government subsidizes the remaining 60 %.	Auto-enrolled in Government insurance if emirate-specific criteria are met; fully funded by the Dubai Government	Government funding through public health system	Government funding through public health system	Government funding through public health system	Mandatory employer provided insurance
	Non-nationals	Mandatory employer-provided insurance	Mandatory employer-provided insurance	Mandatory employer-provided insurance (EBP + Enhanced)	Mandatory employer provided insurance Domestic workers of Nationals are addressed through the government fund	Mandatory employer provided insurance	Mandatory employer provided insurance	Mandatory employer provided insurance

Dubai and Abu Dhabi have developed a model where non-nationals are exclusively covered by PHI and nationals by government-sponsored schemes through third-party administrators (TPA). Kuwait and Qatar, similarly to Dubai and Abu Dhabi, have plans to create separate schemes for nationals and non-nationals, with a slight difference in Qatar, where nationals will continue to be covered within the public health system. In contrast, Oman and Bahrain plans show more similarity with the Saudi model, focusing on eligibility based on a public versus private employer split [101], [102], [103], [104], [105].

Our review registered multiple eligibility for nationals in Kuwait and Saudi Arabia, with the latter prohibiting this in August 2023 [106]. The same provision was implemented in Abu Dhabi earlier in 2009, and an attempt was made in Qatar in 2015 [107], [108]. The issue of dual eligibility is expected to dissipate further as GCC countries introduce health insurance schemes for nationals only, with clear eligibility rules. For instance, Saudi Arabia recently established a single-payer entity for nationals, and Kuwait is expected to do the same [9], [64]. However, this creates opt-in and opt-out options (‘revolving door’ versus ‘one-off’ opting out) for nationals to switch between the private and public sectors, which have been observed in more developed PHI systems around the globe and may offer potential lessons for GCC countries [2].

#### 3.2.3 User charges

User charges are a common practice for PHI in GCC countries, but they are almost non-existent in the public sector (Table 6). PHI mandatory schemes in GCC countries apply different user charge regimes, reflecting different population coverage mix and medical specificities. For instance, in Saudi Arabia and Abu Dhabi, there is no co-payment for hospital admission (except for maternity in Abu Dhabi), while Dubai applies a 10 % coinsurance. Outpatient user charges are applied with variations in extent and exemptions [109], [110], [111]. Oman’s user charges for the mandatory benefit package are similar to the ones in Dubai and Abu Dhabi, with a distinction that maternity is an optional coverage to cater for the single male labour population [112]. Bahrain, as part of the insurance programme scheme for non-nationals, has introduced user charges that are very similar to those of other GCC countries [113]. We found no information on user charges for the forthcoming PHI schemes in Kuwait and Qatar.

**Table 6:** Overview of user charges and exemptions in PHI schemes in GCC countries.

Country	User charges	Exemptions
Saudi Arabia (Basic scheme)	• Inpatient accommodation shared room (no copayment; USD 160/day limit) • Emergency treatment (no copayment) • Primary care clinic visit: family medicine, GP, OBG, Internal medicine (0–5 %) • Specialist clinic with referral (0–10 %) • Specialist clinic without referral (0–50 %) • Medicines: generics and brands without substitution (20 %) • Medicines: brands with available substitution (50 %) • Dental essential and preventive (no copayment; USD 320/year limit) • Dental root canal and emergencies (20 %; USD 213/year limit) • Obesity procedures (0–20 %; USD 4,000 limit)	• Not applicable • Not applicable • Maximum USD 6.7 • Maximum USD 20 • Maximum USD 133 • Maximum USD 8 • Maximum (employer payer agreement) • Not applicable • Not available • Maximum USD 267

Abu Dhabi (Basic scheme)	• Physician (100 % at NNP; copayment of USD 5.5 + USD 2.7 if hospital specialist) • Diagnostics (100 % at NNP; copayment of USD 2.7 for lab and x-ray) • Medicines (30 % coinsurance; USD 409 limit) • Maternity inpatient (USD 136 copayment) • Maternity outpatients (USD 5.5 per consultation + USD 2.7 if hospital specialist)	• Follow up within 7 days • NA • Maximum USD408 p.a. • NA

Dubai	• Inpatient accommodation (20 %) • Hospital tests, treatment & surgery (20 %) • Emergency treatment (20 %) • Emergency transportation (20 %) • Maternity inpatients (10 %) • Outpatient specialist (20 % coinsurance) • Outpatient lab, scans, endoscopies (20 %) • Physiotherapy (20 %) • Medicines (30 %; USD3,133 limit) • Dental emergency (20 %)	• Maximum USD136 per encounter; USD272 p.a. • Same as above • Same as above • Same as above • NA • Follow up within 7 days • NA • NA • NA • NA

Oman	• Inpatient (USD 7,802p.a. limit and shared accommodation up to 30 days; no copayment) • Hospital transfer (USD 260/transfer limit; no copayment) • Basic policy outpatient (USD 1,300p.a. limit; 15 % in PPN) • Basic policy outpatient (USD 1,300p.a. limit; 30 % non PPN) • Medicines (10 % copayment generics only) • Dental (optional coverage; USD 1,300p.a. limit; 20 %) • Optical (optional coverage; USD 1,300p.a. limit; 20 %) • Maternity* (optional coverage; USD 7,802p.a. limit; 20 %)	• NA • NA • Maximum USD 52/visit • Maximum USD 13/visit • Maximum USD 13/visit • NA • NA • NA

Bahrain (Private Cooperative Health Insurance Program)	• Inpatient and day-care procedures (20 %) • Inpatient accommodation (shared room up to USD 26.60) • GP consultation (copayment USD 8) • Outpatient treatment (copayment USD 8) • Diagnostic treatment and service (20 %) • Medications (annual limit USD 1,330; 20 % generic; 40 % brand) • Outpatient physiotherapy (20 %)	• Maximum USD 133/admission; USD 266p.a. • NA • Follow up within 7 days • Follow up within 7 days • Maximum USD 26.6 per visit • NA • NA

* Application moratorium period of 280 days. NNP: Non-network provider

One distinguishing characteristic we found is the maximum physician consultation fees for CHIS, which is the only case in GCC. Maximum fees are set for a general practitioner or a specialist as first registrar doctor (USD 27–40), specialist or consultant as second registrar doctor (USD 53–80) and specialities and subspecialties of importance (USD 107) [110].

### 3.3 Institutional environment

This subsection examines the legal and institutional frameworks underpinning PHI, including the presence (or absence) of dedicated technical and prudential regulators, the scope of their authority and the evolution of regulatory structures.

Most GCC countries have a dedicated health insurance law that establishes PHI schemes (Table 7). Qatar is the exception, where the newly proposed PHI scheme was introduced as part of a wider health system law enacted in 2021, with further details established in implementing regulations [105], [114]. Similarly, Kuwait established a mandatory scheme for non-nationals (Dhaman) through a decision made by the Council of Ministers in 2010 and Oman through a regulatory decision by the Capital Market Authority (CMA) in 2019 [101], [115].

In terms of institutions regulating the market, we see two distinguishing models: (1) a twin-peaks model with separate technical and prudential regulators for PHI (UAE, Bahrain and Qatar) and (2) a sole regulator model where a single regulator supervises PHI (Saudi Arabia, Kuwait and Oman). Dubai and Abu Dhabi have their technical regulators for PHI: the Dubai Health Insurance Corporation and the Abu Dhabi Department of Health [109], [116]. The UAE Central Bank regulates all types of insurance at a federal level.

In the case of the UAE and Saudi Arabia, we have registered shifts from one model to another during the period of this review, with the latest one being in Saudi Arabia, where the Insurance Authority was established as a sole regulator in 2023 [117].

Considering market size, Bahrain and Qatar do not have specific health insurance regulators, but rather departments within the respective councils or ministries [104], [114]. Additionally, both have their central banks regulate and licence different classes of business lines, including PHI [113], [118]. The same can be said for Kuwait and Oman, although in Oman’s case, CMA could be considered as an integrated prudential and technical regulator, as it has prescribed technical requirements and a mandatory benefit package for the Dhamani scheme [112]. In Kuwait, the newly established Insurance Regulator Unit under the Department of Commerce is responsible for the supervision of individual and group PHI schemes for nationals and non-nationals [119]. Currently, there is no clarity on the role of the Kuwait Ministry of Health (MOH) in PHI regulation.

In conclusion, GCC countries employ various institutional arrangements in regulating PHI.

### 3.4 Policy framework

PHI policy frameworks in the GCC countries define who is covered, the objectives of PHI schemes and the type of insurance offered. Although most countries have achieved near-universal coverage for nationals, PHI schemes primarily serve non-national populations, with some systems beginning to expand coverage to nationals employed in the private sector. This subsection summarises the policy goals behind PHI schemes, the degree of integration with public health systems and the design features, such as benefit packages, annual limits and user charges, that determine access and financial protection.

#### 3.4.1 Population coverage and PHI objectives

As reported previously, all GCC countries have almost universal health coverage (UHC), either through government or PHI schemes. The latest data for 2021 show that the GCC average UHC index is 76 compared to 84 for OECD. Within GCC countries, the UAE has the highest index (80), followed by Kuwait (78), Qatar and Bahrain (76), Saudi Arabia (74) and Oman (70) [120].

In terms of population coverage, we see a unique approach driven by demographics where the government takes the responsibility of covering its nationals, whether through public or PHI coverage, or a combination of both.

In Abu Dhabi, nationals are covered through a government-funded programme called Thiqa, managed by the largest PHI company, Daman. In contrast, the rest of the eligible population is covered via basic and enhanced schemes. The basic scheme is a government-subsidised programme for labourers managed by Daman, while the enhanced scheme is for higher-skilled non-nationals underwritten by competing PHI companies [10], [11], [74].

Like Abu Dhabi, Dubai has implemented three schemes. The Insurance System of Advancing Health in Dubai (ISAHD) covers all residents of Dubai, while the remaining population is covered through the Enaya scheme (Dubai non-national government employees) and the Saada government-funded health programme (free healthcare at government providers for UAE nationals) [[72], [121], [122]]. Kuwait follows similar arrangements with plans for a separate scheme for nationals (PHI Company for Kuwaitis), and non-nationals (Dhaman scheme) [123].

Examining hybrid models, Saudi Arabia was the first in the region to introduce a mandatory PHI scheme in 1999, providing and regulating health care services exclusively for non-nationals [124]. In 2002, the scheme was extended to cover nationals in the private sector [[100], [125]]. Similarly, Oman requires both nationals and non-nationals in the private sector to purchase PHI from the market. In contrast, Bahrain has opted for a similar model, where PHI operates in parallel with the Health Insurance Fund (HIF), an independent organisation responsible for mandatory health insurance for nationals and non-nationals working in the government sector.

Regarding the objectives of the PHI schemes, all schemes have clear objectives for their mandate, ranging from ensuring coverage for the population to achieving sustainable health financing and improved services. We notice that the objectives of earlier established schemes relate more to coverage and regulatory aspects, with a focus on non-nationals, while the most recent ones are focusing more on integrated care and financial sustainability. For instance, Bahrain’s objectives are to develop a more integrated health system that provides high-quality services, while at the same time maintaining sustainable health financing and ensuring fair and competitive services to all parties involved in the scheme [126]. Qatar and Dubai have similar objectives to establish an integrated health system of high quality that is efficient and sustainable, and to implement and supervise compulsory health insurance [[105], [122]]. In contrast, Saudi Arabia, Abu Dhabi, Kuwait and Oman are more looking into the provision and regulation of health care, mainly for non-nationals and private sector employees [[101], [109], [112], [124]]. This is expected, as the trajectory of PHI development in the GCC spans a 25-year development period, reflecting different challenges and priorities.

#### 3.4.2 Terms of access

Terms of access vary significantly across the GCC, depending on (1) whether the coverage is tailored to specific population segments (e.g. blue collar or white collar), (2) whether the eligibility extends to nationals or not and (3) requirements to maintain reasonable coordination of benefits for nationals.

The annual limit varies significantly between different schemes. CHIS in Saudi Arabia provides the highest annual limit in the region, with a recent increase from USD 133,000 to USD 266,000, compared to Abu Dhabi’s basic coverage stipulating an annual limit of USD 68,064, and ISAHD in Dubai, where the prescribed minimum standard is a USD 40,840 annual limit [109], [110], [111].

The remaining countries, although they have not implemented their schemes, have revealed their annual limit, except for Qatar and Kuwait, where no official information is available.

For Bahrain, market intelligence reports from a tender issued for the Private Cooperative Health Insurance Programme to select qualified insurer(s) reveal a financial annual limit of USD 39,894, while Oman plans to apply separate annual limits for inpatient and outpatient (USD 7,797 and USD 1,298, respectively) [112], [113].

These variations reflect the distinct contexts of the country’s health insurance system and the various population segments it serves.

In terms of pre-existing conditions and exclusions, we observe a more common approach, characterised by an extensive list of exclusions and measures to address adverse selection. CHIS stipulates a list of 27 exclusions, and a unified medical declaration form is utilised in cases when new beneficiaries are added to a pool or, for small and medium employers, to declare the presence or absence of conditions from a list. ISAHD applies a similar approach, with the addition of a ‘first entrance’ rule, which allows for exclusion for the first 6 months. There is a list of 42 exclusions in addition to services stemming from 13 different scenarios outside of the scope of health insurance [127]. Abu Dhabi applies a 6-month moratorium on first-time entries and excludes 43 services. In both cases, medical declaration forms are used.

Bahrain has not provided further details of the forthcoming scheme. However, the law requires PHI companies to disclose all relevant information to beneficiaries, including the scope of coverage, network, benefits, coinsurance, prior approvals and other pertinent details. Similarly, Qatar provides no further details about the terms of access and user charges. However, the law stipulates that insurance companies cannot reject coverage for eligible beneficiaries if they are compliant with the law and cannot impose a waiting period for pre-existing conditions. Kuwait has not published documentation on the terms of access for Afya and Dhaman. However, based on the concept and objectives, no significant restrictions are expected for Dhaman, beyond provider network requirements and referral to MOH tertiary care. At the same time, Afya members will still have access to public provision of care in addition to private care [[9], [119]]. Oman has published the terms of condition of the Dhamani scheme, which stipulate pre-existing chronic conditions and 42 exclusions [112].

#### 3.4.3 PHI types

The policy framework has influenced the role of PHI in the region (Table 8). In all GCC countries, PHI will have a mandatory role for some, if not all, population segments, exercised under regulatory supervision. This suggests that PHI is becoming the preferred mechanism for healthcare financing among GCC governments, and we can expect a larger role for PHI in the future.

**Table 7:** Overview of institutional arrangements for PHI regulation in GCC countries.

	Prudential regulators		Technical regulators	
	Integrated prudential regulator	Dedicated insurance regulator	Health authority	Health insurance regulator
Bahrain	Bahrain Central Bank		Supreme Council of Health	
Kuwait		Ministry of Commerce		
Oman		Capital Market Authority		
Qatar	Qatar Central Bank		Ministry of Public Health	
Saudi Arabia	Saudi Central Bank (SAMA) *	Insurance Authority		Council of Health Insurance*
Abu Dhabi	UAE Central Bank	Insurance Authority**		
Dubai	UAE Central Bank	Insurance Authority**		

* Saudi Arabia's Council of Ministers decision no.85/2023 transferred mandate from Council of Health Insurance and SAMA to the newly established Insurance Authority.

** Replaced by the UAE Central Bank as per Decretal Federal Law No. 24 of 2020 on the Amendment of certain provision of the Federal Law No.6 of 2007.

While we have not registered an instance where PHI plays a substitutive role, we foresee the need to consider this model as national population demographics change in the region through ageing, drawing from experiences in Germany and Chile [2].

In most cases, PHI in GCC also plays a supplementary role, offering faster access to services and more choice through the private sector, followed by a complementary role that provides additional coverage of services on top of EBP, with limited offerings for co-payments.

In Saudi Arabia, supplementary and complementary roles are becoming an important growth factor for the market, with medium and large employers topping up EBP or nationals purchasing additional coverage voluntarily [128]. In the UAE, the enhanced scheme for non-nationals is considered a supplementary and complementary PHI beyond the basic EBP coverage [10], [11]. PHI in Qatar gained the long-sought role of a mandatory scheme in 2021 with the promulgation of Law No. 22 of 2021, which delineates the roles of the government and employers in financing healthcare. As a result, nationals will remain in the public healthcare system, while non-nationals will be covered by PHI [105]. This may potentially open up opportunities to provide supplementary and complementary PHI products in the market.

In Bahrain, PHI plays both a mandatory and supplementary role, encompassing both nationals and non-nationals. However, nationals are entitled to subsidised government-optional health insurance through HIF (up to 60 %) [104].

When it comes to specialisation of PHI companies, the GCC market is dominated by general insurers, with only a handful of specialised health insurers in Saudi Arabia (three) and the UAE and Kuwait (one each). This comes as no surprise, as the size and maturity of these markets enable mono insurance companies to achieve sufficient economies of scale in their operations and reasonable profitability.

### 3.5 Market characteristics

The structure and performance of PHI markets differ substantially across the GCC (Table 9). Markets in several countries are highly concentrated, dominated by a small number of local insurers. In contrast, others have introduced measures to increase competition, such as allowing foreign insurers to enter and implementing risk-sharing mechanisms. This subsection reviews market structure, levels of concentration, premium-setting practices and performance indicators, including profitability and loss ratios. It also highlights how market characteristics can influence competition, consumer choice and the bargaining power of insurers with healthcare providers.

**Table 8:** Summary of PHI schemes in GCC countries.

**Country**	**Objectives**	**Description**	**PHI type and population**	**Terms of access**	**Specialized vs. non-specialized**
Bahrain	· Integrated health system providing high quality services · Sustainable health financing · Fairness and competition	Mandatory health insurance scheme with single payer (HIF) and PHI covering different categories	Mandatory (expatriates) Supplementary (nationals and expatriates) Voluntary (rest of market)	· Annual limit $39,984 · Pre-existing/chronic condition limit ($5,319) · Waiting time: 6 months*	NA

Kuwait	Afya Dhaman · Reduce waiting times in public · Reduce government spending · Improve services for expatriates	PHI purchased by government for Kuwaiti retirees Mandatory HMO type PHI for expatriates	Mandatory (Dhaman for expatriates) Supplementary (Afya for retirees) Voluntary (rest of market)	Dhaman: PHI: · network restrictions, copayments · standard exclusions (war, HIV, Hep B&C, suicide, other); medical questionnaire and tests depending on age and coverage	One specialized

Oman	NA	Mandatory PHI covering private sector employees	Mandatory PHI for expats and national in private sector	· Annual limit $7,797 (inpatient) and $1,298 (outpatient) · Pre-existing/chronic conditions (outpatient) · 42 exclusions	NA

Qatar	· Integrated health system providing high quality services that is sustainable · Implement and supervise mandatory health insurance	Mandatory PHI covering expatriates	Mandatory for expatriates in private sector	· Not published yet except essential benefit package and exclusions list	NA

Saudi Arabia	· Provide and regulate health care provision to expatriates in Saudi Arabia	Mandatory PHI covering different population categories	Mandatory for expatriates and nationals in private sector	· Annual limit: $133 k/$266 k** · Pre-existing conditions · 27 exclusions	Three specialized

Abu Dhabi	· Cover non-UAE national residents and their families in the Emirate of Abu Dhabi	Mandatory PHI covering non-nationals, later expanded to nationals	Mandatory for expatriates and nationals	Basic · $68,064 annual limit · Pre-insurance congenital deficiency or defect (not life threatening) · First-time entry for high-cost conditions 6 m moratorium · 43 excluded services which could be covered via enhanced coverage · 13 non-insurable scenarios (injuries from accidents, work-related injuries, substance abuse, epidemics, HIV/AIDS)	One specialized

Dubai	· Integrated health system providing high quality services · Sustainable health financing · Attract investments and be competitive · Ensure all parties’ rights	Mandatory PHI for expatriates	Mandatory for expatriates in private sector	· Annual limit: $40,840 · Pre-existing conditions · 27 exclusions	

* Except for maternity.

** Small and Medium Enterprise/Large employer NA: Not Available.

In terms of structure, almost exclusively all current and prospective mandatory PHI schemes are or will be operated by for-profit insurance companies, except in cases where there are transfers by governments for national schemes operated via TPA (Thiqa in Abu Dhabi, and Enaya and Saada in Dubai), or cases where a HIF is a risk-bearer entity (Bahrain). Qatar, until the end of 2015, had a similar non-profit entity that acted as a single payer for the entire population [[10], [86], [104], [121]].

Local insurance companies have dominated the GCC market due to protectionist regulation in the past decades, which allowed licences only to local companies. Most international companies are located in Bahrain, while the fewest are found in Saudi Arabia. However, the real picture of international versus local companies is blurred because in some countries, governments have allowed offshore operations with business-friendly regulations (Bahrain, Qatar and Dubai) [[97], [113], [118], [119], [128]].

Most of the PHI markets show high or moderate levels of market concentration. The highest C3 concentration is registered in Kuwait, at 96 %, followed by Oman and Saudi Arabia, at 85 % and 80 %, respectively. However, the case of Kuwait is entirely due to the underwriting of the Afya scheme by a single company with a market share of 85 % [97]. Bahrain, Abu Dhabi and Qatar exhibit moderate levels of concentration, with C3 at 53 %, 64 % and 57 %, respectively. However, in the case of Abu Dhabi, one company accounted for almost half of all PHI claims in the market. This may explain the relatively low number of international players in these markets, despite efforts by governments to reduce regulatory barriers and enable new entrants into the market, such as the allowance of the first foreign branch licensing in Saudi Arabia [[118], [128]].

Market buyers, in most cases, are employers buying group insurance, either mandated by law or voluntary. Where there is no compulsory mandate, there is a minimum number of members for group insurance, usually ranging from ten to 20 members, depending on the country. In Saudi Arabia, small and medium enterprises are the dominant group, with the 1–9 employees group being the largest (80.9 % of all policies in 2019) [129].

Market conduct is primarily limited to premium-setting elements, as there is no data or literature on cream-skimming in the region, and financial equalisation schemes have not been registered. Wherever PHI is mandatory, due to large pools, the premium setting is community-rated with some variations. For instance, in Saudi Arabia, employers with less than 250 employees may be required to complete a medical declaration form to assess risk pools. Similar arrangements are applied in Dubai (ISAHD) and Abu Dhabi (enhanced scheme) [[130], [131]]. However, regulators have recently introduced regulations to address cases of catastrophic health expenditure through different forms of risk sharing. In Saudi Arabia, an outlier risk-sharing fund called Dhaman was established to cover cases above annual limits, while Dubai established condition-specific risk sharing programme called Basmah. In addition, we have registered a recent policy measure to pool different client pools into one in Saudi Arabia albeit through brokers rather than payers [[132], [133], [134]].

Moral hazard measures primarily rely on coverage limits, user charges and prior authorizations. In contrast, most supply-side measures are more pertinent to mandatory schemes, where regulators have introduced new payment systems, disease management, value-based healthcare, and clinical guidelines (e.g. Saudi Arabia and UAE).

**Fig. 2:**
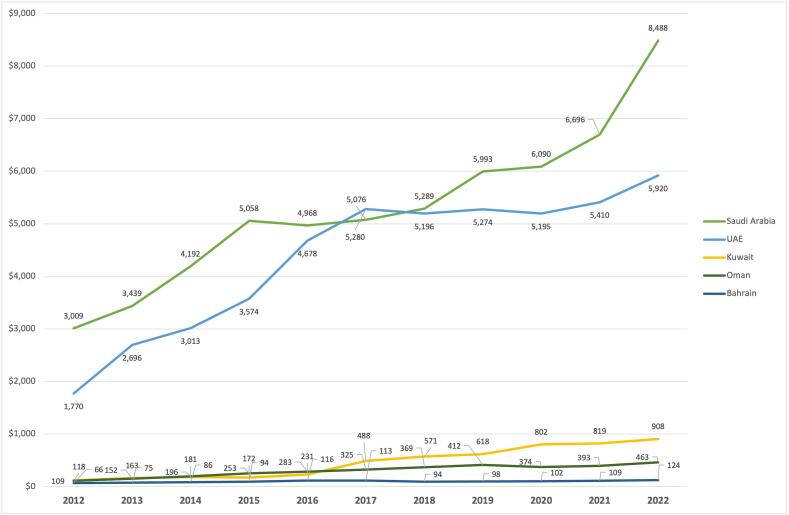
Private Health Insurance written premium by countries (USD millions) (2012–2022).

The GCC PHI market performance has been highly volatile over the past 20 years, reflecting regulatory changes and macroeconomic events such as oil price shock and COVID-19 pandemic. In terms of growth, all markets have experienced growth over the last 15 years, with Kuwait witnessing the highest, followed by Oman, the UAE, Saudi Arabia, and Bahrain (19.7 %, 12.5 %, 8.9 %, 8.5 % and 0.3 %, respectively) (Fig. 2). For Qatar, we found data only for a limited and outdated period (2009–2014, with a CAGR of 16.4 %).

The pace of growth increased post-COVID-19, with Saudi Arabia reporting the strongest growth (27 % from 2021 to 2022). PHI markets in 2022 have performed well with a reasonable loss and profit ratio, except for Oman, which reported a high loss ratio and lower profits. An efficiency study conducted in 2020 using Data Envelope Analysis on 60 GCC insurance companies, concluded that only ten out of 60 were technically efficient, and only two insurers were consistently efficient within the research period (2016–2019) [135]. Related to this, two studies using the same methodology have been critical of the technical efficiency of insurance companies in Saudi Arabia and Oman, suggesting room for improvement in terms of efficiency [[92], [136]].

Although an important performance indicator, PHI administrative costs were not reported, except in the cases of Qatar and Dubai, where the administrative costs in 2014 were 26.3 % and 13 %, respectively. More recent information on Qatar is not available, while Dubai has stopped reporting this information since 2014. It is strongly advised that GCC regulators monitor this performance indicator and, where possible, report it to the public, especially in cases where PHI schemes are mandatory employer-based ones.

## 4 Discussion

Three cross-cutting themes emerged across the GCC countries, each with significant policy implications. First, PHI schemes are expanding rapidly but remain segmented by nationality and employment sector, raising concerns about long-term equity. Unlike countries such as France, where complementary PHI offsets cost-sharing, or Germany and Chile, where substitutive models allow some groups to opt out of public coverage, GCC schemes are primarily built around employer-based coverage for non-nationals and, increasingly, nationals in the private sector [7]. This segmentation risks entrenching a two-tier system if not carefully regulated.

Second, regulatory capacity varies widely. Some countries have adopted ‘twin peaks’ models separating technical and prudential oversight, while others rely on a single regulator. Experience from more mature PHI markets shows that robust, well-coordinated regulation is critical to consumer protection, competition and cost control, particularly as PHI expands beyond expatriate-only coverage. Countries such as Bahrain and Saudi Arabia have begun implementing measures to strengthen oversight, potentially allowing foreign insurers to enter, but gaps remain.

Third, PHI markets are highly concentrated and dominated by local insurers. While concentration can strengthen insurers’ bargaining power with providers and enable economies of scale, it may also reduce consumer choice and create barriers for new entrants. Striking the right balance between market stability and competition will be essential.

These themes are not unique to the GCC and echo challenges faced by other middle- and high-income countries where PHI plays a significant role in healthcare financing. The experience of the Netherlands and Switzerland, where PHI functions as a compulsory, heavily regulated mechanism for universal coverage, illustrates how regulatory design can shape outcomes in access, equity and cost control [2]. Whether strong or weak regulatory frameworks accompany the expansion of PHI in the GCC will ultimately determine the sustainability and inclusiveness of these health systems in the long term.

PHI in GCC countries operate in a complex health system context, interplaying between different health systems, healthcare financing schemes and eligibilities due to legacy systems and policy path dependencies [8], [9], [10], [11], [12], [13], [76]. Compared to other developed health systems, what distinguishes the GCC is the relatively short period over which during this transformation has occurred and the unique demographic circumstances [2], [14].

As PHI is expanding and taking more importance in the overall health system financing, governments will need to plan further policy measures as more nationals become part of PHI schemes [[97], [113], [118], [119], [128]]. This will require policies on harmonising benefits between PHI and public schemes, and the ability of nationals to move from one scheme to another scheme [11], [12], [46].

The institutional environment exhibits robust regulation, encompassing both technical and prudential oversight of PHI, particularly when operating under a mandate. In most cases, we see a twin-peaks regulatory model, with PHI being regulated by both technical and prudential regulators, except in Saudi Arabia and Oman, where sole regulators exist [[117], [137]]. This is not different from the regulatory landscape in Europe, where various regulators concurrently or exclusively regulate PHI, depending on their role [138]. Nevertheless, regardless of the model of choice, it is essential for countries that mandate PHI to establish robust regulatory regimes and institutions to achieve proper market conduct and performance.

The GCC PHI policy framework is becoming more aligned in terms of mandating PHI, whether it is already operational or has been promulgated in laws and regulations. In some instances, PHI’s mandatory role focuses on non-nationals (Qatar, Dubai and Abu Dhabi), while in others, the framework covers nationals in the private sector as well (Saudi Arabia, Oman, Bahrain) [[104], [109], [114], [115], [122], [124]]. It remains to be seen which direction Kuwait will take. Compared to other PHI schemes, Saudi Arabia has mandated a higher annual financial limit and a relatively more generous benefit package, reflecting the fact that it is the only scheme currently covering nationals and needs to maintain harmonisation with public benefits (Table 8). This is also reflected in user charges and access provisions (Table 6). Oman and Bahrain have made similar arrangements for additional coverage for nationals, with Bahrain going further by subsidising this coverage [104]. However, these schemes are not yet operational, and it remains to be seen how they will look in practice. Incoherent and incomplete policy frameworks may affect GCC countries’ health system reform efforts and the achievement of their strategic objectives.

Market structure findings appear to be more homogeneous (Table 9). Structure-wise, all schemes are operated or will be operated by for-profit PHI companies, except in Bahrain, where a mix of government HIF and PHI will be used. High market concentration and the significant presence of local companies are two other characteristics of the GCC market. This, combined with potential behavioural barriers (pricing strategies) and demand-side barriers (brand loyalty, high switching costs and information asymmetry), could further prohibit new entries into the market.

**Table 9:** Overview of market characteristics of PHI in GCC countries (2022 or latest).

Country	Structure			Conduct	Performance			
	# of insurers (local/foreign)	Type of insurer	C3 concentration	Premium setting	Coverage	Average premium growth 2012–2022 (CAGR)	Loss ratio	Profit ratio
Bahrain	21/10	For-profit & non-profit	53 %	Risk rated	NA	0.3 %	72 %	1.39
Kuwait		For-profit	96 %	Risk rated	NA	19.7 %	NA	5.91^a^
Oman	7/7	For-profit	84.5 %	Risk rated	7.9 %	12.5 %	157 %	0.67
Qatar^b^	6/10	For-profit	57 %^b^	Risk rated	NA	9.2 %	NA	1.36
Saudi Arabia	22/1	For-profit	80.2 % c	Risk rated & Community rated	32 %	8.5 %	85 %	1.40
UAE	Not applicable	For-profit	Not applicable	Risk rated & Community rated	Not applicable	8.9 %	83 %	1.27
Dubai (ISAHD)	13/3	For-profit		Risk rated & Community rated	Not applicable	n/a	n/a	n/a
Abu Dhabi (Enhanced)	42	For-profit	63.7 % d	Risk rated & Community rated	80 % e	n/a	n/a	n/a

a 2017.

b Qatar NHA 2009–2014.

c 2020.

d Enhanced scheme 2017.

e Includes basic scheme.

Nevertheless, these barriers do not necessarily translate to a less competitive environment, as high levels of concentration may provide significant economies of scale for the top insurance companies, in the context of a free market where moral hazard and supplier-induced demand fuel medical inflation, this may enhance PHI’s bargaining power over healthcare providers to contain unwarranted cost escalation.

There is very little information available on market conduct, except for premium setting, which is mostly community-rated in the UAE and Saudi Arabia (for mandatory schemes) and experience-rated in other countries (for voluntary PHI).

Examining PHI performance reveals improvements in all aspects investigated (coverage, growth, loss and profit) (Table 9). However, there is still room for improvement, especially in the case of Oman, where the market operates at a loss despite having the second-largest growth in the region in the last ten years [97]. As PHI becomes mandatory in the remaining countries, it is expected that performance will improve. Nevertheless, performance should be the primary objective of regulators as part of governments’ endeavours to achieve sustainability in the long run. In addition, employers who pay for coverage but do not play a significant role in shaping the market may need to be enabled to exert more influence on PHI.

These findings have relevance beyond the GCC. Countries considering or expanding PHI schemes, particularly those that are employer-based or segmented by population group, must anticipate the equity risks of parallel systems. Similarly, the GCC experience underscores the importance of robust technical and prudential regulation of PHI markets, as well as close alignment with UHC objectives. Future studies should benchmark GCC PHI systems against international comparators to better understand which regulatory and design choices most effectively promote equitable and sustainable health system outcomes.

Policy-makers in the GCC face two key imperatives. First, as PHI expands, there is a need to ensure that eligibility rules, benefit packages and user charges do not exacerbate inequities between nationals and non-nationals or between public and private sector employees. Second, PHI growth must be fiscally and institutionally sustainable. This will require more transparent reporting of administrative costs and performance indicators, robust regulation of market conduct (including premium setting) and better integration of PHI schemes with public financing mechanisms. Without these measures, PHI risks becoming a parallel system that undermines the objectives of universal coverage and cost control.

This review has several limitations. Although we conducted a comprehensive search, some relevant grey literature that is not publicly available may have been missed. In addition, the review was completed at the end of 2023, and new developments and regulations may have emerged since then. We were also unable to engage key country experts due to the large number of countries covered, which may have limited our contextual insights. Finally, we did not assess outcomes such as access, efficiency or equity, nor did we benchmark PHI schemes against international comparators. Both areas would require extensive data collection and a dedicated analytical approach. By consolidating evidence on the structure and evolution of PHI in the GCC, however, this review sets the stage for future research to explore these critical questions in greater depth.

## 5 Conclusion

PHI in GCC countries is on an upwards trajectory, increasing in prominence and importance in the region’s healthcare financing. Although similar in terms of policy frameworks, scope and role, this review highlights distinctive features in each country, shaped by the health system context and policy path dependency [139]. While PHI is maturing and subject to increasingly robust technical and prudential regulations, countries remain at different levels of implementation and market maturity. This variation offers governments the opportunity to test and refine their policy frameworks based on evidence and lessons learned.

As PHI becomes a larger share of THE, governments must focus not only on market growth but also on performance, equity and sustainability. This will require stronger regulation of market conduct, closer integration of PHI with public financing mechanisms and mechanisms to engage employers more effectively in shaping policy, given their central role in most schemes. Without these measures, PHI risks entrenching segmentation between nationals and non-nationals and undermining UHC and cost-control objectives.

## Financial support

No financial support was received for this study.

## CRediT authorship contribution statement

**Husein Reka:** Writing – original draft, Visualization, Resources, Methodology, Formal analysis, Data curation, Conceptualization. **Robin van Kessel:** Writing – review & editing, Writing – original draft, Validation, Supervision, Methodology, Formal analysis, Data curation, Conceptualization. **Elias Mossialos:** Writing – review & editing, Validation, Supervision, Methodology, Conceptualization. **Wim Groot:** Writing – review & editing, Validation, Supervision, Methodology, Conceptualization. **Milena Pavlova:** Writing – review & editing, Writing – original draft, Validation, Supervision, Methodology, Conceptualization.

## Declaration of competing interest

The authors declare that they have no known competing financial interests or personal relationships that could have appeared to influence the work reported in this paper.

## Figures and Tables

**Table:** 

**Database**	**Search string**	**Result**
Pubmed	(((“Insurance”[MeSH Terms] OR “insurance*”[Title/Abstract]) AND (“Bahrain”[Text Word] OR “Bahraini”[Text Word] OR “Kuwait”[Text Word] OR “Kuwaiti”[Text Word] OR “Oman”[Text Word] OR “Omani”[Text Word] OR “Qatar”[Text Word] OR “Qatari”[Text Word] OR “Saudi Arabia”[Text Word] OR “KSA”[Text Word] OR “Saudi”[Text Word] OR “United Arab Emirates”[Text Word] OR “UAE”[Text Word] OR “Emirati”[Text Word] OR “Gulf States”[Text Word] OR “Dubai”[Text Word] OR “Abu Dhabi”[Text Word])) OR ((“Bahrain”[Text Word] OR “Bahraini”[Text Word] OR “Kuwait”[Text Word] OR “Kuwaiti”[Text Word] OR “Oman”[Text Word] OR “Omani”[Text Word] OR “Qatar”[Text Word] OR “Qatari”[Text Word] OR “Saudi Arabia”[Text Word] OR “KSA”[Text Word] OR “Saudi”[Text Word] OR “United Arab Emirates”[Text Word] OR “UAE”[Text Word] OR “Emirati”[Text Word] OR “Gulf States”[Text Word] OR “Dubai”[Text Word] OR “Abu Dhabi”[Text Word]) AND (“health*”[Title/Abstract] AND (“expenditure*”[Title/Abstract] OR “financ*”[Title/Abstract] OR “payment*”[Title/Abstract] OR “cost*”[Title/Abstract] OR “budget*”[Title/Abstract] OR “regulat*”[Title/Abstract])) AND (“reform*”[Title/Abstract] OR “transform*”[Title/Abstract] OR “chang*”[Title/Abstract] OR “strateg*”[Title/Abstract] OR “shift*”[Title/Abstract] OR “impact*”[Title/Abstract]))) AND (1999:2023[pdat])	946

Embase	1 Insurance/ or insurance*.ti,ab,kw.	1,368
	2 (Bahrain or Bahraini or Kuwait or Kuwaiti or Oman or Omani or Qatar or Qatari or “Saudi Arabia” or Saudi or KSA or “United Arab Emirates” or UAE or Emirati or “Gulf States” or Dubai or “Abu Dhabi”).ti,ab,hw.	
	3 1 and 2	
	4 (health* and (expenditure* or financ* or payment* or cost* or budget* or regulat*)).ti,ab,kw.	
	5 (reform* or transform* or chang* or strateg* or shift* or impact*).ti,ab,kw.	
	6 2 and 4 and 5	
	7 3 or 6	
	8 limit 7 to yr=“1999 −Current”	

Web of Science	1: TS=(insurance*)	1,368
	2: TS=(Bahrain OR Bahraini OR Kuwait OR Kuwaiti OR Oman OR Omani OR Qatar OR Qatari OR “Saudi Arabia” OR KSA OR Saudi OR “United Arab Emirates” OR UAE OR Emirati OR “Gulf States” OR Dubai OR “Abu Dhabi”)	
	3: #1 AND #2	
	4: TS=(health* AND (expenditure* OR financ* OR payment* OR cost* OR budget* OR regulat*))	
	5: TS=(reform* OR transform* OR chang* OR strateg* OR shift* OR impact*)	
	6: #2 AND #4 AND #5	
	7: #3 OR #6	
	8: #3 OR #6	

Scopus	((TITLE-ABS-KEY ({Insurance} OR {insurance*})) AND (TITLE-ABS-KEY ({Bahrain} OR {Bahraini} OR {Kuwait} OR {Kuwaiti} OR {Oman} OR {Omani} OR {Qatar} OR {Qatari} OR {Saudi Arabia} OR {KSA} OR {Saudi} OR {United Arab Emirates} OR {UAE} OR {Emirati} OR {Gulf States} OR {Dubai} OR {Abu Dhabi}))) OR ((TITLE-ABS-KEY ({Bahrain} OR {Bahraini} OR {Kuwait} OR {Kuwaiti} OR {Oman} OR {Omani} OR {Qatar} OR {Qatari} OR {Saudi Arabia} OR {KSA} OR {Saudi} OR {United Arab Emirates} OR {UAE} OR {Emirati} OR {Gulf States} OR {Dubai} OR {Abu Dhabi})) AND (TITLE-ABS-KEY ({health}) AND (TITLE-ABS-KEY ({expenditure} OR [5] OR {payment} OR {cost} OR {budget} OR))) AND (TITLE-ABS-KEY ({reform} OR {transform} OR {chang} OR {strateg} OR {shift} OR {impact}))) AND PUBYEAR > 1998 AND PUBYEAR < 2024 AND (LIMIT-TO (LANGUAGE, “English”) OR LIMIT-TO (LANGUAGE, “Arabic”))	971

Google Scholar	“private health insurance”|“health insurance”|insurance Qatar|Qatari	300
	“private health insurance”|“health insurance”|insurance Bahrain|Bahraini	300
	“private health insurance”|“health insurance”|insurance Kuwait|Kuwaiti	300
	“private health insurance”|“health insurance”|insurance “Saudi Arabia”|Saudi|KSA	300
	“private health insurance”|“health insurance”|insurance “United Arab Emirates”|UAE|Emirati	300
	“private health insurance”|“health insurance”|insurance Oman|Omani	300

**Table:** 

**Country**	**Entities**
Bahrain	Supreme Council of Health
	Ministry of Heath
	Central Bank of Bahrain
	Social Insurance Organization
	AXCO Market Report and Database

Kuwait	Ministry of Health
	Department of Commerce – Insurance Regulatory Unit
	Kuwait Insurance Federation
	Central Bank of Kuwait
	Kuwait Government Online Legal Portal
	AXCO Market Report and Database

Oman	Ministry of Health
	Capital Market Authority
	Oman Insurance Association
	Ministry of Justice and Legal Affairs
	AXCO Market Report and Database

Qatar	Ministry of Public Health
	Qatar Financial Regulatory Authority
	Qatar Central Bank
	Al Meezan Legal Portal Ministry of Justice
	AXCO Market Report and Database

Saudi Arabia	Ministry of Health
	Saudi Central Bank (SAMA)
	Council of Health Insurance
	Unified National Platform
	AXCO Market Report and Database

UAE	Ministry of Health and Prevention
	Department of Health Abu Dhabi
	Dubai Health Authority
	Insurance System for Advancing Health Care in Dubai (ISAHD)
	Legal Portal of UAE
	UAE Central Bank
	AXCO Market Report and Database

**Table:** 

**Country**	**Search strategy**
Abu Dhabi	Abu Dhabi DOH website was screened for potential material (https://www.doh.gov.ae/en) under resources tab.
	Following sections of resources page were searched manually or via embedded web search engine within https://www.doh.gov.ae/en either by category (health insurance) or keyword (insurance):
	- Publications
	- Open Data
	- Circulars
	- Guidelines
	- Policies
	- Standards
	- Lists & Tools
	- Reporting
	- Jawda Quality Metrics
	- Health Information Exchange Standards

Dubai	Insurance System for Advancing Health Care in Dubai (ISAHD) website was manually searched for relevant material (https://www.isahd.ae/Home/LawAndRegulations)
	Dubai Health Authority Policy and Regulations portal was searched for Insurance as a keyword (https://www.dha.gov.ae/en/licensing-regulations-policies)

UAE	Ministry of Health and Prevention open data portal did not return any result on insurance keyword search (https://mohap.gov.ae/en/open-data/mohap-open-data)
	Law and legislations portal (https://elaws.moj.gov.ae/indexEN.aspx) was searched by using “insurance” keyword
	UAE Central Bank Insurance regulation page was searched for laws, regulation and other (https://rulebook.centralbank.ae/en/rulebook/insurance)
	AXCO Database UAE Life and Benefits report acquired

Bahrain	Supreme Council of Health website Rules and Regulations page was scanned (https://www.sch.org.bh/en/rules-and-regulations.html)
	Ministry of Health website Law and Health Regulations webpage was scanned (https://www.moh.gov.bh/Ministry/HealthLawNew)
	Legislation and Legal Opinion Commission search engine using insurance as a keyword was used (https://www.lloc.gov.bh/Legislation/English)
	Central Bank of Bahrain search engine was used using insurance as a keyword (https://cbben.thomsonreuters.com/rulebook/cbb-regulations-and-resolutions)
	AXCO Database Bahrain Life and Benefits report acquired

Kuwait	Kuwait Central Bank website was searched for insurance laws/regulations (https://www.cbk.gov.kw/en)
	Kuwait Official Gazette is restricted to subscribers only (https://kuwaitalyawm.media.gov.kw/Online/)
	Kuwait Insurance Federation laws and regulations webpage was searched https://kif-kw.com/Pdfs/%D8%A7%D9%84 %D9%82 %D9%88 %D8%A7%D9%86 %D9%8A%D9%86-%D9%88 %D8%A7%D9%84 %D8%AA%D8%B4%D8%B1%D9%8A%D8%B9%D8%A7%D8%AA-18
	Kuwait Ministry of Commerce and Industry Legal and Regulations webpage did not yield any results when searched by keyword insurance (https://www.moci.gov.kw/ar/about-us/deputies-and-departments/assisstant-agency-for-corporate-affairs-and-commercial-licenses/insurance-department/#)
	Kuwait Ministry of Health website was searched but no relevant document was found
	AXCO Database Kuqait Life and Benefits report acquired

Oman	Capital Market Authority Legal Encyclopedia was reviewed for all documents in insurance category (https://e.cma.gov.om/LegislationEncyclopedia/)
	MOH Policies and Guidelines were searched (https://www.moh.gov.om/en/-37)
	Ministry of Justice and Legal Affairs portal was searched by using insurance as a keyword (https://mjla.gov.om/eng/index.aspx#)
	Oman Insurance Association was searched for relevant documentation (https://omaninsuranceassociation.com/)
	AXCO Database Oman Life and Benefits report acquired

Qatar	Meezan platform of MOJ for Qatari legislation was searched used keyword تأمين صحي (https://www.almeezan.qa/AllLegislationsSearch.aspx?language = ar)
	MOPH publications website was searched under the category of Health Financing and Insurance Department (https://www.moph.gov.qa/english/mediacenter/Publications/Pages/default.aspx#)
	Qatar Financial Centre Regulatory Authority publication website was scanned for material (https://qfcra-en.thomsonreuters.com/)
	Qatar Central Bank (https://www.qcb.gov.qa/EN/Pages/Publication.aspx?IndexSelect = 0)
	AXCO Database Qatar Life and Benefits report acquired

Saudi Arabia	Central Bank search engine was used using "insurance" as a keyword (https://www.sama.gov.sa/en-us/insurance/pages/circulars.aspx) for circulars. Law and Regulations of Insurance sector were retrieved from the relevant webpage of SAMA
	Council of Health Insurance website was scanned for regulations, policies, decisions, white papers, annual reports and any other document (https://chi.gov.sa/en/pages/default.aspx)
	United National Platform was searched for Laws and Regulations (https://www.my.gov.sa/wps/portal/snp/aboutksa/rulesandRegulations) using insurance a keyword
	MOH Saudi Arabia publications website screened (https://www.moh.gov.sa/en/Ministry/MediaCenter/Publications/Pages/default.aspx)
	AXCO Database KSA Life and Benefits report acquired

**Table:** 

Reviewer	HR & RVK
Date	20 January 2024

**Paper information:**	
Name	HPR67 Ejadah: Implementation of Value-Based Healthcare in Emirate of Dubai
Author(s)	M. Farghaly, L. A. Alrustamani, A. I. A. Aladawy, E. A. M. A. Suliman and B. Mukherjee
Year of publication	2023
Country/Emirate	Dubai

Full citation information	Farghaly M, Alrustamani LA, Aladawy AIA, Suliman EAMA, Mukherjee B. HPR67 Ejadah: Implementation of Value-Based Healthcare in Emirate of Dubai. Value in Health. 2023;26(6 Supplement):S223 https://dx.https://doi.org/10.1016/j.jval.2023.03.1214

Aim/purpose	Dubai Health Authority (DHA) is pursuing the implementation of Ejadah, a performance management framework designed to help transform current practice from Volume Based Care to Value Based Health Care. The aim is to develop and implement Key Performance Indicators (KPIs) according to evidence-based clinical practice guidelines to support healthcare professionals and healthcare systems adopt practices to optimize patient outcomes and reduce expenditure. To facilitate international benchmarking in compliance with Dubai Health System Performance Assessment Framework (Dubai Health SPA) that is developed collaboratively with OECD
Methods	Descriptive study
Record #	136

**Inclusion/Exclusion Criteria**	
Population	Private Health Insurance in Dubai
Concept	Transformation/Reforms of Dubai Private Health Insurance Scheme
Context	GCC Health System

**Review framework domains studied in literature on private health insurance transformation**	
Health System Framework (e.g. eligibility, sector size, user charges)	
Institutional environment (e.g. legal framework, regulation)	
Private Health Insurance policy framework (e.g. objective, population coverage, system description, types of coverage	
Market structure, conduct and performance (e.g. market concentration, barriers to entry, local vs. international, vertical integration, value-based health care)	Value-Based Health Care initiative

**Key findings related to scoping review question**	
Health System framework	
Eligibility	
Sector size	
User charges	

Institutional environment	
Legal framework	
Material regulation	
Prudential regulation	

Private Health Insurance policy framework	
Objective	
Population coverage	
System description	
Coverage type	

Market structure	
Concentration	
Barriers to entry	
Local vs international	
Vertical Integration	

Value Based Health Care	DHA introduced a VBHC. Ejadah program is planned to be launched in phases. The first phase was launched in November 2022 and included six indications: T2DM, Asthma, URTI, Maternal care, Low back pain & GERD. Training of the payors & providers was conducted on the KPIs for these indications. Baseline analysis and international benchmarking of KPIs after first six months of the launch of the program will be presented to show Dubai health sector's performance. Conclusion(s): Ejadah will improve government oversight of healthcare services enhancing efficiency of health care, improve clinical outcomes as well as patient experience. It will further establish Dubai's position as a global medical destination by introducing value-based, integrated high-quality service delivery system
