# Supplementary material for: Private health insurance in Gulf Cooperation Council countries: A scoping review
Source: Health Policy Open. 2025 Dec 5;10:100157. doi: 10.1016/j.hpopen.2025.100157 (PMC12765444; doi:10.1016/j.hpopen.2025.100157)
Supplement: Supplementary Data 1 [file mmc1.pdf]

Private Health Insurance transformation in the context of health reforms in GCC countries: a scoping review

Grey literature extraction

Authors

Husein Reka[1] Robin van Kessel[2] Elias Mossialos[3] Wim Grooth[4] Milena Pavlova[5]

1. Care and Public Health Research Institute, Health Services Research, University of Maastricht
2. Department of Health Policy, London School of Economics and Political Science
3. Department of Health Policy, London School of Economics and Political Science
4. Care and Public Health Research Institute, Health Services Research, University of Maastricht
5. Care and Public Health Research Institute, Health Services Research, University of Maastricht

Reviewer	Husein Reka, Robin Van Kessel
Date	20 January 2024
**Document information:**	
Name	Extension of Procedures mentioned in Circular No. (23) of 2020 regarding Activity Based Funded Mandates Network Expansion
Author(s)	Department of Health Abu Dhabi
Year of publication	14 April 2023
Country/Emirate	Abu Dhabi
Link	https://www.doh.gov.ae/-/media/6F322BD75B7B471282B3BA77C0C62BC4.ashx
Aim/purpose	Extending activity based funded mandate for public and private hospitals in Abu Dhabi and procedures and prices
Record #	4
**Inclusion/Exclusion Criteria**	
Population	Private Health Insurance in Abu Dhabi
Concept	Transformation/Reforms of Abu Dhabi Private Health Insurance Scheme
Context	GCC Health System
**Review framework domains studied in literature on private health insurance transformation**	
Health System Framework (e.g. eligibility, sector size, user charges)	
Institutional environment (e.g. legal framework, regulation)	
Private Health Insurance policy framework (e.g. objective, population coverage, system description, types of coverage	
Market structure, conduct and performance (e.g. market concentration, barriers to entry, local vs. international, vertical integration, value-based health care)	

**Key findings related to scoping review question**	
Health System framework	
Eligibility	
Sector size	
User charges	
Institutional environment	
Legal framework	
Material regulation	
Prudential regulation	
Private Health Insurance policy framework	
Objective	
Population coverage	
System description	
Coverage type	
Market structure	
Concentration	
Barriers to entry	
Local vs international	
Vertical Integration	
Value Based Health Care	

Reviewer	Husein Reka, Robin Van Kessel
Date	20 January 2024
**Document information:**	
Name	Launching the Flexi Health Insurance Policy
Author(s)	Department of Health Abu Dhabi
Year of publication	16 February 2023
Country/Emirate	Abu Dhabi
Link	https://www.doh.gov.ae/-/media/46EFBDC68D0349B38E62A59C14658B35.ashx
Aim/purpose	Extending health insurance coverage to additional categories or resident and investors
Record #	5
**Inclusion/Exclusion Criteria**	
Population	Private Health Insurance in Abu Dhabii
Concept	Transformation/Reforms of Abu Dhabi Private Health Insurance Scheme
Context	GCC Health System
**Review framework domains studied in literature on private health insurance transformation**	
Health System Framework (e.g. eligibility, sector size, user charges)	Eligibility extension for additional categories in Abu Dhabi
Institutional environment (e.g. legal framework, regulation)	
Private Health Insurance policy framework (e.g. objective, population coverage, system description, types of coverage	
Market structure, conduct and performance (e.g. market concentration, barriers to entry, local vs. international, vertical integration, value-based health care)	
**Key findings related to scoping review question**	
Health System framework	
Eligibility	DOH resolution to amend certain provisions of the Executive Regulations of the Law No. (23) of 2005 regarding Health Insurance in the Emirate of Abu Dhabi to

	extend the eligibility for health insurance to additional categories:<br><br>• xpatriate residing in the Emirate who works in the private sector, provided that his/her monthly income exceeds 5,000 dirhams.<br>• • Investors and holders of free enterprises licenses, who desire to have this policy, and their families and employees.<br><br>• The family of the resident expatriate and his/her workers who are not covered by employer's health insurance policy (governmental or private).
Sector size	
User charges	
Institutional environment	
Legal framework	
Material regulation	
Prudential regulation	
Private Health Insurance policy framework	
Objective	
Population coverage	
System description	
Coverage type	
Market structure	
Concentration	
Barriers to entry	
Local vs international	
Vertical Integration	
Value Based Health Care	

Reviewer	Husein Reka, Robin Van Kessel
Date	20 January 2024
**Document information:**	
Name	Update on Laboratory & Radiology services prices Phase 1
Author(s)	Department of Health Abu Dhabi
Year of publication	28 Dec 2022
Country/Emirate	Abu Dhabi
Link	https://www.doh.gov.ae/-/media/8394AC1B99964DFDBB9CD19BD3412C38.ashx
Aim/purpose	Postponing of lab and radiology prices review
Record #	8
**Inclusion/Exclusion Criteria**	
Population	Private Health Insurance in Abu Dhabii
Concept	Transformation/Reforms of Abu Dhabi Private Health Insurance Scheme
Context	GCC Health System
**Review framework domains studied in literature on private health insurance transformation**	
Health System Framework (e.g. eligibility, sector size, user charges)	i
Institutional environment (e.g. legal framework, regulation)	
Private Health Insurance policy framework (e.g. objective, population coverage, system description, types of coverage	
Market structure, conduct and performance (e.g. market concentration, barriers to entry, local vs. international, vertical integration, value-based health care)	Mandatory tariffs for services
**Key findings related to scoping review question**	
Health System framework	
Eligibility	

Sector size	
User charges	
Institutional environment	
Legal framework	
Material regulation	
Prudential regulation	
Private Health Insurance policy framework	
Objective	
Population coverage	
System description	
Coverage type	
Market structure	
Concentration	
Barriers to entry	
Local vs international	
Vertical Integration	
Value Based Health Care	Regulator postponed an earlier issued mandatory price tariff for labs and radiology services

Reviewer	Husein Reka, Robin Van Kessel
Date	20 January 2024
**Document information:**	
Name	Updated Procedures regarding the Increase in Usage Rate
Author(s)	Department of Health Abu Dhabi
Year of publication	3 Nov 2022
Country/Emirate	Abu Dhabi
Link	https://www.doh.gov.ae/-/media/8394AC1B99964DFDBB9CD19BD3412C38.ashx
Aim/purpose	Utilization review for providers and fines and penalties
Record #	11
**Inclusion/Exclusion Criteria**	
Population	Private Health Insurance in Abu Dhabi
Concept	Transformation/Reforms of Abu Dhabi Private Health Insurance Scheme
Context	GCC Health System
**Review framework domains studied in literature on private health insurance transformation**	
Health System Framework (e.g. eligibility, sector size, user charges)	
Institutional environment (e.g. legal framework, regulation)	
Private Health Insurance policy framework (e.g. objective, population coverage, system description, types of coverage	
Market structure, conduct and performance (e.g. market concentration, barriers to entry, local vs. international, vertical integration, value-based health care)	Valuer based health care
**Key findings related to scoping review question**	
Health System framework	
Eligibility	
Sector size	
User charges	

Institutional environment	
Legal framework	
Material regulation	
Prudential regulation	
Private Health Insurance policy framework	
Objective	
Population coverage	
System description	
Coverage type	
Market structure	
Concentration	
Barriers to entry	
Local vs international	
Vertical Integration	
Value Based Health Care	Regulator informing all health insurance scheme participants on the rules and responsibilities in detecting and alerting on excessive utilization of services in the sector

Reviewer	Husein Reka, Robin Van Kessel
Date	20 January 2024
**Document information:**	
Name	Update on Laboratory & Radiology services prices Phase 1
Author(s)	Department of Health Abu Dhabi
Year of publication	7 Oct 2022
Country/Emirate	Abu Dhabi
Link	https://www.doh.gov.ae/-/media/3301EEBFBC1F457A89E86E88E8D6F6B7.ashx
Aim/purpose	Tariff mandatory price for lab and radiology services
Record #	14
**Inclusion/Exclusion Criteria**	
Population	Private Health Insurance in Abu Dhabii
Concept	Transformation/Reforms of Abu Dhabi Private Health Insurance Scheme
Context	GCC Health System
**Review framework domains studied in literature on private health insurance transformation**	
Health System Framework (e.g. eligibility, sector size, user charges)	
Institutional environment (e.g. legal framework, regulation)	
Private Health Insurance policy framework (e.g. objective, population coverage, system description, types of coverage	
Market structure, conduct and performance (e.g. market concentration, barriers to entry, local vs. international, vertical integration, value-based health care)	Regulator mandating tariffs for lab and radiology services
**Key findings related to scoping review question**	
Health System framework	
Eligibility	
Sector size	
User charges	

Institutional environment	
Legal framework	
Material regulation	
Prudential regulation	
Private Health Insurance policy framework	
Objective	
Population coverage	
System description	
Coverage type	
Market structure	
Concentration	
Barriers to entry	
Local vs international	
Vertical Integration	
Value Based Health Care	As part of efforts to tackle overutilization and effective provision of care, DOH issued mandatory tariff price for 29 lab services and 17 radiology services

Reviewer	Husein Reka, Robin Van Kessel
Date	20 January 2024
**Document information:**	
Name	Mandatory to document the time of any procedure/surgery in the patient's medical record reported using a Time-Based Procedure Code (CPT/Dental).
Author(s)	Department of Health Abu Dhabi
Year of publication	29 Sep 2022
Country/Emirate	Abu Dhabi
Link	https://www.doh.gov.ae/-/media/3301EEBFBC1F457A89E86E88E8D6F6B7.ashx
Aim/purpose	Mandatory recording of procedure time for certain services
Record #	15
**Inclusion/Exclusion Criteria**	
Population	Private Health Insurance in Abu Dhabii
Concept	Transformation/Reforms of Abu Dhabi Private Health Insurance Scheme
Context	GCC Health System
**Review framework domains studied in literature on private health insurance transformation**	
Health System Framework (e.g. eligibility, sector size, user charges)	
Institutional environment (e.g. legal framework, regulation)	
Private Health Insurance policy framework (e.g. objective, population coverage, system description, types of coverage	
Market structure, conduct and performance (e.g. market concentration, barriers to entry, local vs. international, vertical integration, value-based health care)	Regulator mandating measurement of time required for certain services
**Key findings related to scoping review question**	
Health System framework	

Eligibility	
Sector size	
User charges	
Institutional environment	
Legal framework	
Material regulation	
Prudential regulation	
Private Health Insurance policy framework	
Objective	
Population coverage	
System description	
Coverage type	
Market structure	
Concentration	
Barriers to entry	
Local vs international	
Vertical Integration	
Value Based Health Care	DOH made it mandatory to record the “start time” and “end time” of any procedure/surgery in the patient’s medical record reported using a Time-Based Procedure Code (CPT/Dental).

Reviewer	Husein Reka, Robin Van Kessel
Date	20 January 2024
**Document information:**	
Name	Cancellation of Circular No. 56/2020 regarding Decoupling between Enrollment (new/ renew) in the Health Insurance Scheme and Payment of Fines Resulting from Delay in the Enrollment in the Health Insurance Scheme
Author(s)	Department of Health Abu Dhabi
Year of publication	17 Aug 2022
Country/Emirate	Abu Dhabi
Link	https://www.doh.gov.ae/-/media/35F46A7E54754039BE320A0E374CF010.ashx
Aim/purpose	Regulator cancelling previous decision on decoupling renewal and fine payment
Record #	18
**Inclusion/Exclusion Criteria**	
Population	Private Health Insurance in Abu Dhabii
Concept	Transformation/Reforms of Abu Dhabi Private Health Insurance Scheme
Context	GCC Health System
**Review framework domains studied in literature on private health insurance transformation**	
Health System Framework (e.g. eligibility, sector size, user charges)	Enforcement and penalties for mandatory health insurance
Institutional environment (e.g. legal framework, regulation)	
Private Health Insurance policy framework (e.g. objective, population coverage, system description, types of coverage	
Market structure, conduct and performance (e.g. market concentration, barriers to entry, local vs. international, vertical integration, value-based health care)	
**Key findings related to scoping review question**	
Health System framework	

Eligibility	DOH clarified to the market that the decoupling between enrolment and subscription in the health insurance scheme and fines/penalties decoupling has been removed and violators are required to reconcile with DOH
Sector size	
User charges	
Institutional environment	
Legal framework	
Material regulation	
Prudential regulation	
Private Health Insurance policy framework	
Objective	
Population coverage	
System description	
Coverage type	
Market structure	
Concentration	
Barriers to entry	
Local vs international	
Vertical Integration	
Value Based Health Care	

Reviewer	Husein Reka, Robin Van Kessel
Date	20 January 2024
**Document information:**	
Name	Mandatory Update of Personal Records of All Insured Members Electronically on Shafafiya System
Author(s)	Department of Health Abu Dhabi
Year of publication	26 Jul 2022
Country/Emirate	Abu Dhabi
Link	https://www.doh.gov.ae/-/media/10577A3CBB7243A89686BE091E8F201B.ashx
Aim/purpose	Mandatory inclusion of personal ID for health insurance claims system (Shafafiya)
Record #	21
**Inclusion/Exclusion Criteria**	
Population	Private Health Insurance in Abu Dhabii
Concept	Transformation/Reforms of Abu Dhabi Private Health Insurance Scheme
Context	GCC Health System
**Review framework domains studied in literature on private health insurance transformation**	
Health System Framework (e.g. eligibility, sector size, user charges)	Mandatory inclusion of ID in claims management system
Institutional environment (e.g. legal framework, regulation)	
Private Health Insurance policy framework (e.g. objective, population coverage, system description, types of coverage	
Market structure, conduct and performance (e.g. market concentration, barriers to entry, local vs. international, vertical integration, value-based health care)	
**Key findings related to scoping review question**	
Health System framework	

Eligibility	DOH made it mandatory for all claims and registration of individuals in the Shafafiya system to include personal ID
Sector size	
User charges	
Institutional environment	
Legal framework	
Material regulation	
Prudential regulation	
Private Health Insurance policy framework	
Objective	
Population coverage	
System description	
Coverage type	
Market structure	
Concentration	
Barriers to entry	
Local vs international	
Vertical Integration	
Value Based Health Care	

Reviewer	Husein Reka, Robin Van Kessel
Date	20 January 2024
**Document information:**	
Name	Procedures to be followed to address cases of suspected fraud and/or abuse of health services by impersonating third parties for the purpose of illegally benefiting from medical treatment services
Author(s)	Department of Health Abu Dhabi
Year of publication	30 Jun 2022
Country/Emirate	Abu Dhabi
Link	https://www.doh.gov.ae/-/media/10577A3CBB7243A89686BE091E8F201B.ashx
Aim/purpose	Mandatory inclusion of personal ID for health insurance claims system (Shafafiya)
Record #	22
**Inclusion/Exclusion Criteria**	
Population	Private Health Insurance in Abu Dhabii
Concept	Transformation/Reforms of Abu Dhabi Private Health Insurance Scheme
Context	GCC Health System
**Review framework domains studied in literature on private health insurance transformation**	
Health System Framework (e.g. eligibility, sector size, user charges)	Tackling identity fraud abuse in heath insurance
Institutional environment (e.g. legal framework, regulation)	
Private Health Insurance policy framework (e.g. objective, population coverage, system description, types of coverage	
Market structure, conduct and performance (e.g. market concentration, barriers to entry, local vs. international, vertical integration, value-based health care)	
**Key findings related to scoping review question**	
Health System framework	

Eligibility	DOH introduces procedures for health insurance scheme participants on how to tackle fraud and abuse due to impersonation
Sector size	
User charges	
Institutional environment	
Legal framework	
Material regulation	
Prudential regulation	
Private Health Insurance policy framework	
Objective	
Population coverage	
System description	
Coverage type	
Market structure	
Concentration	
Barriers to entry	
Local vs international	
Vertical Integration	
Value Based Health Care	

Reviewer	Husein Reka, Robin Van Kessel
Date	20 January 2024
**Document information:**	
Name	Update to Circular No. 36/2021 on the Timeline for Implementation of Healthcare Reimbursement Reform in the Emirate of Abu Dhabi
Author(s)	Department of Health Abu Dhabi
Year of publication	3 Jun 2022
Country/Emirate	Abu Dhabi
Link	https://www.doh.gov.ae/-/media/1AB9A67B079744159379A6FCD47F6532.ashx
Aim/purpose	Updates to market on reimbursement reforms timelines
Record #	25
**Inclusion/Exclusion Criteria**	
Population	Private Health Insurance in Abu Dhabi
Concept	Transformation/Reforms of Abu Dhabi Private Health Insurance Scheme
Context	GCC Health System
**Review framework domains studied in literature on private health insurance transformation**	
Health System Framework (e.g. eligibility, sector size, user charges)	
Institutional environment (e.g. legal framework, regulation)	
Private Health Insurance policy framework (e.g. objective, population coverage, system description, types of coverage	
Market structure, conduct and performance (e.g. market concentration, barriers to entry, local vs. international, vertical integration, value-based health care)	
**Key findings related to scoping review question**	
Health System framework	
Eligibility	
Sector size	

User charges	
Institutional environment	
Legal framework	
Material regulation	
Prudential regulation	
Private Health Insurance policy framework	
Objective	
Population coverage	
System description	
Coverage type	
Market structure	
Concentration	
Barriers to entry	
Local vs international	
Vertical Integration	
Value Based Health Care	DOH announces the pilot phase to implement the inpatient acute healthcare reimbursement reform changes, through conducting a shadow billing exercise aiming to drive for the market preparedness to accommodate and take the necessary technical steps in participants' billing systems to be in line with the new reimbursement system changes

Reviewer	Husein Reka, Robin Van Kessel
Date	20 January 2024
**Document information:**	
Name	Cases where no violation or fine as a result of Non-participation or Non-renewal of the Health Insurance by the sponsor
Author(s)	Department of Health Abu Dhabi
Year of publication	2 Jun 2022
Country/Emirate	Abu Dhabi
Link	https://www.doh.gov.ae/-/media/FFD9D244FAC24949ACAB37FD84DB87F0.ashx
Aim/purpose	Exemption on violations due to non-renewal
Record #	26
**Inclusion/Exclusion Criteria**	
Population	Private Health Insurance in Abu Dhabii
Concept	Transformation/Reforms of Abu Dhabi Private Health Insurance Scheme
Context	GCC Health System
**Review framework domains studied in literature on private health insurance transformation**	
Health System Framework (e.g. eligibility, sector size, user charges)	Compliance and enforcement clarifications
Institutional environment (e.g. legal framework, regulation)	
Private Health Insurance policy framework (e.g. objective, population coverage, system description, types of coverage	
Market structure, conduct and performance (e.g. market concentration, barriers to entry, local vs. international, vertical integration, value-based health care)	
**Key findings related to scoping review question**	
Health System framework	

Eligibility	DOH clarifies cases and situations under which no violation is considered for non compliance with health insurance mandate
Sector size	
User charges	
Institutional environment	
Legal framework	
Material regulation	
Prudential regulation	
Private Health Insurance policy framework	
Objective	
Population coverage	
System description	
Coverage type	
Market structure	
Concentration	
Barriers to entry	
Local vs international	
Vertical Integration	
Value Based Health Care	

Reviewer	Husein Reka, Robin Van Kessel
Date	20 January 2024
**Document information:**	
Name	Extension of Procedures mentioned in Circular No. (23) of 2020 regarding Activity Based Funded Mandates Network Expansion.
Author(s)	Department of Health Abu Dhabi
Year of publication	19 Apr 2022
Country/Emirate	Abu Dhabi
Link	https://www.doh.gov.ae/-/media/BFA6A5539B974771A7ADDC4ABB27BBBA.ashx
Aim/purpose	Extension of deadlines for Activity Based Funded mandates
Record #	28
**Inclusion/Exclusion Criteria**	
Population	Private Health Insurance in Abu Dhabi
Concept	Transformation/Reforms of Abu Dhabi Private Health Insurance Scheme
Context	GCC Health System
**Review framework domains studied in literature on private health insurance transformation**	
Health System Framework (e.g. eligibility, sector size, user charges)	
Institutional environment (e.g. legal framework, regulation)	
Private Health Insurance policy framework (e.g. objective, population coverage, system description, types of coverage	
Market structure, conduct and performance (e.g. market concentration, barriers to entry, local vs. international, vertical integration, value-based health care)	Activity Based Funding mandate
**Key findings related to scoping review question**	
Health System framework	
Eligibility	

Sector size	
User charges	
Institutional environment	
Legal framework	
Material regulation	
Prudential regulation	
Private Health Insurance policy framework	
Objective	
Population coverage	
System description	
Coverage type	
Market structure	
Concentration	
Barriers to entry	
Local vs international	
Vertical Integration	
Value Based Health Care	DOH extends deadlines of a previous circular NO. 23 of 01 10 2022 on Activity Based Funding mandates

Reviewer	Husein Reka, Robin Van Kessel
Date	20 January 2024
**Document information:**	
Name	Regulating Health Insurance for Golden Visas Permits
Author(s)	Department of Health Abu Dhabi
Year of publication	19 Apr 2022
Country/Emirate	Abu Dhabi
Link	https://www.doh.gov.ae/-/media/20C92256FD494E5FAF7236F0E839BCC9.ashx
Aim/purpose	Extension of eligibility for new category of visa holders
Record #	30
**Inclusion/Exclusion Criteria**	
Population	Private Health Insurance in Abu Dhabii
Concept	Transformation/Reforms of Abu Dhabi Private Health Insurance Scheme
Context	GCC Health System
**Review framework domains studied in literature on private health insurance transformation**	
Health System Framework (e.g. eligibility, sector size, user charges)	Extension of eligibility for new categories
Institutional environment (e.g. legal framework, regulation)	
Private Health Insurance policy framework (e.g. objective, population coverage, system description, types of coverage	
Market structure, conduct and performance (e.g. market concentration, barriers to entry, local vs. international, vertical integration, value-based health care)	
**Key findings related to scoping review question**	
Health System framework	
Eligibility	DOH issued regulations on regulating health insurance for individual seeking golden visa and heir dependants
Sector size	
User charges	

Institutional environment	
Legal framework	
Material regulation	
Prudential regulation	
Private Health Insurance policy framework	
Objective	
Population coverage	
System description	
Coverage type	
Market structure	
Concentration	
Barriers to entry	
Local vs international	
Vertical Integration	
Value Based Health Care	

Reviewer	Husein Reka, Robin Van Kessel
Date	20 January 2024
**Document information:**	
Name	Revision & Update of the Standard Provider Contract (SPC)
Author(s)	Department of Health Abu Dhabi
Year of publication	18 Jan 2022
Country/Emirate	Abu Dhabi
Link	https://www.doh.gov.ae/-/media/4491C88CFBCB4A75A6E777CC1ECCA3E3.ashx
Aim/purpose	Updates on Standard Provider Contracts
Record #	36
**Inclusion/Exclusion Criteria**	
Population	Private Health Insurance in Abu Dhabii
Concept	Transformation/Reforms of Abu Dhabi Private Health Insurance Scheme
Context	GCC Health System
**Review framework domains studied in literature on private health insurance transformation**	
Health System Framework (e.g. eligibility, sector size, user charges)	
Institutional environment (e.g. legal framework, regulation)	
Private Health Insurance policy framework (e.g. objective, population coverage, system description, types of coverage	
Market structure, conduct and performance (e.g. market concentration, barriers to entry, local vs. international, vertical integration, value-based health care)	
**Key findings related to scoping review question**	
Health System framework	
Eligibility	
Sector size	
User charges	

Institutional environment	
Legal framework	
Material regulation	
Prudential regulation	
Private Health Insurance policy framework	
Objective	
Population coverage	
System description	
Coverage type	
Market structure	
Concentration	
Barriers to entry	
Local vs international	
Vertical Integration	
Value Based Health Care	DOH updates SPC with explicit turnaround times for claims submission and adjudication

Reviewer	Husein Reka, Robin Van Kessel
Date	20 January 2024
**Document information:**	
Name	Extension for the procedures mentioned in Circular No. (23) of 2020 regarding Activity Based Funded Mandates Network Expansion
Author(s)	Department of Health Abu Dhabi
Year of publication	12 Jan 2022
Country/Emirate	Abu Dhabi
Link	https://www.doh.gov.ae/-/media/EB36D7383AC94C1090555AC4F2C4C407.ashx
Aim/purpose	Extension of deadlines for Activity Based Funded mandates
Record #	37
**Inclusion/Exclusion Criteria**	
Population	Private Health Insurance in Abu Dhabi
Concept	Transformation/Reforms of Abu Dhabi Private Health Insurance Scheme
Context	GCC Health System
**Review framework domains studied in literature on private health insurance transformation**	
Health System Framework (e.g. eligibility, sector size, user charges)	
Institutional environment (e.g. legal framework, regulation)	
Private Health Insurance policy framework (e.g. objective, population coverage, system description, types of coverage	
Market structure, conduct and performance (e.g. market concentration, barriers to entry, local vs. international, vertical integration, value-based health care)	Activity Based Funding mandate
**Key findings related to scoping review question**	
Health System framework	
Eligibility	

Sector size	
User charges	
Institutional environment	
Legal framework	
Material regulation	
Prudential regulation	
Private Health Insurance policy framework	
Objective	
Population coverage	
System description	
Coverage type	
Market structure	
Concentration	
Barriers to entry	
Local vs international	
Vertical Integration	
Value Based Health Care	DOH extend the procedures mentioned in circular No. (23) until 31/03/2022

Reviewer	Husein Reka, Robin Van Kessel
Date	20 January 2024
**Document information:**	
Name	Extension for the procedures mentioned in Circular No. (23) of 2020 regarding Activity Based Funded Mandates Network Expansion
Author(s)	Department of Health Abu Dhabi
Year of publication	26 Dec 2021
Country/Emirate	Abu Dhabi
Link	https://www.doh.gov.ae/-/media/35988D87E0CF42D1BACA9AE09F322798.ashx
Aim/purpose	ABF mandate extension
Record #	41
**Inclusion/Exclusion Criteria**	
Population	Private Health Insurance in Abu Dhabi
Concept	Transformation/Reforms of Abu Dhabi Private Health Insurance Scheme
Context	GCC Health System
**Review framework domains studied in literature on private health insurance transformation**	
Health System Framework (e.g. eligibility, sector size, user charges)	
Institutional environment (e.g. legal framework, regulation)	
Private Health Insurance policy framework (e.g. objective, population coverage, system description, types of coverage	
Market structure, conduct and performance (e.g. market concentration, barriers to entry, local vs. international, vertical integration, value-based health care)	Medication refill rule for PBMs
**Key findings related to scoping review question**	
Health System framework	
Eligibility	
Sector size	

User charges	
Institutional environment	
Legal framework	
Material regulation	
Prudential regulation	
Private Health Insurance policy framework	
Objective	
Population coverage	
System description	
Coverage type	
Market structure	
Concentration	
Barriers to entry	
Local vs international	
Vertical Integration	
Value Based Health Care	DOH extends deadline for medication refill rule for PBM systems

Reviewer	Husein Reka, Robin Van Kessel
Date	20 January 2024
**Document information:**	
Name	Extension of the period of reduction and exemption from fines pertaining to Basic Policy and other Policies for Individually-sponsored Individuals
Author(s)	Department of Health Abu Dhabi
Year of publication	21 Nov 2021
Country/Emirate	Abu Dhabi
Link	https://www.doh.gov.ae/-/media/66E83933855D448A9DDA21961DE37122.ashx
Aim/purpose	Extension of compliance period
Record #	42
**Inclusion/Exclusion Criteria**	
Population	Private Health Insurance in Abu Dhabi
Concept	Transformation/Reforms of Abu Dhabi Private Health Insurance Scheme
Context	GCC Health System
**Review framework domains studied in literature on private health insurance transformation**	
Health System Framework (e.g. eligibility, sector size, user charges)	Compliance penalties
Institutional environment (e.g. legal framework, regulation)	
Private Health Insurance policy framework (e.g. objective, population coverage, system description, types of coverage	
Market structure, conduct and performance (e.g. market concentration, barriers to entry, local vs. international, vertical integration, value-based health care)	
**Key findings related to scoping review question**	
Health System framework	
Eligibility	
Sector size	

User charges	
Institutional environment	
Legal framework	
Material regulation	
Prudential regulation	
Private Health Insurance policy framework	
Objective	
Population coverage	
System description	
Coverage type	
Market structure	
Concentration	
Barriers to entry	
Local vs international	
Vertical Integration	
Value Based Health Care	DOH clarification on extension and reduction of fines for basic health insurance to include self-employed as well

Reviewer	Husein Reka, Robin Van Kessel
Date	20 January 2024
**Document information:**	
Name	Multiplication of Basic Policy Period (Individual)
Author(s)	Department of Health Abu Dhabi
Year of publication	15 Nov 2021
Country/Emirate	Abu Dhabi
Link	https://www.doh.gov.ae/-/media/15331EA3F6F2448283EC6CF0642C5F55.ashx
Aim/purpose	Policy validity
Record #	43
**Inclusion/Exclusion Criteria**	
Population	Private Health Insurance in Abu Dhabi
Concept	Transformation/Reforms of Abu Dhabi Private Health Insurance Scheme
Context	GCC Health System
**Review framework domains studied in literature on private health insurance transformation**	
Health System Framework (e.g. eligibility, sector size, user charges)	Policy validity more than one year on voluntary basis
Institutional environment (e.g. legal framework, regulation)	
Private Health Insurance policy framework (e.g. objective, population coverage, system description, types of coverage	
Market structure, conduct and performance (e.g. market concentration, barriers to entry, local vs. international, vertical integration, value-based health care)	
**Key findings related to scoping review question**	
Health System framework	
Eligibility	
Sector size	
User charges	
Institutional environment	

Legal framework	
Material regulation	
Prudential regulation	
Private Health Insurance policy framework	
Objective	
Population coverage	
System description	
Coverage type	
Market structure	
Concentration	
Barriers to entry	
Local vs international	
Vertical Integration	
Value Based Health Care	DOH explains that individual basic policy can be issued for more than a calendar year, and that the policy duration may be multiplied to two or three years in maximum within regulations and on voluntary basis

Reviewer	Husein Reka, Robin Van Kessel
Date	20 January 2024
**Document information:**	
Name	Extension of Period of Reduction and Exemption from Fines pertaining to Basic Policy and other Policies for Individually-sponsored Individuals
Author(s)	Department of Health Abu Dhabi
Year of publication	9 Nov 2021
Country/Emirate	Abu Dhabi
Link	https://www.doh.gov.ae/-/media/FAAA15587E74472BAEAC17A8445FAE5B.ashx
Aim/purpose	Extension of period for compliance
Record #	44
**Inclusion/Exclusion Criteria**	
Population	Private Health Insurance in Abu Dhabi
Concept	Transformation/Reforms of Abu Dhabi Private Health Insurance Scheme
Context	GCC Health System
**Review framework domains studied in literature on private health insurance transformation**	
Health System Framework (e.g. eligibility, sector size, user charges)	Extension of period for reduction and exemption from fines
Institutional environment (e.g. legal framework, regulation)	
Private Health Insurance policy framework (e.g. objective, population coverage, system description, types of coverage	
Market structure, conduct and performance (e.g. market concentration, barriers to entry, local vs. international, vertical integration, value-based health care)	
**Key findings related to scoping review question**	
Health System framework	
Eligibility	
Sector size	

User charges	
Institutional environment	
Legal framework	
Material regulation	
Prudential regulation	
Private Health Insurance policy framework	
Objective	
Population coverage	
System description	
Coverage type	
Market structure	
Concentration	
Barriers to entry	
Local vs international	
Vertical Integration	
Value Based Health Care	DOH extends the period for fines reduction and exemption for non-compliance

Reviewer	Husein Reka, Robin Van Kessel
Date	20 January 2024
**Document information:**	
Name	Update to Circular No. 36 / 2021 on the Timeline for Implementation of Healthcare Reimbursement Reform in the Emirate of Abu Dhabi
Author(s)	Department of Health Abu Dhabi
Year of publication	8 Nov 2021
Country/Emirate	Abu Dhabi
Link	https://www.doh.gov.ae/-/media/BA423DE26C0C42289428B9F9C57D3E3E.ashx
Aim/purpose	Update to market on base rate and new adjuster framework
Record #	45
**Inclusion/Exclusion Criteria**	
Population	Private Health Insurance in Abu Dhabi
Concept	Transformation/Reforms of Abu Dhabi Private Health Insurance Scheme
Context	GCC Health System
**Review framework domains studied in literature on private health insurance transformation**	
Health System Framework (e.g. eligibility, sector size, user charges)	
Institutional environment (e.g. legal framework, regulation)	
Private Health Insurance policy framework (e.g. objective, population coverage, system description, types of coverage	
Market structure, conduct and performance (e.g. market concentration, barriers to entry, local vs. international, vertical integration, value-based health care)	
**Key findings related to scoping review question**	
Health System framework	
Eligibility	

Sector size	
User charges	
Institutional environment	
Legal framework	
Material regulation	
Prudential regulation	
Private Health Insurance policy framework	
Objective	
Population coverage	
System description	
Coverage type	
Market structure	
Concentration	
Barriers to entry	
Local vs international	
Vertical Integration	
Value Based Health Care	DOH issue market consultation and timelines on inpatient Base Rate for use with IR DRG v3.3, updated Mandatory Tariff

Reviewer	Husein Reka
Date	20 January 2024
**Document information:**	
Name	Pharmacy Benefits Management (PBM) Update
Author(s)	Department of Health Abu Dhabi
Year of publication	16 Sep 2021
Country/Emirate	Abu Dhabi
Link	https://www.doh.gov.ae/-/media/6A982B3D39C94533B5B6A1DA4B9F3D98.ashx
Aim/purpose	Update on refill rule
Record #	49
**Inclusion/Exclusion Criteria**	
Population	Private Health Insurance in Abu Dhabi
Concept	Transformation/Reforms of Abu Dhabi Private Health Insurance Scheme
Context	GCC Health System
**Review framework domains studied in literature on private health insurance transformation**	
Health System Framework (e.g. eligibility, sector size, user charges)	
Institutional environment (e.g. legal framework, regulation)	
Private Health Insurance policy framework (e.g. objective, population coverage, system description, types of coverage	
Market structure, conduct and performance (e.g. market concentration, barriers to entry, local vs. international, vertical integration, value-based health care)	
**Key findings related to scoping review question**	
Health System framework	
Eligibility	
Sector size	
User charges	
Institutional environment	

Legal framework	
Material regulation	
Prudential regulation	
Private Health Insurance policy framework	
Objective	
Population coverage	
System description	
Coverage type	
Market structure	
Concentration	
Barriers to entry	
Local vs international	
Vertical Integration	
Value Based Health Care	DOH issued market update on refill rule for PBM in Shafafiya system

Reviewer	Husein Reka
Date	20 January 2024
**Document information:**	
Name	Update to Circular No. 36 / 2021 on the Timeline for Implementation of Healthcare Reimbursement Reform in the Emirate of Abu Dhabi
Author(s)	Department of Health Abu Dhabi
Year of publication	1 Sep 2021
Country/Emirate	Abu Dhabi
Link	https://www.doh.gov.ae/-/media/D40B99F04BE04B7FB1A01A4CC97B0D16.ashx
Aim/purpose	Market consultation on base rate and adjusters
Record #	51
**Inclusion/Exclusion Criteria**	
Population	Private Health Insurance in Abu Dhabi
Concept	Transformation/Reforms of Abu Dhabi Private Health Insurance Scheme
Context	GCC Health System
**Review framework domains studied in literature on private health insurance transformation**	
Health System Framework (e.g. eligibility, sector size, user charges)	
Institutional environment (e.g. legal framework, regulation)	
Private Health Insurance policy framework (e.g. objective, population coverage, system description, types of coverage	
Market structure, conduct and performance (e.g. market concentration, barriers to entry, local vs. international, vertical integration, value-based health care)	
**Key findings related to scoping review question**	
Health System framework	
Eligibility	
Sector size	

User charges	
Institutional environment	
Legal framework	
Material regulation	
Prudential regulation	
Private Health Insurance policy framework	
Objective	
Population coverage	
System description	
Coverage type	
Market structure	
Concentration	
Barriers to entry	
Local vs international	
Vertical Integration	
Value Based Health Care	DOH issued market update on market consultations for Inpatient Base Rates and Adjustor Framework that will be implemented on 1 January 2022

Reviewer	Husein Reka
Date	20 January 2024
**Document information:**	
Name	Submitting Remittance Advice Details for Claims
Author(s)	Department of Health Abu Dhabi
Year of publication	29 Aug 2021
Country/Emirate	Abu Dhabi
Link	https://www.doh.gov.ae/-/media/1477DBFAFEA84F8391003D80D46AC095.ashx
Aim/purpose	Financial reporting requirement for health insurance companies
Record #	53
**Inclusion/Exclusion Criteria**	
Population	Private Health Insurance in Abu Dhabi
Concept	Transformation/Reforms of Abu Dhabi Private Health Insurance Scheme
Context	GCC Health System
**Review framework domains studied in literature on private health insurance transformation**	
Health System Framework (e.g. eligibility, sector size, user charges)	
Institutional environment (e.g. legal framework, regulation)	Regulatory requirement for financial reporting
Private Health Insurance policy framework (e.g. objective, population coverage, system description, types of coverage	
Market structure, conduct and performance (e.g. market concentration, barriers to entry, local vs. international, vertical integration, value-based health care)	
**Key findings related to scoping review question**	
Health System framework	
Eligibility	
Sector size	
User charges	

Institutional environment	
Legal framework	
Material regulation	
Prudential regulation	Requirement by regulator that all health insurance companies, licensed to operate in the field of health insurance in the Emirate of Abu Dhabi, are mandated to submit the remittance advice details for claims, along with the quarterly financial statements
Private Health Insurance policy framework	
Objective	
Population coverage	
System description	
Coverage type	
Market structure	
Concentration	
Barriers to entry	
Local vs international	
Vertical Integration	
Value Based Health Care	

Reviewer	Husein Reka
Date	20 January 2024
**Document information:**	
Name	Cancellation of Reduction/Exemption from Health Insurance Fines pertaining to Basic Policy and other Policies for Individuallysponsored Individuals
Author(s)	Department of Health Abu Dhabi
Year of publication	29 Aug 2021
Country/Emirate	Abu Dhabi
Link	https://www.doh.gov.ae/-/media/760219A2B9C4475A95E0ED250F584BAF.ashx
Aim/purpose	Cancellation of previous circular on fines
Record #	54
**Inclusion/Exclusion Criteria**	
Population	Private Health Insurance in Abu Dhabi
Concept	Transformation/Reforms of Abu Dhabi Private Health Insurance Scheme
Context	GCC Health System
**Review framework domains studied in literature on private health insurance transformation**	
Health System Framework (e.g. eligibility, sector size, user charges)	Fines for non-compliance
Institutional environment (e.g. legal framework, regulation)	
Private Health Insurance policy framework (e.g. objective, population coverage, system description, types of coverage	
Market structure, conduct and performance (e.g. market concentration, barriers to entry, local vs. international, vertical integration, value-based health care)	
**Key findings related to scoping review question**	
Health System framework	
Eligibility	DOH cancels the reduction and exemption from health insurance fines for the basic policy and other policies

	belonging to individuals, who are individually sponsored with fines for failure to subscribe at the rate of 300 dirhams per month for each individual
Sector size	
User charges	
Institutional environment	
Legal framework	
Material regulation	
Prudential regulation	
Private Health Insurance policy framework	
Objective	
Population coverage	
System description	
Coverage type	
Market structure	
Concentration	
Barriers to entry	
Local vs international	
Vertical Integration	
Value Based Health Care	

Reviewer	Husein Reka
Date	20 January 2024
**Document information:**	
Name	Addeddum to Circular 36/2021 on the Timeline for Implementation of Healthcare Reimbursement Reform in the Emirate of Abu Dhabi
Author(s)	Department of Health Abu Dhabi
Year of publication	29 Jul 2021
Country/Emirate	Abu Dhabi
Link	https://www.doh.gov.ae/-/media/E2FBD48BF3874BF4BFCFBF85ECE83E82.ashx
Aim/purpose	Market consultation on IR-DRG grouper and base rate
Record #	59
**Inclusion/Exclusion Criteria**	
Population	Private Health Insurance in Abu Dhabi
Concept	Transformation/Reforms of Abu Dhabi Private Health Insurance Scheme
Context	GCC Health System
**Review framework domains studied in literature on private health insurance transformation**	
Health System Framework (e.g. eligibility, sector size, user charges)	
Institutional environment (e.g. legal framework, regulation)	
Private Health Insurance policy framework (e.g. objective, population coverage, system description, types of coverage	
Market structure, conduct and performance (e.g. market concentration, barriers to entry, local vs. international, vertical integration, value-based health care)	Implementation of healthcare reimbursement reform
**Key findings related to scoping review question**	
Health System framework	

Eligibility	DOH provides implementation timelines addendum on grouper product consultations for 3M product and base rates
Sector size	
User charges	
Institutional environment	
Legal framework	
Material regulation	
Prudential regulation	
Private Health Insurance policy framework	
Objective	
Population coverage	
System description	
Coverage type	
Market structure	
Concentration	
Barriers to entry	
Local vs international	
Vertical Integration	
Value Based Health Care	

Reviewer	Husein Reka
Date	20 January 2024
**Document information:**	
Name	Mandatory to notify the employer/ sponsor of renewal of health insurance subscription
Author(s)	Department of Health Abu Dhabi
Year of publication	22 Jun 2021
Country/Emirate	Abu Dhabi
Link	https://www.doh.gov.ae/-/media/292CA045ACDD443190271B5A51B46644.ashx
Aim/purpose	Enforcement of renewal notification for payers
Record #	62
**Inclusion/Exclusion Criteria**	
Population	Private Health Insurance in Abu Dhabi
Concept	Transformation/Reforms of Abu Dhabi Private Health Insurance Scheme
Context	GCC Health System
**Review framework domains studied in literature on private health insurance transformation**	
Health System Framework (e.g. eligibility, sector size, user charges)	
Institutional environment (e.g. legal framework, regulation)	Requirement for notification to employer for renewal
Private Health Insurance policy framework (e.g. objective, population coverage, system description, types of coverage	
Market structure, conduct and performance (e.g. market concentration, barriers to entry, local vs. international, vertical integration, value-based health care)	
**Key findings related to scoping review question**	
Health System framework	
Eligibility	
Sector size	
User charges	

Institutional environment	
Legal framework	
Material regulation	DOH requires health insurance companies to notify by SMS to employer / sponsor in Arabic and English at least 30 days prior to the expiry of the health insurance, and followed by reminder within 14 days
Prudential regulation	
Private Health Insurance policy framework	
Objective	
Population coverage	
System description	
Coverage type	
Market structure	
Concentration	
Barriers to entry	
Local vs international	
Vertical Integration	
Value Based Health Care	

Reviewer	Husein Reka
Date	20 January 2024
**Document information:**	
Name	Health Insurance Non-Covered Drug List (NC) in the Emirate of Abu Dhabi
Author(s)	Department of Health Abu Dhabi
Year of publication	1 Jun 2021
Country/Emirate	Abu Dhabi
Link	https://www.doh.gov.ae/-/media/54E06A3322B14016B4D00475A4FF9E14.ashx
Aim/purpose	Drugs negative list introduction
Record #	66
**Inclusion/Exclusion Criteria**	
Population	Private Health Insurance in Abu Dhabi
Concept	Transformation/Reforms of Abu Dhabi Private Health Insurance Scheme
Context	GCC Health System
**Review framework domains studied in literature on private health insurance transformation**	
Health System Framework (e.g. eligibility, sector size, user charges)	
Institutional environment (e.g. legal framework, regulation)	
Private Health Insurance policy framework (e.g. objective, population coverage, system description, types of coverage	
Market structure, conduct and performance (e.g. market concentration, barriers to entry, local vs. international, vertical integration, value-based health care)	DOH introduces negative drug list
**Key findings related to scoping review question**	
Health System framework	
Eligibility	
Sector size	
User charges	

Institutional environment	
Legal framework	
Material regulation	
Prudential regulation	
Private Health Insurance policy framework	
Objective	
Population coverage	
System description	
Coverage type	
Market structure	
Concentration	
Barriers to entry	
Local vs international	
Vertical Integration	
Value Based Health Care	DOH introduces negative drug list is comprised of products deemed to be of low clinical effectiveness (lack of robust evidence to support their use), or where there exists an alternative conventional product or medicine with superior demonstrated clinical efficacy

Reviewer	Husein Reka
Date	20 January 2024
**Document information:**	
Name	Mandatory Update of Personal Records of all Insured Members Electronically within 24 hours on Shafafiya
Author(s)	Department of Health Abu Dhabi
Year of publication	5 Apr 2021
Country/Emirate	Abu Dhabi
Link	https://www.doh.gov.ae/-/media/53DCD2116EED4C60A8F1E2AFA361A862.ashx
Aim/purpose	Personal records update in Shafafiya
Record #	70
**Inclusion/Exclusion Criteria**	
Population	Private Health Insurance in Abu Dhabi
Concept	Transformation/Reforms of Abu Dhabi Private Health Insurance Scheme
Context	GCC Health System
**Review framework domains studied in literature on private health insurance transformation**	
Health System Framework (e.g. eligibility, sector size, user charges)	Requirement to add personal information
Institutional environment (e.g. legal framework, regulation)	
Private Health Insurance policy framework (e.g. objective, population coverage, system description, types of coverage	
Market structure, conduct and performance (e.g. market concentration, barriers to entry, local vs. international, vertical integration, value-based health care)	
**Key findings related to scoping review question**	
Health System framework	
Eligibility	DOH issues an urgent request for all participants to update personal records in the Shafafiya system within 24 hours

Sector size	
User charges	
Institutional environment	
Legal framework	
Material regulation	
Prudential regulation	
Private Health Insurance policy framework	
Objective	
Population coverage	
System description	
Coverage type	
Market structure	
Concentration	
Barriers to entry	
Local vs international	
Vertical Integration	
Value Based Health Care	

Reviewer	Husein Reka
Date	20 January 2024
**Document information:**	
Name	Mandatory provision of medical treatment services in emergency medical cases
Author(s)	Department of Health Abu Dhabi
Year of publication	31 mar 2021
Country/Emirate	Abu Dhabi
Link	https://www.doh.gov.ae/-/media/E56993CCC0024B3E85218658760A90C2.ashx
Aim/purpose	Mandating emergency services
Record #	71
**Inclusion/Exclusion Criteria**	
Population	Private Health Insurance in Abu Dhabi
Concept	Transformation/Reforms of Abu Dhabi Private Health Insurance Scheme
Context	GCC Health System
**Review framework domains studied in literature on private health insurance transformation**	
Health System Framework (e.g. eligibility, sector size, user charges)	
Institutional environment (e.g. legal framework, regulation)	Emergency care treatment
Private Health Insurance policy framework (e.g. objective, population coverage, system description, types of coverage	
Market structure, conduct and performance (e.g. market concentration, barriers to entry, local vs. international, vertical integration, value-based health care)	
**Key findings related to scoping review question**	
Health System framework	
Eligibility	
Sector size	
User charges	

Institutional environment	
Legal framework	
Material regulation	DOH mandates provision of immediate necessary healthcare services in medical emergencies regardless of insurance status, and regardless of whether the healthcare provider is contracted or not contracted with the patient’s health insurance company
Prudential regulation	
Private Health Insurance policy framework	
Objective	
Population coverage	
System description	
Coverage type	
Market structure	
Concentration	
Barriers to entry	
Local vs international	
Vertical Integration	
Value Based Health Care	

Reviewer	Husein Reka
Date	20 January 2024
**Document information:**	
Name	Timeline for Implementation of Healthcare Reimbursement Reform in the Emirate of Abu Dhabi
Author(s)	Department of Health Abu Dhabi
Year of publication	24 Mar 2021
Country/Emirate	Abu Dhabi
Link	https://www.doh.gov.ae/-/media/1DC01332A3D74E8793B5E93C24DD6880.ashx
Aim/purpose	Changes on DoH Mandatory Tariff and inpatient services reimbursement in 2021
Record #	73
**Inclusion/Exclusion Criteria**	
Population	Private Health Insurance in Abu Dhabi
Concept	Transformation/Reforms of Abu Dhabi Private Health Insurance Scheme
Context	GCC Health System
**Review framework domains studied in literature on private health insurance transformation**	
Health System Framework (e.g. eligibility, sector size, user charges)	
Institutional environment (e.g. legal framework, regulation)	
Private Health Insurance policy framework (e.g. objective, population coverage, system description, types of coverage	
Market structure, conduct and performance (e.g. market concentration, barriers to entry, local vs. international, vertical integration, value-based health care)	Reimbursement reforms
**Key findings related to scoping review question**	
Health System framework	
Eligibility	
Sector size	

User charges	
Institutional environment	
Legal framework	
Material regulation	
Prudential regulation	
Private Health Insurance policy framework	
Objective	
Population coverage	
System description	
Coverage type	
Market structure	
Concentration	
Barriers to entry	
Local vs international	
Vertical Integration	
Value Based Health Care	DOH issues market consultation process for changes on Mandatory Tariff and inpatient services reimbursement in 2021

Reviewer	Husein Reka
Date	20 January 2024
**Document information:**	
Name	Extension of Submission Timeline for Claims Pertaining to ABM Health Services
Author(s)	Department of Health Abu Dhabi
Year of publication	15 Feb 2021
Country/Emirate	Abu Dhabi
Link	https://www.doh.gov.ae/-/media/9209613781244B77AEBEE518150A98BD.ashx
Aim/purpose	Extension of timelines for ABM
Record #	78
**Inclusion/Exclusion Criteria**	
Population	Private Health Insurance in Abu Dhabi
Concept	Transformation/Reforms of Abu Dhabi Private Health Insurance Scheme
Context	GCC Health System
**Review framework domains studied in literature on private health insurance transformation**	
Health System Framework (e.g. eligibility, sector size, user charges)	
Institutional environment (e.g. legal framework, regulation)	
Private Health Insurance policy framework (e.g. objective, population coverage, system description, types of coverage	
Market structure, conduct and performance (e.g. market concentration, barriers to entry, local vs. international, vertical integration, value-based health care)	
**Key findings related to scoping review question**	
Health System framework	
Eligibility	
Sector size	
User charges	

Institutional environment	
Legal framework	
Material regulation	
Prudential regulation	
Private Health Insurance policy framework	
Objective	
Population coverage	
System description	
Coverage type	
Market structure	
Concentration	
Barriers to entry	
Local vs international	
Vertical Integration	
Value Based Health Care	DOH issues extension for ABM claims submission

Reviewer	Husein Reka
Date	20 January 2024
**Document information:**	
Name	Extension of the Circular No.(23) of 2020 Regarding Activity Based Funded Mandates Network Expansion
Author(s)	Department of Health Abu Dhabi
Year of publication	26 Jan 2021
Country/Emirate	Abu Dhabi
Link	https://www.doh.gov.ae/-/media/5DC61ED076EE4AB8A8AA1CE4B0168DBC.ashx
Aim/purpose	Extension of timelines for ABM network expansion
Record #	79
**Inclusion/Exclusion Criteria**	
Population	Private Health Insurance in Abu Dhabi
Concept	Transformation/Reforms of Abu Dhabi Private Health Insurance Scheme
Context	GCC Health System
**Review framework domains studied in literature on private health insurance transformation**	
Health System Framework (e.g. eligibility, sector size, user charges)	
Institutional environment (e.g. legal framework, regulation)	
Private Health Insurance policy framework (e.g. objective, population coverage, system description, types of coverage	
Market structure, conduct and performance (e.g. market concentration, barriers to entry, local vs. international, vertical integration, value-based health care)	Healthcare reimbursement reforms
**Key findings related to scoping review question**	
Health System framework	
Eligibility	
Sector size	
User charges	

Institutional environment	
Legal framework	
Material regulation	
Prudential regulation	
Private Health Insurance policy framework	
Objective	
Population coverage	
System description	
Coverage type	
Market structure	
Concentration	
Barriers to entry	
Local vs international	
Vertical Integration	
Value Based Health Care	DOH extends timelines for ABM network expansion

Reviewer	Husein Reka
Date	20 January 2024
**Document information:**	
Name	Revision of Standard Provider Contract (SPC)
Author(s)	Department of Health Abu Dhabi
Year of publication	9 Dec 2020
Country/Emirate	Abu Dhabi
Link	https://www.doh.gov.ae/-/media/5DC61ED076EE4AB8A8AA1CE4B0168DBC.ashx
Aim/purpose	Revising SPC
Record #	82
**Inclusion/Exclusion Criteria**	
Population	Private Health Insurance in Abu Dhabi
Concept	Transformation/Reforms of Abu Dhabi Private Health Insurance Scheme
Context	GCC Health System
**Review framework domains studied in literature on private health insurance transformation**	
Health System Framework (e.g. eligibility, sector size, user charges)	
Institutional environment (e.g. legal framework, regulation)	SPC revision
Private Health Insurance policy framework (e.g. objective, population coverage, system description, types of coverage	
Market structure, conduct and performance (e.g. market concentration, barriers to entry, local vs. international, vertical integration, value-based health care)	
**Key findings related to scoping review question**	
Health System framework	
Eligibility	
Sector size	
User charges	
Institutional environment	

Legal framework	
Material regulation	DOH revises existing SPC mainly on the claim submission timelines for better management of health insurance products
Prudential regulation	
Private Health Insurance policy framework	
Objective	
Population coverage	
System description	
Coverage type	
Market structure	
Concentration	
Barriers to entry	
Local vs international	
Vertical Integration	
Value Based Health Care	

Reviewer	Husein Reka
Date	20 January 2024
**Document information:**	
Name	Mandatory registration in Health Insurance Electronic Window (living in Abu Dhabi)
Author(s)	Department of Health Abu Dhabi
Year of publication	15 Oct 2020
Country/Emirate	Abu Dhabi
Link	https://www.doh.gov.ae/-/media/20EB05E01D4C4954A6397A78FE2D5FA2.ashx
Aim/purpose	Government gateway registration for health insurance
Record #	84
**Inclusion/Exclusion Criteria**	
Population	Private Health Insurance in Abu Dhabi
Concept	Transformation/Reforms of Abu Dhabi Private Health Insurance Scheme
Context	GCC Health System
**Review framework domains studied in literature on private health insurance transformation**	
Health System Framework (e.g. eligibility, sector size, user charges)	
Institutional environment (e.g. legal framework, regulation)	Regulator requirements for payers
Private Health Insurance policy framework (e.g. objective, population coverage, system description, types of coverage	
Market structure, conduct and performance (e.g. market concentration, barriers to entry, local vs. international, vertical integration, value-based health care)	
**Key findings related to scoping review question**	
Health System framework	
Eligibility	
Sector size	
User charges	

Institutional environment	
Legal framework	
Material regulation	DOH request all health insurance companies to register in electronic platform to provide investors, visitors and expats with health insurance products
Prudential regulation	
Private Health Insurance policy framework	
Objective	
Population coverage	
System description	
Coverage type	
Market structure	
Concentration	
Barriers to entry	
Local vs international	
Vertical Integration	
Value Based Health Care	

Reviewer	Husein Reka
Date	20 January 2024
**Document information:**	
Name	Extension of Circular No (32) and Circular No (66) on Temporary ammendment of Article (3.2) of the Standard Provider Contract related to Administrative Procedures.
Author(s)	Department of Health Abu Dhabi
Year of publication	5 Oct 2020
Country/Emirate	Abu Dhabi
Link	https://www.doh.gov.ae/-/media/0EAA1051C8B94967AF2C052E1771FD8A.ashx
Aim/purpose	Regulator deadline extension
Record #	87
**Inclusion/Exclusion Criteria**	
Population	Private Health Insurance in Abu Dhabi
Concept	Transformation/Reforms of Abu Dhabi Private Health Insurance Scheme
Context	GCC Health System
**Review framework domains studied in literature on private health insurance transformation**	
Health System Framework (e.g. eligibility, sector size, user charges)	
Institutional environment (e.g. legal framework, regulation)	Extension of deadline for SPC administrative changes
Private Health Insurance policy framework (e.g. objective, population coverage, system description, types of coverage	
Market structure, conduct and performance (e.g. market concentration, barriers to entry, local vs. international, vertical integration, value-based health care)	
**Key findings related to scoping review question**	
Health System framework	
Eligibility	
Sector size	

User charges	
Institutional environment	
Legal framework	
Material regulation	DOH extends deadline for compliance with a previously issued circular on SPC
Prudential regulation	
Private Health Insurance policy framework	
Objective	
Population coverage	
System description	
Coverage type	
Market structure	
Concentration	
Barriers to entry	
Local vs international	
Vertical Integration	
Value Based Health Care	

Reviewer	Husein Reka
Date	20 January 2024
**Document information:**	
Name	Activity Based Funded Mandates Network Expansion
Author(s)	Department of Health Abu Dhabi
Year of publication	16 Aug 2020
Country/Emirate	Abu Dhabi
Link	https://www.doh.gov.ae/-/media/4F1E63D86E7D45C08EDD063CFF8EFDB3.ashx
Aim/purpose	Extension of ABM network and mandate
Record #	96
**Inclusion/Exclusion Criteria**	
Population	Private Health Insurance in Abu Dhabi
Concept	Transformation/Reforms of Abu Dhabi Private Health Insurance Scheme
Context	GCC Health System
**Review framework domains studied in literature on private health insurance transformation**	
Health System Framework (e.g. eligibility, sector size, user charges)	
Institutional environment (e.g. legal framework, regulation)	
Private Health Insurance policy framework (e.g. objective, population coverage, system description, types of coverage	
Market structure, conduct and performance (e.g. market concentration, barriers to entry, local vs. international, vertical integration, value-based health care)	Extension of ABM network and mandate
**Key findings related to scoping review question**	
Health System framework	
Eligibility	
Sector size	
User charges	
Institutional environment	

Legal framework	
Material regulation	
Prudential regulation	
Private Health Insurance policy framework	
Objective	
Population coverage	
System description	
Coverage type	
Market structure	
Concentration	
Barriers to entry	
Local vs international	
Vertical Integration	
Value Based Health Care	DOH expands network to include public and private hospital with Daman administering the Activity Based Funded Mandates

Reviewer	Husein Reka
Date	20 January 2024
**Document information:**	
Name	Extension of Circular No (32) on Temporary ammendment of Article 3.2 of the Standard Provider Contract related to Administrative Procedures
Author(s)	Department of Health Abu Dhabi
Year of publication	9 Jul 2020
Country/Emirate	Abu Dhabi
Link	https://www.doh.gov.ae/-/media/AC674A0F2A3E4CE49CF4DD0379B883DB.ashx
Aim/purpose	Extension of previous circular on SPC changes
Record #	99
**Inclusion/Exclusion Criteria**	
Population	Private Health Insurance in Abu Dhabi
Concept	Transformation/Reforms of Abu Dhabi Private Health Insurance Scheme
Context	GCC Health System
**Review framework domains studied in literature on private health insurance transformation**	
Health System Framework (e.g. eligibility, sector size, user charges)	
Institutional environment (e.g. legal framework, regulation)	
Private Health Insurance policy framework (e.g. objective, population coverage, system description, types of coverage	
Market structure, conduct and performance (e.g. market concentration, barriers to entry, local vs. international, vertical integration, value-based health care)	Extension of ABM network and mandate
**Key findings related to scoping review question**	
Health System framework	
Eligibility	
Sector size	

User charges	
Institutional environment	
Legal framework	
Material regulation	DOH extends by three months a deadline for compliance with a previously issued circular on SPC
Prudential regulation	
Private Health Insurance policy framework	
Objective	
Population coverage	
System description	
Coverage type	
Market structure	
Concentration	
Barriers to entry	
Local vs international	
Vertical Integration	
Value Based Health Care	

Reviewer	Husein Reka
Date	20 January 2024
**Document information:**	
Name	Decoupling between the enrollment (new / renew) in the health insurance scheme and the payment of fines resulting from the delay in the enrollment in the health insurance scheme
Author(s)	Department of Health Abu Dhabi
Year of publication	8 Jun 2020
Country/Emirate	Abu Dhabi
Link	https://www.doh.gov.ae/-/media/9E6368CF17C5488A8185B912B32A43EE.ashx
Aim/purpose	Guidance to payers on decoupling
Record #	101
**Inclusion/Exclusion Criteria**	
Population	Private Health Insurance in Abu Dhabi
Concept	Transformation/Reforms of Abu Dhabi Private Health Insurance Scheme
Context	GCC Health System
**Review framework domains studied in literature on private health insurance transformation**	
Health System Framework (e.g. eligibility, sector size, user charges)	Enforcement and penalties for mandatory health insurance
Institutional environment (e.g. legal framework, regulation)	
Private Health Insurance policy framework (e.g. objective, population coverage, system description, types of coverage	
Market structure, conduct and performance (e.g. market concentration, barriers to entry, local vs. international, vertical integration, value-based health care)	
**Key findings related to scoping review question**	
Health System framework	

Eligibility	Due to Covid-10 DOH asks to ease health insurance companies enrolment (new / renew) in the health insurance scheme for the violators after obtaining a written undertaking from the employer / sponsor that they will pay the fines incurred based on this enrolment before the next annual renewal of the health insurance policy and formally inform the violating party of the total amount of the fines incurred
Sector size	
User charges	
Institutional environment	
Legal framework	
Material regulation	
Prudential regulation	
Private Health Insurance policy framework	
Objective	
Population coverage	
System description	
Coverage type	
Market structure	
Concentration	
Barriers to entry	
Local vs international	
Vertical Integration	
Value Based Health Care	

Reviewer	Husein Reka
Date	20 January 2024
**Document information:**	
Name	Temporary amendment of Article 3.2 of the Standard Provider Contract related to Administrative Procedures
Author(s)	Department of Health Abu Dhabi
Year of publication	16 Apr 2020
Country/Emirate	Abu Dhabi
Link	https://www.doh.gov.ae/-/media/7AC6953F097B483CBBA107519371EDDD.ashx
Aim/purpose	Temporary amendments to claims submission time
Record #	104
**Inclusion/Exclusion Criteria**	
Population	Private Health Insurance in Abu Dhabi
Concept	Transformation/Reforms of Abu Dhabi Private Health Insurance Scheme
Context	GCC Health System
**Review framework domains studied in literature on private health insurance transformation**	
Health System Framework (e.g. eligibility, sector size, user charges)	
Institutional environment (e.g. legal framework, regulation)	Temporarily amendments to SPC
Private Health Insurance policy framework (e.g. objective, population coverage, system description, types of coverage	
Market structure, conduct and performance (e.g. market concentration, barriers to entry, local vs. international, vertical integration, value-based health care)	
**Key findings related to scoping review question**	
Health System framework	
Eligibility	
Sector size	
User charges	

Institutional environment	
Legal framework	
Material regulation	DOH extends claims submission timelines from 30 to 90 calendar days temporarily due to Covid-19
Prudential regulation	
Private Health Insurance policy framework	
Objective	
Population coverage	
System description	
Coverage type	
Market structure	
Concentration	
Barriers to entry	
Local vs international	
Vertical Integration	
Value Based Health Care	

Reviewer	Husein Reka
Date	20 January 2024
**Document information:**	
Name	Activity Based Funded Mandates Network Expansion
Author(s)	Department of Health Abu Dhabi
Year of publication	9 Apr 2020
Country/Emirate	Abu Dhabi
Link	https://www.doh.gov.ae/-/media/1C8CE3B441FC4734AAE583EDB29C8292.ashx
Aim/purpose	Expansion of ABF network
Record #	106
**Inclusion/Exclusion Criteria**	
Population	Private Health Insurance in Abu Dhabi
Concept	Transformation/Reforms of Abu Dhabi Private Health Insurance Scheme
Context	GCC Health System
**Review framework domains studied in literature on private health insurance transformation**	
Health System Framework (e.g. eligibility, sector size, user charges)	
Institutional environment (e.g. legal framework, regulation)	
Private Health Insurance policy framework (e.g. objective, population coverage, system description, types of coverage	
Market structure, conduct and performance (e.g. market concentration, barriers to entry, local vs. international, vertical integration, value-based health care)	Expansion of ABM provider network
**Key findings related to scoping review question**	
Health System framework	
Eligibility	
Sector size	
User charges	
Institutional environment	

Legal framework	
Material regulation	
Prudential regulation	
Private Health Insurance policy framework	
Objective	
Population coverage	
System description	
Coverage type	
Market structure	
Concentration	
Barriers to entry	
Local vs international	
Vertical Integration	
Value Based Health Care	Due to Covid-19 DOH expands network coverage to ensure the availability of capabilities and access to the necessary health services

Reviewer	Husein Reka
Date	20 January 2024
**Document information:**	
Name	Rules of Dispensing Medical Prescription for Patients Covered under Health insurance
Author(s)	Department of Health Abu Dhabi
Year of publication	24 Mar 2020
Country/Emirate	Abu Dhabi
Link	https://www.doh.gov.ae/-/media/A93D8BE55D5942C09E02B2DA99168FFB.ashx
Aim/purpose	Medicines dispensing rules and documentation requirements
Record #	110
**Inclusion/Exclusion Criteria**	
Population	Private Health Insurance in Abu Dhabi
Concept	Transformation/Reforms of Abu Dhabi Private Health Insurance Scheme
Context	GCC Health System
**Review framework domains studied in literature on private health insurance transformation**	
Health System Framework (e.g. eligibility, sector size, user charges)	
Institutional environment (e.g. legal framework, regulation)	Medicines dispensing requirements under health insurance
Private Health Insurance policy framework (e.g. objective, population coverage, system description, types of coverage	
Market structure, conduct and performance (e.g. market concentration, barriers to entry, local vs. international, vertical integration, value-based health care)	
**Key findings related to scoping review question**	
Health System framework	
Eligibility	

Sector size	
User charges	
Institutional environment	
Legal framework	
Material regulation	DOH issues circular to mandate procedures and documentation to dispense medicines under health insurance
Prudential regulation	
Private Health Insurance policy framework	
Objective	
Population coverage	
System description	
Coverage type	
Market structure	
Concentration	
Barriers to entry	
Local vs international	
Vertical Integration	
Value Based Health Care	

Reviewer	Husein Reka
Date	20 January 2024
**Document information:**	
Name	Mandatory listing requirement on Shafafiya for facilities licensed by DoH
Author(s)	Department of Health Abu Dhabi
Year of publication	11 Dec 2019
Country/Emirate	Abu Dhabi
Link	https://www.doh.gov.ae/-/media/024037628A434D9AA82442CEBECED6CC.ashx
Aim/purpose	Compliance with dealing with accredited only providers in Shafafiya
Record #	115
**Inclusion/Exclusion Criteria**	
Population	Private Health Insurance in Abu Dhabi
Concept	Transformation/Reforms of Abu Dhabi Private Health Insurance Scheme
Context	GCC Health System
**Review framework domains studied in literature on private health insurance transformation**	
Health System Framework (e.g. eligibility, sector size, user charges)	
Institutional environment (e.g. legal framework, regulation)	Compliance with provider accreditation
Private Health Insurance policy framework (e.g. objective, population coverage, system description, types of coverage	
Market structure, conduct and performance (e.g. market concentration, barriers to entry, local vs. international, vertical integration, value-based health care)	
**Key findings related to scoping review question**	
Health System framework	
Eligibility	
Sector size	

User charges	
Institutional environment	
Legal framework	
Material regulation	DOH enforces quality accreditation requirements for providers via Shafafiya as a prerequisite for payer payments for services
Prudential regulation	
Private Health Insurance policy framework	
Objective	
Population coverage	
System description	
Coverage type	
Market structure	
Concentration	
Barriers to entry	
Local vs international	
Vertical Integration	
Value Based Health Care	

Reviewer	Husein Reka
Date	20 January 2024
**Document information:**	
Name	Mandatory listing requirement on Shafafiya for facilities licensed by DoH
Author(s)	Department of Health Abu Dhabi
Year of publication	11 Dec 2019
Country/Emirate	Abu Dhabi
Link	https://www.doh.gov.ae/-/media/024037628A434D9AA82442CEBECED6CC.ashx
Aim/purpose	Compliance with dealing with accredited only providers in Shafafiya
Record #	115
**Inclusion/Exclusion Criteria**	
Population	Private Health Insurance in Abu Dhabi
Concept	Transformation/Reforms of Abu Dhabi Private Health Insurance Scheme
Context	GCC Health System
**Review framework domains studied in literature on private health insurance transformation**	
Health System Framework (e.g. eligibility, sector size, user charges)	
Institutional environment (e.g. legal framework, regulation)	Compliance with provider accreditation
Private Health Insurance policy framework (e.g. objective, population coverage, system description, types of coverage	
Market structure, conduct and performance (e.g. market concentration, barriers to entry, local vs. international, vertical integration, value-based health care)	
**Key findings related to scoping review question**	
Health System framework	
Eligibility	
Sector size	

User charges	
Institutional environment	
Legal framework	
Material regulation	DOH enforces quality accreditation requirements for providers via Shafafiya as a prerequisite for payer payments for services
Prudential regulation	
Private Health Insurance policy framework	
Objective	
Population coverage	
System description	
Coverage type	
Market structure	
Concentration	
Barriers to entry	
Local vs international	
Vertical Integration	
Value Based Health Care	

Reviewer	Husein Reka
Date	20 January 2024
**Document information:**	
Name	Providing Patients with Medical Services Invoices
Author(s)	Department of Health Abu Dhabi
Year of publication	13 Mar 2019
Country/Emirate	Abu Dhabi
Link	https://www.doh.gov.ae/-/media/57CAF95108224AE2A05013B7B81E2CD2.ashx
Aim/purpose	Requirement to issue invoices to patients
Record #	119
**Inclusion/Exclusion Criteria**	
Population	Private Health Insurance in Abu Dhabi
Concept	Transformation/Reforms of Abu Dhabi Private Health Insurance Scheme
Context	GCC Health System
**Review framework domains studied in literature on private health insurance transformation**	
Health System Framework (e.g. eligibility, sector size, user charges)	
Institutional environment (e.g. legal framework, regulation)	Requirement to issue invoice for rendered services
Private Health Insurance policy framework (e.g. objective, population coverage, system description, types of coverage	
Market structure, conduct and performance (e.g. market concentration, barriers to entry, local vs. international, vertical integration, value-based health care)	
**Key findings related to scoping review question**	
Health System framework	
Eligibility	
Sector size	
User charges	
Institutional environment	

Legal framework	
Material regulation	DOH requires providers to issue medical invoices to patients for rendered services in order to increase awareness on health care cost and avoidance of duplication of services
Prudential regulation	
Private Health Insurance policy framework	
Objective	
Population coverage	
System description	
Coverage type	
Market structure	
Concentration	
Barriers to entry	
Local vs international	
Vertical Integration	
Value Based Health Care	

Reviewer	Husein Reka
Date	20 January 2024
**Document information:**	
Name	New Mechanism of Dispensing Generic Medicines
Author(s)	Department of Health Abu Dhabi
Year of publication	22 Jul 2018
Country/Emirate	Abu Dhabi
Link	https://www.doh.gov.ae/-/media/2FAFD8D1079040DDB80FC504D0260570.ashx
Aim/purpose	Introduction of reference prices for generic medicines
Record #	121
**Inclusion/Exclusion Criteria**	
Population	Private Health Insurance in Abu Dhabi
Concept	Transformation/Reforms of Abu Dhabi Private Health Insurance Scheme
Context	GCC Health System
**Review framework domains studied in literature on private health insurance transformation**	
Health System Framework (e.g. eligibility, sector size, user charges)	
Institutional environment (e.g. legal framework, regulation)	
Private Health Insurance policy framework (e.g. objective, population coverage, system description, types of coverage	
Market structure, conduct and performance (e.g. market concentration, barriers to entry, local vs. international, vertical integration, value-based health care)	Reference pricing for generic medicines
**Key findings related to scoping review question**	
Health System framework	
Eligibility	
Sector size	
User charges	
Institutional environment	

Legal framework	
Material regulation	
Prudential regulation	
Private Health Insurance policy framework	
Objective	
Population coverage	
System description	
Coverage type	
Market structure	
Concentration	
Barriers to entry	
Local vs international	
Vertical Integration	
Value Based Health Care	DOH introduces reference pricing for medicines and choice for patients to chose medicines with co-payments

Reviewer	Husein Reka
Date	20 January 2024
**Document information:**	
Name	Validity of healthcare Licenses as a Precondition for completeness of Claims
Author(s)	Department of Health Abu Dhabi
Year of publication	17 Feb 2018
Country/Emirate	Abu Dhabi
Link	https://www.doh.gov.ae/-/media/F88FE1D883FC4BAC9CAC78975FF7D4F8.ashx
Aim/purpose	Not available
Record #	124
**Inclusion/Exclusion Criteria**	
Population	Private Health Insurance in Abu Dhabi
Concept	Transformation/Reforms of Abu Dhabi Private Health Insurance Scheme
Context	GCC Health System
**Review framework domains studied in literature on private health insurance transformation**	
Health System Framework (e.g. eligibility, sector size, user charges)	
Institutional environment (e.g. legal framework, regulation)	
Private Health Insurance policy framework (e.g. objective, population coverage, system description, types of coverage	
Market structure, conduct and performance (e.g. market concentration, barriers to entry, local vs. international, vertical integration, value-based health care)	
**Key findings related to scoping review question**	
Health System framework	
Eligibility	
Sector size	
User charges	

Institutional environment	
Legal framework	
Material regulation	
Prudential regulation	
Private Health Insurance policy framework	
Objective	
Population coverage	
System description	
Coverage type	
Market structure	
Concentration	
Barriers to entry	
Local vs international	
Vertical Integration	
Value Based Health Care	

Reviewer	Husein Reka
Date	20 January 2024
**Document information:**	
Name	Mandatory Submission of Unified Number to Person Register
Author(s)	Department of Health Abu Dhabi
Year of publication	17 Feb 2018
Country/Emirate	Abu Dhabi
Link	https://www.doh.gov.ae/-/media/DFD064E1559D41AE9F0D654A6A3D849A.ashx
Aim/purpose	Mandatory requirement to register in person register
Record #	125
**Inclusion/Exclusion Criteria**	
Population	Private Health Insurance in Abu Dhabi
Concept	Transformation/Reforms of Abu Dhabi Private Health Insurance Scheme
Context	GCC Health System
**Review framework domains studied in literature on private health insurance transformation**	
Health System Framework (e.g. eligibility, sector size, user charges)	Improving eligibility information
Institutional environment (e.g. legal framework, regulation)	
Private Health Insurance policy framework (e.g. objective, population coverage, system description, types of coverage	
Market structure, conduct and performance (e.g. market concentration, barriers to entry, local vs. international, vertical integration, value-based health care)	
**Key findings related to scoping review question**	
Health System framework	
Eligibility	DOH requires all scheme participants to register personal details in the personal registry of Shafafiya
Sector size	

User charges	
Institutional environment	
Legal framework	
Material regulation	
Prudential regulation	
Private Health Insurance policy framework	
Objective	
Population coverage	
System description	
Coverage type	
Market structure	
Concentration	
Barriers to entry	
Local vs international	
Vertical Integration	
Value Based Health Care	

Reviewer	Husein Reka
Date	20 January 2024
**Document information:**	
Name	Standard Contract governing the Contractual Relation between Healthcare Service Providers, Health Insurance Companies and TPA
Author(s)	Department of Health Abu Dhabi
Year of publication	31 Jan 2018
Country/Emirate	Abu Dhabi
Link	https://www.doh.gov.ae/-/media/DFD064E1559D41AE9F0D654A6A3D849A.ashx
Aim/purpose	Tackling provider abuse practice
Record #	126
**Inclusion/Exclusion Criteria**	
Population	Private Health Insurance in Abu Dhabi
Concept	Transformation/Reforms of Abu Dhabi Private Health Insurance Scheme
Context	GCC Health System
**Review framework domains studied in literature on private health insurance transformation**	
Health System Framework (e.g. eligibility, sector size, user charges)	
Institutional environment (e.g. legal framework, regulation)	
Private Health Insurance policy framework (e.g. objective, population coverage, system description, types of coverage	
Market structure, conduct and performance (e.g. market concentration, barriers to entry, local vs. international, vertical integration, value-based health care)	Overutilization abuse by providers
**Key findings related to scoping review question**	
Health System framework	
Eligibility	
Sector size	

User charges	
Institutional environment	
Legal framework	
Material regulation	
Prudential regulation	
Private Health Insurance policy framework	
Objective	
Population coverage	
System description	
Coverage type	
Market structure	
Concentration	
Barriers to entry	
Local vs international	
Vertical Integration	
Value Based Health Care	DOH requested payer to report cases of abuse through overutilization by providers

Reviewer	Husein Reka
Date	20 January 2024
**Document information:**	
Name	Segment Financial Reporting of Health Insurance for the Emirate of Abu Dhabi
Author(s)	Department of Health Abu Dhabi
Year of publication	28 May 2013
Country/Emirate	Abu Dhabi
Link	https://www.doh.gov.ae/-/media/9494A35B36BC456CADA34A9D2C554079.ashx
Aim/purpose	Mandatory financial reporting to regulator
Record #	129
**Inclusion/Exclusion Criteria**	
Population	Private Health Insurance in Abu Dhabi
Concept	Transformation/Reforms of Abu Dhabi Private Health Insurance Scheme
Context	GCC Health System
**Review framework domains studied in literature on private health insurance transformation**	
Health System Framework (e.g. eligibility, sector size, user charges)	
Institutional environment (e.g. legal framework, regulation)	Financial reporting by payers
Private Health Insurance policy framework (e.g. objective, population coverage, system description, types of coverage	
Market structure, conduct and performance (e.g. market concentration, barriers to entry, local vs. international, vertical integration, value-based health care)	
**Key findings related to scoping review question**	
Health System framework	
Eligibility	
Sector size	
User charges	

Institutional environment	
Legal framework	
Material regulation	
Prudential regulation	Health Authority of Abu Dhabi mandated regular quarterly financial reporting by insurance companies
Private Health Insurance policy framework	
Objective	
Population coverage	
System description	
Coverage type	
Market structure	
Concentration	
Barriers to entry	
Local vs international	
Vertical Integration	
Value Based Health Care	

Reviewer	Husein Reka
Date	20 January 2024
**Document information:**	
Name	Printing of Health Insurance Products Approval Number
Author(s)	Department of Health Abu Dhabi
Year of publication	27 Jul 2010
Country/Emirate	Abu Dhabi
Link	https://www.doh.gov.ae/-/media/95D0CF8505974702806D3F082F359D53.ashx
Aim/purpose	Insurance product approval number printing
Record #	131
**Inclusion/Exclusion Criteria**	
Population	Private Health Insurance in Abu Dhabi
Concept	Transformation/Reforms of Abu Dhabi Private Health Insurance Scheme
Context	GCC Health System
**Review framework domains studied in literature on private health insurance transformation**	
Health System Framework (e.g. eligibility, sector size, user charges)	
Institutional environment (e.g. legal framework, regulation)	Product approval regulation
Private Health Insurance policy framework (e.g. objective, population coverage, system description, types of coverage	
Market structure, conduct and performance (e.g. market concentration, barriers to entry, local vs. international, vertical integration, value-based health care)	
**Key findings related to scoping review question**	
Health System framework	
Eligibility	
Sector size	
User charges	
Institutional environment	

Legal framework	
Material regulation	HAAD mandates product approval printing issues by HAAD for each health insurance policy issued by insurance companies
Prudential regulation	
Private Health Insurance policy framework	
Objective	
Population coverage	
System description	
Coverage type	
Market structure	
Concentration	
Barriers to entry	
Local vs international	
Vertical Integration	
Value Based Health Care	

Reviewer	Husein Reka
Date	20 January 2024
**Document information:**	
Name	Licensing branches of insurance companies in UAE
Author(s)	Department of Health Abu Dhabi
Year of publication	4 May 2010
Country/Emirate	Abu Dhabi
Link	https://www.doh.gov.ae/-/media/A4FECF6BCCC34790BA5E12E1560AFA27.ashx
Aim/purpose	Insurance product approval number printing
Record #	133
**Inclusion/Exclusion Criteria**	
Population	Private Health Insurance in Abu Dhabi
Concept	Transformation/Reforms of Abu Dhabi Private Health Insurance Scheme
Context	GCC Health System
**Review framework domains studied in literature on private health insurance transformation**	
Health System Framework (e.g. eligibility, sector size, user charges)	
Institutional environment (e.g. legal framework, regulation)	Registration with regulator
Private Health Insurance policy framework (e.g. objective, population coverage, system description, types of coverage	
Market structure, conduct and performance (e.g. market concentration, barriers to entry, local vs. international, vertical integration, value-based health care)	
**Key findings related to scoping review question**	
Health System framework	
Eligibility	
Sector size	
User charges	
Institutional environment	

Legal framework	
Material regulation	HAAD requires all healthcare providers, TPAs, and brokers branches operating in Abu Dhabi to register with them
Prudential regulation	
Private Health Insurance policy framework	
Objective	
Population coverage	
System description	
Coverage type	
Market structure	
Concentration	
Barriers to entry	
Local vs international	
Vertical Integration	
Value Based Health Care	

Reviewer	Husein Reka
Date	20 January 2024
**Document information:**	
Name	Dual eligibility
Author(s)	Department of Health Abu Dhabi
Year of publication	22 Oct 2009
Country/Emirate	Abu Dhabi
Link	https://www.doh.gov.ae/-/media/4B20629189CB401F9FE480C7E3A0F770.ashx
Aim/purpose	Prohibition of dual eligibility
Record #	134
**Inclusion/Exclusion Criteria**	
Population	Private Health Insurance in Abu Dhabi
Concept	Transformation/Reforms of Abu Dhabi Private Health Insurance Scheme
Context	GCC Health System
**Review framework domains studied in literature on private health insurance transformation**	
Health System Framework (e.g. eligibility, sector size, user charges)	Dual eligibility
Institutional environment (e.g. legal framework, regulation)	
Private Health Insurance policy framework (e.g. objective, population coverage, system description, types of coverage	
Market structure, conduct and performance (e.g. market concentration, barriers to entry, local vs. international, vertical integration, value-based health care)	
**Key findings related to scoping review question**	
Health System framework	
Eligibility	Dual eligibility for private health insurance for Nationals prohibited
Sector size	
User charges	

Institutional environment	
Legal framework	
Material regulation	
Prudential regulation	
Private Health Insurance policy framework	
Objective	
Population coverage	
System description	
Coverage type	
Market structure	
Concentration	
Barriers to entry	
Local vs international	
Vertical Integration	
Value Based Health Care	

Reviewer	Husein Reka
Date	20 January 2024
**Document information:**	
Name	Circumcision addition as benefit
Author(s)	Department of Health Abu Dhabi
Year of publication	28 Jan 2009
Country/Emirate	Abu Dhabi
Link	https://www.doh.gov.ae/-/media/8B1511BB703D41C1ADAED061E6FA9F40.ashx
Aim/purpose	Addition of service to basic policy
Record #	136
**Inclusion/Exclusion Criteria**	
Population	Private Health Insurance in Abu Dhabi
Concept	Transformation/Reforms of Abu Dhabi Private Health Insurance Scheme
Context	GCC Health System
**Review framework domains studied in literature on private health insurance transformation**	
Health System Framework (e.g. eligibility, sector size, user charges)	Benefit package eligibility
Institutional environment (e.g. legal framework, regulation)	
Private Health Insurance policy framework (e.g. objective, population coverage, system description, types of coverage	
Market structure, conduct and performance (e.g. market concentration, barriers to entry, local vs. international, vertical integration, value-based health care)	
**Key findings related to scoping review question**	
Health System framework	
Eligibility	HAAD decided to include circumcision as a benefit in the basic policy
Sector size	
User charges	

Institutional environment	
Legal framework	
Material regulation	
Prudential regulation	
Private Health Insurance policy framework	
Objective	
Population coverage	
System description	
Coverage type	
Market structure	
Concentration	
Barriers to entry	
Local vs international	
Vertical Integration	
Value Based Health Care	

Reviewer	Husein Reka
Date	20 January 2024
**Document information:**	
Name	Health Insurance Policy for emergency
Author(s)	Department of Health Abu Dhabi
Year of publication	23 Nov 2006
Country/Emirate	Abu Dhabi
Link	https://www.doh.gov.ae/-/media/2179D6C9FC70461385FC6019D797DF83.ashx
Aim/purpose	Explanation of emergency services under health insurance
Record #	137
**Inclusion/Exclusion Criteria**	
Population	Private Health Insurance in Abu Dhabi
Concept	Transformation/Reforms of Abu Dhabi Private Health Insurance Scheme
Context	GCC Health System
**Review framework domains studied in literature on private health insurance transformation**	
Health System Framework (e.g. eligibility, sector size, user charges)	Emergency services under health insurance
Institutional environment (e.g. legal framework, regulation)	
Private Health Insurance policy framework (e.g. objective, population coverage, system description, types of coverage	
Market structure, conduct and performance (e.g. market concentration, barriers to entry, local vs. international, vertical integration, value-based health care)	
**Key findings related to scoping review question**	
Health System framework	
Eligibility	HAAD issued circular explaining emergency services scope and eligibility under mandatory health insurance scheme

Sector size	
User charges	
Institutional environment	
Legal framework	
Material regulation	
Prudential regulation	
Private Health Insurance policy framework	
Objective	
Population coverage	
System description	
Coverage type	
Market structure	
Concentration	
Barriers to entry	
Local vs international	
Vertical Integration	
Value Based Health Care	

Reviewer	Husein Reka
Date	20 January 2024
**Document information:**	
Name	Law no. 23 Concerning Health Insurance in the Emirate of Abu Dhabi and its Executive Regulations
Author(s)	The Executive Council of the Emirate of Abu Dhabi
Year of publication	10 Sep 2005
Country/Emirate	Abu Dhabi
Link	https://www.doh.gov.ae/-/media/C0321C357F884B519CCEE9726F75E779.ashx
Aim/purpose	Establishing mandatory health insurance
Record #	138
**Inclusion/Exclusion Criteria**	
Population	Private Health Insurance in Abu Dhabi
Concept	Transformation/Reforms of Abu Dhabi Private Health Insurance Scheme
Context	GCC Health System
**Review framework domains studied in literature on private health insurance transformation**	
Health System Framework (e.g. eligibility, sector size, user charges)	
Institutional environment (e.g. legal framework, regulation)	Mandatory health insurance legislation
Private Health Insurance policy framework (e.g. objective, population coverage, system description, types of coverage	
Market structure, conduct and performance (e.g. market concentration, barriers to entry, local vs. international, vertical integration, value-based health care)	
**Key findings related to scoping review question**	
Health System framework	
Eligibility	
Sector size	
User charges	

Institutional environment	
Legal framework	The Executive Council of the Emirate of Abu Dhabi promulgated Law no. 23 of 2005 and accompanying implementing regulations to establish the mandatory health insurance scheme in Abu Dhabi
Material regulation	
Prudential regulation	
Private Health Insurance policy framework	
Objective	
Population coverage	
System description	
Coverage type	
Market structure	
Concentration	
Barriers to entry	
Local vs international	
Vertical Integration	
Value Based Health Care	

Reviewer	Husein Reka
Date	20 January 2024
**Document information:**	
Name	Law No. (22) of 2018 concerning the Amendment of Some Provisions of Law No. (23) of 2005 concerning Health Insurance in the Emirate of Abu Dhabi
Author(s)	The Executive Council of the Emirate of Abu Dhabi
Year of publication	22 Nov 2018
Country/Emirate	Abu Dhabi
Link	https://www.doh.gov.ae/-/media/C0321C357F884B519CCEE9726F75E779.ashx
Aim/purpose	Amendments to the mandatory health insurance law
Record #	139
**Inclusion/Exclusion Criteria**	
Population	Private Health Insurance in Abu Dhabi
Concept	Transformation/Reforms of Abu Dhabi Private Health Insurance Scheme
Context	GCC Health System
**Review framework domains studied in literature on private health insurance transformation**	
Health System Framework (e.g. eligibility, sector size, user charges)	
Institutional environment (e.g. legal framework, regulation)	Mandatory health insurance legislation amendments
Private Health Insurance policy framework (e.g. objective, population coverage, system description, types of coverage	
Market structure, conduct and performance (e.g. market concentration, barriers to entry, local vs. international, vertical integration, value-based health care)	
**Key findings related to scoping review question**	
Health System framework	
Eligibility	
Sector size	

User charges	
Institutional environment	
Legal framework	The Executive Council of the Emirate of Abu Dhabi promulgated Law no. 22 of 2018 amending mandatory health insurance law increasing violation of law penalties to AED 10 million for non-compliance with the law, regulations and circulars
Material regulation	
Prudential regulation	
Private Health Insurance policy framework	
Objective	
Population coverage	
System description	
Coverage type	
Market structure	
Concentration	
Barriers to entry	
Local vs international	
Vertical Integration	
Value Based Health Care	

Reviewer	Husein Reka
Date	20 January 2024
**Document information:**	
Name	Chairman of the Executive Council Resolution No. (25) of 2006 Issuing the Executive Regulations of Law No. 23 of 2005 Concerning Health Insurance in the Emirate of Abu Dhabi
Author(s)	The Executive Council of the Emirate of Abu Dhabi
Year of publication	4 Jun 2006
Country/Emirate	Abu Dhabi
Link	https://www.doh.gov.ae/-/media/C0321C357F884B519CCEE9726F75E779.ashx
Aim/purpose	Implementing regulations of health insurance law
Record #	140
**Inclusion/Exclusion Criteria**	
Population	Private Health Insurance in Abu Dhabi
Concept	Transformation/Reforms of Abu Dhabi Private Health Insurance Scheme
Context	GCC Health System
**Review framework domains studied in literature on private health insurance transformation**	
Health System Framework (e.g. eligibility, sector size, user charges)	
Institutional environment (e.g. legal framework, regulation)	Mandatory health insurance implementing regulations
Private Health Insurance policy framework (e.g. objective, population coverage, system description, types of coverage	
Market structure, conduct and performance (e.g. market concentration, barriers to entry, local vs. international, vertical integration, value-based health care)	
**Key findings related to scoping review question**	
Health System framework	
Eligibility	

Sector size	
User charges	
Institutional environment	
Legal framework	The Chairman of Executive Council of the Emirate of Abu Dhabi promulgated Implementing Regulations of the mandatory health insurance scheme in Abu Dhabi
Material regulation	
Prudential regulation	
Private Health Insurance policy framework	
Objective	
Population coverage	
System description	
Coverage type	
Market structure	
Concentration	
Barriers to entry	
Local vs international	
Vertical Integration	
Value Based Health Care	

Reviewer	Husein Reka
Date	20 January 2024
**Document information:**	
Name	Chairman of the Executive Council Resolution No. (83) of 2007 Concerning the Implementation of Health Insurance in respect of Nationals and Persons of Equivalent Status in the Emirate of Abu Dhabi
Author(s)	The Executive Council of the Emirate of Abu Dhabi
Year of publication	13 Dec 2007
Country/Emirate	Abu Dhabi
Link	https://www.doh.gov.ae/-/media/C0321C357F884B519CCEE9726F75E779.ashx
Aim/purpose	Extension of mandatory health insurance for citizens
Record #	141
**Inclusion/Exclusion Criteria**	
Population	Private Health Insurance in Abu Dhabi
Concept	Transformation/Reforms of Abu Dhabi Private Health Insurance Scheme
Context	GCC Health System
**Review framework domains studied in literature on private health insurance transformation**	
Health System Framework (e.g. eligibility, sector size, user charges)	
Institutional environment (e.g. legal framework, regulation)	Mandatory health insurance for citizens
Private Health Insurance policy framework (e.g. objective, population coverage, system description, types of coverage	
Market structure, conduct and performance (e.g. market concentration, barriers to entry, local vs. international, vertical integration, value-based health care)	
**Key findings related to scoping review question**	
Health System framework	
Eligibility	

Sector size	
User charges	
Institutional environment	
Legal framework	The Chairman of Executive Council of the Emirate of Abu Dhabi issued a decision on extending health insurance for citizens and persons of similar status
Material regulation	
Prudential regulation	
Private Health Insurance policy framework	
Objective	
Population coverage	
System description	
Coverage type	
Market structure	
Concentration	
Barriers to entry	
Local vs international	
Vertical Integration	
Value Based Health Care	

Reviewer	Husein Reka
Date	20 January 2024
**Document information:**	
Name	Chairman of the Executive Council Resolution No. (47) of 2008 Amending Certain Provisions of Resolution No. (25) of 2006 Issuing the Executive Regulations of Law No. 23 of 2005 Concerning Health Insurance in the Emirate of Abu Dhabi
Author(s)	The Executive Council of the Emirate of Abu Dhabi
Year of publication	19 Jun 2008
Country/Emirate	Abu Dhabi
Link	https://www.doh.gov.ae/-/media/C0321C357F884B519CCEE9726F75E779.ashx
Aim/purpose	Introduction of basic mandatory health insurance for blue collar workers
Record #	142
**Inclusion/Exclusion Criteria**	
Population	Private Health Insurance in Abu Dhabi
Concept	Transformation/Reforms of Abu Dhabi Private Health Insurance Scheme
Context	GCC Health System
**Review framework domains studied in literature on private health insurance transformation**	
Health System Framework (e.g. eligibility, sector size, user charges)	Mandatory health insurance for low wage workers
Institutional environment (e.g. legal framework, regulation)	
Private Health Insurance policy framework (e.g. objective, population coverage, system description, types of coverage	
Market structure, conduct and performance (e.g. market concentration, barriers to entry, local vs. international, vertical integration, value-based health care)	
**Key findings related to scoping review question**	

Health System framework	
Eligibility	Basic health insurance policy only for the following categories:<br>- People with limited income and their sponsors who receive total salaries according to the following:<br>4,000 dirhams per month with housing, or 5,000 dirhams per month without housing.<br>- Sponsored expatriate residents who are not covered by health insurance by the employer.<br>- Non-citizens who are indigent and in critical humanitarian cases, provided that the Authority sets the necessary controls for this As of July 1, 2008, the exclusive right of the National Health Insurance Company (Daman) to provide compulsory and voluntary health insurance services to all non-citizen government employees and their equivalents and their resident family members, as stipulated in Clause (1) of Article (4) of Emiri Decree No. (39) of 2005 referred to, and Clause (3) of Article (19) From the aforementioned Executive Council Chairman’s Decision No. (25) of 2006
Sector size	
User charges	
Institutional environment	
Legal framework	
Material regulation	
Prudential regulation	
Private Health Insurance policy framework	
Objective	
Population coverage	
System description	
Coverage type	
Market structure	
Concentration	

Barriers to entry	
Local vs international	
Vertical Integration	
Value Based Health Care	

Reviewer	Husein Reka
Date	20 January 2024
**Document information:**	
Name	Resolution No. (CO-014/10) Amending Certain Provisions of Resolution No. (25) of 2006 Issuing the Executive Regulations of Law No. 23 of 2005 Concerning Health Insurance in the Emirate of Abu Dhabi
Author(s)	Health Authority of Abu Dhabi
Year of publication	22 Mar 2010
Country/Emirate	Abu Dhabi
Link	https://www.doh.gov.ae/-/media/C0321C357F884B519CCEE9726F75E779.ashx
Aim/purpose	Introduction of work related injuries in basic mandatory health insurance
Record #	143
**Inclusion/Exclusion Criteria**	
Population	Private Health Insurance in Abu Dhabi
Concept	Transformation/Reforms of Abu Dhabi Private Health Insurance Scheme
Context	GCC Health System
**Review framework domains studied in literature on private health insurance transformation**	
Health System Framework (e.g. eligibility, sector size, user charges)	Benefit package expansion
Institutional environment (e.g. legal framework, regulation)	
Private Health Insurance policy framework (e.g. objective, population coverage, system description, types of coverage	
Market structure, conduct and performance (e.g. market concentration, barriers to entry, local vs. international, vertical integration, value-based health care)	
**Key findings related to scoping review question**	
Health System framework	

Eligibility	Work related injuries and diseases inclusion in benefit package
Sector size	
User charges	
Institutional environment	
Legal framework	
Material regulation	
Prudential regulation	
Private Health Insurance policy framework	
Objective	
Population coverage	
System description	
Coverage type	
Market structure	
Concentration	
Barriers to entry	
Local vs international	
Vertical Integration	
Value Based Health Care	

Reviewer	Husein Reka
Date	20 January 2024
**Document information:**	
Name	Resolution No. (CO-019/10) Amending Certain Provisions of Resolution No. (25) of 2006 Issuing the Executive Regulations of Law No. 23 of 2005 Concerning Health Insurance in the Emirate of Abu Dhabi
Author(s)	Health Authority of Abu Dhabi
Year of publication	24 May 2010
Country/Emirate	Abu Dhabi
Link	https://www.doh.gov.ae/-/media/C0321C357F884B519CCEE9726F75E779.ashx
Aim/purpose	Introduction of work related injuries in basic mandatory health insurance
Record #	144
**Inclusion/Exclusion Criteria**	
Population	Private Health Insurance in Abu Dhabi
Concept	Transformation/Reforms of Abu Dhabi Private Health Insurance Scheme
Context	GCC Health System
**Review framework domains studied in literature on private health insurance transformation**	
Health System Framework (e.g. eligibility, sector size, user charges)	Benefit package expansion
Institutional environment (e.g. legal framework, regulation)	
Private Health Insurance policy framework (e.g. objective, population coverage, system description, types of coverage	
Market structure, conduct and performance (e.g. market concentration, barriers to entry, local vs. international, vertical integration, value-based health care)	
**Key findings related to scoping review question**	
Health System framework	

Eligibility	Medical treatment services for work-related injuries and diseases specified in Law No. (8) of 1980 regarding labor relations, its amendments, and the applicable laws and decisions
Sector size	
User charges	
Institutional environment	
Legal framework	
Material regulation	
Prudential regulation	
Private Health Insurance policy framework	
Objective	
Population coverage	
System description	
Coverage type	
Market structure	
Concentration	
Barriers to entry	
Local vs international	
Vertical Integration	
Value Based Health Care	

Reviewer	Husein Reka
Date	20 January 2024
**Document information:**	
Name	DoH Insurers Manual
Author(s)	Health Authority of Abu Dhabi
Year of publication	1 Nov 2017
Country/Emirate	Abu Dhabi
Link	https://www.doh.gov.ae/-/media/09AEC8C5B6D34AA88DE110F63641B863.ashx
Aim/purpose	Manual providing standards and requirements for all participants of mandatory health insurance scheme
Record #	149
**Inclusion/Exclusion Criteria**	
Population	Private Health Insurance in Abu Dhabi
Concept	Transformation/Reforms of Abu Dhabi Private Health Insurance Scheme
Context	GCC Health System
**Review framework domains studied in literature on private health insurance transformation**	
Health System Framework (e.g. eligibility, sector size, user charges)	
Institutional environment (e.g. legal framework, regulation)	Manual to explain regulatory standards for the health insurance scheme
Private Health Insurance policy framework (e.g. objective, population coverage, system description, types of coverage	
Market structure, conduct and performance (e.g. market concentration, barriers to entry, local vs. international, vertical integration, value-based health care)	
**Key findings related to scoping review question**	
Health System framework	
Eligibility	
Sector size	

User charges	
Institutional environment	
Legal framework	Detailed standard and requirements for all participants of abu dhabi health insurance scheme
Material regulation	
Prudential regulation	
Private Health Insurance policy framework	
Objective	
Population coverage	
System description	
Coverage type	
Market structure	
Concentration	
Barriers to entry	
Local vs international	
Vertical Integration	
Value Based Health Care	

Reviewer	Husein Reka
Date	20 January 2024
**Document information:**	
Name	HAAD Standard for Recovery of Payment for Healthcare Services under the Health Insurance Scheme
Author(s)	Health Authority of Abu Dhabi
Year of publication	9 Jul 2015
Country/Emirate	Abu Dhabi
Link	https://www.doh.gov.ae/-/media/38256EA460354F469F72231FC57C329E.ashx
Aim/purpose	Rules for recovery of payments for claims
Record #	150
**Inclusion/Exclusion Criteria**	
Population	Private Health Insurance in Abu Dhabi
Concept	Transformation/Reforms of Abu Dhabi Private Health Insurance Scheme
Context	GCC Health System
**Review framework domains studied in literature on private health insurance transformation**	
Health System Framework (e.g. eligibility, sector size, user charges)	
Institutional environment (e.g. legal framework, regulation)	Payer and provider standards for payments recovery
Private Health Insurance policy framework (e.g. objective, population coverage, system description, types of coverage	
Market structure, conduct and performance (e.g. market concentration, barriers to entry, local vs. international, vertical integration, value-based health care)	
**Key findings related to scoping review question**	
Health System framework	
Eligibility	
Sector size	
User charges	

Institutional environment	
Legal framework	
Material regulation	Standards issued by HAAD defining the principle and procedures governing the recovery of claims payment in health insurance scheme
Prudential regulation	
Private Health Insurance policy framework	
Objective	
Population coverage	
System description	
Coverage type	
Market structure	
Concentration	
Barriers to entry	
Local vs international	
Vertical Integration	
Value Based Health Care	

Reviewer	Husein Reka
Date	20 January 2024
**Document information:**	
Name	HAAD Standard for Recovery of Payment for Healthcare Services under the Health Insurance Scheme Appendix 1
Author(s)	Health Authority of Abu Dhabi
Year of publication	9 Jul 2015
Country/Emirate	Abu Dhabi
Link	https://www.doh.gov.ae/-/media/E8AFBCA8D84C49379BC7E1060638E549.ashx
Aim/purpose	Recovery of payments for claims process flow chart
Record #	151
**Inclusion/Exclusion Criteria**	
Population	Private Health Insurance in Abu Dhabi
Concept	Transformation/Reforms of Abu Dhabi Private Health Insurance Scheme
Context	GCC Health System
**Review framework domains studied in literature on private health insurance transformation**	
Health System Framework (e.g. eligibility, sector size, user charges)	
Institutional environment (e.g. legal framework, regulation)	Payer and provider standards for payments recovery
Private Health Insurance policy framework (e.g. objective, population coverage, system description, types of coverage	
Market structure, conduct and performance (e.g. market concentration, barriers to entry, local vs. international, vertical integration, value-based health care)	
**Key findings related to scoping review question**	
Health System framework	
Eligibility	
Sector size	
User charges	

Institutional environment	
Legal framework	
Material regulation	Clear recovery process flow chart
Prudential regulation	
Private Health Insurance policy framework	
Objective	
Population coverage	
System description	
Coverage type	
Market structure	
Concentration	
Barriers to entry	
Local vs international	
Vertical Integration	
Value Based Health Care	

Reviewer	Husein Reka
Date	20 January 2024
**Document information:**	
Name	HAAD Health Insurance Claims Adjudication Standard
Author(s)	Health Authority of Abu Dhabi
Year of publication	1 Dec 2010
Country/Emirate	Abu Dhabi
Link	https://www.doh.gov.ae/-/media/AE9135D3019B4DDA83240F910D56BC79.ashx
Aim/purpose	Recovery of payments for claims process flow chart
Record #	152
**Inclusion/Exclusion Criteria**	
Population	Private Health Insurance in Abu Dhabi
Concept	Transformation/Reforms of Abu Dhabi Private Health Insurance Scheme
Context	GCC Health System
**Review framework domains studied in literature on private health insurance transformation**	
Health System Framework (e.g. eligibility, sector size, user charges)	
Institutional environment (e.g. legal framework, regulation)	Claims adjudication standards
Private Health Insurance policy framework (e.g. objective, population coverage, system description, types of coverage	
Market structure, conduct and performance (e.g. market concentration, barriers to entry, local vs. international, vertical integration, value-based health care)	
**Key findings related to scoping review question**	
Health System framework	
Eligibility	
Sector size	
User charges	
Institutional environment	

Legal framework	
Material regulation	HAAD issued claims adjudication standards explaining process and rules for health insurance reimbursement
Prudential regulation	
Private Health Insurance policy framework	
Objective	
Population coverage	
System description	
Coverage type	
Market structure	
Concentration	
Barriers to entry	
Local vs international	
Vertical Integration	
Value Based Health Care	

Reviewer	Husein Reka
Date	20 January 2024
**Document information:**	
Name	HAAD Health Insurance Claims Adjudication Standard. Stakeholder letter
Author(s)	Health Authority of Abu Dhabi
Year of publication	12 Dec 2010
Country/Emirate	Abu Dhabi
Link	https://www.doh.gov.ae/-/media/C261D3682ABE4262BE0B7470A57587F5.ashx
Aim/purpose	Communication to stakeholders on adjudication standards
Record #	153
**Inclusion/Exclusion Criteria**	
Population	Private Health Insurance in Abu Dhabi
Concept	Transformation/Reforms of Abu Dhabi Private Health Insurance Scheme
Context	GCC Health System
**Review framework domains studied in literature on private health insurance transformation**	
Health System Framework (e.g. eligibility, sector size, user charges)	
Institutional environment (e.g. legal framework, regulation)	Dear CEO letter
Private Health Insurance policy framework (e.g. objective, population coverage, system description, types of coverage	
Market structure, conduct and performance (e.g. market concentration, barriers to entry, local vs. international, vertical integration, value-based health care)	
**Key findings related to scoping review question**	
Health System framework	
Eligibility	
Sector size	
User charges	

Institutional environment	
Legal framework	
Material regulation	HAAD issued Dear CEO letter to all participants in the health insurance scheme on the standards to ensure consistency efficiency in claims management
Prudential regulation	
Private Health Insurance policy framework	
Objective	
Population coverage	
System description	
Coverage type	
Market structure	
Concentration	
Barriers to entry	
Local vs international	
Vertical Integration	
Value Based Health Care	

Reviewer	Husein Reka
Date	20 January 2024
**Document information:**	
Name	Law No.11 of 2013 on Health Insurance in the Emirate of Dubai
Author(s)	Government of Dubai
Year of publication	24 Nov 2013
Country/Emirate	Dubai
Link	https://www.isahd.ae/content/docs/Health%20Insurance%20Law.pdf
Aim/purpose	Communication to stakeholders on adjudication standards
Record #	158
**Inclusion/Exclusion Criteria**	
Population	Private Health Insurance in Dubai
Concept	Transformation/Reforms of Dubai Private Health Insurance Scheme
Context	GCC Health System
**Review framework domains studied in literature on private health insurance transformation**	
Health System Framework (e.g. eligibility, sector size, user charges)	
Institutional environment (e.g. legal framework, regulation)	Health Insurance Law mandating health insurance in the Emirate of Dubai
Private Health Insurance policy framework (e.g. objective, population coverage, system description, types of coverage	
Market structure, conduct and performance (e.g. market concentration, barriers to entry, local vs. international, vertical integration, value-based health care)	
**Key findings related to scoping review question**	
Health System framework	
Eligibility	
Sector size	

User charges	
Institutional environment	
Legal framework	Government of Dubai promulgated mandatory health insurance law covering all non nationals in a competitive private health insurance scheme called ISAHD (Insurance System for Advancement of Health in Dubai)
Material regulation	
Prudential regulation	
Private Health Insurance policy framework	
Objective	
Population coverage	
System description	
Coverage type	
Market structure	
Concentration	
Barriers to entry	
Local vs international	
Vertical Integration	
Value Based Health Care	

Reviewer	Husein Reka
Date	20 January 2024
**Document information:**	
Name	Fees and fines related to health insurance in Dubai
Author(s)	Government of Dubai
Year of publication	7 Mar 2016
Country/Emirate	Dubai
Link	https://www.isahd.ae/content/docs/ Executive%20Council%20Resolution%20No.%207%20of%202016.pdf
Aim/purpose	Communication to stakeholders on adjudication standards
Record #	159
**Inclusion/Exclusion Criteria**	
Population	Private Health Insurance in Dubai
Concept	Transformation/Reforms of Dubai Private Health Insurance Scheme
Context	GCC Health System
**Review framework domains studied in literature on private health insurance transformation**	
Health System Framework (e.g. eligibility, sector size, user charges)	
Institutional environment (e.g. legal framework, regulation)	Regulating fees and penalties for participating entities
Private Health Insurance policy framework (e.g. objective, population coverage, system description, types of coverage	
Market structure, conduct and performance (e.g. market concentration, barriers to entry, local vs. international, vertical integration, value-based health care)	
**Key findings related to scoping review question**	
Health System framework	
Eligibility	
Sector size	

User charges	
Institutional environment	
Legal framework	Set of fees for entities registered in the scheme and penalties for non-compliance
Material regulation	
Prudential regulation	
Private Health Insurance policy framework	
Objective	
Population coverage	
System description	
Coverage type	
Market structure	
Concentration	
Barriers to entry	
Local vs international	
Vertical Integration	
Value Based Health Care	

Reviewer	Husein Reka
Date	20 January 2024
**Document information:**	
Name	Executive Council decision no.2 of 2016 on financial liability for traffic accident transportation
Author(s)	Government of Dubai
Year of publication	12 Jan 2016
Country/Emirate	Dubai
Link	https://www.isahd.ae/content/docs/Executive%20Council%20Resolution%20No%202%20of%202016.pdf
Aim/purpose	Communication to stakeholders on adjudication standards
Record #	161
**Inclusion/Exclusion Criteria**	
Population	Private Health Insurance in Dubai
Concept	Transformation/Reforms of Dubai Private Health Insurance Scheme
Context	GCC Health System
**Review framework domains studied in literature on private health insurance transformation**	
Health System Framework (e.g. eligibility, sector size, user charges)	
Institutional environment (e.g. legal framework, regulation)	Regulation for traffic accident medical services
Private Health Insurance policy framework (e.g. objective, population coverage, system description, types of coverage	
Market structure, conduct and performance (e.g. market concentration, barriers to entry, local vs. international, vertical integration, value-based health care)	
**Key findings related to scoping review question**	
Health System framework	
Eligibility	

Sector size	
User charges	
Institutional environment	
Legal framework	Dubai Police and Dubai Ambulance Corporation Services to collect a certain amount for providing ambulance and medical services in traffic accident
Material regulation	
Prudential regulation	
Private Health Insurance policy framework	
Objective	
Population coverage	
System description	
Coverage type	
Market structure	
Concentration	
Barriers to entry	
Local vs international	
Vertical Integration	
Value Based Health Care	

Reviewer	Husein Reka
Date	20 January 2024
**Document information:**	
Name	Executive Council Resolution No. 16 of 2013 – Health Insurance for Dubai Government Employees
Author(s)	Government of Dubai
Year of publication	26 Jun 2013
Country/Emirate	Dubai
Link	????
Aim/purpose	Dubai government employee insurance regulation
Record #	163
**Inclusion/Exclusion Criteria**	
Population	Private Health Insurance in Dubai
Concept	Transformation/Reforms of Dubai Private Health Insurance Scheme
Context	GCC Health System
**Review framework domains studied in literature on private health insurance transformation**	
Health System Framework (e.g. eligibility, sector size, user charges)	Regulating the health insurance of dubai government employees
Institutional environment (e.g. legal framework, regulation)	
Private Health Insurance policy framework (e.g. objective, population coverage, system description, types of coverage	
Market structure, conduct and performance (e.g. market concentration, barriers to entry,	

local vs. international, vertical integration, value-based health care)	
**Key findings related to scoping review question**	
Health System framework	
Eligibility	
Sector size	
User charges	
Institutional environment	
Legal framework	Setting regulations on eligibility, coverage and copayment for government employee and their dependents
Material regulation	
Prudential regulation	
Private Health Insurance policy framework	
Objective	
Population coverage	
System description	
Coverage type	
Market structure	
Concentration	
Barriers to entry	
Local vs international	
Vertical Integration	
Value Based Health Care	

Reviewer	Husein Reka
Date	20 January 2024
**Document information:**	
Name	Decree No.17 of 2018 Establishing institutions affiliated with DHA
Author(s)	Government of Dubai
Year of publication	30 Apr 2018
Country/Emirate	Dubai
Link	https://www.isahd.ae/content/docs/61f8d4be-c509-412a-9e1d-9e335fb0a225.pdf
Aim/purpose	Establishment of health insurance regulator
Record #	165
**Inclusion/Exclusion Criteria**	
Population	Private Health Insurance in Dubai
Concept	Transformation/Reforms of Dubai Private Health Insurance Scheme
Context	GCC Health System
**Review framework domains studied in literature on private health insurance transformation**	
Health System Framework (e.g. eligibility, sector size, user charges)	
Institutional environment (e.g. legal framework, regulation)	Decree establishing Dubai Health Insurance Regulator
Private Health Insurance policy framework (e.g. objective, population coverage, system description, types of coverage	
Market structure, conduct and performance (e.g. market concentration, barriers to entry, local vs. international, vertical integration, value-based health care)	
**Key findings related to scoping review question**	
Health System framework	
Eligibility	
Sector size	
User charges	

Institutional environment	
Legal framework	Designated health insurance regulator spun from Dubai Health Authority with mandate and obligations on regulation health insurance in Dubai
Material regulation	
Prudential regulation	
Private Health Insurance policy framework	
Objective	
Population coverage	
System description	
Coverage type	
Market structure	
Concentration	
Barriers to entry	
Local vs international	
Vertical Integration	
Value Based Health Care	

Reviewer	Husein Reka
Date	20 January 2024
**Document information:**	
Name	Decree No.8 of 2022 Implementing health insurance within Dubai Health Care City
Author(s)	Government of Dubai
Year of publication	30 Apr 2022
Country/Emirate	Dubai
Link	https://www.isahd.ae/content/docs/Decree%20No.%208%20%20of%202022.pdf
Aim/purpose	Extending dubai health insurance in Dubai Health Care City
Record #	167
**Inclusion/Exclusion Criteria**	
Population	Private Health Insurance in Dubai
Concept	Transformation/Reforms of Dubai Private Health Insurance Scheme
Context	GCC Health System
**Review framework domains studied in literature on private health insurance transformation**	
Health System Framework (e.g. eligibility, sector size, user charges)	
Institutional environment (e.g. legal framework, regulation)	Extending ISAHD scheme jurisdiction to Dubai Health Care City
Private Health Insurance policy framework (e.g. objective, population coverage, system description, types of coverage	
Market structure, conduct and performance (e.g. market concentration, barriers to entry, local vs. international, vertical integration, value-based health care)	
**Key findings related to scoping review question**	
Health System framework	
Eligibility	

Sector size	
User charges	
Institutional environment	
Legal framework	Dubai Health Authority given full mandate to implement heath insurance in Dubai Health Care City
Material regulation	
Prudential regulation	
Private Health Insurance policy framework	
Objective	
Population coverage	
System description	
Coverage type	
Market structure	
Concentration	
Barriers to entry	
Local vs international	
Vertical Integration	
Value Based Health Care	

Reviewer	Husein Reka
Date	20 January 2024
**Document information:**	
Name	Policy Directive Number 1 of 2014 (PD 01/2014) Complaints handling
Author(s)	Dubai Health Authority
Year of publication	21 Sep 2014
Country/Emirate	Dubai
Link	https://www.isahd.ae/content/docs/PD%2001-2014%20Complaints%20handling%20(rev%201).pdf
Aim/purpose	Improve customer confidence, satisfaction and loyalty in respect of market participants
Record #	168
**Inclusion/Exclusion Criteria**	
Population	Private Health Insurance in Dubai
Concept	Transformation/Reforms of Dubai Private Health Insurance Scheme
Context	GCC Health System
**Review framework domains studied in literature on private health insurance transformation**	
Health System Framework (e.g. eligibility, sector size, user charges)	
Institutional environment (e.g. legal framework, regulation)	Extending ISAHD scheme jurisdiction to Dubai Health Care City
Private Health Insurance policy framework (e.g. objective, population coverage, system description, types of coverage	
Market structure, conduct and performance (e.g. market concentration, barriers to entry, local vs. international, vertical integration, value-based health care)	Guidance and standards to market on handling complaints
**Key findings related to scoping review question**	
Health System framework	
Eligibility	

Sector size	
User charges	
Institutional environment	
Legal framework	
Material regulation	
Prudential regulation	
Private Health Insurance policy framework	
Objective	
Population coverage	
System description	
Coverage type	
Market structure	
Concentration	
Barriers to entry	
Local vs international	
Vertical Integration	
Value Based Health Care	Dubai Health Authority issued requirements to health insurance companies how to handle effectively complaints

Reviewer	Husein Reka
Date	20 January 2024
**Document information:**	
Name	Policy Directive Number 1 of 2014 (PD 01/2014) Complaints handling
Author(s)	Dubai Health Authority
Year of publication	21 Sep 2014
Country/Emirate	Dubai
Link	https://www.isahd.ae/content/docs/PD%2001-2014%20Complaints%20handling%20(rev%201).pdf
Aim/purpose	Improve customer confidence, satisfaction and loyalty in respect of market participants
Record #	168
**Inclusion/Exclusion Criteria**	
Population	Private Health Insurance in Dubai
Concept	Transformation/Reforms of Dubai Private Health Insurance Scheme
Context	GCC Health System
**Review framework domains studied in literature on private health insurance transformation**	
Health System Framework (e.g. eligibility, sector size, user charges)	
Institutional environment (e.g. legal framework, regulation)	
Private Health Insurance policy framework (e.g. objective, population coverage, system description, types of coverage	
Market structure, conduct and performance (e.g. market concentration, barriers to entry, local vs. international, vertical integration, value-based health care)	Guidance and standards to market on handling complaints
**Key findings related to scoping review question**	
Health System framework	
Eligibility	
Sector size	

User charges	
Institutional environment	
Legal framework	
Material regulation	
Prudential regulation	
Private Health Insurance policy framework	
Objective	
Population coverage	
System description	
Coverage type	
Market structure	
Concentration	
Barriers to entry	
Local vs international	
Vertical Integration	
Value Based Health Care	Dubai Health Authority issued requirements to health insurance companies how to handle effectively complaints

Reviewer	Husein Reka
Date	20 January 2024
**Document information:**	
Name	Policy Directive Number 2 of 2014 Compliance with minimum benefits for all health insurance policies
Author(s)	Dubai Health Authority
Year of publication	24 Sep 2014
Country/Emirate	Dubai
Link	https://www.isahd.ae/content/docs/PD%2002-2014%20Compliance%20with%20minimum%20benefits.pdf
Aim/purpose	Compliance notification to market of health insurance policies with the minimum benefits standards
Record #	169
**Inclusion/Exclusion Criteria**	
Population	Private Health Insurance in Dubai
Concept	Transformation/Reforms of Dubai Private Health Insurance Scheme
Context	GCC Health System
**Review framework domains studied in literature on private health insurance transformation**	
Health System Framework (e.g. eligibility, sector size, user charges)	
Institutional environment (e.g. legal framework, regulation)	Regulator notification to insurance companies to comply with new policies on minimum benefit standards
Private Health Insurance policy framework (e.g. objective, population coverage, system description, types of coverage	
Market structure, conduct and performance (e.g. market concentration, barriers to entry, local vs. international, vertical integration, value-based health care)	
**Key findings related to scoping review question**	
Health System framework	
Eligibility	

Sector size	
User charges	
Institutional environment	
Legal framework	
Material regulation	DHA informs the insurance companies on the need of existing and new policies to maintain minimum benefit standards as per the law and deadlines for enrolling eligible categories by June 2016
Prudential regulation	
Private Health Insurance policy framework	
Objective	
Population coverage	
System description	
Coverage type	
Market structure	
Concentration	
Barriers to entry	
Local vs international	
Vertical Integration	
Value Based Health Care	

Reviewer	Husein Reka
Date	20 January 2024
**Document information:**	
Name	Policy Directive Number 03 of 2014 Healthcare Providers' Price Regulation (HPPR)
Author(s)	Dubai Health Authority
Year of publication	10 Dec 2014
Country/Emirate	Dubai
Link	https://www.isahd.ae/content/docs/PD%2003-2014%20Healthcare%20Provider%20Pricing.pdf
Aim/purpose	Communicate to the health sector the components of the healthcare provider price regulation model (HPPR)
Record #	170
**Inclusion/Exclusion Criteria**	
Population	Private Health Insurance in Dubai
Concept	Transformation/Reforms of Dubai Private Health Insurance Scheme
Context	GCC Health System
**Review framework domains studied in literature on private health insurance transformation**	
Health System Framework (e.g. eligibility, sector size, user charges)	
Institutional environment (e.g. legal framework, regulation)	
Private Health Insurance policy framework (e.g. objective, population coverage, system description, types of coverage	
Market structure, conduct and performance (e.g. market concentration, barriers to entry, local vs. international, vertical integration, value-based health care)	Pricing model framework for providers
**Key findings related to scoping review question**	
Health System framework	
Eligibility	
Sector size	

User charges	
Institutional environment	
Legal framework	
Material regulation	
Prudential regulation	
Private Health Insurance policy framework	
Objective	
Population coverage	
System description	
Coverage type	
Market structure	
Concentration	
Barriers to entry	
Local vs international	
Vertical Integration	
Value Based Health Care	DHA mandated a negotiating framework between payers and providers where all healthcare providers in Dubai and registered on eClaimLink must obtain approval from DHA prior to increasing their gross prices. Negotiations must be based on the DHA approved gross price list adjusted annually for inflation

Reviewer	Husein Reka
Date	20 January 2024
**Document information:**	
Name	Policy Directive Number 2 of 2015 Health insurance policies ("top-up policies") providing additional benefits to a main policy
Author(s)	Dubai Health Authority
Year of publication	15 Nov 2015
Country/Emirate	Dubai
Link	https://www.isahd.ae/content/docs/PD%2002-2015%20Top-up%20policies.pdf
Aim/purpose	Additional benefits (top-up) policies
Record #	171
**Inclusion/Exclusion Criteria**	
Population	Private Health Insurance in Dubai
Concept	Transformation/Reforms of Dubai Private Health Insurance Scheme
Context	GCC Health System
**Review framework domains studied in literature on private health insurance transformation**	
Health System Framework (e.g. eligibility, sector size, user charges)	
Institutional environment (e.g. legal framework, regulation)	
Private Health Insurance policy framework (e.g. objective, population coverage, system description, types of coverage	Top-up voluntary health insurance rules
Market structure, conduct and performance (e.g. market concentration, barriers to entry, local vs. international, vertical integration, value-based health care)	
**Key findings related to scoping review question**	
Health System framework	
Eligibility	
Sector size	

User charges	
Institutional environment	
Legal framework	
Material regulation	
Prudential regulation	
Private Health Insurance policy framework	
Objective	
Population coverage	
System description	
Coverage type	DHA clarified that for top-up additional coverage policy that meets or exceeds the minimum requirements of the Essential Benefits Plan, the individual must have in place such a policy
Market structure	
Concentration	
Barriers to entry	
Local vs international	
Vertical Integration	
Value Based Health Care	

Reviewer	Husein Reka
Date	20 January 2024
**Document information:**	
Name	Policy Directive Number 1 of 2016 Insurance coverage for emergency cases and DHA price list
Author(s)	Dubai Health Authority
Year of publication	20 Apr 2016
Country/Emirate	Dubai
Link	Policy Directive Number 1 of 2016 (PD 012016).pdf
Aim/purpose	DHA policy relating to payment for treatment of emergencies
Record #	172
**Inclusion/Exclusion Criteria**	
Population	Private Health Insurance in Dubai
Concept	Transformation/Reforms of Dubai Private Health Insurance Scheme
Context	GCC Health System
**Review framework domains studied in literature on private health insurance transformation**	
Health System Framework (e.g. eligibility, sector size, user charges)	
Institutional environment (e.g. legal framework, regulation)	
Private Health Insurance policy framework (e.g. objective, population coverage, system description, types of coverage	Emergency treatment coverage as part of minimum benefit requirements
Market structure, conduct and performance (e.g. market concentration, barriers to entry, local vs. international, vertical integration, value-based health care)	
**Key findings related to scoping review question**	
Health System framework	
Eligibility	
Sector size	
User charges	

Institutional environment	
Legal framework	
Material regulation	
Prudential regulation	
Private Health Insurance policy framework	
Objective	
Population coverage	
System description	
Coverage type	DHA mandated emergency services to be covered and paid by health insurance companies
Market structure	
Concentration	
Barriers to entry	
Local vs international	
Vertical Integration	
Value Based Health Care	DHA mandated a negotiating framework between payers and providers where all healthcare providers in Dubai and registered on eClaimLink must obtain approval from DHA prior to increasing their gross prices. Negotiations must be based on the DHA approved gross price list adjusted annually for inflation

Reviewer	Husein Reka
Date	20 January 2024
**Document information:**	
Name	Policy Directive Number 01 of 2017 Medical facilities for Hatta population
Author(s)	Dubai Health Authority
Year of publication	12 Feb 2017
Country/Emirate	Dubai
Link	https://www.isahd.ae/content/docs/PD%2001-2017%20Medical%20Facilities%20for%20Hatta%20Population.pdf
Aim/purpose	Requirement for payers to include Hatta Hospital within their medical provider network due to lack of services in the area
Record #	173
**Inclusion/Exclusion Criteria**	
Population	Private Health Insurance in Dubai
Concept	Transformation/Reforms of Dubai Private Health Insurance Scheme
Context	GCC Health System
**Review framework domains studied in literature on private health insurance transformation**	
Health System Framework (e.g. eligibility, sector size, user charges)	
Institutional environment (e.g. legal framework, regulation)	
Private Health Insurance policy framework (e.g. objective, population coverage, system description, types of coverage	Mandatory network coverage for underserved area of Hatta
Market structure, conduct and performance (e.g. market concentration, barriers to entry, local vs. international, vertical integration, value-based health care)	
**Key findings related to scoping review question**	
Health System framework	
Eligibility	

Sector size	
User charges	
Institutional environment	
Legal framework	
Material regulation	
Prudential regulation	
Private Health Insurance policy framework	
Objective	
Population coverage	DHA mandated all insurance companies to include Hatta hospital in their provider network list and the network level
System description	
Coverage type	
Market structure	
Concentration	
Barriers to entry	
Local vs international	
Vertical Integration	
Value Based Health Care	

Reviewer	Husein Reka
Date	20 January 2024
**Document information:**	
Name	Policy Directive Number 3 of 2018 Patient Support Programs – HCV
Author(s)	Dubai Health Authority
Year of publication	29 Jul 2018
Country/Emirate	Dubai
Link	https://www.isahd.ae/content/docs/PD%2003-2018.pdf
Aim/purpose	Clarifications regarding the HCV Elimination program
Record #	177
**Inclusion/Exclusion Criteria**	
Population	Private Health Insurance in Dubai
Concept	Transformation/Reforms of Dubai Private Health Insurance Scheme
Context	GCC Health System
**Review framework domains studied in literature on private health insurance transformation**	
Health System Framework (e.g. eligibility, sector size, user charges)	
Institutional environment (e.g. legal framework, regulation)	
Private Health Insurance policy framework (e.g. objective, population coverage, system description, types of coverage	Implementation of HCV patient support program
Market structure, conduct and performance (e.g. market concentration, barriers to entry, local vs. international, vertical integration, value-based health care)	
**Key findings related to scoping review question**	
Health System framework	
Eligibility	
Sector size	
User charges	
Institutional environment	

Legal framework	
Material regulation	
Prudential regulation	
Private Health Insurance policy framework	
Objective	
Population coverage	
System description	
Coverage type	DHA issued guidance to payers on HCV patient support program
Market structure	
Concentration	
Barriers to entry	
Local vs international	
Vertical Integration	
Value Based Health Care	

Reviewer	Husein Reka
Date	20 January 2024
**Document information:**	
Name	Policy Directive Number 5 of 2018 Updated TOB for the Essential Benefit Plan
Author(s)	Dubai Health Authority
Year of publication	10 Sep 2018
Country/Emirate	Dubai
Link	https://www.isahd.ae/content/docs/PD%2005-2018.pdf
Aim/purpose	Updates on Essential Benefit Plan Table of Benefits
Record #	178
**Inclusion/Exclusion Criteria**	
Population	Private Health Insurance in Dubai
Concept	Transformation/Reforms of Dubai Private Health Insurance Scheme
Context	GCC Health System
**Review framework domains studied in literature on private health insurance transformation**	
Health System Framework (e.g. eligibility, sector size, user charges)	Essential Benefit Plan
Institutional environment (e.g. legal framework, regulation)	
Private Health Insurance policy framework (e.g. objective, population coverage, system description, types of coverage	
Market structure, conduct and performance (e.g. market concentration, barriers to entry, local vs. international, vertical integration, value-based health care)	
**Key findings related to scoping review question**	
Health System framework	
Eligibility	DHA issued updates on EBP TOB clarifying pre-existing conditions, referral procedures and other
Sector size	
User charges	

Institutional environment	
Legal framework	
Material regulation	
Prudential regulation	
Private Health Insurance policy framework	
Objective	
Population coverage	
System description	
Coverage type	
Market structure	
Concentration	
Barriers to entry	
Local vs international	
Vertical Integration	
Value Based Health Care	

Reviewer	Husein Reka
Date	20 January 2024
**Document information:**	
Name	Policy Directive Number 6 of 2018 Basmah Initiative – Fund structure
Author(s)	Dubai Health Authority
Year of publication	10 Sep 2018
Country/Emirate	Dubai
Link	https://www.isahd.ae/content/docs/PD%2006-2018.pdf
Aim/purpose	BASMAH fund format, computation, reconciliation and methods
Record #	179
**Inclusion/Exclusion Criteria**	
Population	Private Health Insurance in Dubai
Concept	Transformation/Reforms of Dubai Private Health Insurance Scheme
Context	GCC Health System
**Review framework domains studied in literature on private health insurance transformation**	
Health System Framework (e.g. eligibility, sector size, user charges)	Clarifications on Basmah fund concept
Institutional environment (e.g. legal framework, regulation)	
Private Health Insurance policy framework (e.g. objective, population coverage, system description, types of coverage	
Market structure, conduct and performance (e.g. market concentration, barriers to entry, local vs. international, vertical integration, value-based health care)	
**Key findings related to scoping review question**	
Health System framework	
Eligibility	DHA issued clarification on fund concept, how to be used, allocation of funds and reconciliation
Sector size	
User charges	

Institutional environment	
Legal framework	
Material regulation	
Prudential regulation	
Private Health Insurance policy framework	
Objective	
Population coverage	
System description	
Coverage type	
Market structure	
Concentration	
Barriers to entry	
Local vs international	
Vertical Integration	
Value Based Health Care	

Reviewer	Husein Reka
Date	20 January 2024
**Document information:**	
Name	Policy Directive Number 7 of 2018 Updated TOB for the Essential Benefit Plan
Author(s)	Dubai Health Authority
Year of publication	17 Dec 2018
Country/Emirate	Dubai
Link	https://www.isahd.ae/content/docs/PD%2007-2018.pdf
Aim/purpose	Updates on Essential Benefit Plan Table of Benefits
Record #	180
**Inclusion/Exclusion Criteria**	
Population	Private Health Insurance in Dubai
Concept	Transformation/Reforms of Dubai Private Health Insurance Scheme
Context	GCC Health System
**Review framework domains studied in literature on private health insurance transformation**	
Health System Framework (e.g. eligibility, sector size, user charges)	Essential Benefit Plan
Institutional environment (e.g. legal framework, regulation)	
Private Health Insurance policy framework (e.g. objective, population coverage, system description, types of coverage	
Market structure, conduct and performance (e.g. market concentration, barriers to entry, local vs. international, vertical integration, value-based health care)	
**Key findings related to scoping review question**	
Health System framework	
Eligibility	DHA included Adult Pneumococcal Conjugate Vaccine
Sector size	
User charges	
Institutional environment	

Legal framework	
Material regulation	
Prudential regulation	
Private Health Insurance policy framework	
Objective	
Population coverage	
System description	
Coverage type	
Market structure	
Concentration	
Barriers to entry	
Local vs international	
Vertical Integration	
Value Based Health Care	

Reviewer	Husein Reka
Date	20 January 2024
**Document information:**	
Name	eClaimLink Policy Directive Number 01 of 2019 Claims Audit and Recovery in the Emirate of Dubai
Author(s)	Dubai Health Authority
Year of publication	19 Mar 2019
Country/Emirate	Dubai
Link	https://www.isahd.ae/content/docs/PD%2001-2019.pdf
Aim/purpose	Directive regulating audit and recovery process
Record #	181
**Inclusion/Exclusion Criteria**	
Population	Private Health Insurance in Dubai
Concept	Transformation/Reforms of Dubai Private Health Insurance Scheme
Context	GCC Health System
**Review framework domains studied in literature on private health insurance transformation**	
Health System Framework (e.g. eligibility, sector size, user charges)	
Institutional environment (e.g. legal framework, regulation)	
Private Health Insurance policy framework (e.g. objective, population coverage, system description, types of coverage	
Market structure, conduct and performance (e.g. market concentration, barriers to entry, local vs. international, vertical integration, value-based health care)	Fraud, waste and abuse
**Key findings related to scoping review question**	
Health System framework	
Eligibility	
Sector size	
User charges	
Institutional environment	

Legal framework	
Material regulation	
Prudential regulation	
Private Health Insurance policy framework	
Objective	
Population coverage	
System description	
Coverage type	
Market structure	
Concentration	
Barriers to entry	
Local vs international	
Vertical Integration	
Value Based Health Care	DHA issued policy directive strengthening claims audit by providing definitions of FWA, audit process and coordination, and reconciliation and recovery of payment process

Reviewer	Husein Reka
Date	20 January 2024
**Document information:**	
Name	Policy Directive Number 03 of 2019 Settlement of Payment for Emergency Services
Author(s)	Dubai Health Authority
Year of publication	23 Apr 2019
Country/Emirate	Dubai
Link	https://www.isahd.ae/content/docs/PD%2003-2019.pdf
Aim/purpose	Emergency services payment settlement and inclusion in basic health insurance (reiteration)
Record #	182
**Inclusion/Exclusion Criteria**	
Population	Private Health Insurance in Dubai
Concept	Transformation/Reforms of Dubai Private Health Insurance Scheme
Context	GCC Health System
**Review framework domains studied in literature on private health insurance transformation**	
Health System Framework (e.g. eligibility, sector size, user charges)	Emergency services coverage under mandatory health insurance
Institutional environment (e.g. legal framework, regulation)	
Private Health Insurance policy framework (e.g. objective, population coverage, system description, types of coverage	
Market structure, conduct and performance (e.g. market concentration, barriers to entry, local vs. international, vertical integration, value-based health care)	
**Key findings related to scoping review question**	
Health System framework	
Eligibility	DHA reminds all parties that emergency services are part of basic package and on timelines of payment for these services

Sector size	
User charges	
Institutional environment	
Legal framework	
Material regulation	
Prudential regulation	
Private Health Insurance policy framework	
Objective	
Population coverage	
System description	
Coverage type	
Market structure	
Concentration	
Barriers to entry	
Local vs international	
Vertical Integration	
Value Based Health Care	

Reviewer	Husein Reka
Date	20 January 2024
**Document information:**	
Name	Policy Directive Number 04 of 2020 Co-Payments of emergency cases transferred by ambulance
Author(s)	Dubai Health Authority
Year of publication	19 Mar 2020
Country/Emirate	Dubai
Link	https://www.isahd.ae/content/docs/PD%2004-2020.pdf
Aim/purpose	Co-payments regime for emergency cases transfer
Record #	185
**Inclusion/Exclusion Criteria**	
Population	Private Health Insurance in Dubai
Concept	Transformation/Reforms of Dubai Private Health Insurance Scheme
Context	GCC Health System
**Review framework domains studied in literature on private health insurance transformation**	
Health System Framework (e.g. eligibility, sector size, user charges)	Copayment regime for emergency services transport
Institutional environment (e.g. legal framework, regulation)	
Private Health Insurance policy framework (e.g. objective, population coverage, system description, types of coverage	
Market structure, conduct and performance (e.g. market concentration, barriers to entry, local vs. international, vertical integration, value-based health care)	
**Key findings related to scoping review question**	
Health System framework	
Eligibility	
Sector size	

User charges	DHA clarifies that no co-payment should be charged for emergency patients transportation that are insured via Dubai Corporation for Ambulance Services
Institutional environment	
Legal framework	
Material regulation	
Prudential regulation	
Private Health Insurance policy framework	
Objective	
Population coverage	
System description	
Coverage type	
Market structure	
Concentration	
Barriers to entry	
Local vs international	
Vertical Integration	
Value Based Health Care	

Reviewer	Husein Reka
Date	20 January 2024
**Document information:**	
Name	Compliance with Essential Benefits Plan
Author(s)	Dubai Health Authority
Year of publication	24 Nov 2020
Country/Emirate	Dubai
Link	https://www.isahd.ae/content/docs/PD%20Compliance%20with%20Essential%20Benefits%20Plan%20updates%20and%20top-up%20policies_24-11-2020.pd
Aim/purpose	Market guidance on compliance with EBP
Record #	189
**Inclusion/Exclusion Criteria**	
Population	Private Health Insurance in Dubai
Concept	Transformation/Reforms of Dubai Private Health Insurance Scheme
Context	GCC Health System
**Review framework domains studied in literature on private health insurance transformation**	
Health System Framework (e.g. eligibility, sector size, user charges)	Essential Benefit Package compliance
Institutional environment (e.g. legal framework, regulation)	
Private Health Insurance policy framework (e.g. objective, population coverage, system description, types of coverage	
Market structure, conduct and performance (e.g. market concentration, barriers to entry, local vs. international, vertical integration, value-based health care)	
**Key findings related to scoping review question**	
Health System framework	
Eligibility	DHA issued notifications on the dates of phases of mandatory health insurance launch and compliance with new EBP TOB as following:

	a) For employers with employees numbering 1000 or more: 31 October 2014<br>b) For employers with 100-999 employees: 31 July 2015<br>c) For employers with less than 100 employees: 30 June 2016<br>d) For spouses, dependents and domestic workers: 30 June 2016
Sector size	
User charges	
Institutional environment	
Legal framework	
Material regulation	
Prudential regulation	
Private Health Insurance policy framework	
Objective	
Population coverage	
System description	
Coverage type	
Market structure	
Concentration	
Barriers to entry	
Local vs international	
Vertical Integration	
Value Based Health Care	

Reviewer	Husein Reka
Date	20 January 2024
**Document information:**	
Name	Complaints Handling
Author(s)	Dubai Health Authority
Year of publication	24 Nov 2020
Country/Emirate	Dubai
Link	https://www.isahd.ae/content/docs/PD%20Complaints%20 Handling_24-11-2020.pdf
Aim/purpose	Minimum requirements and standards for complaints
Record #	190
**Inclusion/Exclusion Criteria**	
Population	Private Health Insurance in Dubai
Concept	Transformation/Reforms of Dubai Private Health Insurance Scheme
Context	GCC Health System
**Review framework domains studied in literature on private health insurance transformation**	
Health System Framework (e.g. eligibility, sector size, user charges)	
Institutional environment (e.g. legal framework, regulation)	
Private Health Insurance policy framework (e.g. objective, population coverage, system description, types of coverage	
Market structure, conduct and performance (e.g. market concentration, barriers to entry, local vs. international, vertical integration, value-based health care)	Customer satisfaction improvement
**Key findings related to scoping review question**	
Health System framework	
Eligibility	
Sector size	
User charges	
Institutional environment	

Legal framework	
Material regulation	
Prudential regulation	
Private Health Insurance policy framework	
Objective	
Population coverage	
System description	
Coverage type	
Market structure	
Concentration	
Barriers to entry	
Local vs international	
Vertical Integration	
Value Based Health Care	DHA issued detailed guidance to market on complains handling process and KPIs

Reviewer	Husein Reka
Date	20 January 2024
**Document information:**	
Name	Claims Audit and Recovery
Author(s)	Dubai Health Authority
Year of publication	24 Nov 2020
Country/Emirate	Dubai
Link	https://www.isahd.ae/content/docs/PD%20Claims%20Audit%20and%20Recovery_24-11-2020.pdf
Aim/purpose	Overview of the Recovery and Claims Audit practices, standards and procedures
Record #	191
**Inclusion/Exclusion Criteria**	
Population	Private Health Insurance in Dubai
Concept	Transformation/Reforms of Dubai Private Health Insurance Scheme
Context	GCC Health System
**Review framework domains studied in literature on private health insurance transformation**	
Health System Framework (e.g. eligibility, sector size, user charges)	
Institutional environment (e.g. legal framework, regulation)	
Private Health Insurance policy framework (e.g. objective, population coverage, system description, types of coverage	
Market structure, conduct and performance (e.g. market concentration, barriers to entry, local vs. international, vertical integration, value-based health care)	Fraud, waste and abuse
**Key findings related to scoping review question**	
Health System framework	
Eligibility	
Sector size	
User charges	

Institutional environment	
Legal framework	
Material regulation	
Prudential regulation	
Private Health Insurance policy framework	
Objective	
Population coverage	
System description	
Coverage type	
Market structure	
Concentration	
Barriers to entry	
Local vs international	
Vertical Integration	
Value Based Health Care	DHA issued detailed policy directive on claims audit and recovery practices, standards and procedures

Reviewer	Husein Reka
Date	20 January 2024
**Document information:**	
Name	Patient Support Programs
Author(s)	Dubai Health Authority
Year of publication	24 Nov 2020
Country/Emirate	Dubai
Link	https://www.isahd.ae/content/docs/PD%20Patient%20Support%20Programs_24-11-2020.pdf
Aim/purpose	Updates on Basmah Patient Support Program
Record #	192
**Inclusion/Exclusion Criteria**	
Population	Private Health Insurance in Dubai
Concept	Transformation/Reforms of Dubai Private Health Insurance Scheme
Context	GCC Health System
**Review framework domains studied in literature on private health insurance transformation**	
Health System Framework (e.g. eligibility, sector size, user charges)	Single pool fund for cancer treatment
Institutional environment (e.g. legal framework, regulation)	
Private Health Insurance policy framework (e.g. objective, population coverage, system description, types of coverage	
Market structure, conduct and performance (e.g. market concentration, barriers to entry, local vs. international, vertical integration, value-based health care)	
**Key findings related to scoping review question**	
Health System framework	
Eligibility	DHA updates:<br>1st of July 2018, the allocated fund is AED 9.5 /- under the fund budget for 2018 and AED 9.5 /- under fund budget for 2019.

	A dedicated BASMAH fund account has been created for HIPs & PIs to transfer the allocated AED 19/-, therefore there will be no reconciliation required or additional payments accrued by the HIP or PI should an insured and enrolled member exceed their annual limit. Any additional treatment costs will be borne by the Fund.
Sector size	
User charges	
Institutional environment	
Legal framework	
Material regulation	
Prudential regulation	
Private Health Insurance policy framework	
Objective	
Population coverage	
System description	
Coverage type	
Market structure	
Concentration	
Barriers to entry	
Local vs international	
Vertical Integration	
Value Based Health Care	

Reviewer	Husein Reka
Date	20 January 2024
**Document information:**	
Name	Cancer Patient Support Program (BASMAH)
Author(s)	Dubai Health Authority
Year of publication	24 Nov 2020
Country/Emirate	Dubai
Link	https://www.isahd.ae/content/docs/PD%20PSP%20Program_Basmah%20Guidelines_24-11-2020.pdf
Aim/purpose	Terms and conditions for enrolment in Basmah (cancer)
Record #	193
**Inclusion/Exclusion Criteria**	
Population	Private Health Insurance in Dubai
Concept	Transformation/Reforms of Dubai Private Health Insurance Scheme
Context	GCC Health System
**Review framework domains studied in literature on private health insurance transformation**	
Health System Framework (e.g. eligibility, sector size, user charges)	Eligibility and enrolment procedures for cancer patient support program
Institutional environment (e.g. legal framework, regulation)	
Private Health Insurance policy framework (e.g. objective, population coverage, system description, types of coverage	
Market structure, conduct and performance (e.g. market concentration, barriers to entry, local vs. international, vertical integration, value-based health care)	
**Key findings related to scoping review question**	
Health System framework	
Eligibility	DHA issue detailed terms and conditions on eligibility and enrolment with Basmah cancer support program, referral process, funds utilization, and other
Sector size	

User charges	
Institutional environment	
Legal framework	
Material regulation	
Prudential regulation	
Private Health Insurance policy framework	
Objective	
Population coverage	
System description	
Coverage type	
Market structure	
Concentration	
Barriers to entry	
Local vs international	
Vertical Integration	
Value Based Health Care	

Reviewer	Husein Reka
Date	20 January 2024
**Document information:**	
Name	Hepatitis C Patient Support Program
Author(s)	Dubai Health Authority
Year of publication	24 Nov 2020
Country/Emirate	Dubai
Link	https://www.isahd.ae/content/docs/PD%20PSP%20Program_Hepatitis%20C%20Guidelines_24-11-2020.pdf
Aim/purpose	Terms and conditions for enrolment in Basmah (Hep C)
Record #	195
**Inclusion/Exclusion Criteria**	
Population	Private Health Insurance in Dubai
Concept	Transformation/Reforms of Dubai Private Health Insurance Scheme
Context	GCC Health System
**Review framework domains studied in literature on private health insurance transformation**	
Health System Framework (e.g. eligibility, sector size, user charges)	Eligibility and enrolment procedures for Hepatitis C patient support program
Institutional environment (e.g. legal framework, regulation)	
Private Health Insurance policy framework (e.g. objective, population coverage, system description, types of coverage	
Market structure, conduct and performance (e.g. market concentration, barriers to entry, local vs. international, vertical integration, value-based health care)	
**Key findings related to scoping review question**	
Health System framework	
Eligibility	DHA issue detailed terms and conditions on eligibility and enrolment with Basmah Hepatitis C support program, referral process, funds utilization, and other
Sector size	

User charges	
Institutional environment	
Legal framework	
Material regulation	
Prudential regulation	
Private Health Insurance policy framework	
Objective	
Population coverage	
System description	
Coverage type	
Market structure	
Concentration	
Barriers to entry	
Local vs international	
Vertical Integration	
Value Based Health Care	

Reviewer	Husein Reka
Date	20 January 2024
**Document information:**	
Name	Insurance Coverage of Emergency Cases
Author(s)	Dubai Health Authority
Year of publication	24 Nov 2020
Country/Emirate	Dubai
Link	https://www.isahd.ae/content/docs/PD%20Insurance%20coverage%20of%20Emergency%20cases_24-11-2020.pdf
Aim/purpose	Mandate and standards on emergency services under health insurance
Record #	197
**Inclusion/Exclusion Criteria**	
Population	Private Health Insurance in Dubai
Concept	Transformation/Reforms of Dubai Private Health Insurance Scheme
Context	GCC Health System
**Review framework domains studied in literature on private health insurance transformation**	
Health System Framework (e.g. eligibility, sector size, user charges)	Emergency services definition eligibility and coverage
Institutional environment (e.g. legal framework, regulation)	
Private Health Insurance policy framework (e.g. objective, population coverage, system description, types of coverage	
Market structure, conduct and performance (e.g. market concentration, barriers to entry, local vs. international, vertical integration, value-based health care)	
**Key findings related to scoping review question**	
Health System framework	
Eligibility	DHA market guidance on the legal requirement of a minimum treatment costs necessary to stabilize the

	patient's condition and prior to a transfer to a network facility must be covered under the insurance policy
Sector size	
User charges	
Institutional environment	
Legal framework	
Material regulation	
Prudential regulation	
Private Health Insurance policy framework	
Objective	
Population coverage	
System description	
Coverage type	
Market structure	
Concentration	
Barriers to entry	
Local vs international	
Vertical Integration	
Value Based Health Care	

Reviewer	Husein Reka
Date	20 January 2024
**Document information:**	
Name	Healthcare Providers' Price Regulation
Author(s)	Dubai Health Authority
Year of publication	24 Nov 2020
Country/Emirate	Dubai
Link	https://www.isahd.ae/content/docs/PD%20Healthcare%20Providers'%20Price%20Regulation_24-11-2020.pdf
Aim/purpose	Healthcare provider price regulation model for 2015
Record #	198
**Inclusion/Exclusion Criteria**	
Population	Private Health Insurance in Dubai
Concept	Transformation/Reforms of Dubai Private Health Insurance Scheme
Context	GCC Health System
**Review framework domains studied in literature on private health insurance transformation**	
Health System Framework (e.g. eligibility, sector size, user charges)	Emergency services definition eligibility and coverage
Institutional environment (e.g. legal framework, regulation)	
Private Health Insurance policy framework (e.g. objective, population coverage, system description, types of coverage	
Market structure, conduct and performance (e.g. market concentration, barriers to entry, local vs. international, vertical integration, value-based health care)	Service pricing regulation model
**Key findings related to scoping review question**	
Health System framework	
Eligibility	
Sector size	
User charges	
Institutional environment	

Legal framework	
Material regulation	
Prudential regulation	
Private Health Insurance policy framework	
Objective	
Population coverage	
System description	
Coverage type	
Market structure	
Concentration	
Barriers to entry	
Local vs international	
Vertical Integration	
Value Based Health Care	DHA issued policy directive on the Health Care Provider Price Regulation (HPPR) model for 2015

Reviewer	Husein Reka
Date	20 January 2024
**Document information:**	
Name	Updated TOB for the Essential Benefit Plan
Author(s)	Dubai Health Authority
Year of publication	21 Nov 2021
Country/Emirate	Dubai
Link	https://www.isahd.ae/content/docs/PD%2001-2021%20Updated%20TOB%20for%20Essential%20Benefit%20Plan.pdf
Aim/purpose	Healthcare provider price regulation model for 2015
Record #	199
**Inclusion/Exclusion Criteria**	
Population	Private Health Insurance in Dubai
Concept	Transformation/Reforms of Dubai Private Health Insurance Scheme
Context	GCC Health System
**Review framework domains studied in literature on private health insurance transformation**	
Health System Framework (e.g. eligibility, sector size, user charges)	Updates on Essential Benefit Package Terms of Benefits
Institutional environment (e.g. legal framework, regulation)	
Private Health Insurance policy framework (e.g. objective, population coverage, system description, types of coverage	
Market structure, conduct and performance (e.g. market concentration, barriers to entry, local vs. international, vertical integration, value-based health care)	
**Key findings related to scoping review question**	
Health System framework	
Eligibility	DHA updated EBP TOB on pre-existing conditions, maternity services, and referral procedures for inpatient and outpatient

Sector size	
User charges	
Institutional environment	
Legal framework	
Material regulation	
Prudential regulation	
Private Health Insurance policy framework	
Objective	
Population coverage	
System description	
Coverage type	
Market structure	
Concentration	
Barriers to entry	
Local vs international	
Vertical Integration	
Value Based Health Care	DHA issued policy directive on the Health Care Provider Price Regulation (HPPR) model for 2015

Reviewer	Husein Reka
Date	20 January 2024
**Document information:**	
Name	Maternity and newborn coverage policy
Author(s)	Dubai Health Authority
Year of publication	29 Jul 2022
Country/Emirate	Dubai
Link	https://www.isahd.ae/content/docs/PD%2001-2023%20Maternity%20and%20New%20Born%20Coverage%20Policy.pdf
Aim/purpose	Healthcare provider price regulation model for 2015
Record #	200
**Inclusion/Exclusion Criteria**	
Population	Private Health Insurance in Dubai
Concept	Transformation/Reforms of Dubai Private Health Insurance Scheme
Context	GCC Health System
**Review framework domains studied in literature on private health insurance transformation**	
Health System Framework (e.g. eligibility, sector size, user charges)	Updates on Essential Benefit Package for maternity and ante natal care
Institutional environment (e.g. legal framework, regulation)	
Private Health Insurance policy framework (e.g. objective, population coverage, system description, types of coverage	
Market structure, conduct and performance (e.g. market concentration, barriers to entry, local vs. international, vertical integration, value-based health care)	
**Key findings related to scoping review question**	
Health System framework	
Eligibility	DHA mandates and specifies circumstances under which maternity and ante natal care services are covered as part of EBP

Sector size	
User charges	
Institutional environment	
Legal framework	
Material regulation	
Prudential regulation	
Private Health Insurance policy framework	
Objective	
Population coverage	
System description	
Coverage type	
Market structure	
Concentration	
Barriers to entry	
Local vs international	
Vertical Integration	
Value Based Health Care	

Reviewer	Husein Reka
Date	20 January 2024
**Document information:**	
Name	Circular Number 2 of 2014 Compliance regarding self-funded schemes, compliance with minimum benefits levels and access to Lower Salary Band (LSB) employees
Author(s)	Dubai Health Authority
Year of publication	4 Sep 2014
Country/Emirate	Dubai
Link	https://www.isahd.ae/content/docs/GC%2002-2014%20Self-funded%20and%20EBP%20compliance.pdf
Aim/purpose	Reiteration to market on minimum benefits of new scheme
Record #	202
**Inclusion/Exclusion Criteria**	
Population	Private Health Insurance in Dubai
Concept	Transformation/Reforms of Dubai Private Health Insurance Scheme
Context	GCC Health System
**Review framework domains studied in literature on private health insurance transformation**	
Health System Framework (e.g. eligibility, sector size, user charges)	Minimum requirements for EBP
Institutional environment (e.g. legal framework, regulation)	
Private Health Insurance policy framework (e.g. objective, population coverage, system description, types of coverage	
Market structure, conduct and performance (e.g. market concentration, barriers to entry, local vs. international, vertical integration, value-based health care)	
**Key findings related to scoping review question**	
Health System framework	
Eligibility	DHA reiterates guidance to market on several points:

	- No new self-administered self-funded schemes allowed<br>- Rules on sales of policies for LSB policies<br>- Marketing and conduct of business compliance with new Health Insurance Law
Sector size	
User charges	
Institutional environment	
Legal framework	
Material regulation	
Prudential regulation	
Private Health Insurance policy framework	
Objective	
Population coverage	
System description	
Coverage type	
Market structure	
Concentration	
Barriers to entry	
Local vs international	
Vertical Integration	
Value Based Health Care	

Reviewer	Husein Reka
Date	20 January 2024
**Document information:**	
Name	Circular Number 4 of 2014 Activities of Health Insurance Claims Management Companies
Author(s)	Dubai Health Authority
Year of publication	2 Nov 2014
Country/Emirate	Dubai
Link	https://www.isahd.ae/content/docs/GC%2004-2014%20TPA%20activities.pdf
Aim/purpose	Reminder to TPAs on their scope of activities
Record #	204
**Inclusion/Exclusion Criteria**	
Population	Private Health Insurance in Dubai
Concept	Transformation/Reforms of Dubai Private Health Insurance Scheme
Context	GCC Health System
**Review framework domains studied in literature on private health insurance transformation**	
Health System Framework (e.g. eligibility, sector size, user charges)	Minimum requirements for EBP
Institutional environment (e.g. legal framework, regulation)	
Private Health Insurance policy framework (e.g. objective, population coverage, system description, types of coverage	
Market structure, conduct and performance (e.g. market concentration, barriers to entry, local vs. international, vertical integration, value-based health care)	Reminder to TPAs on conduct of business
**Key findings related to scoping review question**	
Health System framework	
Eligibility	
Sector size	
User charges	

Institutional environment	
Legal framework	
Material regulation	
Prudential regulation	
Private Health Insurance policy framework	
Objective	
Population coverage	
System description	
Coverage type	
Market structure	
Concentration	
Barriers to entry	
Local vs international	
Vertical Integration	DHA reminded TPAs on their scope of activities and the prohibition of marketing and sales activities, non-compliance of which may lead to suspension or withdrawal of license
Value Based Health Care	

Reviewer	Husein Reka
Date	20 January 2024
**Document information:**	
Name	Circular Number 5 of 2014 DHA approach to enforcement of fines and penalties
Author(s)	Dubai Health Authority
Year of publication	18 Nov 2014
Country/Emirate	Dubai
Link	https://www.isahd.ae/content/docs/GC%2005-2014%20Enforcement%20of%20fines%20and%20penalties.pdf
Aim/purpose	Market guidance on implementation of fines and penalties
Record #	205
**Inclusion/Exclusion Criteria**	
Population	Private Health Insurance in Dubai
Concept	Transformation/Reforms of Dubai Private Health Insurance Scheme
Context	GCC Health System
**Review framework domains studied in literature on private health insurance transformation**	
Health System Framework (e.g. eligibility, sector size, user charges)	
Institutional environment (e.g. legal framework, regulation)	Fines and penalties by regulator
Private Health Insurance policy framework (e.g. objective, population coverage, system description, types of coverage	
Market structure, conduct and performance (e.g. market concentration, barriers to entry, local vs. international, vertical integration, value-based health care)	
**Key findings related to scoping review question**	
Health System framework	
Eligibility	
Sector size	

User charges	
Institutional environment	
Legal framework	
Material regulation	DHA clarifies that fines and penalties to be released soon as part of Law no. 11 of 2013 Executive Regulations will apply retrospectively for any breach of law after the law came in force (14 Feb 2014)
Prudential regulation	
Private Health Insurance policy framework	
Objective	
Population coverage	
System description	
Coverage type	
Market structure	
Concentration	
Barriers to entry	
Local vs international	
Vertical Integration	
Value Based Health Care	

Reviewer	Husein Reka
Date	20 January 2024
**Document information:**	
Name	Circular Number 5 of 2015 HIP applications 2015
Author(s)	Dubai Health Authority
Year of publication	16 Jan 2015
Country/Emirate	Dubai
Link	https://www.isahd.ae/content/docs/GC%2005-2015%20HIP%20status%20results%202015.pdf
Aim/purpose	Updates to compliance status of HIP applicants for 2015
Record #	207
**Inclusion/Exclusion Criteria**	
Population	Private Health Insurance in Dubai
Concept	Transformation/Reforms of Dubai Private Health Insurance Scheme
Context	GCC Health System
**Review framework domains studied in literature on private health insurance transformation**	
Health System Framework (e.g. eligibility, sector size, user charges)	
Institutional environment (e.g. legal framework, regulation)	Qualification to participate in ISAHD scheme
Private Health Insurance policy framework (e.g. objective, population coverage, system description, types of coverage	
Market structure, conduct and performance (e.g. market concentration, barriers to entry, local vs. international, vertical integration, value-based health care)	
**Key findings related to scoping review question**	
Health System framework	
Eligibility	
Sector size	
User charges	
Institutional environment	

Legal framework	
Material regulation	DHA published an updated list of insurance companies compliant (conditional and unconditional) with Dubai Health Insurance Permit
Prudential regulation	
Private Health Insurance policy framework	
Objective	
Population coverage	
System description	
Coverage type	
Market structure	
Concentration	
Barriers to entry	
Local vs international	
Vertical Integration	
Value Based Health Care	

Reviewer	Husein Reka
Date	20 January 2024
**Document information:**	
Name	Circular Number 10 of 2015 Participating Insurer (PI) applications and renewals for 2016
Author(s)	Dubai Health Authority
Year of publication	15 Dec 2015
Country/Emirate	Dubai
Link	https://www.isahd.ae/content/docs/GC%2010-2015%20PI%20applications%20and%20renewals%20for%202016.pdf
Aim/purpose	DHA decision on Participating Insurer status for 2016
Record #	209
**Inclusion/Exclusion Criteria**	
Population	Private Health Insurance in Dubai
Concept	Transformation/Reforms of Dubai Private Health Insurance Scheme
Context	GCC Health System
**Review framework domains studied in literature on private health insurance transformation**	
Health System Framework (e.g. eligibility, sector size, user charges)	
Institutional environment (e.g. legal framework, regulation)	Qualification to participate in ISAHD scheme
Private Health Insurance policy framework (e.g. objective, population coverage, system description, types of coverage	
Market structure, conduct and performance (e.g. market concentration, barriers to entry, local vs. international, vertical integration, value-based health care)	
**Key findings related to scoping review question**	
Health System framework	
Eligibility	
Sector size	

User charges	
Institutional environment	
Legal framework	
Material regulation	DHA informed that no new applications for PI for 2016 will be opened and that the requirement for existing PI to renew will be announced within two weeks
Prudential regulation	
Private Health Insurance policy framework	
Objective	
Population coverage	
System description	
Coverage type	
Market structure	
Concentration	
Barriers to entry	
Local vs international	
Vertical Integration	
Value Based Health Care	

Reviewer	Husein Reka
Date	20 January 2024
**Document information:**	
Name	Circular Number 1 of 2016 Registration and regulation of health insurance intermediaries
Author(s)	Dubai Health Authority
Year of publication	4 Jan 2016
Country/Emirate	Dubai
Link	https://www.isahd.ae/content/docs/GC%2001-2016%20HIIP%20status%20of%20intermediaries.pdf
Aim/purpose	Unconditional compliance status award for intermediares
Record #	210
**Inclusion/Exclusion Criteria**	
Population	Private Health Insurance in Dubai
Concept	Transformation/Reforms of Dubai Private Health Insurance Scheme
Context	GCC Health System
**Review framework domains studied in literature on private health insurance transformation**	
Health System Framework (e.g. eligibility, sector size, user charges)	
Institutional environment (e.g. legal framework, regulation)	Qualification to participate in ISAHD scheme
Private Health Insurance policy framework (e.g. objective, population coverage, system description, types of coverage	
Market structure, conduct and performance (e.g. market concentration, barriers to entry, local vs. international, vertical integration, value-based health care)	
**Key findings related to scoping review question**	
Health System framework	
Eligibility	
Sector size	
User charges	

Institutional environment	
Legal framework	
Material regulation	DHA issued a list of approved health insurance intermediaries
Prudential regulation	
Private Health Insurance policy framework	
Objective	
Population coverage	
System description	
Coverage type	
Market structure	
Concentration	
Barriers to entry	
Local vs international	
Vertical Integration	
Value Based Health Care	

Reviewer	Husein Reka
Date	20 January 2024
**Document information:**	
Name	Circular Number 2 of 2016 (special needs members)
Author(s)	Dubai Health Authority
Year of publication	19 Jun 2016
Country/Emirate	Dubai
Link	https://www.isahd.ae/content/docs/GC%2002-2016%20Special%20Needs%20Members.pdf
Aim/purpose	Minimum benefits standards and market practice for members with special needs
Record #	212
**Inclusion/Exclusion Criteria**	
Population	Private Health Insurance in Dubai
Concept	Transformation/Reforms of Dubai Private Health Insurance Scheme
Context	GCC Health System
**Review framework domains studied in literature on private health insurance transformation**	
Health System Framework (e.g. eligibility, sector size, user charges)	
Institutional environment (e.g. legal framework, regulation)	
Private Health Insurance policy framework (e.g. objective, population coverage, system description, types of coverage	
Market structure, conduct and performance (e.g. market concentration, barriers to entry, local vs. international, vertical integration, value-based health care)	Conduct of business
**Key findings related to scoping review question**	
Health System framework	
Eligibility	
Sector size	
User charges	

Institutional environment	
Legal framework	
Material regulation	
Prudential regulation	
Private Health Insurance policy framework	
Objective	
Population coverage	
System description	
Coverage type	
Market structure	
Concentration	
Barriers to entry	DHA reminds insurance companies that individuals with special needs are eligible for health insurance and are not permitted to decline quoting for them
Local vs international	
Vertical Integration	
Value Based Health Care	

Reviewer	Husein Reka
Date	20 January 2024
**Document information:**	
Name	Circular Number 3 of 2016 (marketing/advertising medical insurance)
Author(s)	Dubai Health Authority
Year of publication	19 Jun 2016
Country/Emirate	Dubai
Link	https://www.isahd.ae/content/docs/GC%2003-2016%20Marketing%20or%20Advertising%20Medical%20Insurance.pdf
Aim/purpose	Information on marketing or advertising requirements
Record #	213
**Inclusion/Exclusion Criteria**	
Population	Private Health Insurance in Dubai
Concept	Transformation/Reforms of Dubai Private Health Insurance Scheme
Context	GCC Health System
**Review framework domains studied in literature on private health insurance transformation**	
Health System Framework (e.g. eligibility, sector size, user charges)	
Institutional environment (e.g. legal framework, regulation)	
Private Health Insurance policy framework (e.g. objective, population coverage, system description, types of coverage	
Market structure, conduct and performance (e.g. market concentration, barriers to entry, local vs. international, vertical integration, value-based health care)	Conduct of business
**Key findings related to scoping review question**	
Health System framework	
Eligibility	
Sector size	

User charges	
Institutional environment	
Legal framework	
Material regulation	
Prudential regulation	
Private Health Insurance policy framework	
Objective	
Population coverage	
System description	
Coverage type	
Market structure	
Concentration	
Barriers to entry	
Local vs international	
Vertical Integration	
Value Based Health Care	DHA mandates that all marketing material in regards to Mandatory Health Insurance must be approved by HFD of DHA prior to circulation. This applies to all forms of media and is applicable to any parties involved in marketing medical insurance

Reviewer	Husein Reka
Date	20 January 2024
**Document information:**	
Name	Circular Number 4 of 2016 Mandatory deadline for all citizens and residents of Dubai to be insured
Author(s)	Dubai Health Authority
Year of publication	23 Jun 2016
Country/Emirate	Dubai
Link	https://www.isahd.ae/content/docs/GC%2004-2016%20Mandatory%20Insurance%20Deadline.pdf
Aim/purpose	Clarification on the deadline date for mandatory health insurance in the Emirate of Dubai
Record #	214
**Inclusion/Exclusion Criteria**	
Population	Private Health Insurance in Dubai
Concept	Transformation/Reforms of Dubai Private Health Insurance Scheme
Context	GCC Health System
**Review framework domains studied in literature on private health insurance transformation**	
Health System Framework (e.g. eligibility, sector size, user charges)	
Institutional environment (e.g. legal framework, regulation)	Mandatory Health Insurance deadline
Private Health Insurance policy framework (e.g. objective, population coverage, system description, types of coverage	
Market structure, conduct and performance (e.g. market concentration, barriers to entry, local vs. international, vertical integration, value-based health care)	
**Key findings related to scoping review question**	
Health System framework	
Eligibility	
Sector size	

User charges	
Institutional environment	
Legal framework	DHA reiterates on the deadline for mandatory health insurance mandatory deadline still remains as June 30th, 2016 and provides information on scheme details
Material regulation	
Prudential regulation	
Private Health Insurance policy framework	
Objective	
Population coverage	
System description	
Coverage type	
Market structure	
Concentration	
Barriers to entry	
Local vs international	
Vertical Integration	
Value Based Health Care	

Reviewer	Husein Reka
Date	20 January 2024
**Document information:**	
Name	Circular Number 5 of 2016 Registration and regulation of health insurance intermediaries
Author(s)	Dubai Health Authority
Year of publication	23 Aug 2016
Country/Emirate	Dubai
Link	https://www.isahd.ae/content/docs/GC%2005-2016%20HIIP%20status%20of%20intermediaries.pdf
Aim/purpose	Notification of intermediaries with “Unconditional Compliance” status
Record #	215
**Inclusion/Exclusion Criteria**	
Population	Private Health Insurance in Dubai
Concept	Transformation/Reforms of Dubai Private Health Insurance Scheme
Context	GCC Health System
**Review framework domains studied in literature on private health insurance transformation**	
Health System Framework (e.g. eligibility, sector size, user charges)	
Institutional environment (e.g. legal framework, regulation)	Brokers regulation
Private Health Insurance policy framework (e.g. objective, population coverage, system description, types of coverage	
Market structure, conduct and performance (e.g. market concentration, barriers to entry, local vs. international, vertical integration, value-based health care)	
**Key findings related to scoping review question**	
Health System framework	
Eligibility	
Sector size	

User charges	
Institutional environment	
Legal framework	
Material regulation	DHA issued a list of intermediaries awarded the “Unconditional Compliance” status
Prudential regulation	
Private Health Insurance policy framework	
Objective	
Population coverage	
System description	
Coverage type	
Market structure	
Concentration	
Barriers to entry	
Local vs international	
Vertical Integration	
Value Based Health Care	

Reviewer	Husein Reka
Date	20 January 2024
**Document information:**	
Name	Circular Number 6 of 2016 Member Register
Author(s)	Dubai Health Authority
Year of publication	25 Aug 2016
Country/Emirate	Dubai
Link	https://www.isahd.ae/content/docs/GC%2006-2016%20Member%20Register.pdf
Aim/purpose	Importance of the member register
Record #	216
**Inclusion/Exclusion Criteria**	
Population	Private Health Insurance in Dubai
Concept	Transformation/Reforms of Dubai Private Health Insurance Scheme
Context	GCC Health System
**Review framework domains studied in literature on private health insurance transformation**	
Health System Framework (e.g. eligibility, sector size, user charges)	Member registration
Institutional environment (e.g. legal framework, regulation)	
Private Health Insurance policy framework (e.g. objective, population coverage, system description, types of coverage	
Market structure, conduct and performance (e.g. market concentration, barriers to entry, local vs. international, vertical integration, value-based health care)	
**Key findings related to scoping review question**	
Health System framework	
Eligibility	DHA reiterated the importance of member registry due to discrepancy with actual number of insured lives in Dubai, and reminded on penalties for non-compliance
Sector size	

User charges	
Institutional environment	
Legal framework	
Material regulation	
Prudential regulation	
Private Health Insurance policy framework	
Objective	
Population coverage	
System description	
Coverage type	
Market structure	
Concentration	
Barriers to entry	
Local vs international	
Vertical Integration	
Value Based Health Care	

Reviewer	Husein Reka
Date	20 January 2024
**Document information:**	
Name	Circular Number 7 of 2016 The eAuthorization Initiative
Author(s)	Dubai Health Authority
Year of publication	13 Oct 2016
Country/Emirate	Dubai
Link	https://www.isahd.ae/content/docs/GC%2007-2016%20The%20eClaimLink%20eAuthorization%20Initiative.pdf
Aim/purpose	Updates on the eAuthorization initiative on eClaimLink
Record #	217
**Inclusion/Exclusion Criteria**	
Population	Private Health Insurance in Dubai
Concept	Transformation/Reforms of Dubai Private Health Insurance Scheme
Context	GCC Health System
**Review framework domains studied in literature on private health insurance transformation**	
Health System Framework (e.g. eligibility, sector size, user charges)	
Institutional environment (e.g. legal framework, regulation)	Regulating health insurance information exchange
Private Health Insurance policy framework (e.g. objective, population coverage, system description, types of coverage	
Market structure, conduct and performance (e.g. market concentration, barriers to entry, local vs. international, vertical integration, value-based health care)	
**Key findings related to scoping review question**	
Health System framework	
Eligibility	
Sector size	
User charges	

Institutional environment	
Legal framework	
Material regulation	DHA updates market on the issuance ofa mandatory eAuthorization application for providers as part of Dubai’s health insurance exchange platform (eClaimLink)
Prudential regulation	
Private Health Insurance policy framework	
Objective	
Population coverage	
System description	
Coverage type	
Market structure	
Concentration	
Barriers to entry	
Local vs international	
Vertical Integration	
Value Based Health Care	

Reviewer	Husein Reka
Date	20 January 2024
**Document information:**	
Name	Circular Number 8 of 2016 Mandatory member information & Final Deadline
Author(s)	Dubai Health Authority
Year of publication	24 Nov 2016
Country/Emirate	Dubai
Link	https://www.isahd.ae/content/docs/GC%2008-2016%20Mandatory%20Member%20Information%20and%20Final%20Deadline.pdf
Aim/purpose	Notification on the implications on Dubai visa as a result of lack of mandated information being provided to Health Insurer's and TPA's
Record #	218
**Inclusion/Exclusion Criteria**	
Population	Private Health Insurance in Dubai
Concept	Transformation/Reforms of Dubai Private Health Insurance Scheme
Context	GCC Health System
**Review framework domains studied in literature on private health insurance transformation**	
Health System Framework (e.g. eligibility, sector size, user charges)	
Institutional environment (e.g. legal framework, regulation)	Compliance with Health Insurance Law and immigration rules
Private Health Insurance policy framework (e.g. objective, population coverage, system description, types of coverage	
Market structure, conduct and performance (e.g. market concentration, barriers to entry, local vs. international, vertical integration, value-based health care)	
**Key findings related to scoping review question**	

Health System framework	
Eligibility	
Sector size	
User charges	
Institutional environment	
Legal framework	DHA warns all parties that missing or erroneous data for mandatory data on mandatory insurance policies could lead to issues with visa issuance and renewal with General Directorate of Residency & Foreigners Affairs (GDRFA)
Material regulation	
Prudential regulation	
Private Health Insurance policy framework	
Objective	
Population coverage	
System description	
Coverage type	
Market structure	
Concentration	
Barriers to entry	
Local vs international	
Vertical Integration	
Value Based Health Care	

Reviewer	Husein Reka
Date	20 January 2024
**Document information:**	
Name	Circular Number 9 of 2016 Pro rata refund of premiums for individually sponsored members
Author(s)	Dubai Health Authority
Year of publication	30 Nov 2016
Country/Emirate	Dubai
Link	https://www.isahd.ae/content/docs/GC%2009-2016%20Premium%20Refunds.pdf
Aim/purpose	Obligations to refund premiums due to visa cancellation
Record #	219
**Inclusion/Exclusion Criteria**	
Population	Private Health Insurance in Dubai
Concept	Transformation/Reforms of Dubai Private Health Insurance Scheme
Context	GCC Health System
**Review framework domains studied in literature on private health insurance transformation**	
Health System Framework (e.g. eligibility, sector size, user charges)	
Institutional environment (e.g. legal framework, regulation)	Compliance with premium reimbursement rules
Private Health Insurance policy framework (e.g. objective, population coverage, system description, types of coverage	
Market structure, conduct and performance (e.g. market concentration, barriers to entry, local vs. international, vertical integration, value-based health care)	
**Key findings related to scoping review question**	
Health System framework	
Eligibility	
Sector size	
User charges	

Institutional environment	
Legal framework	
Material regulation	DHA reiterates through this circular the requirement to reimburse pro rata remaining premium to beneficiaries in case of visa cancellation or no renewal
Prudential regulation	
Private Health Insurance policy framework	
Objective	
Population coverage	
System description	
Coverage type	
Market structure	
Concentration	
Barriers to entry	
Local vs international	
Vertical Integration	
Value Based Health Care	

Reviewer	Husein Reka
Date	20 January 2024
**Document information:**	
Name	Circular Number 1 of 2017 Mandatory member information & Final Deadline
Author(s)	Dubai Health Authority
Year of publication	16 Jan 2017
Country/Emirate	Dubai
Link	https://www.isahd.ae/content/docs/GC%2001-2017%20Member%20Register%20Validation%20with%20GDRFAD.PDF
Aim/purpose	Activaiton of validation check for member registration
Record #	220
**Inclusion/Exclusion Criteria**	
Population	Private Health Insurance in Dubai
Concept	Transformation/Reforms of Dubai Private Health Insurance Scheme
Context	GCC Health System
**Review framework domains studied in literature on private health insurance transformation**	
Health System Framework (e.g. eligibility, sector size, user charges)	Member registration
Institutional environment (e.g. legal framework, regulation)	
Private Health Insurance policy framework (e.g. objective, population coverage, system description, types of coverage	
Market structure, conduct and performance (e.g. market concentration, barriers to entry, local vs. international, vertical integration, value-based health care)	
**Key findings related to scoping review question**	
Health System framework	
Eligibility	DHA informs market on the requirement to register members and the introduction of a validation check with

	General Directorate of Residency & Foreigners Affairs (GDRFA)
Sector size	
User charges	
Institutional environment	
Legal framework	
Material regulation	
Prudential regulation	
Private Health Insurance policy framework	
Objective	
Population coverage	
System description	
Coverage type	
Market structure	
Concentration	
Barriers to entry	
Local vs international	
Vertical Integration	
Value Based Health Care	

Reviewer	Husein Reka
Date	20 January 2024
**Document information:**	
Name	Circular Number 1 of 2017 Mandatory member information & Final Deadline
Author(s)	Dubai Health Authority
Year of publication	16 Jan 2017
Country/Emirate	Dubai
Link	https://www.isahd.ae/content/docs/GC%2001-2017%20Member%20Register%20Validation%20with%20GDRFAD.PDF
Aim/purpose	Activaiton of validation check for member registration
Record #	220
**Inclusion/Exclusion Criteria**	
Population	Private Health Insurance in Dubai
Concept	Transformation/Reforms of Dubai Private Health Insurance Scheme
Context	GCC Health System
**Review framework domains studied in literature on private health insurance transformation**	
Health System Framework (e.g. eligibility, sector size, user charges)	Member registration
Institutional environment (e.g. legal framework, regulation)	
Private Health Insurance policy framework (e.g. objective, population coverage, system description, types of coverage	
Market structure, conduct and performance (e.g. market concentration, barriers to entry, local vs. international, vertical integration, value-based health care)	
**Key findings related to scoping review question**	
Health System framework	
Eligibility	DHA informs market on the requirement to register members and the introduction of a validation check with

	General Directorate of Residency & Foreigners Affairs (GDRFA)
Sector size	
User charges	
Institutional environment	
Legal framework	
Material regulation	
Prudential regulation	
Private Health Insurance policy framework	
Objective	
Population coverage	
System description	
Coverage type	
Market structure	
Concentration	
Barriers to entry	
Local vs international	
Vertical Integration	
Value Based Health Care	

Reviewer	Husein Reka
Date	20 January 2024
**Document information:**	
Name	Circular Number 2 of 2017 Member Register failed uploads
Author(s)	Dubai Health Authority
Year of publication	26 Mar 2017
Country/Emirate	Dubai
Link	https://www.isahd.ae/content/docs/GC%2002-2017%20Member%20Register%20Failed%20Uploads.pdf
Aim/purpose	Importance of timely upload to the member register and requirements for failed uploads
Record #	221
**Inclusion/Exclusion Criteria**	
Population	Private Health Insurance in Dubai
Concept	Transformation/Reforms of Dubai Private Health Insurance Scheme
Context	GCC Health System
**Review framework domains studied in literature on private health insurance transformation**	
Health System Framework (e.g. eligibility, sector size, user charges)	Member registration
Institutional environment (e.g. legal framework, regulation)	
Private Health Insurance policy framework (e.g. objective, population coverage, system description, types of coverage	
Market structure, conduct and performance (e.g. market concentration, barriers to entry, local vs. international, vertical integration, value-based health care)	
**Key findings related to scoping review question**	
Health System framework	
Eligibility	DHA reiterates the responsibilities of insurers and third party administrators in uploading members in real time

	and the requirement for insurance certificate issuance in the next 6 months
Sector size	
User charges	
Institutional environment	
Legal framework	
Material regulation	
Prudential regulation	
Private Health Insurance policy framework	
Objective	
Population coverage	
System description	
Coverage type	
Market structure	
Concentration	
Barriers to entry	
Local vs international	
Vertical Integration	
Value Based Health Care	

Reviewer	Husein Reka
Date	20 January 2024
**Document information:**	
Name	Circular Number 3 of 2017 DRG Implementation – Phase II of Shadow Billing
Author(s)	Dubai Health Authority
Year of publication	15 Oct 2017
Country/Emirate	Dubai
Link	https://www.isahd.ae/content/docs/General%20Circular%20DRG%20Implementation%2004102017%20v3.pdf
Aim/purpose	To advise market participants the effective date of DRG Implementation – Phase II of Shadow Billing
Record #	222
**Inclusion/Exclusion Criteria**	
Population	Private Health Insurance in Dubai
Concept	Transformation/Reforms of Dubai Private Health Insurance Scheme
Context	GCC Health System
**Review framework domains studied in literature on private health insurance transformation**	
Health System Framework (e.g. eligibility, sector size, user charges)	
Institutional environment (e.g. legal framework, regulation)	
Private Health Insurance policy framework (e.g. objective, population coverage, system description, types of coverage	
Market structure, conduct and performance (e.g. market concentration, barriers to entry, local vs. international, vertical integration, value-based health care)	Reimbursement reforms
**Key findings related to scoping review question**	
Health System framework	
Eligibility	
Sector size	

User charges	
Institutional environment	
Legal framework	
Material regulation	
Prudential regulation	
Private Health Insurance policy framework	
Objective	
Population coverage	
System description	
Coverage type	
Market structure	
Concentration	
Barriers to entry	
Local vs international	
Vertical Integration	
Value Based Health Care	DHA informs market on Phase 2 of DRG shadow billing period and encourages market to deliver best practice in clinical coding and documentation, and timely submission of claims

Reviewer	Husein Reka
Date	20 January 2024
**Document information:**	
Name	Circular Number 1 of 2018 DRG Implementation –Phase II of Shadow Billing
Author(s)	Dubai Health Authority
Year of publication	25 Feb 2018
Country/Emirate	Dubai
Link	https://www.isahd.ae/content/docs/GC%2001-2018%20DRG%20Implementation%20-%20Phase%20II%20of%20Shadow%20Billing.pdf
Aim/purpose	Continuation of Phase II of Shadow Billing throughout 2018
Record #	225
**Inclusion/Exclusion Criteria**	
Population	Private Health Insurance in Dubai
Concept	Transformation/Reforms of Dubai Private Health Insurance Scheme
Context	GCC Health System
**Review framework domains studied in literature on private health insurance transformation**	
Health System Framework (e.g. eligibility, sector size, user charges)	
Institutional environment (e.g. legal framework, regulation)	
Private Health Insurance policy framework (e.g. objective, population coverage, system description, types of coverage	
Market structure, conduct and performance (e.g. market concentration, barriers to entry, local vs. international, vertical integration, value-based health care)	Reimbursement reforms
**Key findings related to scoping review question**	
Health System framework	
Eligibility	

Sector size	
User charges	
Institutional environment	
Legal framework	
Material regulation	
Prudential regulation	
Private Health Insurance policy framework	
Objective	
Population coverage	
System description	
Coverage type	
Market structure	
Concentration	
Barriers to entry	
Local vs international	
Vertical Integration	
Value Based Health Care	DHA informs market that it aims Phase 2 DRG shadow billing to continue until end of 2018 in preparation for full implementation in 2019

Reviewer	Husein Reka
Date	20 January 2024
**Document information:**	
Name	Circular Number 2 of 2018 Collection of DRG Negotiation Factors
Author(s)	Dubai Health Authority
Year of publication	4 Apr 2018
Country/Emirate	Dubai
Link	https://www.isahd.ae/content/docs/General%20Circular%20DRG%20Negotiation%20Factors%2004042018.pdf
Aim/purpose	Collection of DRG negotiation factors from providers and payers during DRG Implementation Phase II of Shadow Billing
Record #	226
**Inclusion/Exclusion Criteria**	
Population	Private Health Insurance in Dubai
Concept	Transformation/Reforms of Dubai Private Health Insurance Scheme
Context	GCC Health System
**Review framework domains studied in literature on private health insurance transformation**	
Health System Framework (e.g. eligibility, sector size, user charges)	
Institutional environment (e.g. legal framework, regulation)	
Private Health Insurance policy framework (e.g. objective, population coverage, system description, types of coverage	
Market structure, conduct and performance (e.g. market concentration, barriers to entry, local vs. international, vertical integration, value-based health care)	Reimbursement reforms
**Key findings related to scoping review question**	
Health System framework	
Eligibility	

Sector size	
User charges	
Institutional environment	
Legal framework	
Material regulation	
Prudential regulation	
Private Health Insurance policy framework	
Objective	
Population coverage	
System description	
Coverage type	
Market structure	
Concentration	
Barriers to entry	
Local vs international	
Vertical Integration	
Value Based Health Care	DHA requests payers and providers to submit negotiation factors with them by 26 June 2018

Reviewer	Husein Reka
Date	20 January 2024
**Document information:**	
Name	Circular Number 4 of 2018 The ePrescription Initiative – Enforcement of the eRx Cycle, eRx Reference Number & Pharmacist Edit Restrictions
Author(s)	Dubai Health Authority
Year of publication	12 Apr 2018
Country/Emirate	Dubai
Link	https://www.isahd.ae/content/docs/eClaimLink%20General%20Circular%20GC04%202018%20-%20The%20ePrescription%20Initiative%20-%20Update.pdf
Aim/purpose	Market updates on the ePrescription mandate
Record #	228
**Inclusion/Exclusion Criteria**	
Population	Private Health Insurance in Dubai
Concept	Transformation/Reforms of Dubai Private Health Insurance Scheme
Context	GCC Health System
**Review framework domains studied in literature on private health insurance transformation**	
Health System Framework (e.g. eligibility, sector size, user charges)	
Institutional environment (e.g. legal framework, regulation)	Mandating of ePrescription system for health insurance
Private Health Insurance policy framework (e.g. objective, population coverage, system description, types of coverage	
Market structure, conduct and performance (e.g. market concentration, barriers to entry, local vs. international, vertical integration, value-based health care)	
**Key findings related to scoping review question**	
Health System framework	

Eligibility	
Sector size	
User charges	
Institutional environment	
Legal framework	
Material regulation	DHA mandates ePrescription system for health insurance
Prudential regulation	
Private Health Insurance policy framework	
Objective	
Population coverage	
System description	
Coverage type	
Market structure	
Concentration	
Barriers to entry	
Local vs international	
Vertical Integration	
Value Based Health Care	

Reviewer	Husein Reka
Date	20 January 2024
**Document information:**	
Name	Circular Number 7 of 2018 DRG implementation exempted hospitals
Author(s)	Dubai Health Authority
Year of publication	20 Jun 2018
Country/Emirate	Dubai
Link	https://www.isahd.ae/content/docs/eClaimLink%20General%20Circular%20GC07%202018%20-%20DRG%20Implementation%20-%20Exempted%20Hospitals.pdf
Aim/purpose	Clarification to market on exempted hospitals for DRG implementation
Record #	231
**Inclusion/Exclusion Criteria**	
Population	Private Health Insurance in Dubai
Concept	Transformation/Reforms of Dubai Private Health Insurance Scheme
Context	GCC Health System
**Review framework domains studied in literature on private health insurance transformation**	
Health System Framework (e.g. eligibility, sector size, user charges)	
Institutional environment (e.g. legal framework, regulation)	
Private Health Insurance policy framework (e.g. objective, population coverage, system description, types of coverage	
Market structure, conduct and performance (e.g. market concentration, barriers to entry, local vs. international, vertical integration, value-based health care)	Reimbursement reforms
**Key findings related to scoping review question**	
Health System framework	

Eligibility	
Sector size	
User charges	
Institutional environment	
Legal framework	
Material regulation	
Prudential regulation	
Private Health Insurance policy framework	
Objective	
Population coverage	
System description	
Coverage type	
Market structure	
Concentration	
Barriers to entry	
Local vs international	
Vertical Integration	
Value Based Health Care	DHA informs market on a number of hospitals exempted from shadow billing DRG implementation (DHA hospitals, MOHP hospitals and non-DHA/non-DHCC licensed hospitals)

Reviewer	Husein Reka
Date	20 January 2024
**Document information:**	
Name	Circular Number 8 of 2018 Clarifications on Collection of DRG Negotiation Factors and Reporting Requirements of DRG Financial Parameters in Phase II of Shadow Billing
Author(s)	Dubai Health Authority
Year of publication	29 Jul 2018
Country/Emirate	Dubai
Link	https://www.isahd.ae/content/docs/eClaimLink%20General%20Circular%20GC08%202018%20-%20Clarifications%20on%20Collection%20of%20DRG%20Negotiation%20Factors%20and%20Reporting%20Requirements%20of%20DRG%20Financial%20Parameters%2029072018.pdf
Aim/purpose	Clarification to market on collection of DRG negotiation factors
Record #	232
**Inclusion/Exclusion Criteria**	
Population	Private Health Insurance in Dubai
Concept	Transformation/Reforms of Dubai Private Health Insurance Scheme
Context	GCC Health System
**Review framework domains studied in literature on private health insurance transformation**	
Health System Framework (e.g. eligibility, sector size, user charges)	
Institutional environment (e.g. legal framework, regulation)	
Private Health Insurance policy framework (e.g. objective, population coverage, system description, types of coverage	

Market structure, conduct and performance (e.g. market concentration, barriers to entry, local vs. international, vertical integration, value-based health care)	Reimbursement reforms
**Key findings related to scoping review question**	
Health System framework	
Eligibility	
Sector size	
User charges	
Institutional environment	
Legal framework	
Material regulation	
Prudential regulation	
Private Health Insurance policy framework	
Objective	
Population coverage	
System description	
Coverage type	
Market structure	
Concentration	
Barriers to entry	
Local vs international	
Vertical Integration	
Value Based Health Care	DHA issues final deadline for payers and providers who have not finalized their negotiations to submit them by 20 Aug 2018, and reporting requirements of DRG parameters

Reviewer	Husein Reka
Date	20 January 2024
**Document information:**	
Name	Circular Number 10 of 2018 Cessation of Direct Billing Paper Claim Forms
Author(s)	Dubai Health Authority
Year of publication	1 Nov 2018
Country/Emirate	Dubai
Link	https://www.isahd.ae/content/docs/GC10%202018%20-%20Cessation%20of%20Direct%20Billing%20Paper%20Claim%20Forms.pdf
Aim/purpose	Cessation of paper claims for direct billing
Record #	234
**Inclusion/Exclusion Criteria**	
Population	Private Health Insurance in Dubai
Concept	Transformation/Reforms of Dubai Private Health Insurance Scheme
Context	GCC Health System
**Review framework domains studied in literature on private health insurance transformation**	
Health System Framework (e.g. eligibility, sector size, user charges)	
Institutional environment (e.g. legal framework, regulation)	Billing regulation
Private Health Insurance policy framework (e.g. objective, population coverage, system description, types of coverage	
Market structure, conduct and performance (e.g. market concentration, barriers to entry, local vs. international, vertical integration, value-based health care)	
**Key findings related to scoping review question**	
Health System framework	
Eligibility	
Sector size	

User charges	
Institutional environment	
Legal framework	
Material regulation	DHA informs market on paper form cessation for billing purposes and the requirement to secure patient consent form for disclosure of medical details for billing purposes
Prudential regulation	
Private Health Insurance policy framework	
Objective	
Population coverage	
System description	
Coverage type	
Market structure	
Concentration	
Barriers to entry	
Local vs international	
Vertical Integration	
Value Based Health Care	

Reviewer	Husein Reka
Date	20 January 2024
**Document information:**	
Name	Circular Number 01 of 2019 Unnecessary medical treatments, diagnostics or medications at medical providers
Author(s)	Dubai Health Authority
Year of publication	14 Jan 2019
Country/Emirate	Dubai
Link	https://www.isahd.ae/content/docs/GC%2001-2019.pdf
Aim/purpose	Waring providers on prolonged hospital stay and overutilization of medical treatments
Record #	237
**Inclusion/Exclusion Criteria**	
Population	Private Health Insurance in Dubai
Concept	Transformation/Reforms of Dubai Private Health Insurance Scheme
Context	GCC Health System
**Review framework domains studied in literature on private health insurance transformation**	
Health System Framework (e.g. eligibility, sector size, user charges)	
Institutional environment (e.g. legal framework, regulation)	Fraud waste and abuse
Private Health Insurance policy framework (e.g. objective, population coverage, system description, types of coverage	
Market structure, conduct and performance (e.g. market concentration, barriers to entry, local vs. international, vertical integration, value-based health care)	
**Key findings related to scoping review question**	
Health System framework	
Eligibility	
Sector size	

User charges	
Institutional environment	
Legal framework	
Material regulation	DHA based on number of complaints from both payers and providers on prolonged discharge of patients, overutilization of services, and refusal to settle claims, has published the mechanisms of raising escalations with the regulator and repercussions
Prudential regulation	
Private Health Insurance policy framework	
Objective	
Population coverage	
System description	
Coverage type	
Market structure	
Concentration	
Barriers to entry	
Local vs international	
Vertical Integration	
Value Based Health Care	

Reviewer	Husein Reka
Date	20 January 2024
**Document information:**	
Name	Circular Number 02 of 2019 Pro rata refund of premiums for individually sponsored members
Author(s)	Dubai Health Authority
Year of publication	28 Mar 2019
Country/Emirate	Dubai
Link	https://www.isahd.ae/content/docs/GC%2002-2019%20v1%20Premium%20Refunds%2028032019.pdf
Aim/purpose	Clarify the practice of refunds for insured members under individual sponsorship
Record #	238
**Inclusion/Exclusion Criteria**	
Population	Private Health Insurance in Dubai
Concept	Transformation/Reforms of Dubai Private Health Insurance Scheme
Context	GCC Health System
**Review framework domains studied in literature on private health insurance transformation**	
Health System Framework (e.g. eligibility, sector size, user charges)	
Institutional environment (e.g. legal framework, regulation)	Premium reimbursement
Private Health Insurance policy framework (e.g. objective, population coverage, system description, types of coverage	
Market structure, conduct and performance (e.g. market concentration, barriers to entry, local vs. international, vertical integration, value-based health care)	
**Key findings related to scoping review question**	
Health System framework	
Eligibility	
Sector size	

User charges	
Institutional environment	
Legal framework	
Material regulation	DHA replaced previous circular on this topic (Circular no. 09 of 2016) with this circular by providing alternative scenarios on cases when policy cancellation and refund is made
Prudential regulation	
Private Health Insurance policy framework	
Objective	
Population coverage	
System description	
Coverage type	
Market structure	
Concentration	
Barriers to entry	
Local vs international	
Vertical Integration	
Value Based Health Care	

Reviewer	Husein Reka
Date	20 January 2024
**Document information:**	
Name	Circular Number 07 of 2019 Ethical Standards of Business Conduct
Author(s)	Dubai Health Authority
Year of publication	7 Jul 2019
Country/Emirate	Dubai
Link	https://www.isahd.ae/content/docs/GC%2007-2019.pdf
Aim/purpose	Ensure compliance to ethical standards for business conduct
Record #	243
**Inclusion/Exclusion Criteria**	
Population	Private Health Insurance in Dubai
Concept	Transformation/Reforms of Dubai Private Health Insurance Scheme
Context	GCC Health System
**Review framework domains studied in literature on private health insurance transformation**	
Health System Framework (e.g. eligibility, sector size, user charges)	
Institutional environment (e.g. legal framework, regulation)	Conduct of business ethical standards
Private Health Insurance policy framework (e.g. objective, population coverage, system description, types of coverage	
Market structure, conduct and performance (e.g. market concentration, barriers to entry, local vs. international, vertical integration, value-based health care)	
**Key findings related to scoping review question**	
Health System framework	
Eligibility	
Sector size	
User charges	

Institutional environment	
Legal framework	
Material regulation	DHA published a list of ethical standards for conduct of business for market to adopt in support of DHA objectives as a regulator
Prudential regulation	
Private Health Insurance policy framework	
Objective	
Population coverage	
System description	
Coverage type	
Market structure	
Concentration	
Barriers to entry	
Local vs international	
Vertical Integration	
Value Based Health Care	

Reviewer	Husein Reka
Date	20 January 2024
**Document information:**	
Name	Circular Number 03 of 2020 Upgrade of ICD-10-CM, CPT and HCPCS Code Sets to Version 2018
Author(s)	Dubai Health Authority
Year of publication	13 Feb 2020
Country/Emirate	Dubai
Link	https://www.isahd.ae/content/docs/GC%2003-2020%20Upgrade%20of%20ICD-10-CM,%20CPT%20and%20HCPCS%20Code%20Sets%20to%20Version%202018.pdf
Aim/purpose	Notive on the upgrade of ICD-10-CM, CPT and HCPCS code sets to version 2018
Record #	248
**Inclusion/Exclusion Criteria**	
Population	Private Health Insurance in Dubai
Concept	Transformation/Reforms of Dubai Private Health Insurance Scheme
Context	GCC Health System
**Review framework domains studied in literature on private health insurance transformation**	
Health System Framework (e.g. eligibility, sector size, user charges)	
Institutional environment (e.g. legal framework, regulation)	
Private Health Insurance policy framework (e.g. objective, population coverage, system description, types of coverage	
Market structure, conduct and performance (e.g. market concentration, barriers to entry, local vs. international, vertical integration, value-based health care)	Reimbursement reforms
**Key findings related to scoping review question**	
Health System framework	

Eligibility	
Sector size	
User charges	
Institutional environment	
Legal framework	
Material regulation	
Prudential regulation	
Private Health Insurance policy framework	
Objective	
Population coverage	
System description	
Coverage type	
Market structure	
Concentration	
Barriers to entry	
Local vs international	
Vertical Integration	
Value Based Health Care	DHA informs market that as of 1 April 2020 all service providers are mandated to submit claims using 2018 versions of ICD-10- CM, CPT and HCPCS

Reviewer	Husein Reka
Date	20 January 2024
**Document information:**	
Name	Circular Number 03 of 2020 Upgrade of ICD-10-CM, CPT and HCPCS Code Sets to Version 2018
Author(s)	Dubai Health Authority
Year of publication	13 Feb 2020
Country/Emirate	Dubai
Link	https://www.isahd.ae/content/docs/GC%2003-2020%20Upgrade%20of%20ICD-10-CM,%20CPT%20and%20HCPCS%20Code%20Sets%20to%20Version%202018.pdf
Aim/purpose	Notice on the upgrade of ICD-10-CM, CPT and HCPCS code sets to version 2018
Record #	248
**Inclusion/Exclusion Criteria**	
Population	Private Health Insurance in Dubai
Concept	Transformation/Reforms of Dubai Private Health Insurance Scheme
Context	GCC Health System
**Review framework domains studied in literature on private health insurance transformation**	
Health System Framework (e.g. eligibility, sector size, user charges)	
Institutional environment (e.g. legal framework, regulation)	
Private Health Insurance policy framework (e.g. objective, population coverage, system description, types of coverage	
Market structure, conduct and performance (e.g. market concentration, barriers to entry, local vs. international, vertical integration, value-based health care)	Reimbursement reforms
**Key findings related to scoping review question**	
Health System framework	

Eligibility	
Sector size	
User charges	
Institutional environment	
Legal framework	
Material regulation	
Prudential regulation	
Private Health Insurance policy framework	
Objective	
Population coverage	
System description	
Coverage type	
Market structure	
Concentration	
Barriers to entry	
Local vs international	
Vertical Integration	
Value Based Health Care	DHA informs market that as of 1 April 2020 all service providers are mandated to submit claims using 2018 versions of ICD-10- CM, CPT and HCPCS

Reviewer	Husein Reka
Date	20 January 2024
**Document information:**	
Name	Circular Number 04 of 2020 Full DRG Implementation
Author(s)	Dubai Health Authority
Year of publication	13 Feb 2020
Country/Emirate	Dubai
Link	https://www.isahd.ae/content/docs/GC%2004-2020%20Full%20DRG%20Implementation.pdf
Aim/purpose	Notice on full DRG implementation
Record #	252
**Inclusion/Exclusion Criteria**	
Population	Private Health Insurance in Dubai
Concept	Transformation/Reforms of Dubai Private Health Insurance Scheme
Context	GCC Health System
**Review framework domains studied in literature on private health insurance transformation**	
Health System Framework (e.g. eligibility, sector size, user charges)	
Institutional environment (e.g. legal framework, regulation)	
Private Health Insurance policy framework (e.g. objective, population coverage, system description, types of coverage	
Market structure, conduct and performance (e.g. market concentration, barriers to entry, local vs. international, vertical integration, value-based health care)	Reimbursement reforms
**Key findings related to scoping review question**	
Health System framework	
Eligibility	
Sector size	
User charges	
Institutional environment	

Legal framework	
Material regulation	
Prudential regulation	
Private Health Insurance policy framework	
Objective	
Population coverage	
System description	
Coverage type	
Market structure	
Concentration	
Barriers to entry	
Local vs international	
Vertical Integration	
Value Based Health Care	DHA informed market that full DRG Implementation will be effective as of 00:00, 1 April 2020 for all DHA and DHCC licensed hospitals

Reviewer	Husein Reka
Date	20 January 2024
**Document information:**	
Name	Circular Number 05 of 2020 Settlement of Outstanding claims and Update of claim payments cycle
Author(s)	Dubai Health Authority
Year of publication	31 Mar 2020
Country/Emirate	Dubai
Link	https://www.isahd.ae/content/docs/GC%2005-2020.pdf
Aim/purpose	Requirement to settle all outstanding claims
Record #	253
**Inclusion/Exclusion Criteria**	
Population	Private Health Insurance in Dubai
Concept	Transformation/Reforms of Dubai Private Health Insurance Scheme
Context	GCC Health System
**Review framework domains studied in literature on private health insurance transformation**	
Health System Framework (e.g. eligibility, sector size, user charges)	
Institutional environment (e.g. legal framework, regulation)	Claims settlements
Private Health Insurance policy framework (e.g. objective, population coverage, system description, types of coverage	
Market structure, conduct and performance (e.g. market concentration, barriers to entry, local vs. international, vertical integration, value-based health care)	
**Key findings related to scoping review question**	
Health System framework	
Eligibility	
Sector size	
User charges	
Institutional environment	

Legal framework	
Material regulation	DHA requires all payers to settle all current outstanding and agreed claims or any issued unsettled GOPs received as of March 30th 2020 to their respective network providers, regardless of timelines stated in mutual contractual agreements
Prudential regulation	
Private Health Insurance policy framework	
Objective	
Population coverage	
System description	
Coverage type	
Market structure	
Concentration	
Barriers to entry	
Local vs international	
Vertical Integration	
Value Based Health Care	

Reviewer	Husein Reka
Date	20 January 2024
**Document information:**	
Name	Circular Number 06 of 2020 Postponement of Full DRG Implementation
Author(s)	Dubai Health Authority
Year of publication	26 Mar 2020
Country/Emirate	Dubai
Link	https://www.isahd.ae/content/docs/GC%2006-2020.pdf
Aim/purpose	Notice on postponement of full DRG implementation
Record #	254
**Inclusion/Exclusion Criteria**	
Population	Private Health Insurance in Dubai
Concept	Transformation/Reforms of Dubai Private Health Insurance Scheme
Context	GCC Health System
**Review framework domains studied in literature on private health insurance transformation**	
Health System Framework (e.g. eligibility, sector size, user charges)	
Institutional environment (e.g. legal framework, regulation)	
Private Health Insurance policy framework (e.g. objective, population coverage, system description, types of coverage	
Market structure, conduct and performance (e.g. market concentration, barriers to entry, local vs. international, vertical integration, value-based health care)	Reimbursement reforms
**Key findings related to scoping review question**	
Health System framework	
Eligibility	
Sector size	
User charges	
Institutional environment	

Legal framework	
Material regulation	
Prudential regulation	
Private Health Insurance policy framework	
Objective	
Population coverage	
System description	
Coverage type	
Market structure	
Concentration	
Barriers to entry	
Local vs international	
Vertical Integration	
Value Based Health Care	DHA announced the postponement of full DRG implementation to 1 May 2020 due to more time required for preparations

Reviewer	Husein Reka
Date	20 January 2024
**Document information:**	
Name	Circular Number 07 of 2020 Postponement of Upgrade of ICD-10-CM, CPT and HCPCS Code Sets to Version 2018
Author(s)	Dubai Health Authority
Year of publication	26 Mar 2020
Country/Emirate	Dubai
Link	https://www.isahd.ae/content/docs/GC%2007-2020.pdf
Aim/purpose	Notice on postponement of the upgrade of ICD-10-CM, CPT and HCPCS code sets to version 2018
Record #	255
**Inclusion/Exclusion Criteria**	
Population	Private Health Insurance in Dubai
Concept	Transformation/Reforms of Dubai Private Health Insurance Scheme
Context	GCC Health System
**Review framework domains studied in literature on private health insurance transformation**	
Health System Framework (e.g. eligibility, sector size, user charges)	
Institutional environment (e.g. legal framework, regulation)	
Private Health Insurance policy framework (e.g. objective, population coverage, system description, types of coverage	
Market structure, conduct and performance (e.g. market concentration, barriers to entry, local vs. international, vertical integration, value-based health care)	Reimbursement reforms
**Key findings related to scoping review question**	
Health System framework	
Eligibility	
Sector size	
User charges	

Institutional environment	
Legal framework	
Material regulation	
Prudential regulation	
Private Health Insurance policy framework	
Objective	
Population coverage	
System description	
Coverage type	
Market structure	
Concentration	
Barriers to entry	
Local vs international	
Vertical Integration	
Value Based Health Care	DHA announced the postponement of the upgrade of ICD-10-CM, CPT and HCPCS code sets to version 2018 to 1 May 2020 due to more time required for preparations

Reviewer	Husein Reka
Date	20 January 2024
**Document information:**	
Name	Circular Number 11 of 2020 Postponement of Full DRG Implement
Author(s)	Dubai Health Authority
Year of publication	24 Apr 2020
Country/Emirate	Dubai
Link	https://www.isahd.ae/content/docs/GC%2011-2020.pdf
Aim/purpose	Notice on postponement of full DRG Implementation
Record #	259
**Inclusion/Exclusion Criteria**	
Population	Private Health Insurance in Dubai
Concept	Transformation/Reforms of Dubai Private Health Insurance Scheme
Context	GCC Health System
**Review framework domains studied in literature on private health insurance transformation**	
Health System Framework (e.g. eligibility, sector size, user charges)	
Institutional environment (e.g. legal framework, regulation)	
Private Health Insurance policy framework (e.g. objective, population coverage, system description, types of coverage	
Market structure, conduct and performance (e.g. market concentration, barriers to entry, local vs. international, vertical integration, value-based health care)	Reimbursement reforms
**Key findings related to scoping review question**	
Health System framework	
Eligibility	
Sector size	
User charges	
Institutional environment	

Legal framework	
Material regulation	
Prudential regulation	
Private Health Insurance policy framework	
Objective	
Population coverage	
System description	
Coverage type	
Market structure	
Concentration	
Barriers to entry	
Local vs international	
Vertical Integration	
Value Based Health Care	DHA announced the postponement of full DRG implementation to 1 June 2020 due to more time required for preparations

Reviewer	Husein Reka
Date	20 January 2024
**Document information:**	
Name	Circular Number 12 of 2020 Postponement of Upgrade of ICD-10-CM, CPT and HCPCS Code Sets to Version 2018
Author(s)	Dubai Health Authority
Year of publication	21 Apr 2020
Country/Emirate	Dubai
Link	https://www.isahd.ae/content/docs/GC%2012-2020.pdf
Aim/purpose	Notice on postponement of the upgrade of ICD-10-CM, CPT and HCPCS code sets to version 2018
Record #	260
**Inclusion/Exclusion Criteria**	
Population	Private Health Insurance in Dubai
Concept	Transformation/Reforms of Dubai Private Health Insurance Scheme
Context	GCC Health System
**Review framework domains studied in literature on private health insurance transformation**	
Health System Framework (e.g. eligibility, sector size, user charges)	
Institutional environment (e.g. legal framework, regulation)	
Private Health Insurance policy framework (e.g. objective, population coverage, system description, types of coverage	
Market structure, conduct and performance (e.g. market concentration, barriers to entry, local vs. international, vertical integration, value-based health care)	Reimbursement reforms
**Key findings related to scoping review question**	
Health System framework	
Eligibility	
Sector size	
User charges	

Institutional environment	
Legal framework	
Material regulation	
Prudential regulation	
Private Health Insurance policy framework	
Objective	
Population coverage	
System description	
Coverage type	
Market structure	
Concentration	
Barriers to entry	
Local vs international	
Vertical Integration	
Value Based Health Care	DHA announced the postponement of the upgrade of ICD-10-CM, CPT and HCPCS code sets to version 2018 to 1 June 2020 due to more time required for preparations

Reviewer	Husein Reka
Date	20 January 2024
**Document information:**	
Name	Circular Number 16 of 2020 Postponement of Full DRG Implement
Author(s)	Dubai Health Authority
Year of publication	28 May 2020
Country/Emirate	Dubai
Link	https://www.isahd.ae/content/docs/GC%2016-2020.pdf
Aim/purpose	Notice on postponement of full DRG Implementation
Record #	264
**Inclusion/Exclusion Criteria**	
Population	Private Health Insurance in Dubai
Concept	Transformation/Reforms of Dubai Private Health Insurance Scheme
Context	GCC Health System
**Review framework domains studied in literature on private health insurance transformation**	
Health System Framework (e.g. eligibility, sector size, user charges)	
Institutional environment (e.g. legal framework, regulation)	
Private Health Insurance policy framework (e.g. objective, population coverage, system description, types of coverage	
Market structure, conduct and performance (e.g. market concentration, barriers to entry, local vs. international, vertical integration, value-based health care)	Reimbursement reforms
**Key findings related to scoping review question**	
Health System framework	
Eligibility	
Sector size	
User charges	
Institutional environment	

Legal framework	
Material regulation	
Prudential regulation	
Private Health Insurance policy framework	
Objective	
Population coverage	
System description	
Coverage type	
Market structure	
Concentration	
Barriers to entry	
Local vs international	
Vertical Integration	
Value Based Health Care	DHA announced the postponement of full DRG implementation to 1 July 2020 due to more time required for preparations

Reviewer	Husein Reka
Date	20 January 2024
**Document information:**	
Name	Circular Number 17 of 2020 Postponement of Upgrade of ICD-10-CM, CPT and HCPCS Code Sets to Version 2018
Author(s)	Dubai Health Authority
Year of publication	28 May 2020
Country/Emirate	Dubai
Link	https://www.isahd.ae/content/docs/GC%2017-2020.pdf
Aim/purpose	Notice on postponement of the upgrade of ICD-10-CM, CPT and HCPCS code sets to version 2018
Record #	265
**Inclusion/Exclusion Criteria**	
Population	Private Health Insurance in Dubai
Concept	Transformation/Reforms of Dubai Private Health Insurance Scheme
Context	GCC Health System
**Review framework domains studied in literature on private health insurance transformation**	
Health System Framework (e.g. eligibility, sector size, user charges)	
Institutional environment (e.g. legal framework, regulation)	
Private Health Insurance policy framework (e.g. objective, population coverage, system description, types of coverage	
Market structure, conduct and performance (e.g. market concentration, barriers to entry, local vs. international, vertical integration, value-based health care)	Reimbursement reforms
**Key findings related to scoping review question**	
Health System framework	
Eligibility	
Sector size	
User charges	

Institutional environment	
Legal framework	
Material regulation	
Prudential regulation	
Private Health Insurance policy framework	
Objective	
Population coverage	
System description	
Coverage type	
Market structure	
Concentration	
Barriers to entry	
Local vs international	
Vertical Integration	
Value Based Health Care	DHA announced the postponement of the upgrade of ICD-10-CM, CPT and HCPCS code sets to version 2018 to 1 July 2020 due to more time required for preparations

Reviewer	Husein Reka
Date	20 January 2024
**Document information:**	
Name	Circular Number 19 of 2020 Postponement of Full DRG Implement
Author(s)	Dubai Health Authority
Year of publication	30 June 2020
Country/Emirate	Dubai
Link	https://www.isahd.ae/content/docs/GC%2019-2020.pdf
Aim/purpose	Notice on postponement of full DRG Implementation
Record #	267
**Inclusion/Exclusion Criteria**	
Population	Private Health Insurance in Dubai
Concept	Transformation/Reforms of Dubai Private Health Insurance Scheme
Context	GCC Health System
**Review framework domains studied in literature on private health insurance transformation**	
Health System Framework (e.g. eligibility, sector size, user charges)	
Institutional environment (e.g. legal framework, regulation)	
Private Health Insurance policy framework (e.g. objective, population coverage, system description, types of coverage	
Market structure, conduct and performance (e.g. market concentration, barriers to entry, local vs. international, vertical integration, value-based health care)	Reimbursement reforms
**Key findings related to scoping review question**	
Health System framework	
Eligibility	
Sector size	
User charges	
Institutional environment	

Legal framework	
Material regulation	
Prudential regulation	
Private Health Insurance policy framework	
Objective	
Population coverage	
System description	
Coverage type	
Market structure	
Concentration	
Barriers to entry	
Local vs international	
Vertical Integration	
Value Based Health Care	DHA announced the postponement of full DRG implementation to 1 September 2020 due to more time required for preparations

Reviewer	Husein Reka
Date	20 January 2024
**Document information:**	
Name	Circular Number 20 of 2020 Postponement of Upgrade of ICD-10-CM, CPT and HCPCS Code Sets to Version 2018
Author(s)	Dubai Health Authority
Year of publication	30 Jun 2020
Country/Emirate	Dubai
Link	https://www.isahd.ae/content/docs/GC%2020-2020.pdf
Aim/purpose	Notice on postponement of the upgrade of ICD-10-CM, CPT and HCPCS code sets to version 2018
Record #	268
**Inclusion/Exclusion Criteria**	
Population	Private Health Insurance in Dubai
Concept	Transformation/Reforms of Dubai Private Health Insurance Scheme
Context	GCC Health System
**Review framework domains studied in literature on private health insurance transformation**	
Health System Framework (e.g. eligibility, sector size, user charges)	
Institutional environment (e.g. legal framework, regulation)	
Private Health Insurance policy framework (e.g. objective, population coverage, system description, types of coverage	
Market structure, conduct and performance (e.g. market concentration, barriers to entry, local vs. international, vertical integration, value-based health care)	Reimbursement reforms
**Key findings related to scoping review question**	
Health System framework	
Eligibility	
Sector size	
User charges	

Institutional environment	
Legal framework	
Material regulation	
Prudential regulation	
Private Health Insurance policy framework	
Objective	
Population coverage	
System description	
Coverage type	
Market structure	
Concentration	
Barriers to entry	
Local vs international	
Vertical Integration	
Value Based Health Care	DHA announced the postponement of the upgrade of ICD-10-CM, CPT and HCPCS code sets to version 2018 to 1 September 2020 due to more time required for preparations

Reviewer	Husein Reka
Date	20 January 2024
**Document information:**	
Name	Circular Number 21 of 2020 Full DRG Implementation Updates
Author(s)	Dubai Health Authority
Year of publication	24 Aug 2020
Country/Emirate	Dubai
Link	https://www.isahd.ae/content/docs/GC-21-2020.pdf
Aim/purpose	Updates on full DRG Implementation
Record #	269
**Inclusion/Exclusion Criteria**	
Population	Private Health Insurance in Dubai
Concept	Transformation/Reforms of Dubai Private Health Insurance Scheme
Context	GCC Health System
**Review framework domains studied in literature on private health insurance transformation**	
Health System Framework (e.g. eligibility, sector size, user charges)	
Institutional environment (e.g. legal framework, regulation)	
Private Health Insurance policy framework (e.g. objective, population coverage, system description, types of coverage	
Market structure, conduct and performance (e.g. market concentration, barriers to entry, local vs. international, vertical integration, value-based health care)	Reimbursement reforms
**Key findings related to scoping review question**	
Health System framework	
Eligibility	
Sector size	
User charges	
Institutional environment	

Legal framework	
Material regulation	
Prudential regulation	
Private Health Insurance policy framework	
Objective	
Population coverage	
System description	
Coverage type	
Market structure	
Concentration	
Barriers to entry	
Local vs international	
Vertical Integration	
Value Based Health Care	DHA issues a manual that provides guidance to adjudicate claims correctly and consistently in accordance to standardized adjudication rules. Hospitals and Insurers requested to submit and adjudicate all inpatient claims in accordance to the adjudication standards in the manual respectively

Reviewer	Husein Reka
Date	20 January 2024
**Document information:**	
Name	Circular Number 02 of 2021 Essential Benefit Plan Formulary
Author(s)	Dubai Health Authority
Year of publication	17 Feb 2021
Country/Emirate	Dubai
Link	https://www.isahd.ae/content/docs/GC%2002-2021.pdf
Aim/purpose	Launch and implementation of unified drug formulary
Record #	272
**Inclusion/Exclusion Criteria**	
Population	Private Health Insurance in Dubai
Concept	Transformation/Reforms of Dubai Private Health Insurance Scheme
Context	GCC Health System
**Review framework domains studied in literature on private health insurance transformation**	
Health System Framework (e.g. eligibility, sector size, user charges)	
Institutional environment (e.g. legal framework, regulation)	
Private Health Insurance policy framework (e.g. objective, population coverage, system description, types of coverage	
Market structure, conduct and performance (e.g. market concentration, barriers to entry, local vs. international, vertical integration, value-based health care)	Unified Drug Formulary
**Key findings related to scoping review question**	
Health System framework	
Eligibility	
Sector size	
User charges	
Institutional environment	

Legal framework	
Material regulation	
Prudential regulation	
Private Health Insurance policy framework	
Objective	
Population coverage	
System description	
Coverage type	
Market structure	
Concentration	
Barriers to entry	
Local vs international	
Vertical Integration	
Value Based Health Care	DHA launches unified drug formulary as part of essential benefit package of ISAHD, a comprehensive reference list of outpatient medications based on Dubai Drug Codes (DDC)

Reviewer	Husein Reka
Date	20 January 2024
**Document information:**	
Name	Circular Number 04 of 2021 Day Case Admission Policy
Author(s)	Dubai Health Authority
Year of publication	1 Apr 2021
Country/Emirate	Dubai
Link	https://www.isahd.ae/content/docs/GC%20Day%20Case%20Admission%20General%20Circluar.pdf
Aim/purpose	Introduction of day case admission policy
Record #	274
**Inclusion/Exclusion Criteria**	
Population	Private Health Insurance in Dubai
Concept	Transformation/Reforms of Dubai Private Health Insurance Scheme
Context	GCC Health System
**Review framework domains studied in literature on private health insurance transformation**	
Health System Framework (e.g. eligibility, sector size, user charges)	
Institutional environment (e.g. legal framework, regulation)	
Private Health Insurance policy framework (e.g. objective, population coverage, system description, types of coverage	
Market structure, conduct and performance (e.g. market concentration, barriers to entry, local vs. international, vertical integration, value-based health care)	Reimbursement reforms
**Key findings related to scoping review question**	
Health System framework	
Eligibility	
Sector size	
User charges	
Institutional environment	

Legal framework	
Material regulation	
Prudential regulation	
Private Health Insurance policy framework	
Objective	
Population coverage	
System description	
Coverage type	
Market structure	
Concentration	
Barriers to entry	
Local vs international	
Vertical Integration	
Value Based Health Care	DHA as part of DRG implementation experience noticed a shift of activity from multi-day inpatient admissions to day case admissions due to different payment system (early hour admission and discharge at late hours). Regulator changed definition of multi-day admission cases to up to 12 hours stay and introduced CPT codes of list of procedures

Reviewer	Husein Reka
Date	20 January 2024
**Document information:**	
Name	Circular Number 05 of 2021 Rolling out 3M software new version (IR-DRG v3.3 Codefinder / Grouper) and adding the fixes of current version (IR-DRG v3.01)
Author(s)	Dubai Health Authority
Year of publication	23 May 2021
Country/Emirate	Dubai
Link	https://www.isahd.ae/content/docs/GC%2005-2021.pdf
Aim/purpose	Notification on changes in 3M grouper software
Record #	275
**Inclusion/Exclusion Criteria**	
Population	Private Health Insurance in Dubai
Concept	Transformation/Reforms of Dubai Private Health Insurance Scheme
Context	GCC Health System
**Review framework domains studied in literature on private health insurance transformation**	
Health System Framework (e.g. eligibility, sector size, user charges)	
Institutional environment (e.g. legal framework, regulation)	
Private Health Insurance policy framework (e.g. objective, population coverage, system description, types of coverage	
Market structure, conduct and performance (e.g. market concentration, barriers to entry, local vs. international, vertical integration, value-based health care)	Reimbursement reforms
**Key findings related to scoping review question**	
Health System framework	
Eligibility	
Sector size	
User charges	

Institutional environment	
Legal framework	
Material regulation	
Prudential regulation	
Private Health Insurance policy framework	
Objective	
Population coverage	
System description	
Coverage type	
Market structure	
Concentration	
Barriers to entry	
Local vs international	
Vertical Integration	
Value Based Health Care	DHA notified payers and providers on IR-DRG v3.01 improvements and IR-DRG v3.3 Codefinder / grouper roll out

Reviewer	Husein Reka
Date	20 January 2024
**Document information:**	
Name	Circular Number 06 of 2021 Fund transfer for Hepatitis C & B Patient Support Program
Author(s)	Dubai Health Authority
Year of publication	2 Sep 2021
Country/Emirate	Dubai
Link	https://www.isahd.ae/content/docs/GC%2006-2021%20(1).pdf
Aim/purpose	Notification of allocation of funds to patient support programs
Record #	276
**Inclusion/Exclusion Criteria**	
Population	Private Health Insurance in Dubai
Concept	Transformation/Reforms of Dubai Private Health Insurance Scheme
Context	GCC Health System
**Review framework domains studied in literature on private health insurance transformation**	
Health System Framework (e.g. eligibility, sector size, user charges)	Single pooling funds
Institutional environment (e.g. legal framework, regulation)	
Private Health Insurance policy framework (e.g. objective, population coverage, system description, types of coverage	
Market structure, conduct and performance (e.g. market concentration, barriers to entry, local vs. international, vertical integration, value-based health care)	
**Key findings related to scoping review question**	
Health System framework	
Eligibility	DHA due to lack of compliance with funding of this program by health insurance companies, took over the

	operations of this fund and its expansion to Hepatitis at no additional premium
Sector size	
User charges	
Institutional environment	
Legal framework	
Material regulation	
Prudential regulation	
Private Health Insurance policy framework	
Objective	
Population coverage	
System description	
Coverage type	
Market structure	
Concentration	
Barriers to entry	
Local vs international	
Vertical Integration	
Value Based Health Care	DHA notified payers and providers on IR-DRG v3.01 improvements and IR-DRG v3.3 Codefinder / grouper roll out

Reviewer	Husein Reka
Date	20 January 2024
**Document information:**	
Name	Circular Number 01 of 2022 - Patient Support Program – New workflow process
Author(s)	Dubai Health Authority
Year of publication	19 Jan 2022
Country/Emirate	Dubai
Link	https://www.isahd.ae/content/docs/GC%2001-2022.pdf
Aim/purpose	New workflow process for patient support programs
Record #	278
**Inclusion/Exclusion Criteria**	
Population	Private Health Insurance in Dubai
Concept	Transformation/Reforms of Dubai Private Health Insurance Scheme
Context	GCC Health System
**Review framework domains studied in literature on private health insurance transformation**	
Health System Framework (e.g. eligibility, sector size, user charges)	Single pooling funds
Institutional environment (e.g. legal framework, regulation)	
Private Health Insurance policy framework (e.g. objective, population coverage, system description, types of coverage	
Market structure, conduct and performance (e.g. market concentration, barriers to entry, local vs. international, vertical integration, value-based health care)	
**Key findings related to scoping review question**	
Health System framework	
Eligibility	DHA revealed new workflow process after migrating Basmah Fund from commercial vendor to Dubai Health Insurance Corporation
Sector size	

User charges	
Institutional environment	
Legal framework	
Material regulation	
Prudential regulation	
Private Health Insurance policy framework	
Objective	
Population coverage	
System description	
Coverage type	
Market structure	
Concentration	
Barriers to entry	
Local vs international	
Vertical Integration	
Value Based Health Care	DHA notified payers and providers on IR-DRG v3.01 improvements and IR-DRG v3.3 Codefinder / grouper roll out

Reviewer	Husein Reka
Date	20 January 2024
**Document information:**	
Name	Circular Number 03 of 2022 Full DRG Implementation – Year 2
Author(s)	Dubai Health Authority
Year of publication	18 Mar 2022
Country/Emirate	Dubai
Link	https://www.isahd.ae/content/docs/GC%2003%202022.pdf
Aim/purpose	Full DRG Year 2 implementation effective date
Record #	280
**Inclusion/Exclusion Criteria**	
Population	Private Health Insurance in Dubai
Concept	Transformation/Reforms of Dubai Private Health Insurance Scheme
Context	GCC Health System
**Review framework domains studied in literature on private health insurance transformation**	
Health System Framework (e.g. eligibility, sector size, user charges)	
Institutional environment (e.g. legal framework, regulation)	
Private Health Insurance policy framework (e.g. objective, population coverage, system description, types of coverage	
Market structure, conduct and performance (e.g. market concentration, barriers to entry, local vs. international, vertical integration, value-based health care)	Reimbursement reforms
**Key findings related to scoping review question**	
Health System framework	
Eligibility	
Sector size	
User charges	
Institutional environment	

Legal framework	
Material regulation	
Prudential regulation	
Private Health Insurance policy framework	
Objective	
Population coverage	
System description	
Coverage type	
Market structure	
Concentration	
Barriers to entry	
Local vs international	
Vertical Integration	
Value Based Health Care	DHA notified that Year 2 full DRG implementation will be effective as of 1 April 2022 and published a number of guidelines and rules

Reviewer	Husein Reka
Date	20 January 2024
**Document information:**	
Name	Circular Number 04 of 2022 Excluding Public Hospitals under the Government of Dubai form Emergency Factor 1.
Author(s)	Dubai Health Authority
Year of publication	1 Jul 2022
Country/Emirate	Dubai
Link	https://www.isahd.ae/content/docs/GC%2004%202022.pdf
Aim/purpose	Negotiating factor for non-network emergency cases
Record #	281
**Inclusion/Exclusion Criteria**	
Population	Private Health Insurance in Dubai
Concept	Transformation/Reforms of Dubai Private Health Insurance Scheme
Context	GCC Health System
**Review framework domains studied in literature on private health insurance transformation**	
Health System Framework (e.g. eligibility, sector size, user charges)	
Institutional environment (e.g. legal framework, regulation)	
Private Health Insurance policy framework (e.g. objective, population coverage, system description, types of coverage	
Market structure, conduct and performance (e.g. market concentration, barriers to entry, local vs. international, vertical integration, value-based health care)	Reimbursement reforms
**Key findings related to scoping review question**	
Health System framework	
Eligibility	
Sector size	
User charges	
Institutional environment	

Legal framework	
Material regulation	
Prudential regulation	
Private Health Insurance policy framework	
Objective	
Population coverage	
System description	
Coverage type	
Market structure	
Concentration	
Barriers to entry	
Local vs international	
Vertical Integration	
Value Based Health Care	DHA clarified that negotiation factor 1.3 for non-network emergency cases is not applicable at public hospitals under the Government of Dubai

Reviewer	Husein Reka
Date	20 January 2024
**Document information:**	
Name	Circular Number 05 of 2022 : Prescriptions covered by Health Insurance and Discounts
Author(s)	Dubai Health Authority
Year of publication	16 Aug 2022
Country/Emirate	Dubai
Link	https://www.isahd.ae/content/docs/GC%2005-2022%20-%20Prescriptions%20Covered%20by%20Health%20Insurance%20and%20Discounts.pdf
Aim/purpose	Prohibition of discounts/promotions for drugs covered under health insurance
Record #	282
**Inclusion/Exclusion Criteria**	
Population	Private Health Insurance in Dubai
Concept	Transformation/Reforms of Dubai Private Health Insurance Scheme
Context	GCC Health System
**Review framework domains studied in literature on private health insurance transformation**	
Health System Framework (e.g. eligibility, sector size, user charges)	
Institutional environment (e.g. legal framework, regulation)	
Private Health Insurance policy framework (e.g. objective, population coverage, system description, types of coverage	
Market structure, conduct and performance (e.g. market concentration, barriers to entry, local vs. international, vertical integration, value-based health care)	
**Key findings related to scoping review question**	
Health System framework	
Eligibility	

Sector size	
User charges	
Institutional environment	
Legal framework	
Material regulation	
Prudential regulation	
Private Health Insurance policy framework	
Objective	
Population coverage	
System description	
Coverage type	
Market structure	
Concentration	
Barriers to entry	
Local vs international	
Vertical Integration	
Value Based Health Care	DHA prohibits combination of reimbursable prescription drugs covered under health insurance with promotions and discounts

Reviewer	Husein Reka
Date	20 January 2024
**Document information:**	
Name	Circular Number 06 of 2022 Electronic Claim submissions for Dubai related Policies
Author(s)	Dubai Health Authority
Year of publication	20 Oct 2022
Country/Emirate	Dubai
Link	https://www.isahd.ae/content/docs/GC%2006-2022.pdf
Aim/purpose	Using e-claims portal for claims submission for DHA compliant policies
Record #	283
**Inclusion/Exclusion Criteria**	
Population	Private Health Insurance in Dubai
Concept	Transformation/Reforms of Dubai Private Health Insurance Scheme
Context	GCC Health System
**Review framework domains studied in literature on private health insurance transformation**	
Health System Framework (e.g. eligibility, sector size, user charges)	
Institutional environment (e.g. legal framework, regulation)	Electronic submission of claims
Private Health Insurance policy framework (e.g. objective, population coverage, system description, types of coverage	
Market structure, conduct and performance (e.g. market concentration, barriers to entry, local vs. international, vertical integration, value-based health care)	
**Key findings related to scoping review question**	
Health System framework	
Eligibility	
Sector size	
User charges	

Institutional environment	
Legal framework	
Material regulation	DHA informs that e-claims portal is the only channel for claims submission and any other forms of submission will trigger automatic rejection. A 90 day grace period given to providers inside and outside of Emirate of Dubai to register with the system
Prudential regulation	
Private Health Insurance policy framework	
Objective	
Population coverage	
System description	
Coverage type	
Market structure	
Concentration	
Barriers to entry	
Local vs international	
Vertical Integration	
Value Based Health Care	

Reviewer	Husein Reka
Date	20 January 2024
**Document information:**	
Name	Circular Number 08 of 2022 Updating the data of the insured members in the “E-Claims” portal – Member Register
Author(s)	Dubai Health Authority
Year of publication	17 Oct 2022
Country/Emirate	Dubai
Link	https://www.isahd.ae/content/docs/GC%2008-2022.pdf
Aim/purpose	Using e-claims portal for member registration
Record #	285
**Inclusion/Exclusion Criteria**	
Population	Private Health Insurance in Dubai
Concept	Transformation/Reforms of Dubai Private Health Insurance Scheme
Context	GCC Health System
**Review framework domains studied in literature on private health insurance transformation**	
Health System Framework (e.g. eligibility, sector size, user charges)	
Institutional environment (e.g. legal framework, regulation)	Electronic submission of claims
Private Health Insurance policy framework (e.g. objective, population coverage, system description, types of coverage	
Market structure, conduct and performance (e.g. market concentration, barriers to entry, local vs. international, vertical integration, value-based health care)	
**Key findings related to scoping review question**	
Health System framework	
Eligibility	
Sector size	
User charges	

Institutional environment	
Legal framework	
Material regulation	DHA informed all concerned parties of the health insurance system in the Emirates of Dubai must use the E-claims portal to update the data of the insured members by uploading the data in the “member register”, provided the scope of coverage is for all in force insurance policies & the new insurance policies for issuance at the time of issuance of this circular
Prudential regulation	
Private Health Insurance policy framework	
Objective	
Population coverage	
System description	
Coverage type	
Market structure	
Concentration	
Barriers to entry	
Local vs international	
Vertical Integration	
Value Based Health Care	

Reviewer	Husein Reka
Date	20 January 2024
**Document information:**	
Name	Circular Number 10 of 2022 – DRG Implementation for Eye Hospitals & Eye Centers
Author(s)	Dubai Health Authority
Year of publication	29 Nov 2022
Country/Emirate	Dubai
Link	https://www.isahd.ae/content/docs/GC%2010-2022.pdf
Aim/purpose	Eye hospitals to follow Year 2 full DRG implementation
Record #	287
**Inclusion/Exclusion Criteria**	
Population	Private Health Insurance in Dubai
Concept	Transformation/Reforms of Dubai Private Health Insurance Scheme
Context	GCC Health System
**Review framework domains studied in literature on private health insurance transformation**	
Health System Framework (e.g. eligibility, sector size, user charges)	
Institutional environment (e.g. legal framework, regulation)	
Private Health Insurance policy framework (e.g. objective, population coverage, system description, types of coverage	
Market structure, conduct and performance (e.g. market concentration, barriers to entry, local vs. international, vertical integration, value-based health care)	Reimbursement reforms
**Key findings related to scoping review question**	
Health System framework	
Eligibility	
Sector size	
User charges	
Institutional environment	

Legal framework	
Material regulation	
Prudential regulation	
Private Health Insurance policy framework	
Objective	
Population coverage	
System description	
Coverage type	
Market structure	
Concentration	
Barriers to entry	
Local vs international	
Vertical Integration	
Value Based Health Care	DHA announcing the inclusion of eye hospitals licensed by DHA will follow Year 2 full DRG implementation

Reviewer	Husein Reka
Date	20 January 2024
**Document information:**	
Name	Circular Number 11 of 2022 Expansion of Patient Support Program CoE to private providers
Author(s)	Dubai Health Authority
Year of publication	25 Nov 2022
Country/Emirate	Dubai
Link	https://www.isahd.ae/content/docs/GC%2011-2022.pdf
Aim/purpose	Expansion of PSP to private hospitals
Record #	288
**Inclusion/Exclusion Criteria**	
Population	Private Health Insurance in Dubai
Concept	Transformation/Reforms of Dubai Private Health Insurance Scheme
Context	GCC Health System
**Review framework domains studied in literature on private health insurance transformation**	
Health System Framework (e.g. eligibility, sector size, user charges)	Single pool fund network expansion
Institutional environment (e.g. legal framework, regulation)	
Private Health Insurance policy framework (e.g. objective, population coverage, system description, types of coverage	
Market structure, conduct and performance (e.g. market concentration, barriers to entry, local vs. international, vertical integration, value-based health care)	
**Key findings related to scoping review question**	
Health System framework	
Eligibility	DHA announces the expansion of patient support program to include private providers to the network of PSP
Sector size	
User charges	

Institutional environment	
Legal framework	
Material regulation	
Prudential regulation	
Private Health Insurance policy framework	
Objective	
Population coverage	
System description	
Coverage type	
Market structure	
Concentration	
Barriers to entry	
Local vs international	
Vertical Integration	
Value Based Health Care	

Reviewer	Husein Reka
Date	20 January 2024
**Document information:**	
Name	Circular Number 02 of 2023 Full DRG Implementation – Year 3
Author(s)	Dubai Health Authority
Year of publication	24 May 2023
Country/Emirate	Dubai
Link	https://www.isahd.ae/content/docs/GC%2002-2023.pdf
Aim/purpose	Year 3 full DRG implementation effective date
Record #	290
**Inclusion/Exclusion Criteria**	
Population	Private Health Insurance in Dubai
Concept	Transformation/Reforms of Dubai Private Health Insurance Scheme
Context	GCC Health System
**Review framework domains studied in literature on private health insurance transformation**	
Health System Framework (e.g. eligibility, sector size, user charges)	Single pool fund network expansion
Institutional environment (e.g. legal framework, regulation)	
Private Health Insurance policy framework (e.g. objective, population coverage, system description, types of coverage	
Market structure, conduct and performance (e.g. market concentration, barriers to entry, local vs. international, vertical integration, value-based health care)	Reimbursement reforms
**Key findings related to scoping review question**	
Health System framework	
Eligibility	
Sector size	
User charges	
Institutional environment	

Legal framework	
Material regulation	
Prudential regulation	
Private Health Insurance policy framework	
Objective	
Population coverage	
System description	
Coverage type	
Market structure	
Concentration	
Barriers to entry	
Local vs international	
Vertical Integration	
Value Based Health Care	DHA informed that Year 3 Full DRG Implementation will be effective as of 00:00, Saturday 1 st July 2023 for all DHA licensed hospitals and day care facilities

Reviewer	Husein Reka
Date	20 January 2024
**Document information:**	
Name	Circular Number 03 of 2023 The contractual relationship between the healthcare providers & insurance companies of the health insurance system in the Emirate of Dubai
Author(s)	Dubai Health Authority
Year of publication	1 Jun 2023
Country/Emirate	Dubai
Link	https://www.isahd.ae/content/docs/GC%2003-2023.pdf
Aim/purpose	Regulatiing contractual arrangements between payers and providers
Record #	291
**Inclusion/Exclusion Criteria**	
Population	Private Health Insurance in Dubai
Concept	Transformation/Reforms of Dubai Private Health Insurance Scheme
Context	GCC Health System
**Review framework domains studied in literature on private health insurance transformation**	
Health System Framework (e.g. eligibility, sector size, user charges)	
Institutional environment (e.g. legal framework, regulation)	Standard contracts
Private Health Insurance policy framework (e.g. objective, population coverage, system description, types of coverage	
Market structure, conduct and performance (e.g. market concentration, barriers to entry, local vs. international, vertical integration, value-based health care)	
**Key findings related to scoping review question**	
Health System framework	
Eligibility	
Sector size	

User charges	
Institutional environment	
Legal framework	
Material regulation	DHA requests that contracts should have standard elements and the parties must regulate all aspects and matters related to the contractual agreements between them in a clear and explicit manner, so as to ensure that all data contained in the contract are met with the published requirements
Prudential regulation	
Private Health Insurance policy framework	
Objective	
Population coverage	
System description	
Coverage type	
Market structure	
Concentration	
Barriers to entry	
Local vs international	
Vertical Integration	
Value Based Health Care	

Reviewer	Husein Reka
Date	20 January 2024
**Document information:**	
Name	Circular Number 04 of 2023 – Upgrade of ICD-10-CM, CPT and HCPCS Code sets to version 2021
Author(s)	Dubai Health Authority
Year of publication	6 Jul 2023
Country/Emirate	Dubai
Link	https://www.isahd.ae/content/docs/GC%2004-2023.pdf
Aim/purpose	Upgrade of ICD-10-CM, CPT and HCPCS to version 2021
Record #	292
**Inclusion/Exclusion Criteria**	
Population	Private Health Insurance in Dubai
Concept	Transformation/Reforms of Dubai Private Health Insurance Scheme
Context	GCC Health System
**Review framework domains studied in literature on private health insurance transformation**	
Health System Framework (e.g. eligibility, sector size, user charges)	
Institutional environment (e.g. legal framework, regulation)	
Private Health Insurance policy framework (e.g. objective, population coverage, system description, types of coverage	
Market structure, conduct and performance (e.g. market concentration, barriers to entry, local vs. international, vertical integration, value-based health care)	Reimbursement reform
**Key findings related to scoping review question**	
Health System framework	
Eligibility	
Sector size	
User charges	
Institutional environment	

Legal framework	
Material regulation	
Prudential regulation	
Private Health Insurance policy framework	
Objective	
Population coverage	
System description	
Coverage type	
Market structure	
Concentration	
Barriers to entry	
Local vs international	
Vertical Integration	
Value Based Health Care	DHA announced the upgrade of all ICD-10-CM, CPT and HCPCS from 2018 code sets to 2021 code sets

Reviewer	Husein Reka
Date	20 January 2024
**Document information:**	
Name	Standards Notice Number 1 of 2014 Facilitation agreements between a Participating Insurer (PI) and a non-Participating Insurer (non-PI) in relation to Lower Salary Band (LSB) workers
Author(s)	Dubai Health Authority
Year of publication	10 Nov 2014
Country/Emirate	Dubai
Link	https://www.isahd.ae/content/docs/SN%2001-2014%20Facilitation%20agreements.pdf
Aim/purpose	Specify the essential clauses of any facilitation agreements between PIs and non-PIs in relation to LSB workers
Record #	293
**Inclusion/Exclusion Criteria**	
Population	Private Health Insurance in Dubai
Concept	Transformation/Reforms of Dubai Private Health Insurance Scheme
Context	GCC Health System
**Review framework domains studied in literature on private health insurance transformation**	
Health System Framework (e.g. eligibility, sector size, user charges)	
Institutional environment (e.g. legal framework, regulation)	Material and prudential provisions for insurance companies
Private Health Insurance policy framework (e.g. objective, population coverage, system description, types of coverage	
Market structure, conduct and performance (e.g. market concentration, barriers to entry, local vs. international, vertical integration, value-based health care)	

Key findings related to scoping review question	
Health System framework	
Eligibility	
Sector size	
User charges	
Institutional environment	
Legal framework	
Material regulation	DHA advise to insurance companies who are contemplating establishing facilitation agreements between PIs and non-PIs in relation to the insurance of LSB workers of the expected essential matters to address certain aspects through specific clauses (reinsurance, renumeration, branding, commission, non-competition)
Prudential regulation	DHA advise to insurance companies who are contemplating establishing facilitation agreements between PIs and non-PIs in relation to the insurance of LSB workers of the expected essential matters to address certain aspects through specific clauses (reinsurance, renumeration, branding, commission, non-competition)
Private Health Insurance policy framework	
Objective	
Population coverage	
System description	
Coverage type	
Market structure	
Concentration	
Barriers to entry	
Local vs international	
Vertical Integration	
Value Based Health Care	

Reviewer	Husein Reka
Date	20 January 2024
**Document information:**	
Name	Standards Notice Number 3 of 2015 Health insurance claims records
Author(s)	Dubai Health Authority
Year of publication	9 Jun 2015
Country/Emirate	Dubai
Link	https://www.isahd.ae/content/docs/SN%2003-2015%20Standardised%20claims%20records.pdf
Aim/purpose	Mandate standardised content of health insurance claims records produced by insurance companies
Record #	296
**Inclusion/Exclusion Criteria**	
Population	Private Health Insurance in Dubai
Concept	Transformation/Reforms of Dubai Private Health Insurance Scheme
Context	GCC Health System
**Review framework domains studied in literature on private health insurance transformation**	
Health System Framework (e.g. eligibility, sector size, user charges)	
Institutional environment (e.g. legal framework, regulation)	Standardization of health insurance claims
Private Health Insurance policy framework (e.g. objective, population coverage, system description, types of coverage	
Market structure, conduct and performance (e.g. market concentration, barriers to entry, local vs. international, vertical integration, value-based health care)	
**Key findings related to scoping review question**	
Health System framework	
Eligibility	
Sector size	

User charges	
Institutional environment	
Legal framework	
Material regulation	DHA issued a standard notice on the standards and format of claims records data for compliance by all insurance companies
Prudential regulation	
Private Health Insurance policy framework	
Objective	
Population coverage	
System description	
Coverage type	
Market structure	
Concentration	
Barriers to entry	
Local vs international	
Vertical Integration	
Value Based Health Care	

Reviewer	Husein Reka
Date	20 January 2024
**Document information:**	
Name	Standards Notice Number 4 of 2015 Marketing of health insurance in or into the Emirate of Dubai
Author(s)	Dubai Health Authority
Year of publication	25 Oct 2015
Country/Emirate	Dubai
Link	https://www.isahd.ae/content/docs/SN%2004-2015%20Marketing%20standards.pdf
Aim/purpose	Health insurance marketing rules in the Emirate of Dubai
Record #	297
**Inclusion/Exclusion Criteria**	
Population	Private Health Insurance in Dubai
Concept	Transformation/Reforms of Dubai Private Health Insurance Scheme
Context	GCC Health System
**Review framework domains studied in literature on private health insurance transformation**	
Health System Framework (e.g. eligibility, sector size, user charges)	
Institutional environment (e.g. legal framework, regulation)	Marketing rules for health insurance
Private Health Insurance policy framework (e.g. objective, population coverage, system description, types of coverage	
Market structure, conduct and performance (e.g. market concentration, barriers to entry, local vs. international, vertical integration, value-based health care)	
**Key findings related to scoping review question**	
Health System framework	
Eligibility	
Sector size	
User charges	

Institutional environment	
Legal framework	
Material regulation	DHA issued general rules and definitions on marketing activity related to health insurance in Dubai
Prudential regulation	
Private Health Insurance policy framework	
Objective	
Population coverage	
System description	
Coverage type	
Market structure	
Concentration	
Barriers to entry	
Local vs international	
Vertical Integration	
Value Based Health Care	

Reviewer	Husein Reka
Date	20 January 2024
**Document information:**	
Name	Standards Notice Number 1 of 2017 Intermediary Remuneration on LSB products
Author(s)	Dubai Health Authority
Year of publication	19 Oct 2017
Country/Emirate	Dubai
Link	https://www.isahd.ae/content/docs/SN%2001-2017.pdf
Aim/purpose	New regulation on the maximum allowable remuneration to intermediaries on any products quoted to LSB members
Record #	298
**Inclusion/Exclusion Criteria**	
Population	Private Health Insurance in Dubai
Concept	Transformation/Reforms of Dubai Private Health Insurance Scheme
Context	GCC Health System
**Review framework domains studied in literature on private health insurance transformation**	
Health System Framework (e.g. eligibility, sector size, user charges)	
Institutional environment (e.g. legal framework, regulation)	Intermediary fees regulation
Private Health Insurance policy framework (e.g. objective, population coverage, system description, types of coverage	
Market structure, conduct and performance (e.g. market concentration, barriers to entry, local vs. international, vertical integration, value-based health care)	
**Key findings related to scoping review question**	
Health System framework	
Eligibility	
Sector size	
User charges	

Institutional environment	
Legal framework	
Material regulation	DHA mandated a maximum of 5% commission to intermediaries on products quoted for LSB member
Prudential regulation	
Private Health Insurance policy framework	
Objective	
Population coverage	
System description	
Coverage type	
Market structure	
Concentration	
Barriers to entry	
Local vs international	
Vertical Integration	
Value Based Health Care	

Reviewer	Husein Reka
Date	20 January 2024
**Document information:**	
Name	Standards Notice Number 1 of 2018 Health insurance claims records
Author(s)	Dubai Health Authority
Year of publication	10 Sep 2018
Country/Emirate	Dubai
Link	https://www.isahd.ae/content/docs/SN%2001-2018%20v%201.0%20050918.pdf
Aim/purpose	Advise to insurance companies on the required standard format to be adopted when issuing claims records data
Record #	299
**Inclusion/Exclusion Criteria**	
Population	Private Health Insurance in Dubai
Concept	Transformation/Reforms of Dubai Private Health Insurance Scheme
Context	GCC Health System
**Review framework domains studied in literature on private health insurance transformation**	
Health System Framework (e.g. eligibility, sector size, user charges)	
Institutional environment (e.g. legal framework, regulation)	Claims record data standards
Private Health Insurance policy framework (e.g. objective, population coverage, system description, types of coverage	
Market structure, conduct and performance (e.g. market concentration, barriers to entry, local vs. international, vertical integration, value-based health care)	
**Key findings related to scoping review question**	
Health System framework	
Eligibility	
Sector size	

User charges	
Institutional environment	
Legal framework	
Material regulation	DHA issued a new standard on claims record data replacing earlier issued standards for compliance by 1 October 2018
Prudential regulation	
Private Health Insurance policy framework	
Objective	
Population coverage	
System description	
Coverage type	
Market structure	
Concentration	
Barriers to entry	
Local vs international	
Vertical Integration	
Value Based Health Care	

Reviewer	Husein Reka
Date	20 January 2024
**Document information:**	
Name	Standards Notice Number 2 of 2019 Use of Remittance Advices and Denial codes
Author(s)	Dubai Health Authority
Year of publication	1 Aug 2019
Country/Emirate	Dubai
Link	https://www.isahd.ae/content/docs/SN%2002-2019.pdf
Aim/purpose	Clarify and reiterate payers obligations when posting an RA to ensure compliance and avoid penalties
Record #	301
**Inclusion/Exclusion Criteria**	
Population	Private Health Insurance in Dubai
Concept	Transformation/Reforms of Dubai Private Health Insurance Scheme
Context	GCC Health System
**Review framework domains studied in literature on private health insurance transformation**	
Health System Framework (e.g. eligibility, sector size, user charges)	
Institutional environment (e.g. legal framework, regulation)	Remittance Advice and denials
Private Health Insurance policy framework (e.g. objective, population coverage, system description, types of coverage	
Market structure, conduct and performance (e.g. market concentration, barriers to entry, local vs. international, vertical integration, value-based health care)	
**Key findings related to scoping review question**	
Health System framework	
Eligibility	
Sector size	
User charges	

Institutional environment	
Legal framework	
Material regulation	DHA reiterates the need of compliance with the requirement to post remittance advice and denials code in eClaimLink system
Prudential regulation	
Private Health Insurance policy framework	
Objective	
Population coverage	
System description	
Coverage type	
Market structure	
Concentration	
Barriers to entry	
Local vs international	
Vertical Integration	
Value Based Health Care	

Reviewer	Husein Reka
Date	20 January 2024
**Document information:**	
Name	Standards Notice Number 3 of 2019 Claim Cycle Standardisation
Author(s)	Dubai Health Authority
Year of publication	31 Jul 2019
Country/Emirate	Dubai
Link	https://www.isahd.ae/content/docs/SN%2003-2019.pdf
Aim/purpose	Standardisation of the claim cycle timelines and permissible frequency of submissions
Record #	302
**Inclusion/Exclusion Criteria**	
Population	Private Health Insurance in Dubai
Concept	Transformation/Reforms of Dubai Private Health Insurance Scheme
Context	GCC Health System
**Review framework domains studied in literature on private health insurance transformation**	
Health System Framework (e.g. eligibility, sector size, user charges)	
Institutional environment (e.g. legal framework, regulation)	Claims cycle standardization
Private Health Insurance policy framework (e.g. objective, population coverage, system description, types of coverage	
Market structure, conduct and performance (e.g. market concentration, barriers to entry, local vs. international, vertical integration, value-based health care)	
**Key findings related to scoping review question**	
Health System framework	
Eligibility	
Sector size	
User charges	

Institutional environment	
Legal framework	
Material regulation	DHA issued standards and definitions on Submission, Resubmission, Remittance and Payment Timelines
Prudential regulation	
Private Health Insurance policy framework	
Objective	
Population coverage	
System description	
Coverage type	
Market structure	
Concentration	
Barriers to entry	
Local vs international	
Vertical Integration	
Value Based Health Care	

Reviewer	Husein Reka
Date	20 January 2024
**Document information:**	
Name	Standards Notice Number 4 of 2019 Member Register
Author(s)	Dubai Health Authority
Year of publication	4 Aug 2019
Country/Emirate	Dubai
Link	https://www.isahd.ae/content/docs/SN%2004-2019%2004082019.pdf
Aim/purpose	Mandate the updated structure of the member register
Record #	303
**Inclusion/Exclusion Criteria**	
Population	Private Health Insurance in Dubai
Concept	Transformation/Reforms of Dubai Private Health Insurance Scheme
Context	GCC Health System
**Review framework domains studied in literature on private health insurance transformation**	
Health System Framework (e.g. eligibility, sector size, user charges)	
Institutional environment (e.g. legal framework, regulation)	Member register standardization
Private Health Insurance policy framework (e.g. objective, population coverage, system description, types of coverage	
Market structure, conduct and performance (e.g. market concentration, barriers to entry, local vs. international, vertical integration, value-based health care)	
**Key findings related to scoping review question**	
Health System framework	
Eligibility	
Sector size	
User charges	
Institutional environment	

Legal framework	
Material regulation	DHA issued notice to market that new format will be in use as of October 1st 2019 to be upload as per attached format
Prudential regulation	
Private Health Insurance policy framework	
Objective	
Population coverage	
System description	
Coverage type	
Market structure	
Concentration	
Barriers to entry	
Local vs international	
Vertical Integration	
Value Based Health Care	

Reviewer	Husein Reka
Date	20 January 2024
**Document information:**	
Name	Procedural Notice Number 1 of 2014 Participating Insurer status submissions for 2015
Author(s)	Dubai Health Authority
Year of publication	27 Oct 2014
Country/Emirate	Dubai
Link	https://www.isahd.ae/content/docs/PN%2001-2014%20PI%202015%20submissions.pdf
Aim/purpose	Information and detail on the requirements for application, the process of applying and relevant dates
Record #	304
**Inclusion/Exclusion Criteria**	
Population	Private Health Insurance in Dubai
Concept	Transformation/Reforms of Dubai Private Health Insurance Scheme
Context	GCC Health System
**Review framework domains studied in literature on private health insurance transformation**	
Health System Framework (e.g. eligibility, sector size, user charges)	
Institutional environment (e.g. legal framework, regulation)	Payer qualifications
Private Health Insurance policy framework (e.g. objective, population coverage, system description, types of coverage	
Market structure, conduct and performance (e.g. market concentration, barriers to entry, local vs. international, vertical integration, value-based health care)	
**Key findings related to scoping review question**	
Health System framework	
Eligibility	
Sector size	

User charges	
Institutional environment	
Legal framework	
Material regulation	DHA issued notification to all interested parties on the procedure for an application for PI status for the calendar year 2015
Prudential regulation	
Private Health Insurance policy framework	
Objective	
Population coverage	
System description	
Coverage type	
Market structure	
Concentration	
Barriers to entry	
Local vs international	
Vertical Integration	
Value Based Health Care	

Reviewer	Husein Reka
Date	20 January 2024
**Document information:**	
Name	Procedural Notice Number 3 of 2014 Price Regulation for Healthcare Services in Dubai
Author(s)	Dubai Health Authority
Year of publication	6 Nov 2014
Country/Emirate	Dubai
Link	https://www.isahd.ae/content/docs/PN%2003-2014%20Provider%20price%20regulation.pdf
Aim/purpose	Requirements of an application, the process of applying and relevant timelines for price increase in 2015
Record #	305
**Inclusion/Exclusion Criteria**	
Population	Private Health Insurance in Dubai
Concept	Transformation/Reforms of Dubai Private Health Insurance Scheme
Context	GCC Health System
**Review framework domains studied in literature on private health insurance transformation**	
Health System Framework (e.g. eligibility, sector size, user charges)	
Institutional environment (e.g. legal framework, regulation)	
Private Health Insurance policy framework (e.g. objective, population coverage, system description, types of coverage	
Market structure, conduct and performance (e.g. market concentration, barriers to entry, local vs. international, vertical integration, value-based health care)	Price regulation
**Key findings related to scoping review question**	
Health System framework	
Eligibility	
Sector size	

User charges	
Institutional environment	
Legal framework	
Material regulation	
Prudential regulation	
Private Health Insurance policy framework	
Objective	
Population coverage	
System description	
Coverage type	
Market structure	
Concentration	
Barriers to entry	
Local vs international	
Vertical Integration	
Value Based Health Care	DHA issued a procedural notice to notify all interested parties of the procedure to be followed to increase prices for healthcare services and the dates by which submissions must be made

Reviewer	Husein Reka
Date	20 January 2024
**Document information:**	
Name	Procedural Notice Number 2 of 2014 Certification that employers and other sponsors hold health insurance policies
Author(s)	Dubai Health Authority
Year of publication	27 Nov 2014
Country/Emirate	Dubai
Link	https://www.isahd.ae/content/docs/PN%2002-2014%20Health%20insurance%20certificates.pdf
Aim/purpose	Details on the procedure to be followed by insurers to issue a “Certificate of Health Insurance” (CHI)
Record #	306
**Inclusion/Exclusion Criteria**	
Population	Private Health Insurance in Dubai
Concept	Transformation/Reforms of Dubai Private Health Insurance Scheme
Context	GCC Health System
**Review framework domains studied in literature on private health insurance transformation**	
Health System Framework (e.g. eligibility, sector size, user charges)	Proof of insurance coverage
Institutional environment (e.g. legal framework, regulation)	
Private Health Insurance policy framework (e.g. objective, population coverage, system description, types of coverage	
Market structure, conduct and performance (e.g. market concentration, barriers to entry, local vs. international, vertical integration, value-based health care)	
**Key findings related to scoping review question**	
Health System framework	

Eligibility	DHA issued a notice detailing format and procedures of issuing Certificate of Health Insurance
Sector size	
User charges	
Institutional environment	
Legal framework	
Material regulation	
Prudential regulation	
Private Health Insurance policy framework	
Objective	
Population coverage	
System description	
Coverage type	
Market structure	
Concentration	
Barriers to entry	
Local vs international	
Vertical Integration	
Value Based Health Care	DHA issued a procedural notice to notify all interested parties of the procedure to be followed to increase prices for healthcare services and the dates by which submissions must be made

Reviewer	Husein Reka
Date	20 January 2024
**Document information:**	
Name	Procedural Notice Number 4 of 2014 Participating Insurer (PI) status renewal submissions for 2015
Author(s)	Dubai Health Authority
Year of publication	21 Nov 2014
Country/Emirate	Dubai
Link	https://www.isahd.ae/content/docs/PN%2004-2014%20PI%20renewal%20for%202015.pdf
Aim/purpose	Notify all PIs of the procedure to renew PI status for the calendar year 2015
Record #	307
**Inclusion/Exclusion Criteria**	
Population	Private Health Insurance in Dubai
Concept	Transformation/Reforms of Dubai Private Health Insurance Scheme
Context	GCC Health System
**Review framework domains studied in literature on private health insurance transformation**	
Health System Framework (e.g. eligibility, sector size, user charges)	
Institutional environment (e.g. legal framework, regulation)	Payer qualification
Private Health Insurance policy framework (e.g. objective, population coverage, system description, types of coverage	
Market structure, conduct and performance (e.g. market concentration, barriers to entry, local vs. international, vertical integration, value-based health care)	
**Key findings related to scoping review question**	
Health System framework	
Eligibility	
Sector size	

User charges	
Institutional environment	
Legal framework	
Material regulation	DHA issued a notice to advise the procedure to be followed by all companies who act as intermediaries between an insurer and a client in the marketing or selling of health insurance plans to clients in the Emirate of Dubai in order to apply for the Dubai Health Insurance Intermediary Permit (HIIP)
Prudential regulation	
Private Health Insurance policy framework	
Objective	
Population coverage	
System description	
Coverage type	
Market structure	
Concentration	
Barriers to entry	
Local vs international	
Vertical Integration	
Value Based Health Care	

Reviewer	Husein Reka
Date	20 January 2024
**Document information:**	
Name	Procedural Notice Number 5 of 2014 Registration of health insurance intermediaries and their representatives
Author(s)	Dubai Health Authority
Year of publication	30 Nov 2014
Country/Emirate	Dubai
Link	https://www.isahd.ae/content/docs/PN%2005-2014%20Registration%20of%20health%20insurance%20intermediaries%20and%20their%20representatives.pdf
Aim/purpose	Announce the opening of the online application for the Dubai Health Insurance Intermediary Permit (HIIP)
Record #	308
**Inclusion/Exclusion Criteria**	
Population	Private Health Insurance in Dubai
Concept	Transformation/Reforms of Dubai Private Health Insurance Scheme
Context	GCC Health System
**Review framework domains studied in literature on private health insurance transformation**	
Health System Framework (e.g. eligibility, sector size, user charges)	
Institutional environment (e.g. legal framework, regulation)	Intermediary qualification
Private Health Insurance policy framework (e.g. objective, population coverage, system description, types of coverage	
Market structure, conduct and performance (e.g. market concentration, barriers to entry, local vs. international, vertical integration, value-based health care)	
**Key findings related to scoping review question**	
Health System framework	
Eligibility	

Sector size	
User charges	
Institutional environment	
Legal framework	
Material regulation	DHA issued a notice to advise the procedure to be followed by all companies who act as intermediaries between an insurer and a client in the marketing or selling of health insurance plans to clients in the Emirate of Dubai in order to apply for the Dubai Health Insurance Intermediary Permit (HIIP)
Prudential regulation	
Private Health Insurance policy framework	
Objective	
Population coverage	
System description	
Coverage type	
Market structure	
Concentration	
Barriers to entry	
Local vs international	
Vertical Integration	
Value Based Health Care	

Reviewer	Husein Reka
Date	20 January 2024
**Document information:**	
Name	Procedural Notice Number 2 of 2015 Fraud, waste and abuse (FWA) in health insurance
Author(s)	Dubai Health Authority
Year of publication	10 Jun 2015
Country/Emirate	Dubai
Link	https://www.isahd.ae/content/docs/PN%2002-2015%20v1.4%20Fraud,%20waste%20and%20abuse%20return.pdf
Aim/purpose	Requirements to make returns in relation to the identification, reduction and eradication of fraud, waste and abuse
Record #	310
**Inclusion/Exclusion Criteria**	
Population	Private Health Insurance in Dubai
Concept	Transformation/Reforms of Dubai Private Health Insurance Scheme
Context	GCC Health System
**Review framework domains studied in literature on private health insurance transformation**	
Health System Framework (e.g. eligibility, sector size, user charges)	
Institutional environment (e.g. legal framework, regulation)	
Private Health Insurance policy framework (e.g. objective, population coverage, system description, types of coverage	
Market structure, conduct and performance (e.g. market concentration, barriers to entry, local vs. international, vertical integration, value-based health care)	Fraud waste and abuse
**Key findings related to scoping review question**	
Health System framework	

Eligibility	
Sector size	
User charges	
Institutional environment	
Legal framework	
Material regulation	
Prudential regulation	
Private Health Insurance policy framework	
Objective	
Population coverage	
System description	
Coverage type	
Market structure	
Concentration	
Barriers to entry	
Local vs international	
Vertical Integration	
Value Based Health Care	DHA notified all insurers and health insurance claims management companies holding Health Insurance Permits granted by Health Funding Department (HFD) of Dubai Health Authority (DHA) on the requirements to report FWA return and share investigation logs

Reviewer	Husein Reka
Date	20 January 2024
**Document information:**	
Name	Procedural Notice Number 3 of 2015 Payment Model for Healthcare Services in Dubai - 2016
Author(s)	Dubai Health Authority
Year of publication	1 Nov 2015
Country/Emirate	Dubai
Link	https://www.isahd.ae/content/docs/PN%2003-2015%20Price%20Regulation.pdf
Aim/purpose	Specify characteristics of the interim price regulation model and the criteria which healthcare providers will be required to fulfill in order to apply for change in their respective price lists for 2016
Record #	311
**Inclusion/Exclusion Criteria**	
Population	Private Health Insurance in Dubai
Concept	Transformation/Reforms of Dubai Private Health Insurance Scheme
Context	GCC Health System
**Review framework domains studied in literature on private health insurance transformation**	
Health System Framework (e.g. eligibility, sector size, user charges)	
Institutional environment (e.g. legal framework, regulation)	Pricing health care services
Private Health Insurance policy framework (e.g. objective, population coverage, system description, types of coverage	
Market structure, conduct and performance (e.g. market concentration, barriers to entry, local vs. international, vertical integration, value-based health care)	
**Key findings related to scoping review question**	
Health System framework	

Eligibility	
Sector size	
User charges	
Institutional environment	
Legal framework	
Material regulation	DHA notified all interested parties on the procedure to be followed to increase prices for healthcare services, and the dates and deadlines
Prudential regulation	
Private Health Insurance policy framework	
Objective	
Population coverage	
System description	
Coverage type	
Market structure	
Concentration	
Barriers to entry	
Local vs international	
Vertical Integration	
Value Based Health Care	

Reviewer	Husein Reka
Date	20 January 2024
**Document information:**	
Name	Procedural Notice Number 1 of 2019 Patient Support Programs (BASMAH & HCV)
Author(s)	Dubai Health Authority
Year of publication	16 Jan 2019
Country/Emirate	Dubai
Link	https://www.isahd.ae/content/docs/PN%2001-2019.pdf
Aim/purpose	Advise on the requirement of amounts to be collected and transferred regarding the patient support programs
Record #	318
**Inclusion/Exclusion Criteria**	
Population	Private Health Insurance in Dubai
Concept	Transformation/Reforms of Dubai Private Health Insurance Scheme
Context	GCC Health System
**Review framework domains studied in literature on private health insurance transformation**	
Health System Framework (e.g. eligibility, sector size, user charges)	Pooling of funds
Institutional environment (e.g. legal framework, regulation)	
Private Health Insurance policy framework (e.g. objective, population coverage, system description, types of coverage	
Market structure, conduct and performance (e.g. market concentration, barriers to entry, local vs. international, vertical integration, value-based health care)	
**Key findings related to scoping review question**	
Health System framework	
Eligibility	DHA issued a circular clarifying allocation amounts and the intended uses and timeline for transfer of funds for BASMAH account and transfer requirements

Sector size	
User charges	
Institutional environment	
Legal framework	
Material regulation	DHA notified all interested parties on the procedure to be followed to increase prices for healthcare services, and the dates and deadlines
Prudential regulation	
Private Health Insurance policy framework	
Objective	
Population coverage	
System description	
Coverage type	
Market structure	
Concentration	
Barriers to entry	
Local vs international	
Vertical Integration	
Value Based Health Care	

Reviewer	Husein Reka
Date	20 January 2024
**Document information:**	
Name	Procedural Notice Number 2 of 2019 Clarification of Index Rate for 2019
Author(s)	Dubai Health Authority
Year of publication	24 Jan 2019
Country/Emirate	Dubai
Link	https://www.isahd.ae/content/docs/PN%2002-2019.pdf
Aim/purpose	Advise on the ndex rate for 2018
Record #	319
**Inclusion/Exclusion Criteria**	
Population	Private Health Insurance in Dubai
Concept	Transformation/Reforms of Dubai Private Health Insurance Scheme
Context	GCC Health System
**Review framework domains studied in literature on private health insurance transformation**	
Health System Framework (e.g. eligibility, sector size, user charges)	Pooling of funds
Institutional environment (e.g. legal framework, regulation)	
Private Health Insurance policy framework (e.g. objective, population coverage, system description, types of coverage	
Market structure, conduct and performance (e.g. market concentration, barriers to entry, local vs. international, vertical integration, value-based health care)	
**Key findings related to scoping review question**	
Health System framework	
Eligibility	DHA clarifies confusion on the index rate for 2019 and the impact of the Patient support programs (BASMAH & HCV) on the index rate, that effective January 1st 2019 the index rates for PIs is their existing submitted index rate in

	the year of 2018 in addition to AED 37 + VAT for the support programs
Sector size	
User charges	
Institutional environment	
Legal framework	
Material regulation	
Prudential regulation	
Private Health Insurance policy framework	
Objective	
Population coverage	
System description	
Coverage type	
Market structure	
Concentration	
Barriers to entry	
Local vs international	
Vertical Integration	
Value Based Health Care	

Reviewer	Husein Reka
Date	20 January 2024
**Document information:**	
Name	Procedural Notice Number 3 of 2019 BASMAH Fund Transfer
Author(s)	Dubai Health Authority
Year of publication	14 Feb 2019
Country/Emirate	Dubai
Link	https://www.isahd.ae/content/docs/PN%2003-2019.pdf
Aim/purpose	Advise on VAT treatment of BASMAH fund transfer
Record #	320
**Inclusion/Exclusion Criteria**	
Population	Private Health Insurance in Dubai
Concept	Transformation/Reforms of Dubai Private Health Insurance Scheme
Context	GCC Health System
**Review framework domains studied in literature on private health insurance transformation**	
Health System Framework (e.g. eligibility, sector size, user charges)	Pooling of funds
Institutional environment (e.g. legal framework, regulation)	
Private Health Insurance policy framework (e.g. objective, population coverage, system description, types of coverage	
Market structure, conduct and performance (e.g. market concentration, barriers to entry, local vs. international, vertical integration, value-based health care)	
**Key findings related to scoping review question**	
Health System framework	
Eligibility	DHA clarifies that all PIs and HIPs that BASMAH fund transfers to the fund account should be transferred without VAT until further notice. This does not mean VAT should not be collected; however only the AED 19 per insured

	member excluding VAT should be transferred the year of 2018 in addition to AED 37 + VAT for the support programs
Sector size	
User charges	
Institutional environment	
Legal framework	
Material regulation	
Prudential regulation	
Private Health Insurance policy framework	
Objective	
Population coverage	
System description	
Coverage type	
Market structure	
Concentration	
Barriers to entry	
Local vs international	
Vertical Integration	
Value Based Health Care	

Reviewer	Husein Reka
Date	20 January 2024
**Document information:**	
Name	Procedural Notice Number 1 of 2020 Claim Cycle Standardization
Author(s)	Dubai Health Authority
Year of publication	14 Feb 2019
Country/Emirate	Dubai
Link	https://www.isahd.ae/content/docs/PN%2001-2020%20.pdf
Aim/purpose	Procedural Notice on Claim Cycle Standardization
Record #	322
**Inclusion/Exclusion Criteria**	
Population	Private Health Insurance in Dubai
Concept	Transformation/Reforms of Dubai Private Health Insurance Scheme
Context	GCC Health System
**Review framework domains studied in literature on private health insurance transformation**	
Health System Framework (e.g. eligibility, sector size, user charges)	
Institutional environment (e.g. legal framework, regulation)	Claims cycle standardization
Private Health Insurance policy framework (e.g. objective, population coverage, system description, types of coverage	
Market structure, conduct and performance (e.g. market concentration, barriers to entry, local vs. international, vertical integration, value-based health care)	
**Key findings related to scoping review question**	
Health System framework	
Eligibility	
Sector size	
User charges	

Institutional environment	
Legal framework	
Material regulation	DHA clarifies notifies all payers and providers, that further to the timelines stated in Standards Notice 03 of 2019, providers are granted (if required) to have an additional 30 days to submit the initial claim only
Prudential regulation	
Private Health Insurance policy framework	
Objective	
Population coverage	
System description	
Coverage type	
Market structure	
Concentration	
Barriers to entry	
Local vs international	
Vertical Integration	
Value Based Health Care	

Reviewer	Husein Reka
Date	20 January 2024
**Document information:**	
Name	General Circular Number 3 of 2015 Registration and regulation of health insurance intermediaries
Author(s)	Dubai Health Authority
Year of publication	16 Apr 2015
Country/Emirate	Dubai
Link	https://www.isahd.ae/content/docs/GC%2003-2015%20Registration%20of%20health%20insurance%20intermediaries%20(rev%201).pdf
Aim/purpose	Notification on awarded “Conditional Compliance” “Prohibited” or “Suspended” status from transacting health insurance
Record #	331
**Inclusion/Exclusion Criteria**	
Population	Private Health Insurance in Dubai
Concept	Transformation/Reforms of Dubai Private Health Insurance Scheme
Context	GCC Health System
**Review framework domains studied in literature on private health insurance transformation**	
Health System Framework (e.g. eligibility, sector size, user charges)	
Institutional environment (e.g. legal framework, regulation)	Intermediaries’ qualification
Private Health Insurance policy framework (e.g. objective, population coverage, system description, types of coverage	
Market structure, conduct and performance (e.g. market concentration, barriers to entry, local vs. international, vertical integration, value-based health care)	
**Key findings related to scoping review question**	
Health System framework	

Eligibility	
Sector size	
User charges	
Institutional environment	
Legal framework	
Material regulation	DHA notifies all entities participating in the health insurance market within the Emirate of Dubai of the current status of intermediaries who are or may wish to intermediate in health insurance business within the Emirate
Prudential regulation	
Private Health Insurance policy framework	
Objective	
Population coverage	
System description	
Coverage type	
Market structure	
Concentration	
Barriers to entry	
Local vs international	
Vertical Integration	
Value Based Health Care	

Reviewer	Husein Reka
Date	20 January 2024
**Document information:**	
Name	Policy Directive Number 1 of 2015 Complaints handling
Author(s)	Dubai Health Authority
Year of publication	15 Jul 2015
Country/Emirate	Dubai
Link	https://www.isahd.ae/content/docs/PD%2001-2014%20Complaints%20handling.pdf
Aim/purpose	To specify the complaints minimum requirements and standards
Record #	333
**Inclusion/Exclusion Criteria**	
Population	Private Health Insurance in Dubai
Concept	Transformation/Reforms of Dubai Private Health Insurance Scheme
Context	GCC Health System
**Review framework domains studied in literature on private health insurance transformation**	
Health System Framework (e.g. eligibility, sector size, user charges)	
Institutional environment (e.g. legal framework, regulation)	Complaint standards
Private Health Insurance policy framework (e.g. objective, population coverage, system description, types of coverage	
Market structure, conduct and performance (e.g. market concentration, barriers to entry, local vs. international, vertical integration, value-based health care)	
**Key findings related to scoping review question**	
Health System framework	
Eligibility	
Sector size	
User charges	

Institutional environment	
Legal framework	
Material regulation	DHA issues complaints definition and standards and procedures
Prudential regulation	
Private Health Insurance policy framework	
Objective	
Population coverage	
System description	
Coverage type	
Market structure	
Concentration	
Barriers to entry	
Local vs international	
Vertical Integration	
Value Based Health Care	

Reviewer	Husein Reka
Date	20 January 2024
**Document information:**	
Name	Policy Directive Number 4 of 2018 Pregnancy Declarations
Author(s)	Dubai Health Authority
Year of publication	10 Sep 2018
Country/Emirate	Dubai
Link	https://www.isahd.ae/content/docs/PD%2004-2018%20v1.2.pdf
Aim/purpose	To specify the new required disclosures on pregnancy to be captured in where policies include medical declarations
Record #	334
**Inclusion/Exclusion Criteria**	
Population	Private Health Insurance in Dubai
Concept	Transformation/Reforms of Dubai Private Health Insurance Scheme
Context	GCC Health System
**Review framework domains studied in literature on private health insurance transformation**	
Health System Framework (e.g. eligibility, sector size, user charges)	Medical declaration for pregnancy
Institutional environment (e.g. legal framework, regulation)	
Private Health Insurance policy framework (e.g. objective, population coverage, system description, types of coverage	
Market structure, conduct and performance (e.g. market concentration, barriers to entry, local vs. international, vertical integration, value-based health care)	
**Key findings related to scoping review question**	
Health System framework	
Eligibility	DHA issued policy directive to inform HIPs of the new required information on the medical declaration forms,

	timelines for implementation, enforcement and treatment of undeclared pregnancies
Sector size	
User charges	
Institutional environment	
Legal framework	
Material regulation	DHA issues complaints definition and standards and procedures
Prudential regulation	
Private Health Insurance policy framework	
Objective	
Population coverage	
System description	
Coverage type	
Market structure	
Concentration	
Barriers to entry	
Local vs international	
Vertical Integration	
Value Based Health Care	

Reviewer	Husein Reka
Date	20 January 2024
**Document information:**	
Name	Procedural Directive Number 2 of 2019 New Born additions
Author(s)	Dubai Health Authority
Year of publication	30 Apr 2019
Country/Emirate	Dubai
Link	https://www.isahd.ae/content/docs/PD%2002-2019%20New%20Born%20Additions.pdf
Aim/purpose	To advise on the treatment of new born additions
Record #	335
**Inclusion/Exclusion Criteria**	
Population	Private Health Insurance in Dubai
Concept	Transformation/Reforms of Dubai Private Health Insurance Scheme
Context	GCC Health System
**Review framework domains studied in literature on private health insurance transformation**	
Health System Framework (e.g. eligibility, sector size, user charges)	New-born coverage
Institutional environment (e.g. legal framework, regulation)	
Private Health Insurance policy framework (e.g. objective, population coverage, system description, types of coverage	
Market structure, conduct and performance (e.g. market concentration, barriers to entry, local vs. international, vertical integration, value-based health care)	
**Key findings related to scoping review question**	
Health System framework	
Eligibility	DHA issued policy directive that no insurer may impose waiting periods of any kind on new borns, whether they are waiting periods against pre-existing conditions or any

	other conditions, and must be covered under the mother's policy for 30 days and/or up to the mother's annual limit
Sector size	
User charges	
Institutional environment	
Legal framework	
Material regulation	DHA issues complaints definition and standards and procedures
Prudential regulation	
Private Health Insurance policy framework	
Objective	
Population coverage	
System description	
Coverage type	
Market structure	
Concentration	
Barriers to entry	
Local vs international	
Vertical Integration	
Value Based Health Care	

Reviewer	Husein Reka
Date	20 January 2024
**Document information:**	
Name	Policy Directive DHA/DHI/HE/PP/006<br>Maternity Coverage and New Born Additions
Author(s)	Dubai Health Authority
Year of publication	24 Nov 2020
Country/Emirate	Dubai
Link	https://www.isahd.ae/content/docs/PD%20Maternity%20Coverage%20and%20New%20Born%20Additions_24-11-2020.pdf
Aim/purpose	To clarify the maternity coverage to be provided under the mandatory medical insurance policy
Record #	336
**Inclusion/Exclusion Criteria**	
Population	Private Health Insurance in Dubai
Concept	Transformation/Reforms of Dubai Private Health Insurance Scheme
Context	GCC Health System
**Review framework domains studied in literature on private health insurance transformation**	
Health System Framework (e.g. eligibility, sector size, user charges)	Maternity and new born coverage
Institutional environment (e.g. legal framework, regulation)	
Private Health Insurance policy framework (e.g. objective, population coverage, system description, types of coverage	
Market structure, conduct and performance (e.g. market concentration, barriers to entry, local vs. international, vertical integration, value-based health care)	
**Key findings related to scoping review question**	
Health System framework	

Eligibility	DHA advises that all females married at time of enrolment and of child bearing capacity must be covered at least to the extent of the limits specified in the EBP ToB during the term of the policy
Sector size	
User charges	
Institutional environment	
Legal framework	
Material regulation	
Prudential regulation	
Private Health Insurance policy framework	
Objective	
Population coverage	
System description	
Coverage type	
Market structure	
Concentration	
Barriers to entry	
Local vs international	
Vertical Integration	
Value Based Health Care	

Reviewer	Husein Reka
Date	20 January 2024
**Document information:**	
Name	Federal Law No.6 of 2007 On the establishment of the Insurance Authority and Regulation of insurance operations
Author(s)	UAE President
Year of publication	15 Feb 2007
Country/Emirate	United Arab Emirates
Link	https://elaws.moj.gov.ae/mainfrominfoheader.aspx?val=EL1&anchor=Anchor7&np=&lmp=undefined&f=UAE-LC-En_2007-02-05_00006_Kait&fp=%D8%AA%D8%B4%D8%B1%D9%8A%D8%B9+%D8%A7%D9%84%D8%A7%D9%85%D8%A7%D8%B1%D8%A7%D8%AA+%D8%A7%D9%84%D8%B9%D8%B1%D8%A8%D9%8A%D8%A9+%D8%A7%D9%84%D9%85%D8%AA%D8%AD%D8%AF%D8%A9+%D8%A8%D8%A7%D9%84%D8%A7%D9%86%D9%83%D9%84%D9%8A%D8%B2%D9%8A%D8%A9+%2F+INSURANCE&lawRef=S-EL1_2_645&op=UAE-MOJ_LC-En%5c00_INSURANCE%5cUAE-LC-En_2007-02-05_00006_Kait.html%23Anchor7&Words=insurance
Aim/purpose	Insurance Authority establishment law
Record #	340
**Inclusion/Exclusion Criteria**	
Population	Private Health Insurance in UAE
Concept	Transformation/Reforms of UAE Private Health Insurance Scheme
Context	GCC Health System
**Review framework domains studied in literature on private health insurance transformation**	
Health System Framework (e.g. eligibility, sector size, user charges)	

Institutional environment (e.g. legal framework, regulation)	Law introducing dedicated insurance regulaotr
Private Health Insurance policy framework (e.g. objective, population coverage, system description, types of coverage	
Market structure, conduct and performance (e.g. market concentration, barriers to entry, local vs. international, vertical integration, value-based health care)	
**Key findings related to scoping review question**	
Health System framework	
Eligibility	
Sector size	
User charges	
Institutional environment	
Legal framework	UAE President issues Federal Law establishing a dedicated insurance regulator for UAE
Material regulation	
Prudential regulation	
Private Health Insurance policy framework	
Objective	
Population coverage	
System description	
Coverage type	
Market structure	
Concentration	
Barriers to entry	
Local vs international	
Vertical Integration	
Value Based Health Care	

Reviewer	Husein Reka
Date	20 January 2024
**Document information:**	
Name	Cooperative Health Insurance Law
Author(s)	Council of Ministers
Year of publication	18 Apr 1999
Country/Emirate	Saudi Arabia
Link	https://laws.boe.gov.sa/BoeLaws/Laws/LawDetails/87c1482d-fa95-41ea-98b6-a9a700f2a529/2
Aim/purpose	Establishment of a mandatory health insurance scheme
Record #	356
**Inclusion/Exclusion Criteria**	
Population	Private Health Insurance in Saudi Arabia
Concept	Transformation/Reforms of Saudi Arabia Private Health Insurance Scheme
Context	GCC Health System
**Review framework domains studied in literature on private health insurance transformation**	
Health System Framework (e.g. eligibility, sector size, user charges)	
Institutional environment (e.g. legal framework, regulation)	Law introducing mandatory health insurance scheme
Private Health Insurance policy framework (e.g. objective, population coverage, system description, types of coverage	
Market structure, conduct and performance (e.g. market concentration, barriers to entry, local vs. international, vertical integration, value-based health care)	
**Key findings related to scoping review question**	
Health System framework	
Eligibility	
Sector size	
User charges	
Institutional environment	

Legal framework	Royal Decree No. M/10 of 1999 establishes the Cooperative Health Insurance Scheme (CHIS)
Material regulation	
Prudential regulation	
Private Health Insurance policy framework	
Objective	
Population coverage	
System description	
Coverage type	
Market structure	
Concentration	
Barriers to entry	
Local vs international	
Vertical Integration	
Value Based Health Care	

Reviewer	Husein Reka
Date	20 January 2024
**Document information:**	
Name	INTRODUCING PATIENT CLASSIFICATION SYSTEMS IN SAUDI HEALTH INSURANCE MARKET White Paper on Diagnoses Related Groups Classification System in CHI
Author(s)	Council of Health Insurance
Year of publication	1 Oct 2022
Country/Emirate	Saudi Arabia
Link	https://chi.gov.sa/en/knowledge-center/researchlibrary/DRG%20White%20Paper..%2010%20Oct.pdf
Aim/purpose	White paper to market on AR-DRG implementation in private health sector
Record #	369
**Inclusion/Exclusion Criteria**	
Population	Private Health Insurance in Saudi Arabia
Concept	Transformation/Reforms of Saudi Arabia Private Health Insurance Scheme
Context	GCC Health System
**Review framework domains studied in literature on private health insurance transformation**	
Health System Framework (e.g. eligibility, sector size, user charges)	
Institutional environment (e.g. legal framework, regulation)	
Private Health Insurance policy framework (e.g. objective, population coverage, system description, types of coverage	
Market structure, conduct and performance (e.g. market concentration, barriers to entry, local vs. international, vertical integration, value-based health care)	Reimbursement reforms
**Key findings related to scoping review question**	

Health System framework	
Eligibility	
Sector size	
User charges	
Institutional environment	
Legal framework	
Material regulation	
Prudential regulation	
Private Health Insurance policy framework	
Objective	
Population coverage	
System description	
Coverage type	
Market structure	
Concentration	
Barriers to entry	
Local vs international	
Vertical Integration	
Value Based Health Care	Council of Health Insurance document outlining sector strategy on introducing AR-DRG as a patient classification system with phases and implementation timelines for the market

Reviewer	Husein Reka
Date	20 January 2024
**Document information:**	
Name	Improving Health In Saudi Arabia through Population Health Management
Author(s)	Council of Health Insurance
Year of publication	1 Dec 2021
Country/Emirate	Saudi Arabia
Link	https://chi.gov.sa/en/knowledge-center/researchlibrary/CCHI%20Population%20Health%20WP.pdf
Aim/purpose	White paper to market on Population Health Management for private health insurance scheme
Record #	370
**Inclusion/Exclusion Criteria**	
Population	Private Health Insurance in Saudi Arabia
Concept	Transformation/Reforms of Saudi Arabia Private Health Insurance Scheme
Context	GCC Health System
**Review framework domains studied in literature on private health insurance transformation**	
Health System Framework (e.g. eligibility, sector size, user charges)	
Institutional environment (e.g. legal framework, regulation)	
Private Health Insurance policy framework (e.g. objective, population coverage, system description, types of coverage	
Market structure, conduct and performance (e.g. market concentration, barriers to entry, local vs. international, vertical integration, value-based health care)	Reimbursement reforms
**Key findings related to scoping review question**	
Health System framework	
Eligibility	

Sector size	
User charges	
Institutional environment	
Legal framework	
Material regulation	
Prudential regulation	
Private Health Insurance policy framework	
Objective	
Population coverage	
System description	
Coverage type	
Market structure	
Concentration	
Barriers to entry	
Local vs international	
Vertical Integration	
Value Based Health Care	Council of Health Insurance document outlining sector strategy on introducing AR-DRG as a patient classification system with phases and implementation timelines for the market

Reviewer	Husein Reka
Date	20 January 2024
**Document information:**	
Name	VALUE-BASED HEALTH CARE IN SAUDI HEALTH INSURANCE MARKET
Author(s)	Council of Health Insurance
Year of publication	1 Jan 2022
Country/Emirate	Saudi Arabia
Link	https://chi.gov.sa/en/knowledge-center/researchlibrary/VBHC%20White%20Paper%20Version%20Final.pdf
Aim/purpose	White paper on Value-Based Health Care strategy for Cooperative Health Insurance Scheme
Record #	371
**Inclusion/Exclusion Criteria**	
Population	Private Health Insurance in Saudi Arabia
Concept	Transformation/Reforms of Saudi Arabia Private Health Insurance Scheme
Context	GCC Health System
**Review framework domains studied in literature on private health insurance transformation**	
Health System Framework (e.g. eligibility, sector size, user charges)	
Institutional environment (e.g. legal framework, regulation)	
Private Health Insurance policy framework (e.g. objective, population coverage, system description, types of coverage	
Market structure, conduct and performance (e.g. market concentration, barriers to entry, local vs. international, vertical integration, value-based health care)	Value-Based Health Care
**Key findings related to scoping review question**	
Health System framework	
Eligibility	

Sector size	
User charges	
Institutional environment	
Legal framework	
Material regulation	
Prudential regulation	
Private Health Insurance policy framework	
Objective	
Population coverage	
System description	
Coverage type	
Market structure	
Concentration	
Barriers to entry	
Local vs international	
Vertical Integration	
Value Based Health Care	Council of Health Insurance document outlining Value-Based Health Care strategy for the private health insurance sector as part of their 2020-2024, including phases and timelines of introducing value-based payments, PROMs, PREMs and other

Reviewer	Husein Reka
Date	20 January 2024
**Document information:**	
Name	VALUE-BASED HEALTH CARE IN SAUDI HEALTH INSURANCE MARKET
Author(s)	Council of Health Insurance
Year of publication	1 Jan 2022
Country/Emirate	Saudi Arabia
Link	https://chi.gov.sa/en/knowledge-center/researchlibrary/VBHC%20White%20Paper%20Version%20Final.pdf
Aim/purpose	Saudi Arabia's Patient Reported Outcome Measurement strategy
Record #	372
**Inclusion/Exclusion Criteria**	
Population	Private Health Insurance in Saudi Arabia
Concept	Transformation/Reforms of Saudi Arabia Private Health Insurance Scheme
Context	GCC Health System
**Review framework domains studied in literature on private health insurance transformation**	
Health System Framework (e.g. eligibility, sector size, user charges)	
Institutional environment (e.g. legal framework, regulation)	
Private Health Insurance policy framework (e.g. objective, population coverage, system description, types of coverage	
Market structure, conduct and performance (e.g. market concentration, barriers to entry, local vs. international, vertical integration, value-based health care)	Value-Based Health Care
**Key findings related to scoping review question**	
Health System framework	
Eligibility	

Sector size	
User charges	
Institutional environment	
Legal framework	
Material regulation	
Prudential regulation	
Private Health Insurance policy framework	
Objective	
Population coverage	
System description	
Coverage type	
Market structure	
Concentration	
Barriers to entry	
Local vs international	
Vertical Integration	
Value Based Health Care	National PROM strategy devised by the Council of Health Insurance and Center for Value Improvement in Health on conditions and PROMs tools to be introduced as part of VBP initiative

Reviewer	Husein Reka
Date	20 January 2024
**Document information:**	
Name	Regulation of qualification of claims management companies
Author(s)	Council of Health Insurance
Year of publication	2 Jul 2022
Country/Emirate	Saudi Arabia
Link	https://chi.gov.sa/en/Rules/Pages/list2.aspx
Aim/purpose	Rules and regulations qualifyin Third Party Administrators
Record #	379
**Inclusion/Exclusion Criteria**	
Population	Private Health Insurance in Saudi Arabia
Concept	Transformation/Reforms of Saudi Arabia Private Health Insurance Scheme
Context	GCC Health System
**Review framework domains studied in literature on private health insurance transformation**	
Health System Framework (e.g. eligibility, sector size, user charges)	
Institutional environment (e.g. legal framework, regulation)	Third Party Administrator qualification
Private Health Insurance policy framework (e.g. objective, population coverage, system description, types of coverage	
Market structure, conduct and performance (e.g. market concentration, barriers to entry, local vs. international, vertical integration, value-based health care)	
**Key findings related to scoping review question**	
Health System framework	
Eligibility	
Sector size	
User charges	
Institutional environment	

Legal framework	
Material regulation	Rules for qualification of Third Party Administrator for health insurance companies to operate under the Cooperative Health Insurance Scheme
Prudential regulation	
Private Health Insurance policy framework	
Objective	
Population coverage	
System description	
Coverage type	
Market structure	
Concentration	
Barriers to entry	
Local vs international	
Vertical Integration	
Value Based Health Care	National PROM strategy devised by the Council of Health Insurance and Center for Value Improvement in Health on conditions and PROMs tools to be introduced as part of VBP initiative

Reviewer	Husein Reka
Date	20 January 2024
**Document information:**	
Name	Circular no. 98 Beneficiaries Communication
Author(s)	Council of Health Insurance
Year of publication	15 Aug 2022
Country/Emirate	Saudi Arabia
Link	https://chi.gov.sa/en/Rules/Documents/Decisions/BenfCommsMEMO.pdf
Aim/purpose	Beneficiaries Communication
Record #	380
**Inclusion/Exclusion Criteria**	
Population	Private Health Insurance in Saudi Arabia
Concept	Transformation/Reforms of Saudi Arabia Private Health Insurance Scheme
Context	GCC Health System
**Review framework domains studied in literature on private health insurance transformation**	
Health System Framework (e.g. eligibility, sector size, user charges)	
Institutional environment (e.g. legal framework, regulation)	Beneficiary rights and communication
Private Health Insurance policy framework (e.g. objective, population coverage, system description, types of coverage	
Market structure, conduct and performance (e.g. market concentration, barriers to entry, local vs. international, vertical integration, value-based health care)	
**Key findings related to scoping review question**	
Health System framework	
Eligibility	
Sector size	
User charges	
Institutional environment	

Legal framework	
Material regulation	Requirements for mandatory disclosure and communication for beneficiaries by qualified health insurance companies
Prudential regulation	
Private Health Insurance policy framework	
Objective	
Population coverage	
System description	
Coverage type	
Market structure	
Concentration	
Barriers to entry	
Local vs international	
Vertical Integration	
Value Based Health Care	

Reviewer	Husein Reka
Date	20 January 2024
**Document information:**	
Name	Circular no. 957 Regarding the crieria for dispensing insulin pumps
Author(s)	Council of Health Insurance
Year of publication	15 Aug 2022
Country/Emirate	Saudi Arabia
Link	https://chi.gov.sa/en/Rules/Documents/Decisions/Regarding%20the%20criteria%20for%20dispensing%20insulin%20pumps.pdf
Aim/purpose	Beneficiaries Communication
Record #	383
**Inclusion/Exclusion Criteria**	
Population	Private Health Insurance in Saudi Arabia
Concept	Transformation/Reforms of Saudi Arabia Private Health Insurance Scheme
Context	GCC Health System
**Review framework domains studied in literature on private health insurance transformation**	
Health System Framework (e.g. eligibility, sector size, user charges)	
Institutional environment (e.g. legal framework, regulation)	Beneficiary rights and communication
Private Health Insurance policy framework (e.g. objective, population coverage, system description, types of coverage	
Market structure, conduct and performance (e.g. market concentration, barriers to entry, local vs. international, vertical integration, value-based health care)	Health Technology Assessment for Essential Benefit Package
**Key findings related to scoping review question**	
Health System framework	
Eligibility	
Sector size	

User charges	
Institutional environment	
Legal framework	
Material regulation	
Prudential regulation	
Private Health Insurance policy framework	
Objective	
Population coverage	
System description	
Coverage type	
Market structure	
Concentration	
Barriers to entry	
Local vs international	
Vertical Integration	
Value Based Health Care	Health Technology Assessment guidance to market on eligibility rules for dispensing insulin pumps as part of the new Essential Benefit Package

Reviewer	Husein Reka
Date	20 January 2024
**Document information:**	
Name	Circular no. 895 Approval of the manual of procedures regulating the relation between the Governmental Health Facilities and Insurance Companies
Author(s)	Council of Health Insurance
Year of publication	17 Dec 2020
Country/Emirate	Saudi Arabia
Link	https://chi.gov.sa/en/Rules/Documents/Decisions/Circular%20regard%20the%20approval%20of%20the%20Manual%20of%20Procedures%20regulating%20the%20relation%20between%20the%20Governmental%20Health%20Facilities%20and%20Insurance%20Companies.pdf
Aim/purpose	Manual on contracting public health providers
Record #	387
**Inclusion/Exclusion Criteria**	
Population	Private Health Insurance in Saudi Arabia
Concept	Transformation/Reforms of Saudi Arabia Private Health Insurance Scheme
Context	GCC Health System
**Review framework domains studied in literature on private health insurance transformation**	
Health System Framework (e.g. eligibility, sector size, user charges)	
Institutional environment (e.g. legal framework, regulation)	
Private Health Insurance policy framework (e.g. objective, population coverage, system description, types of coverage	Contracting public health facilities for private health insurance beneficiaries
Market structure, conduct and performance (e.g. market concentration, barriers to entry, local vs. international, vertical integration, value-based health care)	
**Key findings related to scoping review question**	

Health System framework	
Eligibility	
Sector size	
User charges	
Institutional environment	
Legal framework	
Material regulation	
Prudential regulation	
Private Health Insurance policy framework	
Objective	
Population coverage	Introduction of payment of rendered services by qualified public health care providers for beneficiaries that have Private Health Insurance coverage, through procedures regulating access, benefits and payment of rendered services through AR-DRG for admitted and SBS for non-admitted services
System description	
Coverage type	
Market structure	
Concentration	
Barriers to entry	
Local vs international	
Vertical Integration	
Value Based Health Care	

Reviewer	Husein Reka
Date	20 January 2024
**Document information:**	
Name	Urgent Circulation To All Accredited Pharmacies on Rewards and points scheme
Author(s)	Council of Health Insurance
Year of publication	1 Jan 2018
Country/Emirate	Saudi Arabia
Link	https://chi.gov.sa/en/Rules/Documents/Decisions/Urgent%20Circulation%20To%20All%20Accredited%20Pharmacies.pdf
Aim/purpose	Beneficiaries Communication
Record #	390
**Inclusion/Exclusion Criteria**	
Population	Private Health Insurance in Saudi Arabia
Concept	Transformation/Reforms of Saudi Arabia Private Health Insurance Scheme
Context	GCC Health System
**Review framework domains studied in literature on private health insurance transformation**	
Health System Framework (e.g. eligibility, sector size, user charges)	
Institutional environment (e.g. legal framework, regulation)	Prohibition of rewards for dispensed medicines
Private Health Insurance policy framework (e.g. objective, population coverage, system description, types of coverage	
Market structure, conduct and performance (e.g. market concentration, barriers to entry, local vs. international, vertical integration, value-based health care)	
**Key findings related to scoping review question**	
Health System framework	
Eligibility	
Sector size	

User charges	
Institutional environment	
Legal framework	
Material regulation	Prohibition on providing offers to beneficiaries when dispensing medicines
Prudential regulation	
Private Health Insurance policy framework	
Objective	
Population coverage	
System description	
Coverage type	
Market structure	
Concentration	
Barriers to entry	
Local vs international	
Vertical Integration	
Value Based Health Care	

Reviewer	Husein Reka
Date	20 January 2024
**Document information:**	
Name	Circular no. 1733/1439/3531/ Urgent Circulation To All Qualified Companies and Accredited Healthcare Service Providers on copyament rules
Author(s)	Council of Health Insurance
Year of publication	30 Jul 2018
Country/Emirate	Saudi Arabia
Link	https://chi.gov.sa/en/Rules/Documents/Decisions/Urgent%20Circulation%20To%20All%20Qualified%20Companies%20and%20Accredited%20Healthcare%20Service%20Providers.pdf
Aim/purpose	Copayment rulse
Record #	391
**Inclusion/Exclusion Criteria**	
Population	Private Health Insurance in Saudi Arabia
Concept	Transformation/Reforms of Saudi Arabia Private Health Insurance Scheme
Context	GCC Health System
**Review framework domains studied in literature on private health insurance transformation**	
Health System Framework (e.g. eligibility, sector size, user charges)	User charges regime
Institutional environment (e.g. legal framework, regulation)	
Private Health Insurance policy framework (e.g. objective, population coverage, system description, types of coverage	
Market structure, conduct and performance (e.g. market concentration, barriers to entry, local vs. international, vertical integration, value-based health care)	
**Key findings related to scoping review question**	
Health System framework	

Eligibility	
Sector size	
User charges	Co-payment rules: MPN: (0-20%) Max 75 SR OHN: (0-20%) Max 300 SR OCN: (0-20%) Max 100 SR
Institutional environment	
Legal framework	
Material regulation	
Prudential regulation	
Private Health Insurance policy framework	
Objective	
Population coverage	
System description	
Coverage type	
Market structure	
Concentration	
Barriers to entry	
Local vs international	
Vertical Integration	
Value Based Health Care	

Reviewer	Husein Reka
Date	20 January 2024
**Document information:**	
Name	Health Facilities Officials - National Address registration
Author(s)	Council of Health Insurance
Year of publication	1 Jan 2015
Country/Emirate	Saudi Arabia
Link	https://chi.gov.sa/en/Rules/Documents/Decisions/Health%20Facility%20Officials.pdf
Aim/purpose	National address registration requirement
Record #	392
**Inclusion/Exclusion Criteria**	
Population	Private Health Insurance in Saudi Arabia
Concept	Transformation/Reforms of Saudi Arabia Private Health Insurance Scheme
Context	GCC Health System
**Review framework domains studied in literature on private health insurance transformation**	
Health System Framework (e.g. eligibility, sector size, user charges)	
Institutional environment (e.g. legal framework, regulation)	National address requirement
Private Health Insurance policy framework (e.g. objective, population coverage, system description, types of coverage	
Market structure, conduct and performance (e.g. market concentration, barriers to entry, local vs. international, vertical integration, value-based health care)	
**Key findings related to scoping review question**	
Health System framework	
Eligibility	
Sector size	
User charges	
Institutional environment	

Legal framework	
Material regulation	Requirement to register national address
Prudential regulation	
Private Health Insurance policy framework	
Objective	
Population coverage	
System description	
Coverage type	
Market structure	
Concentration	
Barriers to entry	
Local vs international	
Vertical Integration	
Value Based Health Care	

Reviewer	Husein Reka
Date	20 January 2024
**Document information:**	
Name	CHI-08-BL-03-1/0 Telehealth policies and procedures (Telemedicine)
Author(s)	Council of Health Insurance
Year of publication	1 Jan 2022
Country/Emirate	Saudi Arabia
Link	https://www.chi.gov.sa/knowledge-center/DocLib/%D8%A7%D9%84%D8%B3%D9%8A%D8%A7%D8%B3%D8%A7%D8%AA%20%D9%88%D8%A7%D9%84%D8%A5%D8%AC%D8%B1%D8%A7%D8%A1%D8%A7%D8%AA%20%D8%A7%D9%84%D9%85%D9%86%D8%B8%D9%85%D8%A9%20%D9%84%D9%84%D8%B1%D8%B9%D8%A7%D9%8A%D8%A9%20%D8%A7%D9%84%D8%B5%D8%AD%D9%8A%D8%A9%20%D8%B9%D9%86%20%D8%A8%D8%B9%D8%AF.pdf
Aim/purpose	Telemedicine inclusion in EBP
Record #	393
**Inclusion/Exclusion Criteria**	
Population	Private Health Insurance in Saudi Arabia
Concept	Transformation/Reforms of Saudi Arabia Private Health Insurance Scheme
Context	GCC Health System
**Review framework domains studied in literature on private health insurance transformation**	
Health System Framework (e.g. eligibility, sector size, user charges)	Benefit package addition
Institutional environment (e.g. legal framework, regulation)	
Private Health Insurance policy framework (e.g. objective, population coverage, system description, types of coverage	

Market structure, conduct and performance (e.g. market concentration, barriers to entry, local vs. international, vertical integration, value-based health care)	
**Key findings related to scoping review question**	
Health System framework	
Eligibility	Rules and procedures of providing telemedicine/telehealth services as part of new Essential Benefit Package
Sector size	
User charges	
Institutional environment	
Legal framework	
Material regulation	
Prudential regulation	
Private Health Insurance policy framework	
Objective	
Population coverage	
System description	
Coverage type	
Market structure	
Concentration	
Barriers to entry	
Local vs international	
Vertical Integration	
Value Based Health Care	

Reviewer	Husein Reka
Date	20 January 2024
**Document information:**	
Name	CCHI-08-BL-02-1/0 Health Insurance Beneficiaries Rights Board of Directors session no.117
Author(s)	Council of Health Insurance
Year of publication	7 Nov 2021
Country/Emirate	Saudi Arabia
Link	https://chi.gov.sa/en/AboutCCHI/Rules/document/Health%20Insurance%20Beneficiaries%20Rights%20177%20.pdf
Aim/purpose	Health insurance beneficiaries rights
Record #	394
**Inclusion/Exclusion Criteria**	
Population	Private Health Insurance in Saudi Arabia
Concept	Transformation/Reforms of Saudi Arabia Private Health Insurance Scheme
Context	GCC Health System
**Review framework domains studied in literature on private health insurance transformation**	
Health System Framework (e.g. eligibility, sector size, user charges)	
Institutional environment (e.g. legal framework, regulation)	Health insurance beneficiaries rights
Private Health Insurance policy framework (e.g. objective, population coverage, system description, types of coverage	
Market structure, conduct and performance (e.g. market concentration, barriers to entry, local vs. international, vertical integration, value-based health care)	
**Key findings related to scoping review question**	
Health System framework	
Eligibility	
Sector size	
User charges	

Institutional environment	
Legal framework	
Material regulation	Health insurance beneficiaries’ rights
Prudential regulation	
Private Health Insurance policy framework	
Objective	
Population coverage	
System description	
Coverage type	
Market structure	
Concentration	
Barriers to entry	
Local vs international	
Vertical Integration	
Value Based Health Care	

Reviewer	Husein Reka
Date	20 January 2024
**Document information:**	
Name	CCHI-06-PR/EN-02-1/0 Private Health Insurance Sector Conduct Policy (Fraud, Waste and Abuse)
Author(s)	Council of Health Insurance
Year of publication	1 Jan 2020
Country/Emirate	Saudi Arabia
Link	https://www.ia.gov.sa/Documents/HealthInsuranceAndCirculars/en/Policy_Private%20Health%20Insurance%20Sector%20Conduct%20Policy.pdf
Aim/purpose	Policy on Fraud, Waste and Abuse
Record #	395
**Inclusion/Exclusion Criteria**	
Population	Private Health Insurance in Saudi Arabia
Concept	Transformation/Reforms of Saudi Arabia Private Health Insurance Scheme
Context	GCC Health System
**Review framework domains studied in literature on private health insurance transformation**	
Health System Framework (e.g. eligibility, sector size, user charges)	
Institutional environment (e.g. legal framework, regulation)	Policy on Fraud, Waste and Abuse
Private Health Insurance policy framework (e.g. objective, population coverage, system description, types of coverage	
Market structure, conduct and performance (e.g. market concentration, barriers to entry, local vs. international, vertical integration, value-based health care)	
**Key findings related to scoping review question**	
Health System framework	
Eligibility	
Sector size	

User charges	
Institutional environment	
Legal framework	
Material regulation	Policy on Fraud, Waste and Abuse
Prudential regulation	
Private Health Insurance policy framework	
Objective	
Population coverage	
System description	
Coverage type	
Market structure	
Concentration	
Barriers to entry	
Local vs international	
Vertical Integration	
Value Based Health Care	

Reviewer	Husein Reka
Date	20 January 2024
**Document information:**	
Name	The Benefits of Unified Policy for Council of Cooperative Health Insurance
Author(s)	Council of Health Insurance
Year of publication	1 Jan 2018
Country/Emirate	Saudi Arabia
Link	https://chi.gov.sa/en/AboutCCHI/Rules/document/The%20Benefits%20of%20Unified%20Policy%20for%20Council%20of%20Cooperative%20Health%20Insurance.pdf
Aim/purpose	Benefit package elements (old benefits)
Record #	396
**Inclusion/Exclusion Criteria**	
Population	Private Health Insurance in Saudi Arabia
Concept	Transformation/Reforms of Saudi Arabia Private Health Insurance Scheme
Context	GCC Health System
**Review framework domains studied in literature on private health insurance transformation**	
Health System Framework (e.g. eligibility, sector size, user charges)	Benefit package elements (old benefits)
Institutional environment (e.g. legal framework, regulation)	
Private Health Insurance policy framework (e.g. objective, population coverage, system description, types of coverage	
Market structure, conduct and performance (e.g. market concentration, barriers to entry, local vs. international, vertical integration, value-based health care)	
**Key findings related to scoping review question**	
Health System framework	
Eligibility	Benefit package elements (old benefits)
Sector size	

User charges	
Institutional environment	
Legal framework	
Material regulation	Policy on Fraud, Waste and Abuse
Prudential regulation	
Private Health Insurance policy framework	
Objective	
Population coverage	
System description	
Coverage type	
Market structure	
Concentration	
Barriers to entry	
Local vs international	
Vertical Integration	
Value Based Health Care	

Reviewer	Husein Reka
Date	20 January 2024
**Document information:**	
Name	Ministerial Decision no. R/1/18/3 Cooperative Health Insurance Policy
Author(s)	Council of Health Insurance
Year of publication	16 Aug 2018
Country/Emirate	Saudi Arabia
Link	https://chi.gov.sa/en/AboutCCHI/Rules/document/The%20Benefits%20of%20Unified%20Policy%20for%20Council%20of%20Cooperative%20Health%20Insurance.pdf
Aim/purpose	Schedule of Benefits for beneficiaries (old schdule of benefits)
Record #	397
**Inclusion/Exclusion Criteria**	
Population	Private Health Insurance in Saudi Arabia
Concept	Transformation/Reforms of Saudi Arabia Private Health Insurance Scheme
Context	GCC Health System
**Review framework domains studied in literature on private health insurance transformation**	
Health System Framework (e.g. eligibility, sector size, user charges)	Schedule of Benefits for beneficiaries (old schdule of benefits)
Institutional environment (e.g. legal framework, regulation)	
Private Health Insurance policy framework (e.g. objective, population coverage, system description, types of coverage	
Market structure, conduct and performance (e.g. market concentration, barriers to entry, local vs. international, vertical integration, value-based health care)	
**Key findings related to scoping review question**	
Health System framework	

Eligibility	Schedule of Benefits for beneficiaries (old schdule of benefits)
Sector size	
User charges	
Institutional environment	
Legal framework	
Material regulation	
Prudential regulation	
Private Health Insurance policy framework	
Objective	
Population coverage	
System description	
Coverage type	
Market structure	
Concentration	
Barriers to entry	
Local vs international	
Vertical Integration	
Value Based Health Care	

Reviewer	Husein Reka
Date	20 January 2024
**Document information:**	
Name	Ministerial Decision no. 9/25/1/DH IMPLEMENTING REGULATIONS OF THE COOPERATIVE HEALTH INSURANCE LAW
Author(s)	Council of Health Insurance
Year of publication	13 Feb 2014
Country/Emirate	Saudi Arabia
Link	https://chi.gov.sa/en/AboutCCHI/Rules/document/The%20Benefits%20of%20Unified%20Policy%20for%20Council%20of%20Cooperative%20Health%20Insurance.pdf
Aim/purpose	Implementing Regulations of Cooperative Health Insurance Law
Record #	398
**Inclusion/Exclusion Criteria**	
Population	Private Health Insurance in Saudi Arabia
Concept	Transformation/Reforms of Saudi Arabia Private Health Insurance Scheme
Context	GCC Health System
**Review framework domains studied in literature on private health insurance transformation**	
Health System Framework (e.g. eligibility, sector size, user charges)	
Institutional environment (e.g. legal framework, regulation)	Implementing Regulations of Cooperative Health Insurance Law
Private Health Insurance policy framework (e.g. objective, population coverage, system description, types of coverage	
Market structure, conduct and performance (e.g. market concentration, barriers to entry, local vs. international, vertical integration, value-based health care)	
**Key findings related to scoping review question**	

Health System framework	
Eligibility	
Sector size	
User charges	
Institutional environment	
Legal framework	Implementing Regulations of Cooperative Health Insurance Law
Material regulation	
Prudential regulation	
Private Health Insurance policy framework	
Objective	
Population coverage	
System description	
Coverage type	
Market structure	
Concentration	
Barriers to entry	
Local vs international	
Vertical Integration	
Value Based Health Care	

Reviewer	Husein Reka
Date	20 January 2024
**Document information:**	
Name	Benefit Package Tiers for Private Health Insurance Beneficiaries
Author(s)	Council of Health Insurance
Year of publication	1 Oct 2022
Country/Emirate	Saudi Arabia
Link	https://chi.gov.sa/en/Rules/PublishingImages/Pages/Forms/EditForm/EBP_Booklet_5_Tiers%20Benefit%20Package%20for%20Private%20Health%20Insurance%20Beneficiaries_EN.pdf
Aim/purpose	Standardized products for new Essential Benefit Package
Record #	399
**Inclusion/Exclusion Criteria**	
Population	Private Health Insurance in Saudi Arabia
Concept	Transformation/Reforms of Saudi Arabia Private Health Insurance Scheme
Context	GCC Health System
**Review framework domains studied in literature on private health insurance transformation**	
Health System Framework (e.g. eligibility, sector size, user charges)	Standardized products for new Essential Benefit Package
Institutional environment (e.g. legal framework, regulation)	
Private Health Insurance policy framework (e.g. objective, population coverage, system description, types of coverage	
Market structure, conduct and performance (e.g. market concentration, barriers to entry, local vs. international, vertical integration, value-based health care)	
**Key findings related to scoping review question**	
Health System framework	
Eligibility	Standardized products for new Essential Benefit Package

Sector size	
User charges	
Institutional environment	
Legal framework	
Material regulation	
Prudential regulation	
Private Health Insurance policy framework	
Objective	
Population coverage	
System description	
Coverage type	
Market structure	
Concentration	
Barriers to entry	
Local vs international	
Vertical Integration	
Value Based Health Care	

Reviewer	Husein Reka
Date	20 January 2024
**Document information:**	
Name	Insurance Drug Formulary
Author(s)	Council of Health Insurance
Year of publication	1 Oct 2022
Country/Emirate	Saudi Arabia
Link	https://chi.gov.sa/en/Rules/Documents/Insurance%20Drug%20Formulary%20(IDF).pdf
Aim/purpose	Introduction of Drug Formulary for private health insurance with requirement for generic prescription and dispensing
Record #	400
**Inclusion/Exclusion Criteria**	
Population	Private Health Insurance in Saudi Arabia
Concept	Transformation/Reforms of Saudi Arabia Private Health Insurance Scheme
Context	GCC Health System
**Review framework domains studied in literature on private health insurance transformation**	
Health System Framework (e.g. eligibility, sector size, user charges)	Introduction of Drug Formulary for private health insurance with requirement for generic prescription and dispensing
Institutional environment (e.g. legal framework, regulation)	
Private Health Insurance policy framework (e.g. objective, population coverage, system description, types of coverage	
Market structure, conduct and performance (e.g. market concentration, barriers to entry, local vs. international, vertical integration, value-based health care)	
**Key findings related to scoping review question**	
Health System framework	

Eligibility	Introduction of Drug Formulary for private health insurance with requirement for generic prescription and dispensing
Sector size	
User charges	
Institutional environment	
Legal framework	
Material regulation	
Prudential regulation	
Private Health Insurance policy framework	
Objective	
Population coverage	
System description	
Coverage type	
Market structure	
Concentration	
Barriers to entry	
Local vs international	
Vertical Integration	
Value Based Health Care	

Reviewer	Husein Reka
Date	20 January 2024
**Document information:**	
Name	CHI's Enhanced Essential Benefit Package: Prioritizing Your Health & Wellbeing
Author(s)	Council of Health Insurance
Year of publication	1 Oct 2022
Country/Emirate	Saudi Arabia
Link	https://eportal.cchi.gov.sa/EBP/pdfs/reports/EBP/en/EBP_Booklet_1_Beneficiaries_En.pdf
Aim/purpose	Communication document to beneficiaries on new EBP
Record #	401
**Inclusion/Exclusion Criteria**	
Population	Private Health Insurance in Saudi Arabia
Concept	Transformation/Reforms of Saudi Arabia Private Health Insurance Scheme
Context	GCC Health System
**Review framework domains studied in literature on private health insurance transformation**	
Health System Framework (e.g. eligibility, sector size, user charges)	Communication document to beneficiaries on new EBP
Institutional environment (e.g. legal framework, regulation)	
Private Health Insurance policy framework (e.g. objective, population coverage, system description, types of coverage	
Market structure, conduct and performance (e.g. market concentration, barriers to entry, local vs. international, vertical integration, value-based health care)	
**Key findings related to scoping review question**	
Health System framework	
Eligibility	Communication document to beneficiaries on new EBP
Sector size	
User charges	

Institutional environment	
Legal framework	
Material regulation	
Prudential regulation	
Private Health Insurance policy framework	
Objective	
Population coverage	
System description	
Coverage type	
Market structure	
Concentration	
Barriers to entry	
Local vs international	
Vertical Integration	
Value Based Health Care	

Reviewer	Husein Reka
Date	20 January 2024
**Document information:**	
Name	Decree No. (5) Of 2013 establishing the Supreme Council of Health
Author(s)	Council of Ministers
Year of publication	31 Jan 2013
Country/Emirate	Bahrain
Link	https://www.lloc.gov.bh/PDF/D0513.pdf
Aim/purpose	Legal framework for health insurance regulation
Record #	426
**Inclusion/Exclusion Criteria**	
Population	Private Health Insurance in Bahrain
Concept	Transformation/Reforms of Bahrain Private Health Insurance Scheme
Context	GCC Health System
**Review framework domains studied in literature on private health insurance transformation**	
Health System Framework (e.g. eligibility, sector size, user charges)	
Institutional environment (e.g. legal framework, regulation)	Legal framework for health insurance mandate and regulation
Private Health Insurance policy framework (e.g. objective, population coverage, system description, types of coverage	
Market structure, conduct and performance (e.g. market concentration, barriers to entry, local vs. international, vertical integration, value-based health care)	
**Key findings related to scoping review question**	
Health System framework	
Eligibility	
Sector size	
User charges	

Institutional environment	
Legal framework	Article 4 point 2 gives Bahrain SCH to develop a policy for financing health services through implementing a comprehensive health insurance system
Material regulation	
Prudential regulation	
Private Health Insurance policy framework	
Objective	
Population coverage	
System description	
Coverage type	
Market structure	
Concentration	
Barriers to entry	
Local vs international	
Vertical Integration	
Value Based Health Care	

Reviewer	Husein Reka
Date	20 January 2024
**Document information:**	
Name	Decree No. (35) For the year 2019 amending some provisions of Decree No. (5) For the year 2013 establishing the Supreme Council of Health.
Author(s)	Council of Ministers
Year of publication	11 Apr 2019
Country/Emirate	Bahrain
Link	https://www.lloc.gov.bh/PDF/D3519.pdf
Aim/purpose	Additional mandates for SCH
Record #	457
**Inclusion/Exclusion Criteria**	
Population	Private Health Insurance in Bahrain
Concept	Transformation/Reforms of Bahrain Private Health Insurance Scheme
Context	GCC Health System
**Review framework domains studied in literature on private health insurance transformation**	
Health System Framework (e.g. eligibility, sector size, user charges)	
Institutional environment (e.g. legal framework, regulation)	Expanding legal framework for health insurance mandate and regulation
Private Health Insurance policy framework (e.g. objective, population coverage, system description, types of coverage	
Market structure, conduct and performance (e.g. market concentration, barriers to entry, local vs. international, vertical integration, value-based health care)	
**Key findings related to scoping review question**	
Health System framework	
Eligibility	
Sector size	

User charges	
Institutional environment	
Legal framework	New clause added to SCH mandate to establish policies for the supply, storage, supervision and management of medicines used in the health insurance system
Material regulation	
Prudential regulation	
Private Health Insurance policy framework	
Objective	
Population coverage	
System description	
Coverage type	
Market structure	
Concentration	
Barriers to entry	
Local vs international	
Vertical Integration	
Value Based Health Care	

Reviewer	Husein Reka
Date	20 January 2024
**Document information:**	
Name	Decision of the Chairman of the Supreme Council of Health No. (43) For the year 2019 determining the hospitals and primary healthcare centers that are subject to the Health Insurance Law issued by Law No. (23) For the year 2018
Author(s)	Supreme Council of Health
Year of publication	9 May 2019
Country/Emirate	Bahrain
Link	https://www.lloc.gov.bh/PDF/RSCH4319.pdf
Aim/purpose	Additional mandates for SCH
Record #	459
**Inclusion/Exclusion Criteria**	
Population	Private Health Insurance in Bahrain
Concept	Transformation/Reforms of Bahrain Private Health Insurance Scheme
Context	GCC Health System
**Review framework domains studied in literature on private health insurance transformation**	
Health System Framework (e.g. eligibility, sector size, user charges)	
Institutional environment (e.g. legal framework, regulation)	
Private Health Insurance policy framework (e.g. objective, population coverage, system description, types of coverage	Extension of provider network to public health facilities
Market structure, conduct and performance (e.g. market concentration, barriers to entry, local vs. international, vertical integration, value-based health care)	
**Key findings related to scoping review question**	
Health System framework	
Eligibility	

Sector size	
User charges	
Institutional environment	
Legal framework	
Material regulation	
Prudential regulation	
Private Health Insurance policy framework	
Objective	
Population coverage	Extended government health care providers coverage through Health Insurance Law: 1- Hospitals affiliated with the Ministry of Health, the statement of which is attached to this decision. 2- Primary health care centers, the statement of which is attached to this decision.
System description	
Coverage type	
Market structure	
Concentration	
Barriers to entry	
Local vs international	
Vertical Integration	
Value Based Health Care	

Reviewer	Husein Reka
Date	20 January 2024
**Document information:**	
Name	Royal decree no. (35) For the year 2019 on regulating the relationship of the Bahrain Defense Force with the Supreme Council of Health and the Health Insurance Fund.
Author(s)	Royal Court
Year of publication	4 Jul 2019
Country/Emirate	Bahrain
Link	https://www.lloc.gov.bh/PDF/O3519.pdf
Aim/purpose	Extension of eligibility
Record #	460
**Inclusion/Exclusion Criteria**	
Population	Private Health Insurance in Bahrain
Concept	Transformation/Reforms of Bahrain Private Health Insurance Scheme
Context	GCC Health System
**Review framework domains studied in literature on private health insurance transformation**	
Health System Framework (e.g. eligibility, sector size, user charges)	Royal decree on expanding eligibility for health insurance
Institutional environment (e.g. legal framework, regulation)	
Private Health Insurance policy framework (e.g. objective, population coverage, system description, types of coverage	
Market structure, conduct and performance (e.g. market concentration, barriers to entry, local vs. international, vertical integration, value-based health care)	
**Key findings related to scoping review question**	
Health System framework	
Eligibility	Extending coverage to members of the Bahrain Defense Force and their family members at the expense of the

	state to all governmental health institutions and centers subject to the Health Insurance Law promulgated by Law No. (23) of 2018
Sector size	
User charges	
Institutional environment	
Legal framework	
Material regulation	
Prudential regulation	
Private Health Insurance policy framework	
Objective	
Population coverage	
System description	
Coverage type	
Market structure	
Concentration	
Barriers to entry	
Local vs international	
Vertical Integration	
Value Based Health Care	

Reviewer	Husein Reka
Date	20 January 2024
**Document information:**	
Name	Decision of the Chairman of the Supreme Council of Health No. (39) Of 2022 on the regulation of private medical practice in government hospitals.
Author(s)	Supreme Council of Health
Year of publication	23 Jun 2022
Country/Emirate	Bahrain
Link	https://www.lloc.gov.bh/PDF/RSCH3922.pdf
Aim/purpose	Establishing business centers in government hospitals
Record #	486
**Inclusion/Exclusion Criteria**	
Population	Private Health Insurance in Bahrain
Concept	Transformation/Reforms of Bahrain Private Health Insurance Scheme
Context	GCC Health System
**Review framework domains studied in literature on private health insurance transformation**	
Health System Framework (e.g. eligibility, sector size, user charges)	
Institutional environment (e.g. legal framework, regulation)	
Private Health Insurance policy framework (e.g. objective, population coverage, system description, types of coverage	Establishing business centres able to bill private health insurance
Market structure, conduct and performance (e.g. market concentration, barriers to entry, local vs. international, vertical integration, value-based health care)	
**Key findings related to scoping review question**	
Health System framework	
Eligibility	
Sector size	
User charges	

Institutional environment	
Legal framework	
Material regulation	
Prudential regulation	
Private Health Insurance policy framework	
Objective	
Population coverage	Rules for government hospitals to establish private provision of care via health insurance companies by establishing Private Health Care Teams
System description	
Coverage type	
Market structure	
Concentration	
Barriers to entry	
Local vs international	
Vertical Integration	
Value Based Health Care	

Reviewer	Husein Reka
Date	20 January 2024
**Document information:**	
Name	Health insurance law.
Author(s)	Council of Ministers
Year of publication	30 May 2018
Country/Emirate	Bahrain
Link	https://www.moh.gov.bh/Content/Upload/File/638248457033688553-SEHATI-Law_en.pdf
Aim/purpose	Law establishing mandatory health insurance scheme SEHATI
Record #	488
**Inclusion/Exclusion Criteria**	
Population	Private Health Insurance in Bahrain
Concept	Transformation/Reforms of Bahrain Private Health Insurance Scheme
Context	GCC Health System
**Review framework domains studied in literature on private health insurance transformation**	
Health System Framework (e.g. eligibility, sector size, user charges)	
Institutional environment (e.g. legal framework, regulation)	Mandatory health insurance scheme law
Private Health Insurance policy framework (e.g. objective, population coverage, system description, types of coverage	
Market structure, conduct and performance (e.g. market concentration, barriers to entry, local vs. international, vertical integration, value-based health care)	
**Key findings related to scoping review question**	
Health System framework	
Eligibility	
Sector size	
User charges	

Institutional environment	
Legal framework	Law establishing Sehaty Health Insurance Scheme and Health Insurance Fund in the Kingdom of Bahrain (SEHATI)
Material regulation	
Prudential regulation	
Private Health Insurance policy framework	
Objective	
Population coverage	
System description	
Coverage type	
Market structure	
Concentration	
Barriers to entry	
Local vs international	
Vertical Integration	
Value Based Health Care	

Reviewer	Husein Reka
Date	20 January 2024
**Document information:**	
Name	Health insurance law.
Author(s)	Council of Ministers
Year of publication	30 May 2018
Country/Emirate	Bahrain
Link	https://www.moh.gov.bh/Content/Upload/File/638248457033688553-SEHATI-Law_en.pdf
Aim/purpose	Law establishing mandatory health insurance scheme SEHATI
Record #	488
**Inclusion/Exclusion Criteria**	
Population	Private Health Insurance in Bahrain
Concept	Transformation/Reforms of Bahrain Private Health Insurance Scheme
Context	GCC Health System
**Review framework domains studied in literature on private health insurance transformation**	
Health System Framework (e.g. eligibility, sector size, user charges)	
Institutional environment (e.g. legal framework, regulation)	Mandatory health insurance scheme law
Private Health Insurance policy framework (e.g. objective, population coverage, system description, types of coverage	
Market structure, conduct and performance (e.g. market concentration, barriers to entry, local vs. international, vertical integration, value-based health care)	
**Key findings related to scoping review question**	
Health System framework	
Eligibility	
Sector size	
User charges	

Institutional environment	
Legal framework	Law establishing Sehaty Health Insurance Scheme and Health Insurance Fund in the Kingdom of Bahrain (SEHATI)
Material regulation	
Prudential regulation	
Private Health Insurance policy framework	
Objective	
Population coverage	
System description	
Coverage type	
Market structure	
Concentration	
Barriers to entry	
Local vs international	
Vertical Integration	
Value Based Health Care	

Reviewer	Husein Reka
Date	20 January 2024
**Document information:**	
Name	Executive Council resolution no.1 of 2018 Approving fees for Dubai Corporation for Ambulance Services
Author(s)	Government of Dubai
Year of publication	24 Jan 2018
Country/Emirate	Dubai
Link	https://www.isahd.ae/content/docs/Executive%20Council%20Resolution%20No%201%20of%202018%20%E2%80%93%20Ambulance%20Service%20Fees.pdf
Aim/purpose	Advise on VAT treatment of BASMAH fund transfer
Record #	496
**Inclusion/Exclusion Criteria**	
Population	Private Health Insurance in Dubai
Concept	Transformation/Reforms of Dubai Private Health Insurance Scheme
Context	GCC Health System
**Review framework domains studied in literature on private health insurance transformation**	
Health System Framework (e.g. eligibility, sector size, user charges)	
Institutional environment (e.g. legal framework, regulation)	
Private Health Insurance policy framework (e.g. objective, population coverage, system description, types of coverage	Inclusion of ambulance services in mandatory health insurance (ISAHD) scheme
Market structure, conduct and performance (e.g. market concentration, barriers to entry, local vs. international, vertical integration, value-based health care)	
**Key findings related to scoping review question**	
Health System framework	
Eligibility	
Sector size	

User charges	
Institutional environment	
Legal framework	
Material regulation	
Prudential regulation	
Private Health Insurance policy framework	
Objective	
Population coverage	Health Insurance Companies to cover ambulance service as per schedule of fees for their insurees via Duabi Ambulance Services Corporation
System description	
Coverage type	
Market structure	
Concentration	
Barriers to entry	
Local vs international	
Vertical Integration	
Value Based Health Care	

Reviewer	Husein Reka
Date	20 January 2024
**Document information:**	
Name	COM decision no. 27 of 2016 on establishing a committee to select PHI companies to provide health insurance for citizens
Author(s)	Council of Ministers
Year of publication	17 Nov 2016
Country/Emirate	Qatar
Link	https://www.almeezan.qa/ClarificationsNoteDetails.aspx?id=14590&language=ar
Aim/purpose	Preparations for tender for PHI coverage for citizens
Record #	713
**Inclusion/Exclusion Criteria**	
Population	Private Health Insurance in Qatar
Concept	Transformation/Reforms of Qatar Private Health Insurance Scheme
Context	GCC Health System
**Review framework domains studied in literature on private health insurance transformation**	
Health System Framework (e.g. eligibility, sector size, user charges)	
Institutional environment (e.g. legal framework, regulation)	
Private Health Insurance policy framework (e.g. objective, population coverage, system description, types of coverage	Interim PHI coverage tender process for citizens of Qatar
Market structure, conduct and performance (e.g. market concentration, barriers to entry, local vs. international, vertical integration, value-based health care)	
**Key findings related to scoping review question**	
Health System framework	
Eligibility	
Sector size	

User charges	
Institutional environment	
Legal framework	
Material regulation	
Prudential regulation	
Private Health Insurance policy framework	
Objective	
Population coverage	Council of Ministers decision to establish a selection committee to select Private Health Insurance companies that will provide health insurance coverage for citizens (post abolition of SEHA health insurance scheme). The committee task was to issue a tender for selection of insurance providers based on Ministry of Public Health benefit package recommended until the new health insurance law is promulgated
System description	
Coverage type	
Market structure	
Concentration	
Barriers to entry	
Local vs international	
Vertical Integration	
Value Based Health Care	

Reviewer	Husein Reka
Date	20 January 2024
**Document information:**	
Name	Law no. 7 of 2013 on establishing social health insurance scheme
Author(s)	Council of Ministers
Year of publication	16 Jun 2013
Country/Emirate	Qatar
Link	https://www.almeezan.qa/ClarificationsNoteDetails.aspx?id=8987&language=ar
Aim/purpose	Promulgation of Social Health Insurance scheme SEHA
Record #	717
**Inclusion/Exclusion Criteria**	
Population	Private Health Insurance in Qatar
Concept	Transformation/Reforms of Qatar Private Health Insurance Scheme
Context	GCC Health System
**Review framework domains studied in literature on private health insurance transformation**	
Health System Framework (e.g. eligibility, sector size, user charges)	
Institutional environment (e.g. legal framework, regulation)	Law establishing Social Health Insurance Scheme (SEHA) in Qatar
Private Health Insurance policy framework (e.g. objective, population coverage, system description, types of coverage	
Market structure, conduct and performance (e.g. market concentration, barriers to entry, local vs. international, vertical integration, value-based health care)	
**Key findings related to scoping review question**	
Health System framework	
Eligibility	
Sector size	

User charges	
Institutional environment	
Legal framework	Although this law established a social health insurance scheme through single payer system, article 11 of this law is the first ever provision enabling Private Health Insurance companies to provide additional health services and the requirement to be licensed with health insurance regulator for the same
Material regulation	
Prudential regulation	
Private Health Insurance policy framework	
Objective	
Population coverage	
System description	
Coverage type	
Market structure	
Concentration	
Barriers to entry	
Local vs international	
Vertical Integration	
Value Based Health Care	

Reviewer	Husein Reka
Date	20 January 2024
**Document information:**	
Name	Minister of Public Health Decree No. 22 of 2013 on Implemeting Regulations of Law No. 7 of 2013 on social health insurance scheme
Author(s)	Minister of Public Health
Year of publication	28 Oct 2013
Country/Emirate	Qatar
Link	https://www.almeezan.qa/ClarificationsNoteDetails.aspx?id=9791&language=ar
Aim/purpose	SEHA scheme implementing regulations
Record #	718
**Inclusion/Exclusion Criteria**	
Population	Private Health Insurance in Qatar
Concept	Transformation/Reforms of Qatar Private Health Insurance Scheme
Context	GCC Health System
**Review framework domains studied in literature on private health insurance transformation**	
Health System Framework (e.g. eligibility, sector size, user charges)	
Institutional environment (e.g. legal framework, regulation)	Law establishing Social Health Insurance Scheme (SEHA) in Qatar
Private Health Insurance policy framework (e.g. objective, population coverage, system description, types of coverage	
Market structure, conduct and performance (e.g. market concentration, barriers to entry, local vs. international, vertical integration, value-based health care)	
**Key findings related to scoping review question**	
Health System framework	
Eligibility	

Sector size	
User charges	
Institutional environment	
Legal framework	Chapter four of this regulation regulates comprehensively the participation of private health insurance companies through regulatory mechanisms
Material regulation	
Prudential regulation	
Private Health Insurance policy framework	
Objective	
Population coverage	
System description	
Coverage type	
Market structure	
Concentration	
Barriers to entry	
Local vs international	
Vertical Integration	
Value Based Health Care	

Reviewer	Husein Reka
Date	20 January 2024
**Document information:**	
Name	Minister of Business and Trade decision No. 16 of 2013 on establishing the National Health Insurance Company QSC
Author(s)	Ministry of Business and Trade
Year of publication	14 Apr 2013
Country/Emirate	Qatar
Link	https://www.almeezan.qa/ClarificationsNoteDetails.aspx?id=12581&language=ar
Aim/purpose	National Health Insurance Company establishment
Record #	719
**Inclusion/Exclusion Criteria**	
Population	Private Health Insurance in Qatar
Concept	Transformation/Reforms of Qatar Private Health Insurance Scheme
Context	GCC Health System
**Review framework domains studied in literature on private health insurance transformation**	
Health System Framework (e.g. eligibility, sector size, user charges)	
Institutional environment (e.g. legal framework, regulation)	Decree establishing National Health Insurance Company
Private Health Insurance policy framework (e.g. objective, population coverage, system description, types of coverage	
Market structure, conduct and performance (e.g. market concentration, barriers to entry, local vs. international, vertical integration, value-based health care)	
**Key findings related to scoping review question**	
Health System framework	
Eligibility	
Sector size	

User charges	
Institutional environment	
Legal framework	National Health Insurance Company as a single payer for the basic benefit package established as a Qatari Shareholding Company with Ministry of Business and Trade
Material regulation	
Prudential regulation	
Private Health Insurance policy framework	
Objective	
Population coverage	
System description	
Coverage type	
Market structure	
Concentration	
Barriers to entry	
Local vs international	
Vertical Integration	
Value Based Health Care	

Reviewer	Husein Reka
Date	20 January 2024
**Document information:**	
Name	Minister of Business and Trade decision No. 16 of 2013 on establishing the National Health Insurance Company QSC
Author(s)	Ministry of Business and Trade
Year of publication	14 Apr 2013
Country/Emirate	Qatar
Link	https://www.almeezan.qa/ClarificationsNoteDetails.aspx?id=12581&language=ar
Aim/purpose	National Health Insurance Company establishment
Record #	719
**Inclusion/Exclusion Criteria**	
Population	Private Health Insurance in Qatar
Concept	Transformation/Reforms of Qatar Private Health Insurance Scheme
Context	GCC Health System
**Review framework domains studied in literature on private health insurance transformation**	
Health System Framework (e.g. eligibility, sector size, user charges)	
Institutional environment (e.g. legal framework, regulation)	Decree establishing National Health Insurance Company
Private Health Insurance policy framework (e.g. objective, population coverage, system description, types of coverage	
Market structure, conduct and performance (e.g. market concentration, barriers to entry, local vs. international, vertical integration, value-based health care)	
**Key findings related to scoping review question**	
Health System framework	
Eligibility	
Sector size	

User charges	
Institutional environment	
Legal framework	National Health Insurance Company as a single payer for the basic benefit package established as a Qatari Shareholding Company with Ministry of Business and Trade
Material regulation	
Prudential regulation	
Private Health Insurance policy framework	
Objective	
Population coverage	
System description	
Coverage type	
Market structure	
Concentration	
Barriers to entry	
Local vs international	
Vertical Integration	
Value Based Health Care	

Reviewer	Husein Reka
Date	20 January 2024
**Document information:**	
Name	Law No. (22) of 2021 regulating the provision of healthcare services within the State
Author(s)	Council of Ministers
Year of publication	18 Oct 2021
Country/Emirate	Qatar
Link	https://www.moph.gov.qa/_layouts/download.aspx?SourceUrl=/Admin/Lists/PublicationsAttachments/Attachments/220/Law%20No.%20(22)%20of%202021%20regulating%20the%20provision%20of%20healthcare%20services%20within%20the%20State.pdf
Aim/purpose	Promulgating new health insurance system in Qatar
Record #	721
**Inclusion/Exclusion Criteria**	
Population	Private Health Insurance in Qatar
Concept	Transformation/Reforms of Qatar Private Health Insurance Scheme
Context	GCC Health System
**Review framework domains studied in literature on private health insurance transformation**	
Health System Framework (e.g. eligibility, sector size, user charges)	
Institutional environment (e.g. legal framework, regulation)	Law giving mandate for a new health insurance scheme in Qatar based on Private Health Insurance
Private Health Insurance policy framework (e.g. objective, population coverage, system description, types of coverage	
Market structure, conduct and performance (e.g. market concentration, barriers to entry, local vs. international, vertical integration, value-based health care)	
**Key findings related to scoping review question**	

Health System framework	
Eligibility	
Sector size	
User charges	
Institutional environment	
Legal framework	Chapter two of this law determines free access to Government health providers for citizen through health card. Chapter 3 of this law requires all expats and visitors to have basic health insurance coverage through private health insurance companies in the country. Article 23 prohibits vertical integration between payer, TPA and provider
Material regulation	
Prudential regulation	
Private Health Insurance policy framework	
Objective	
Population coverage	
System description	
Coverage type	
Market structure	
Concentration	
Barriers to entry	
Local vs international	
Vertical Integration	
Value Based Health Care	

Reviewer	Husein Reka
Date	20 January 2024
**Document information:**	
Name	Minister of Health Decision No. 8 of 2022 issuing the executive regulations of Law No. (22) of 2021 regulating health care services within the country
Author(s)	Ministry of Public Health
Year of publication	23 Mar 2022
Country/Emirate	Qatar
Link	https://www.moph.gov.qa/_layouts/download.aspx?SourceUrl=/Admin/Lists/PublicationsAttachments/Attachments/222/Minister%20of%20Health%20Decision%20No.%208%20of%202022%20issuing%20the%20executive%20regulations%20of%20Law%20No.%20(22)%20of%202021%20regulating%20health%20care%20services%20within%20the%20country.pdf
Aim/purpose	Regulating private health insurance in Qatar
Record #	722
**Inclusion/Exclusion Criteria**	
Population	Private Health Insurance in Qatar
Concept	Transformation/Reforms of Qatar Private Health Insurance Scheme
Context	GCC Health System
**Review framework domains studied in literature on private health insurance transformation**	
Health System Framework (e.g. eligibility, sector size, user charges)	
Institutional environment (e.g. legal framework, regulation)	Regulations detailing new mandatory private health insurance scheme
Private Health Insurance policy framework (e.g. objective, population coverage, system description, types of coverage	

Market structure, conduct and performance (e.g. market concentration, barriers to entry, local vs. international, vertical integration, value-based health care)	
**Key findings related to scoping review question**	
Health System framework	
Eligibility	
Sector size	
User charges	
Institutional environment	
Legal framework	Regulation pursuant to Law no. 22 of 2021 regulating participants of the compulsory private health insurance scheme, mechanisms of premium collection, basic benefit package and other for residents and visitors
Material regulation	
Prudential regulation	
Private Health Insurance policy framework	
Objective	
Population coverage	
System description	
Coverage type	
Market structure	
Concentration	
Barriers to entry	
Local vs international	
Vertical Integration	
Value Based Health Care	

Reviewer	Husein Reka
Date	20 January 2024
**Document information:**	
Name	Qatar National Health Care Coding and Terminology Standards
Author(s)	Ministry of Public Health
Year of publication	20 Dec 2022
Country/Emirate	Qatar
Link	https://www.moph.gov.qa/_layouts/download.aspx?SourceUrl=/Admin/Lists/PublicationsAttachments/Attachments/224/QNH-CODING-TERMINOLOGY-ENG.pdf
Aim/purpose	Directive on clincial coding and terminology standards
Record #	724
**Inclusion/Exclusion Criteria**	
Population	Private Health Insurance in Qatar
Concept	Transformation/Reforms of Qatar Private Health Insurance Scheme
Context	GCC Health System
**Review framework domains studied in literature on private health insurance transformation**	
Health System Framework (e.g. eligibility, sector size, user charges)	
Institutional environment (e.g. legal framework, regulation)	
Private Health Insurance policy framework (e.g. objective, population coverage, system description, types of coverage	
Market structure, conduct and performance (e.g. market concentration, barriers to entry, local vs. international, vertical integration, value-based health care)	New coding and terminology standards in Qatar
**Key findings related to scoping review question**	
Health System framework	
Eligibility	
Sector size	

User charges	
Institutional environment	
Legal framework	
Material regulation	
Prudential regulation	
Private Health Insurance policy framework	
Objective	
Population coverage	
System description	
Coverage type	
Market structure	
Concentration	
Barriers to entry	
Local vs international	
Vertical Integration	
Value Based Health Care	Effective January 2023, Qatar mandated new coding and patient classification by switching from ICD-10 AM to ICD-10 CM and from AR-DRG to IR-DRG, and CPT

Reviewer	Husein Reka
Date	20 January 2024
**Document information:**	
Name	Qatar National Health Insurance Data Management
Author(s)	Ministry of Public Health
Year of publication	20 Dec 2022
Country/Emirate	Qatar
Link	https://www.moph.gov.qa/_layouts/download.aspx?SourceUrl=/Admin/Lists/PublicationsAttachments/Attachments/226/QNHI-DATA-MANAGEMENT-ENG.pdf
Aim/purpose	Directive on health insurance data management standards
Record #	725
**Inclusion/Exclusion Criteria**	
Population	Private Health Insurance in Qatar
Concept	Transformation/Reforms of Qatar Private Health Insurance Scheme
Context	GCC Health System
**Review framework domains studied in literature on private health insurance transformation**	
Health System Framework (e.g. eligibility, sector size, user charges)	
Institutional environment (e.g. legal framework, regulation)	
Private Health Insurance policy framework (e.g. objective, population coverage, system description, types of coverage	
Market structure, conduct and performance (e.g. market concentration, barriers to entry, local vs. international, vertical integration, value-based health care)	Health insurance data management standards in Qatar
**Key findings related to scoping review question**	
Health System framework	
Eligibility	
Sector size	
User charges	

Institutional environment	
Legal framework	
Material regulation	
Prudential regulation	
Private Health Insurance policy framework	
Objective	
Population coverage	
System description	
Coverage type	
Market structure	
Concentration	
Barriers to entry	
Local vs international	
Vertical Integration	
Value Based Health Care	This issued directive sets standards for Qatar Health Information Exchange system and Qatar Health Insurance Solutions

Reviewer	Husein Reka
Date	20 January 2024
**Document information:**	
Name	Decision (KH/115/2022) to issue controls for electronic connectivity to the health insurance system
Author(s)	Capital Market Authority
Year of publication	2 Aug 2022
Country/Emirate	Oman
Link	https://e.cma.gov.om/LegislationEncyclopedia/GetPDF/67?type=Decision
Aim/purpose	Directive on health insurance data management standards
Record #	735
**Inclusion/Exclusion Criteria**	
Population	Private Health Insurance in Oman
Concept	Transformation/Reforms of Oman Private Health Insurance Scheme
Context	GCC Health System
**Review framework domains studied in literature on private health insurance transformation**	
Health System Framework (e.g. eligibility, sector size, user charges)	
Institutional environment (e.g. legal framework, regulation)	
Private Health Insurance policy framework (e.g. objective, population coverage, system description, types of coverage	
Market structure, conduct and performance (e.g. market concentration, barriers to entry, local vs. international, vertical integration, value-based health care)	
**Key findings related to scoping review question**	
Health System framework	
Eligibility	
Sector size	
User charges	

Institutional environment	
Legal framework	
Material regulation	
Prudential regulation	
Private Health Insurance policy framework	
Objective	
Population coverage	
System description	
Coverage type	
Market structure	
Concentration	
Barriers to entry	
Local vs international	
Vertical Integration	
Value Based Health Care	

Reviewer	Husein Reka
Date	20 January 2024
**Document information:**	
Name	Decision No. KH/107/2022 On the Issuance of the Regulation of the Practice of the Activity of Management of The Revenue Cycle of the Health Insurance Claims
Author(s)	Capital Market Authority
Year of publication	6 Jul 2022
Country/Emirate	Oman
Link	https://e.cma.gov.om/LegislationEncyclopedia/GetPDF/65?type=Regulation
Aim/purpose	Regulating TPA in Oman
Record #	736
**Inclusion/Exclusion Criteria**	
Population	Private Health Insurance in Oman
Concept	Transformation/Reforms of Oman Private Health Insurance Scheme
Context	GCC Health System
**Review framework domains studied in literature on private health insurance transformation**	
Health System Framework (e.g. eligibility, sector size, user charges)	
Institutional environment (e.g. legal framework, regulation)	TPA regulation
Private Health Insurance policy framework (e.g. objective, population coverage, system description, types of coverage	
Market structure, conduct and performance (e.g. market concentration, barriers to entry, local vs. international, vertical integration, value-based health care)	
**Key findings related to scoping review question**	
Health System framework	
Eligibility	
Sector size	

User charges	
Institutional environment	
Legal framework	
Material regulation	Regulating the participation of Third Party Administrators for health insurance claims in the Sultana of Oman
Prudential regulation	
Private Health Insurance policy framework	
Objective	
Population coverage	
System description	
Coverage type	
Market structure	
Concentration	
Barriers to entry	
Local vs international	
Vertical Integration	
Value Based Health Care	

Reviewer	Husein Reka
Date	20 January 2024
**Document information:**	
Name	Circular No. (KH/3/2021) Unified Health Insurance Policy Form
Author(s)	Capital Market Authority
Year of publication	14 Jan 2021
Country/Emirate	Oman
Link	https://e.cma.gov.om/LegislationEncyclopedia/GetPDF/51?type=Circular
Aim/purpose	Unifiying health insurance policies in Oman
Record #	742
**Inclusion/Exclusion Criteria**	
Population	Private Health Insurance in Oman
Concept	Transformation/Reforms of Oman Private Health Insurance Scheme
Context	GCC Health System
**Review framework domains studied in literature on private health insurance transformation**	
Health System Framework (e.g. eligibility, sector size, user charges)	
Institutional environment (e.g. legal framework, regulation)	Unified Health Policy
Private Health Insurance policy framework (e.g. objective, population coverage, system description, types of coverage	
Market structure, conduct and performance (e.g. market concentration, barriers to entry, local vs. international, vertical integration, value-based health care)	
**Key findings related to scoping review question**	
Health System framework	
Eligibility	
Sector size	
User charges	

Institutional environment	
Legal framework	
Material regulation	CMA issued a circular unifying all private health insurance forms for the mandatory health insurance in Oman
Prudential regulation	
Private Health Insurance policy framework	
Objective	
Population coverage	
System description	
Coverage type	
Market structure	
Concentration	
Barriers to entry	
Local vs international	
Vertical Integration	
Value Based Health Care	

Reviewer	Husein Reka
Date	20 January 2024
**Document information:**	
Name	Decision No. KH34/2020 Issuing the Regulation of the Practice of Health Insurance Claims Management Activity
Author(s)	Capital Market Authority
Year of publication	19 Jul 2020
Country/Emirate	Oman
Link	https://e.cma.gov.om/LegislationEncyclopedia/GetPDF/14?type=Regulation
Aim/purpose	TPA regulation
Record #	744
**Inclusion/Exclusion Criteria**	
Population	Private Health Insurance in Oman
Concept	Transformation/Reforms of Oman Private Health Insurance Scheme
Context	GCC Health System
**Review framework domains studied in literature on private health insurance transformation**	
Health System Framework (e.g. eligibility, sector size, user charges)	
Institutional environment (e.g. legal framework, regulation)	TPA regulation
Private Health Insurance policy framework (e.g. objective, population coverage, system description, types of coverage	
Market structure, conduct and performance (e.g. market concentration, barriers to entry, local vs. international, vertical integration, value-based health care)	
**Key findings related to scoping review question**	
Health System framework	
Eligibility	
Sector size	
User charges	

Institutional environment	
Legal framework	
Material regulation	CMA issued a circular regulating the participation of Third-Party Administrators for health insurance claims in the Sultana of Oman
Prudential regulation	
Private Health Insurance policy framework	
Objective	
Population coverage	
System description	
Coverage type	
Market structure	
Concentration	
Barriers to entry	
Local vs international	
Vertical Integration	
Value Based Health Care	

Reviewer	Husein Reka
Date	20 January 2024
**Document information:**	
Name	Decision No. 78/2019 On the Issuance of the Health Insurance Rules
Author(s)	Capital Market Authority
Year of publication	8 Oct 2019
Country/Emirate	Oman
Link	https://e.cma.gov.om/LegislationEncyclopedia/GetPDF/11?type=Decision
Aim/purpose	
Record #	746
**Inclusion/Exclusion Criteria**	
Population	Private Health Insurance in Oman
Concept	Transformation/Reforms of Oman Private Health Insurance Scheme
Context	GCC Health System
**Review framework domains studied in literature on private health insurance transformation**	
Health System Framework (e.g. eligibility, sector size, user charges)	
Institutional environment (e.g. legal framework, regulation)	Regulation of health insurance
Private Health Insurance policy framework (e.g. objective, population coverage, system description, types of coverage	
Market structure, conduct and performance (e.g. market concentration, barriers to entry, local vs. international, vertical integration, value-based health care)	
**Key findings related to scoping review question**	
Health System framework	
Eligibility	
Sector size	
User charges	

Institutional environment	
Legal framework	
Material regulation	Decision regulating health insurance participants (licensing, policy, premium, obligations, etc).
Prudential regulation	
Private Health Insurance policy framework	
Objective	
Population coverage	
System description	
Coverage type	
Market structure	
Concentration	
Barriers to entry	
Local vs international	
Vertical Integration	Article 6 prohibits vertical integration between payers, providers and TPAs
Value Based Health Care	

Reviewer	Husein Reka
Date	20 January 2024
**Document information:**	
Name	No. 34/19 On the Issuance of The Unified Health Insurance Policy Form
Author(s)	Capital Market Authority
Year of publication	24 Mar 2019
Country/Emirate	Oman
Link	https://e.cma.gov.om/LegislationEncyclopedia/GetPDF/11?type=Decision
Aim/purpose	
Record #	748
**Inclusion/Exclusion Criteria**	
Population	Private Health Insurance in Oman
Concept	Transformation/Reforms of Oman Private Health Insurance Scheme
Context	GCC Health System
**Review framework domains studied in literature on private health insurance transformation**	
Health System Framework (e.g. eligibility, sector size, user charges)	
Institutional environment (e.g. legal framework, regulation)	Regulation of health insurance
Private Health Insurance policy framework (e.g. objective, population coverage, system description, types of coverage	
Market structure, conduct and performance (e.g. market concentration, barriers to entry, local vs. international, vertical integration, value-based health care)	
**Key findings related to scoping review question**	
Health System framework	
Eligibility	
Sector size	
User charges	

Institutional environment	
Legal framework	
Material regulation	Policy form covering benefit package, types of insurance, claims management and other aspects of mandatory health insurance
Prudential regulation	
Private Health Insurance policy framework	
Objective	
Population coverage	
System description	
Coverage type	
Market structure	
Concentration	
Barriers to entry	
Local vs international	
Vertical Integration	
Value Based Health Care	

Reviewer	Husein Reka
Date	20 January 2024
**Document information:**	
Name	Royal Decree No. 11/16 On the Issuance of Takaful Insurance Law
Author(s)	Sultan Court
Year of publication	24 Mar 2019
Country/Emirate	Oman
Link	https://e.cma.gov.om/LegislationEncyclopedia/GetPDF/11?type=Decision
Aim/purpose	
Record #	752
**Inclusion/Exclusion Criteria**	
Population	Private Health Insurance in Oman
Concept	Transformation/Reforms of Oman Private Health Insurance Scheme
Context	GCC Health System
**Review framework domains studied in literature on private health insurance transformation**	
Health System Framework (e.g. eligibility, sector size, user charges)	
Institutional environment (e.g. legal framework, regulation)	Takaful insurance
Private Health Insurance policy framework (e.g. objective, population coverage, system description, types of coverage	
Market structure, conduct and performance (e.g. market concentration, barriers to entry, local vs. international, vertical integration, value-based health care)	
**Key findings related to scoping review question**	
Health System framework	
Eligibility	
Sector size	
User charges	

Institutional environment	
Legal framework	Health Takaful is one of the Takaful Business branches
Material regulation	
Prudential regulation	
Private Health Insurance policy framework	
Objective	
Population coverage	
System description	
Coverage type	
Market structure	
Concentration	
Barriers to entry	
Local vs international	
Vertical Integration	
Value Based Health Care	

Reviewer	Husein Reka
Date	20 January 2024
**Document information:**	
Name	Royal Decree No. 45 / 2021 Amending provisions of Takaful law
Author(s)	Sultan Court
Year of publication	17 Jun 2021
Country/Emirate	Oman
Link	https://e.cma.gov.om/LegislationEncyclopedia/DownloadReference?documentId=43
Aim/purpose	
Record #	753
**Inclusion/Exclusion Criteria**	
Population	Private Health Insurance in Oman
Concept	Transformation/Reforms of Oman Private Health Insurance Scheme
Context	GCC Health System
**Review framework domains studied in literature on private health insurance transformation**	
Health System Framework (e.g. eligibility, sector size, user charges)	
Institutional environment (e.g. legal framework, regulation)	
Private Health Insurance policy framework (e.g. objective, population coverage, system description, types of coverage	
Market structure, conduct and performance (e.g. market concentration, barriers to entry, local vs. international, vertical integration, value-based health care)	
**Key findings related to scoping review question**	
Health System framework	
Eligibility	
Sector size	
User charges	

Institutional environment	
Legal framework	
Material regulation	
Prudential regulation	
Private Health Insurance policy framework	
Objective	
Population coverage	
System description	
Coverage type	
Market structure	
Concentration	
Barriers to entry	
Local vs international	
Vertical Integration	
Value Based Health Care	

Reviewer	Husein Reka
Date	20 January 2024
**Document information:**	
Name	Issuing the Health Insurance Rules
Author(s)	Capital market Authority
Year of publication	20 Oct 2019
Country/Emirate	Oman
Link	https://mjla.gov.om/eng/legislation/decisions/404
Aim/purpose	
Record #	783
**Inclusion/Exclusion Criteria**	
Population	Private Health Insurance in Oman
Concept	Transformation/Reforms of Oman Private Health Insurance Scheme
Context	GCC Health System
**Review framework domains studied in literature on private health insurance transformation**	
Health System Framework (e.g. eligibility, sector size, user charges)	
Institutional environment (e.g. legal framework, regulation)	
Private Health Insurance policy framework (e.g. objective, population coverage, system description, types of coverage	
Market structure, conduct and performance (e.g. market concentration, barriers to entry, local vs. international, vertical integration, value-based health care)	
**Key findings related to scoping review question**	
Health System framework	
Eligibility	
Sector size	
User charges	
Institutional environment	
Legal framework	

Material regulation	
Prudential regulation	
Private Health Insurance policy framework	
Objective	
Population coverage	
System description	
Coverage type	
Market structure	
Concentration	
Barriers to entry	
Local vs international	
Vertical Integration	
Value Based Health Care	

Reviewer	Husein Reka
Date	20 January 2024
**Document information:**	
Name	Issuing the Regulation Governing the Activity of Health Insurance Claims Management
Author(s)	Capital market Authority
Year of publication	19 Jul 2020
Country/Emirate	Oman
Link	https://mjla.gov.om/eng/legislation/decisions/404
Aim/purpose	Health insurance claims management regulation
Record #	787
**Inclusion/Exclusion Criteria**	
Population	Private Health Insurance in Oman
Concept	Transformation/Reforms of Oman Private Health Insurance Scheme
Context	GCC Health System
**Review framework domains studied in literature on private health insurance transformation**	
Health System Framework (e.g. eligibility, sector size, user charges)	
Institutional environment (e.g. legal framework, regulation)	
Private Health Insurance policy framework (e.g. objective, population coverage, system description, types of coverage	
Market structure, conduct and performance (e.g. market concentration, barriers to entry, local vs. international, vertical integration, value-based health care)	
**Key findings related to scoping review question**	
Health System framework	
Eligibility	
Sector size	
User charges	
Institutional environment	

Legal framework	
Material regulation	
Prudential regulation	
Private Health Insurance policy framework	
Objective	
Population coverage	
System description	
Coverage type	
Market structure	
Concentration	
Barriers to entry	
Local vs international	
Vertical Integration	
Value Based Health Care	

Reviewer	Husein Reka
Date	20 January 2024
**Document information:**	
Name	Issuing the Visitors Health Insurance Policy Form
Author(s)	Capital market Authority
Year of publication	2 Nov 2022
Country/Emirate	Oman
Link	https://mjla.gov.om/eng/legislation/decisions/404
Aim/purpose	Health insurance claims management regulation
Record #	793
**Inclusion/Exclusion Criteria**	
Population	Private Health Insurance in Oman
Concept	Transformation/Reforms of Oman Private Health Insurance Scheme
Context	GCC Health System
**Review framework domains studied in literature on private health insurance transformation**	
Health System Framework (e.g. eligibility, sector size, user charges)	
Institutional environment (e.g. legal framework, regulation)	
Private Health Insurance policy framework (e.g. objective, population coverage, system description, types of coverage	
Market structure, conduct and performance (e.g. market concentration, barriers to entry, local vs. international, vertical integration, value-based health care)	
**Key findings related to scoping review question**	
Health System framework	
Eligibility	
Sector size	
User charges	
Institutional environment	
Legal framework	

Material regulation	
Prudential regulation	
Private Health Insurance policy framework	
Objective	
Population coverage	
System description	
Coverage type	
Market structure	
Concentration	
Barriers to entry	
Local vs international	
Vertical Integration	
Value Based Health Care	

Reviewer	Husein Reka
Date	20 January 2024
**Document information:**	
Name	Minister of Public Health's Decision 17 of 2022 on health insurance premium of health care services for visitors
Author(s)	Ministry of Public Health
Year of publication	4 Sep 2022
Country/Emirate	Qatar
Link	https://www.moph.gov.qa/_layouts/download.aspx?SourceUrl=/Admin/Lists/PublicationsAttachments/Attachments/221/Minister%20of%20Public%20Health%27s%20Decision%2017%20of%202022%20on%20health%20insurance%20premium%20of%20health%20care%
Aim/purpose	Directive on health insurance data management standards
Record #	725
**Inclusion/Exclusion Criteria**	
Population	Private Health Insurance in Qatar
Concept	Transformation/Reforms of Qatar Private Health Insurance Scheme
Context	GCC Health System
**Review framework domains studied in literature on private health insurance transformation**	
Health System Framework (e.g. eligibility, sector size, user charges)	
Institutional environment (e.g. legal framework, regulation)	Visitor health insurance mandatory
Private Health Insurance policy framework (e.g. objective, population coverage, system description, types of coverage	
Market structure, conduct and performance (e.g. market concentration, barriers to entry, local vs. international, vertical integration, value-based health care)	
**Key findings related to scoping review question**	
Health System framework	

Eligibility	
Sector size	
User charges	
Institutional environment	
Legal framework	Ministerial decision on visitor visa premium (QAR 50/month) as part of visa issuance process
Material regulation	
Prudential regulation	
Private Health Insurance policy framework	
Objective	
Population coverage	
System description	
Coverage type	
Market structure	
Concentration	
Barriers to entry	
Local vs international	
Vertical Integration	
Value Based Health Care	

Reviewer	Husein Reka
Date	20 January 2024
**Document information:**	
Name	Federal Law No. 5 of 2012 Amending Some Provisions of the Federal Law No. 6 of 2007 concerning the Establishment of the Insurance Authority and Organization of its Operations
Author(s)	UAE President
Year of publication	1 Jan 2012
Country/Emirate	United Arab Emirates
Link	https://rulebook.centralbank.ae/en/rulebook/federal-law-no-5-2012-amending-some-provisions-federal-law-no-6-2007-concerning
Aim/purpose	Directive on health insurance data management standards
Record #	875
**Inclusion/Exclusion Criteria**	
Population	Private Health Insurance in UAE
Concept	Transformation/Reforms of UAE Private Health Insurance Scheme
Context	GCC Health System
**Review framework domains studied in literature on private health insurance transformation**	
Health System Framework (e.g. eligibility, sector size, user charges)	
Institutional environment (e.g. legal framework, regulation)	
Private Health Insurance policy framework (e.g. objective, population coverage, system description, types of coverage	
Market structure, conduct and performance (e.g. market concentration, barriers to entry, local vs. international, vertical integration, value-based health care)	
**Key findings related to scoping review question**	
Health System framework	

Eligibility	
Sector size	
User charges	
Institutional environment	
Legal framework	
Material regulation	
Prudential regulation	
Private Health Insurance policy framework	
Objective	
Population coverage	
System description	
Coverage type	
Market structure	
Concentration	
Barriers to entry	Amendments on board composition and IA revenues
Local vs international	
Vertical Integration	
Value Based Health Care	

Reviewer	Husein Reka
Date	20 January 2024
**Document information:**	
Name	Decretal Federal Law No. (24) of 2020 On the Amendment of Certain Provisions of the Federal Law No. (6) of 2007 On Establishment of the Insurance Authority & Organization of Insurance Operations
Author(s)	UAE President
Year of publication	27 Sep 2020
Country/Emirate	United Arab Emirates
Link	https://rulebook.centralbank.ae/sites/default/files/en_net_file_store/CBUAE_EN_4202_VER1.pdf
Aim/purpose	Abolition of UAE Insurance Authority
Record #	876
**Inclusion/Exclusion Criteria**	
Population	Private Health Insurance in UAE
Concept	Transformation/Reforms of UAE Private Health Insurance Scheme
Context	GCC Health System
**Review framework domains studied in literature on private health insurance transformation**	
Health System Framework (e.g. eligibility, sector size, user charges)	
Institutional environment (e.g. legal framework, regulation)	Insurance Authority abolished
Private Health Insurance policy framework (e.g. objective, population coverage, system description, types of coverage	
Market structure, conduct and performance (e.g. market concentration, barriers to entry, local vs. international, vertical integration, value-based health care)	
**Key findings related to scoping review question**	
Health System framework	
Eligibility	

Sector size	
User charges	
Institutional environment	
Legal framework	Insurance Authority scraped and insurance supervision moved back to Central Bank . The word (Authority "IA") shall be replaced by the phrase "The Central Bank", wherever mentioned in Federal Law No. (6) of 2007 referred to herein.<br>2. The word (Minister) shall replaced by the word "Chairmen", wherever mentioned in Federal Law No. (6) of 2007 referred to herein and in the bylaws, regulations and resolutions issued in implementation thereof.<br>3. The phrase (The Director General) shall be replaced by the phrase (The Governor of the Central Bank) wherever mentioned in Federal Law No. (6)
Material regulation	
Prudential regulation	
Private Health Insurance policy framework	
Objective	
Population coverage	
System description	
Coverage type	
Market structure	
Concentration	
Barriers to entry	
Local vs international	
Vertical Integration	
Value Based Health Care	

Reviewer	Husein Reka
Date	20 January 2024
**Document information:**	
Name	Insurance Authority Board Resolution No. 9 of 2011 Concerning the Instructions for Licensing Health Insurance Third Party Administrators and Regulation and Control of their Business
Author(s)	Insurance Authority
Year of publication	4 Dec 2011
Country/Emirate	United Arab Emirates
Link	https://www.centralbank.ae/media/ouwbmz4o/9-of-2011-concerning-licensing-tpas.pdf
Aim/purpose	Abolition of UAE Insurance Authority
Record #	878
**Inclusion/Exclusion Criteria**	
Population	Private Health Insurance in UAE
Concept	Transformation/Reforms of UAE Private Health Insurance Scheme
Context	GCC Health System
**Review framework domains studied in literature on private health insurance transformation**	
Health System Framework (e.g. eligibility, sector size, user charges)	
Institutional environment (e.g. legal framework, regulation)	Insurance Authority abolished
Private Health Insurance policy framework (e.g. objective, population coverage, system description, types of coverage	
Market structure, conduct and performance (e.g. market concentration, barriers to entry, local vs. international, vertical integration, value-based health care)	
**Key findings related to scoping review question**	
Health System framework	
Eligibility	

Sector size	
User charges	
Institutional environment	
Legal framework	Insurance Authority scraped and insurance supervision moved back to Central Bank . The word (Authority “IA”) shall be replaced by the phrase “The Central Bank”, wherever mentioned in Federal Law No. (6) of 2007 referred to herein.<br>2. The word (Minister) shall replaced by the word “Chairmen”, wherever mentioned in Federal Law No. (6) of 2007 referred to herein and in the bylaws, regulations and resolutions issued in implementation thereof.<br>3. The phrase (The Director General) shall be replaced by the phrase (The Governor of the Central Bank) wherever mentioned in Federal Law No. (6)
Material regulation	
Prudential regulation	
Private Health Insurance policy framework	
Objective	
Population coverage	
System description	
Coverage type	
Market structure	
Concentration	
Barriers to entry	
Local vs international	
Vertical Integration	
Value Based Health Care	

Reviewer	Husein Reka
Date	20 January 2024
**Document information:**	
Name	Law No. 15 of 2019 amending some provisions of Law No. (1) of 1999 regarding health insurance for foreigners and imposing fees for health services
Author(s)	Council of Ministers
Year of publication	24 Mar 2019
Country/Emirate	Kuwait
Link	https://kif-kw.com/Viewer/%D9%82%D8%A7%D9%86%D9%88%D9%86-%D8%B1%D9%82%D9%85-15-%D9%84%D8%B3%D9%86%D8%A9-2019-%D8%A8%D8%AA%D8%B9%D8%AF%D9%8A%D9%84--1332
Aim/purpose	Law amendment to require visitors health insurance
Record #	893
**Inclusion/Exclusion Criteria**	
Population	Private Health Insurance in Kuwait
Concept	Transformation/Reforms of Kuwait Private Health Insurance Scheme
Context	GCC Health System
**Review framework domains studied in literature on private health insurance transformation**	
Health System Framework (e.g. eligibility, sector size, user charges)	
Institutional environment (e.g. legal framework, regulation)	Law amendments
Private Health Insurance policy framework (e.g. objective, population coverage, system description, types of coverage	
Market structure, conduct and performance (e.g. market concentration, barriers to entry, local vs. international, vertical integration, value-based health care)	

**Key findings related to scoping review question**	
Health System framework	
Eligibility	
Sector size	
User charges	
Institutional environment	
Legal framework	Amendments to consider foreigners as temporary visitors to be required to buy health insurance
Material regulation	
Prudential regulation	
Private Health Insurance policy framework	
Objective	
Population coverage	
System description	
Coverage type	
Market structure	
Concentration	
Barriers to entry	
Local vs international	
Vertical Integration	
Value Based Health Care	

Reviewer	Husein Reka
Date	20 January 2024
**Document information:**	
Name	Decree Law No. 114 of 2014 regarding health insurance for retirees and its explanatory memorandum
Author(s)	Council of Ministers
Year of publication	1 Jan 2014
Country/Emirate	Kuwait
Link	https://kif-kw.com/Viewer/%D9%85%D8%B1%D8%B3%D9%88%D9%85-%D8%A8%D9%82%D8%A7%D9%86%D9%88%D9%86-%D8%B1%D9%82%D9%85-114-%D9%84%D8%B3%D9%86%D8%A9--73
Aim/purpose	Introduction of private health insurance for retirees
Record #	920
**Inclusion/Exclusion Criteria**	
Population	Private Health Insurance in Kuwait
Concept	Transformation/Reforms of Kuwait Private Health Insurance Scheme
Context	GCC Health System
**Review framework domains studied in literature on private health insurance transformation**	
Health System Framework (e.g. eligibility, sector size, user charges)	
Institutional environment (e.g. legal framework, regulation)	Promulgation of law
Private Health Insurance policy framework (e.g. objective, population coverage, system description, types of coverage	
Market structure, conduct and performance (e.g. market concentration, barriers to entry, local vs. international, vertical integration, value-based health care)	
**Key findings related to scoping review question**	

Health System framework	
Eligibility	
Sector size	
User charges	
Institutional environment	
Legal framework	Law on coverage of Kuwaiti retirees with Health Insurance managed by MOH through a tender to Private Health Insurance Companies
Material regulation	
Prudential regulation	
Private Health Insurance policy framework	
Objective	
Population coverage	
System description	
Coverage type	
Market structure	
Concentration	
Barriers to entry	
Local vs international	
Vertical Integration	
Value Based Health Care	

Reviewer	Husein Reka
Date	20 January 2024
**Document information:**	
Name	Implementing regulations for the cooperative health insurance system UPDATED VERSION
Author(s)	Council of Health Insurance
Year of publication	1 Jan 2022
Country/Emirate	Saudi Arabia
Link	https://www.chi.gov.sa/knowledge-center/DocLib/اللائحة%20التنفيذية%20لنظام%20الضمان%20الصحي%20التعاوني%2006%202023م%20.pdf
Aim/purpose	Update implementing regulation
Record #	946
**Inclusion/Exclusion Criteria**	
Population	Private Health Insurance in Saudi Arabia
Concept	Transformation/Reforms of Saudi Arabia Private Health Insurance Scheme
Context	GCC Health System
**Review framework domains studied in literature on private health insurance transformation**	
Health System Framework (e.g. eligibility, sector size, user charges)	
Institutional environment (e.g. legal framework, regulation)	
Private Health Insurance policy framework (e.g. objective, population coverage, system description, types of coverage	
Market structure, conduct and performance (e.g. market concentration, barriers to entry, local vs. international, vertical integration, value-based health care)	Updated implementing regulations on vertical integration
**Key findings related to scoping review question**	
Health System framework	
Eligibility	
Sector size	

User charges	
Institutional environment	
Legal framework	
Material regulation	
Prudential regulation	
Private Health Insurance policy framework	
Objective	
Population coverage	
System description	
Coverage type	
Market structure	
Concentration	
Barriers to entry	
Local vs international	
Vertical Integration	Article 117 prohibiting vertical integration abrogated
Value Based Health Care	

Reviewer	Husein Reka
Date	20 January 2024
**Document information:**	
Name	Appendix No. 5 Unified Health Insurance Card
Author(s)	Council of Health Insurance
Year of publication	1 Jan 2018
Country/Emirate	Saudi Arabia
Link	https://chi.gov.sa/Pages/PageNotFoundError.aspx?requestUrl=http://chi.gov.sa/AboutCCHI/Rules/list5/%D9%85%D9%84%D8%AD%D9%82%20%D8%B1%D9%82%D9%85%205.pdf
Aim/purpose	Health insurance card mandated
Record #	948
**Inclusion/Exclusion Criteria**	
Population	Private Health Insurance in Saudi Arabia
Concept	Transformation/Reforms of Saudi Arabia Private Health Insurance Scheme
Context	GCC Health System
**Review framework domains studied in literature on private health insurance transformation**	
Health System Framework (e.g. eligibility, sector size, user charges)	
Institutional environment (e.g. legal framework, regulation)	Requirements and format of Health Insurance Card
Private Health Insurance policy framework (e.g. objective, population coverage, system description, types of coverage	
Market structure, conduct and performance (e.g. market concentration, barriers to entry, local vs. international, vertical integration, value-based health care)	
**Key findings related to scoping review question**	
Health System framework	
Eligibility	
Sector size	

User charges	
Institutional environment	
Legal framework	
Material regulation	Requirements and format of Health Insurance Card
Prudential regulation	
Private Health Insurance policy framework	
Objective	
Population coverage	
System description	
Coverage type	
Market structure	
Concentration	
Barriers to entry	
Local vs international	
Vertical Integration	
Value Based Health Care	

Reviewer	Husein Reka
Date	20 January 2024
**Document information:**	
Name	Decision no. 18/5/R of Chairman of the Board Regulations of dispute and resolution center
Author(s)	Council of Health Insurance
Year of publication	28 Jan 2021
Country/Emirate	Saudi Arabia
Link	https://chi.gov.sa/Pages/PageNotFoundError.aspx?requestUrl=http://chi.gov.sa/AboutCCHI/Rules/list5/%D9%84%D8%A7%D8%A6%D8%AD%D8%A9%20%D9%85%D8%B1%D9%83%D8%B2%20%D8%A7%D9%84%D8%B5%D9%84%D8%AD%20%D9%88%D8%A7%D9%84%D8%AA%D8%B3%D9%88%D9%8A%D8%A7%D8%AA%20%D8%A7%D9%84%D9%85%D8%B9%D8%AA%D9%85%D8%AF%D8%A9.pdf
Aim/purpose	Health insurance card mandated
Record #	950
**Inclusion/Exclusion Criteria**	
Population	Private Health Insurance in Saudi Arabia
Concept	Transformation/Reforms of Saudi Arabia Private Health Insurance Scheme
Context	GCC Health System
**Review framework domains studied in literature on private health insurance transformation**	
Health System Framework (e.g. eligibility, sector size, user charges)	
Institutional environment (e.g. legal framework, regulation)	Dispute and resolution mechanism
Private Health Insurance policy framework (e.g. objective, population coverage, system description, types of coverage	

Market structure, conduct and performance (e.g. market concentration, barriers to entry, local vs. international, vertical integration, value-based health care)	
**Key findings related to scoping review question**	
Health System framework	
Eligibility	
Sector size	
User charges	
Institutional environment	
Legal framework	
Material regulation	Dispute and resolution mechanism for cooperative health insurance scheme participants
Prudential regulation	
Private Health Insurance policy framework	
Objective	
Population coverage	
System description	
Coverage type	
Market structure	
Concentration	
Barriers to entry	
Local vs international	
Vertical Integration	
Value Based Health Care	

Reviewer	Husein Reka
Date	20 January 2024
**Document information:**	
Name	Policy CCHI-08/3-PR-01-1/0 Policies and procedures for covering orphans with health insurance through foster families
Author(s)	Council of Health Insurance
Year of publication	1 Jan 2022
Country/Emirate	Saudi Arabia
Link	https://chi.gov.sa/Pages/PageNotFoundError.aspx?requestUrl=http://chi.gov.sa/AboutCCHI/Rules/list5/%D8%B3%D9%8A%D8%A7%D8%B3%D8%A7%D8%AA%20%D9%88%D8%A5%D8%AC%D8%B1%D8%A7%D8%A1%D8%A7%D8%AA%20%D8%B4%D9%85%D9%88%D9%84%20%D8%A7%D9%84%D8%A3%D9%8A%D8%AA%D8%A7%D9%85%20%D8%A7%D9%84%D9%85%D8%AD%D8%AA%D8%B6%D9%86%D9%8A%D9%86%20%D9%84%D8%AF%D9%89%20%D8%A7%D9%84%D8%A3%D8%B3%D8%B1%20%D8%A7%D9%84%D9%83%D8%A7%D9%81%D9%84%D8%A9%20%D8%A8%D8%A7%D9%84%D8%AA%D8%A7%D9%85%D9%8A%D9%86%20%D8%A7%D9%84%D8%B5%D8%AD%D9%8A%20%D8%A7%D9%84%D8%A7%D9%84%D8%B2%D8%A7%D9%85%D9%8A.pdf
Aim/purpose	Coverage of orphans adopted by foster families
Record #	951
**Inclusion/Exclusion Criteria**	
Population	Private Health Insurance in Saudi Arabia
Concept	Transformation/Reforms of Saudi Arabia Private Health Insurance Scheme
Context	GCC Health System
**Review framework domains studied in literature on private health insurance transformation**	

Health System Framework (e.g. eligibility, sector size, user charges)	Orphans' coverage
Institutional environment (e.g. legal framework, regulation)	
Private Health Insurance policy framework (e.g. objective, population coverage, system description, types of coverage	
Market structure, conduct and performance (e.g. market concentration, barriers to entry, local vs. international, vertical integration, value-based health care)	
**Key findings related to scoping review question**	
Health System framework	
Eligibility	Mechanisms of coverage of orphans adopted by foster families as per Council of Ministers decision no. 47 of 16/10/1443 Hijri
Sector size	
User charges	
Institutional environment	
Legal framework	
Material regulation	
Prudential regulation	
Private Health Insurance policy framework	
Objective	
Population coverage	
System description	
Coverage type	
Market structure	
Concentration	
Barriers to entry	
Local vs international	
Vertical Integration	
Value Based Health Care	

Reviewer	Husein Reka
Date	20 January 2024
**Document information:**	
Name	CCHI-08-PR-02-1/0 Beneficiaries Health Insurance Coverage Policy
Author(s)	Council of Health Insurance
Year of publication	1 Jan 2022
Country/Emirate	Saudi Arabia
Link	https://chi.gov.sa/Pages/PageNotFoundError.aspx?requestUrl=http://chi.gov.sa/AboutCCHI/Rules/list5/%D8%B3%D9%8A%D8%A7%D8%B3%D8%A9%20%D8%AA%D8%BA%D8%B7%D9%8A%D8%A9%20%D9%85%D8%B3%D8%AA%D9%81%D9%8A%D8%AF%D9%8A%20%D8%A7%D9%84%D8%AA%D8%A3%D9%85%D9%8A%D9%86%20%D8%A7%D9%84%D8%B5%D8%AD%D9%8A.pdf
Aim/purpose	Coverage policy
Record #	952
**Inclusion/Exclusion Criteria**	
Population	Private Health Insurance in Saudi Arabia
Concept	Transformation/Reforms of Saudi Arabia Private Health Insurance Scheme
Context	GCC Health System
**Review framework domains studied in literature on private health insurance transformation**	
Health System Framework (e.g. eligibility, sector size, user charges)	Coverage mechanisms
Institutional environment (e.g. legal framework, regulation)	
Private Health Insurance policy framework (e.g. objective, population coverage, system description, types of coverage	
Market structure, conduct and performance (e.g. market concentration, barriers to entry, local vs. international, vertical integration, value-based health care)	

**Key findings related to scoping review question**	
Health System framework	
Eligibility	Mechanisms of coverage of beneficiaries of the cooperative health insurance scheme
Sector size	
User charges	
Institutional environment	
Legal framework	
Material regulation	
Prudential regulation	
Private Health Insurance policy framework	
Objective	
Population coverage	
System description	
Coverage type	
Market structure	
Concentration	
Barriers to entry	
Local vs international	
Vertical Integration	
Value Based Health Care	

Reviewer	Husein Reka
Date	20 January 2024
**Document information:**	
Name	Unified Medical Disclosure Form (Appendix no.8)
Author(s)	Council of Health Insurance
Year of publication	1 Nov 2022
Country/Emirate	Saudi Arabia
Link	https://chi.gov.sa/Pages/PageNotFoundError.aspx?requestUrl=http://chi.gov.sa/AboutCCHI/Rules/list5/%D9%86%D9%85%D9%88%D8%B0%D8%AC%20%D8%A7%D9%84%D8%A5%D9%81%D8%B5%D8%A7%D8%AD%20%D8%A7%D9%84%D8%B7%D8%A8%D9%8A%20%D8%A7%D9%84%D9%85%D9%88%D8%AD%D8%AF.pdf
Aim/purpose	Unified Medical Form for pre-existing conditions
Record #	954
**Inclusion/Exclusion Criteria**	
Population	Private Health Insurance in Saudi Arabia
Concept	Transformation/Reforms of Saudi Arabia Private Health Insurance Scheme
Context	GCC Health System
**Review framework domains studied in literature on private health insurance transformation**	
Health System Framework (e.g. eligibility, sector size, user charges)	
Institutional environment (e.g. legal framework, regulation)	Medical Disclosure Form
Private Health Insurance policy framework (e.g. objective, population coverage, system description, types of coverage	
Market structure, conduct and performance (e.g. market concentration, barriers to entry, local vs. international, vertical integration, value-based health care)	
**Key findings related to scoping review question**	
Health System framework	

Eligibility	
Sector size	
User charges	
Institutional environment	
Legal framework	
Material regulation	Standardized Medical Disclosure Form
Prudential regulation	
Private Health Insurance policy framework	
Objective	
Population coverage	
System description	
Coverage type	
Market structure	
Concentration	
Barriers to entry	
Local vs international	
Vertical Integration	
Value Based Health Care	

Reviewer	Husein Reka
Date	20 January 2024
**Document information:**	
Name	CHI-09-PO-02-1/0 Beneficiary Centric Policy
Author(s)	Council of Health Insurance
Year of publication	7 Sep 2023
Country/Emirate	Saudi Arabia
Link	https://chi.gov.sa/Pages/PageNotFoundError.aspx?requestUrl=http://chi.gov.sa/AboutCCHI/Rules/list5/%D8%B3%D9%8A%D8%A7%D8%B3%D8%A9%20%D9%85%D8%B1%D9%83%D8%B2%D9%8A%D8%A9%20%D8%A7%D9%84%D9%85%D8%B3%D8%AA%D9%81%D9%8A%D8%AF.pdf
Aim/purpose	Mechanisms to collect information on beneficiaries experience
Record #	955
**Inclusion/Exclusion Criteria**	
Population	Private Health Insurance in Saudi Arabia
Concept	Transformation/Reforms of Saudi Arabia Private Health Insurance Scheme
Context	GCC Health System
**Review framework domains studied in literature on private health insurance transformation**	
Health System Framework (e.g. eligibility, sector size, user charges)	
Institutional environment (e.g. legal framework, regulation)	Mechanisms to collect information on beneficiaries experience
Private Health Insurance policy framework (e.g. objective, population coverage, system description, types of coverage	
Market structure, conduct and performance (e.g. market concentration, barriers to entry, local vs. international, vertical integration, value-based health care)	

**Key findings related to scoping review question**	
Health System framework	
Eligibility	
Sector size	
User charges	
Institutional environment	
Legal framework	
Material regulation	
Prudential regulation	
Private Health Insurance policy framework	
Objective	
Population coverage	
System description	
Coverage type	
Market structure	
Concentration	
Barriers to entry	
Local vs international	
Vertical Integration	
Value Based Health Care	

Reviewer	Husein Reka
Date	20 January 2024
**Document information:**	
Name	CHI-07/3/1/-MA-3/1/0 Medication Refill Guide in the Health Insurance Sector
Author(s)	Council of Health Insurance
Year of publication	1 Nov 2023
Country/Emirate	Saudi Arabia
Link	https://chi.gov.sa/Pages/PageNotFoundError.aspx?requestUrl=http://chi.gov.sa/AboutCCHI/Rules/list5/%D8%B3%D9%8A%D8%A7%D8%B3%D8%A9%20%D9%85%D8%B1%D9%83%D8%B2%D9%8A%D8%A9%20%D8%A7%D9%84%D9%85%D8%B3%D8%AA%D9%81%D9%8A%D8%AF.pdf
Aim/purpose	Refill guidelines
Record #	956
**Inclusion/Exclusion Criteria**	
Population	Private Health Insurance in Saudi Arabia
Concept	Transformation/Reforms of Saudi Arabia Private Health Insurance Scheme
Context	GCC Health System
**Review framework domains studied in literature on private health insurance transformation**	
Health System Framework (e.g. eligibility, sector size, user charges)	
Institutional environment (e.g. legal framework, regulation)	
Private Health Insurance policy framework (e.g. objective, population coverage, system description, types of coverage	Explanation on prescription refill and medical conditions eligible in private health insurance
Market structure, conduct and performance (e.g. market concentration, barriers to entry, local vs. international, vertical integration, value-based health care)	
**Key findings related to scoping review question**	

Health System framework	
Eligibility	
Sector size	
User charges	
Institutional environment	
Legal framework	
Material regulation	
Prudential regulation	
Private Health Insurance policy framework	
Objective	
Population coverage	Explanation on prescription refill and medical conditions eligible in private health insurance
System description	
Coverage type	
Market structure	
Concentration	
Barriers to entry	
Local vs international	
Vertical Integration	
Value Based Health Care	

Reviewer	Husein Reka
Date	20 January 2024
**Document information:**	
Name	Covering the beneficiary emergency conditions
Author(s)	Council of Health Insurance
Year of publication	21 Jul 2020
Country/Emirate	Saudi Arabia
Link	https://chi.gov.sa/MediaCenter/Decisions/Documents/%D8%AA%D8%B9%D9%85%D9%8A%D9%85%20%D8%AA%D8%BA%D8%B7%D9%8A%D8%A9%20%D8%A7%D9%84%D8%AD%D8%A7%D9%84%D8%A7%D8%AA%20%D8%A7%D9%84%D8%B7%D8%A7%D8%B1%D8%A6%D8%A9%20%D9%84%D9%84%D9%85%D8%A4%D9%85%D9%86%20%D9%84%D9%87%20F.V.pdf
Aim/purpose	Refill guidelines
Record #	957
**Inclusion/Exclusion Criteria**	
Population	Private Health Insurance in Saudi Arabia
Concept	Transformation/Reforms of Saudi Arabia Private Health Insurance Scheme
Context	GCC Health System
**Review framework domains studied in literature on private health insurance transformation**	
Health System Framework (e.g. eligibility, sector size, user charges)	
Institutional environment (e.g. legal framework, regulation)	
Private Health Insurance policy framework (e.g. objective, population coverage, system description, types of coverage	Emergency services coverage
Market structure, conduct and performance (e.g. market concentration, barriers to entry, local vs. international, vertical integration, value-based health care)	
**Key findings related to scoping review question**	

Health System framework	
Eligibility	
Sector size	
User charges	
Institutional environment	
Legal framework	
Material regulation	
Prudential regulation	
Private Health Insurance policy framework	
Objective	
Population coverage	Requirement to cover emergency services by private health insurance as per MOH standards
System description	
Coverage type	
Market structure	
Concentration	
Barriers to entry	
Local vs international	
Vertical Integration	
Value Based Health Care	

Reviewer	Husein Reka
Date	20 January 2024
**Document information:**	
Name	Electronic Health Insurance Card
Author(s)	Council of Health Insurance
Year of publication	18 Jul 2019
Country/Emirate	Saudi Arabia
Link	https://chi.gov.sa/MediaCenter/Decisions/Documents/%D8%AA%D8%B9%D9%85%D9%8A%D9%85%20%D8%AA%D8%BA%D8%B7%D9%8A%D8%A9%20%D8%A7%D9%84%D8%AD%D8%A7%D9%84%D8%A7%D8%AA%20%D8%A7%D9%84%D8%B7%D8%A7%D8%B1%D8%A6%D8%A9%20%D9%84%D9%84%D9%85%D8%A4%D9%85%D9%86%20%D9%84%D9%87%20F.V.pdf
Aim/purpose	Electronic Health Insurance Card
Record #	960
**Inclusion/Exclusion Criteria**	
Population	Private Health Insurance in Saudi Arabia
Concept	Transformation/Reforms of Saudi Arabia Private Health Insurance Scheme
Context	GCC Health System
**Review framework domains studied in literature on private health insurance transformation**	
Health System Framework (e.g. eligibility, sector size, user charges)	
Institutional environment (e.g. legal framework, regulation)	Electronic Health Insurance Card
Private Health Insurance policy framework (e.g. objective, population coverage, system description, types of coverage	
Market structure, conduct and performance (e.g. market concentration, barriers to entry, local vs. international, vertical integration, value-based health care)	
**Key findings related to scoping review question**	

Health System framework	
Eligibility	
Sector size	
User charges	
Institutional environment	
Legal framework	
Material regulation	Issuing electronic health insurance card only to beneficiaries (except class C beneficiaries who may not possess electronic devices)
Prudential regulation	
Private Health Insurance policy framework	
Objective	
Population coverage	Requirement to cover emergency services by private health insurance as per MOH standards
System description	
Coverage type	
Market structure	
Concentration	
Barriers to entry	
Local vs international	
Vertical Integration	
Value Based Health Care	

Reviewer	Husein Reka
Date	20 January 2024
**Document information:**	
Name	Coverage of HIV testing for pregnant women on the health insurance policy 3531/1440/1636
Author(s)	Council of Health Insurance
Year of publication	22 Apr 2019
Country/Emirate	Saudi Arabia
Link	https://chi.gov.sa/MediaCenter/Decisions/Documents/%D8%AA%D8%BA%D8%B7%D9%8A%D8%A9%20%D9%81%D8%AD%D8%B5%20%D8%A7%D9%84%D8%A5%D9%8A%D8%AF%D8%B2%20%D9%84%D9%84%D9%85%D8%B1%D8%A3%D8%A9%20%D8%A7%D9%84%D8%AD%D8%A7%D9%85%D9%84%20%D8%B9%D9%84%D9%89%20%D9%88%D8%AB%D9%8A%D9%82%D8%A9%20%D8%A7%D9%84%D8%AA%D8%A3%D9%85%D9%8A%D9%86%20%D8%A7%D9%84%D8%B5%D8%AD%D9%8A.pdf
Aim/purpose	HIV coverage for pregnant women
Record #	962
**Inclusion/Exclusion Criteria**	
Population	Private Health Insurance in Saudi Arabia
Concept	Transformation/Reforms of Saudi Arabia Private Health Insurance Scheme
Context	GCC Health System
**Review framework domains studied in literature on private health insurance transformation**	
Health System Framework (e.g. eligibility, sector size, user charges)	
Institutional environment (e.g. legal framework, regulation)	
Private Health Insurance policy framework (e.g. objective, population coverage, system description, types of coverage	Coverage of HIV testing during pregnancy

Market structure, conduct and performance (e.g. market concentration, barriers to entry, local vs. international, vertical integration, value-based health care)	
**Key findings related to scoping review question**	
Health System framework	
Eligibility	
Sector size	
User charges	
Institutional environment	
Legal framework	
Material regulation	Coverage of HIV testing during pregnancy
Prudential regulation	
Private Health Insurance policy framework	
Objective	
Population coverage	
System description	
Coverage type	
Market structure	
Concentration	
Barriers to entry	
Local vs international	
Vertical Integration	
Value Based Health Care	

Reviewer	Husein Reka
Date	20 January 2024
**Document information:**	
Name	Coverage of cancer treatment in the unified health insurance policy 3531/1208
Author(s)	Council of Health Insurance
Year of publication	28 Feb 2019
Country/Emirate	Saudi Arabia
Link	https://chi.gov.sa/MediaCenter/Decisions/Documents/%D8%AA%D8%BA%D8%B7%D9%8A%D8%A9%20%D8%B9%D9%84%D8%A7%D8%AC%20%D9%85%D8%B1%D8%B6%D9%89%20%D8%A7%D9%84%D8%B3%D8%B1%D8%B7%D8%A7%D9%86%20%D9%81%D9%8A%20%D9%88%D8%AB%D9%8A%D9%82%D8%A9%20%D8%A7%D9%84%D8%B6%D9%85%D8%A7%D9%86%20%D8%A7%D9%84%D8%B5%D8%AD%D9%8A%20%D8%A7%D9%84%D9%85%D9%88%D8%AD%D8%AF%D8%A9%20.pdf
Aim/purpose	Cancer coverage
Record #	964
**Inclusion/Exclusion Criteria**	
Population	Private Health Insurance in Saudi Arabia
Concept	Transformation/Reforms of Saudi Arabia Private Health Insurance Scheme
Context	GCC Health System
**Review framework domains studied in literature on private health insurance transformation**	
Health System Framework (e.g. eligibility, sector size, user charges)	
Institutional environment (e.g. legal framework, regulation)	
Private Health Insurance policy framework (e.g. objective, population coverage, system description, types of coverage	Cancer coverage

Market structure, conduct and performance (e.g. market concentration, barriers to entry, local vs. international, vertical integration, value-based health care)	
**Key findings related to scoping review question**	
Health System framework	
Eligibility	
Sector size	
User charges	
Institutional environment	
Legal framework	
Material regulation	Compliance with cancer coverage
Prudential regulation	
Private Health Insurance policy framework	
Objective	
Population coverage	
System description	
Coverage type	
Market structure	
Concentration	
Barriers to entry	
Local vs international	
Vertical Integration	
Value Based Health Care	

Reviewer	Husein Reka
Date	20 January 2024
**Document information:**	
Name	Copayment, UCAF/DECAF and triage 3531/1439/1733
Author(s)	Council of Health Insurance
Year of publication	30 Jul 2018
Country/Emirate	Saudi Arabia
Link	https://chi.gov.sa/Pages/PageNotFoundError.aspx?requestUrl=http://chi.gov.sa/InsuranceCompanies/Decisions/Documents/%D8%AA%D8%B9%D9%85%D9%8A%D9%85.pdf
Aim/purpose	Copayment
Record #	965
**Inclusion/Exclusion Criteria**	
Population	Private Health Insurance in Saudi Arabia
Concept	Transformation/Reforms of Saudi Arabia Private Health Insurance Scheme
Context	GCC Health System
**Review framework domains studied in literature on private health insurance transformation**	
Health System Framework (e.g. eligibility, sector size, user charges)	Copayment
Institutional environment (e.g. legal framework, regulation)	
Private Health Insurance policy framework (e.g. objective, population coverage, system description, types of coverage	
Market structure, conduct and performance (e.g. market concentration, barriers to entry, local vs. international, vertical integration, value-based health care)	
**Key findings related to scoping review question**	
Health System framework	
Eligibility	
Sector size	

User charges	Standardized copayment and claims forms
Institutional environment	
Legal framework	
Material regulation	
Prudential regulation	
Private Health Insurance policy framework	
Objective	
Population coverage	
System description	
Coverage type	
Market structure	
Concentration	
Barriers to entry	
Local vs international	
Vertical Integration	
Value Based Health Care	

Reviewer	Husein Reka
Date	20 January 2024
**Document information:**	
Name	A mechanism for settling and paying health service provider claims 3531/1681
Author(s)	Council of Health Insurance
Year of publication	22 Jul 2018
Country/Emirate	Saudi Arabia
Link	https://chi.gov.sa/Pages/PageNotFoundError.aspx?requestUrl=http://chi.gov.sa/InsuranceCompanies/Decisions/Documents/%D8%AA%D8%B9%D9%85%D9%8A%D9%85%20%D8%A7%D9%84%D9%8A%D8%A9%20%D8%AA%D8%B3%D9%88%D9%8A%D8%A9%20%D9%88%D8%B3%D8%AF%D8%A7%D8%AF%20%D9%85%D8%B3%D8%AA%D8%AD%D9%82%D8%A7%D8%AA%20%D9%85%D9%82%D8%AF%D9%85%D9%8A%20%D8%A7%D9%84%D8%AE%D8%AF%D9%85%D8%A9%20%D8%A7%D9%84%D8%B5%D8%AD%D9%8A%D8%A9.pdf
Aim/purpose	Claims submission cycle
Record #	966
**Inclusion/Exclusion Criteria**	
Population	Private Health Insurance in Saudi Arabia
Concept	Transformation/Reforms of Saudi Arabia Private Health Insurance Scheme
Context	GCC Health System
**Review framework domains studied in literature on private health insurance transformation**	
Health System Framework (e.g. eligibility, sector size, user charges)	
Institutional environment (e.g. legal framework, regulation)	Claims submission cycle
Private Health Insurance policy framework (e.g. objective, population coverage, system description, types of coverage	

Market structure, conduct and performance (e.g. market concentration, barriers to entry, local vs. international, vertical integration, value-based health care)	
**Key findings related to scoping review question**	
Health System framework	
Eligibility	
Sector size	
User charges	
Institutional environment	
Legal framework	
Material regulation	Claims submission cycle
Prudential regulation	
Private Health Insurance policy framework	
Objective	
Population coverage	
System description	
Coverage type	
Market structure	
Concentration	
Barriers to entry	
Local vs international	
Vertical Integration	
Value Based Health Care	

Reviewer	Husein Reka
Date	20 January 2024
**Document information:**	
Name	Requirements for Qualifying (Renewal of Qualification) for Claims Management Companies Companies
Author(s)	Council of Health Insurance
Year of publication	18 Nov 2017
Country/Emirate	Saudi Arabia
Link	https://chi.gov.sa/MediaCenter/Decisions/Pages/Decision5.aspx
Aim/purpose	TPA qualification
Record #	968
**Inclusion/Exclusion Criteria**	
Population	Private Health Insurance in Saudi Arabia
Concept	Transformation/Reforms of Saudi Arabia Private Health Insurance Scheme
Context	GCC Health System
**Review framework domains studied in literature on private health insurance transformation**	
Health System Framework (e.g. eligibility, sector size, user charges)	
Institutional environment (e.g. legal framework, regulation)	TPA qualification
Private Health Insurance policy framework (e.g. objective, population coverage, system description, types of coverage	
Market structure, conduct and performance (e.g. market concentration, barriers to entry, local vs. international, vertical integration, value-based health care)	
**Key findings related to scoping review question**	
Health System framework	
Eligibility	
Sector size	
User charges	

Institutional environment	
Legal framework	
Material regulation	Requirements and procedures for TPA to qualify for CHIS
Prudential regulation	
Private Health Insurance policy framework	
Objective	
Population coverage	
System description	
Coverage type	
Market structure	
Concentration	
Barriers to entry	
Local vs international	
Vertical Integration	
Value Based Health Care	

Reviewer	Husein Reka
Date	20 January 2024
**Document information:**	
Name	Applying the tenth international clincial coding system for diseases Australian Modification - sixth edition
Author(s)	Council of Health Insurance
Year of publication	19 Apr 2018
Country/Emirate	Saudi Arabia
Link	https://chi.gov.sa/MediaCenter/Decisions/Documents/ICD10-AM_Applying_Mandatory.pdf
Aim/purpose	Patient classification mandate ICD-10 AM Ed 6
Record #	969
**Inclusion/Exclusion Criteria**	
Population	Private Health Insurance in Saudi Arabia
Concept	Transformation/Reforms of Saudi Arabia Private Health Insurance Scheme
Context	GCC Health System
**Review framework domains studied in literature on private health insurance transformation**	
Health System Framework (e.g. eligibility, sector size, user charges)	
Institutional environment (e.g. legal framework, regulation)	
Private Health Insurance policy framework (e.g. objective, population coverage, system description, types of coverage	
Market structure, conduct and performance (e.g. market concentration, barriers to entry, local vs. international, vertical integration, value-based health care)	Patient classification system
**Key findings related to scoping review question**	
Health System framework	
Eligibility	
Sector size	
User charges	

Institutional environment	
Legal framework	
Material regulation	Requirements and procedures for TPA to qualify for CHIS
Prudential regulation	
Private Health Insurance policy framework	
Objective	
Population coverage	
System description	
Coverage type	
Market structure	
Concentration	
Barriers to entry	
Local vs international	
Vertical Integration	
Value Based Health Care	Requirement from the market to apply national approved clinical coding and patient classification system (ICD-10AM and AR-DRG)

Reviewer	Husein Reka
Date	20 January 2024
**Document information:**	
Name	Telemedicine Policy 1301
Author(s)	Council of Health Insurance
Year of publication	8 Jan 2023
Country/Emirate	Saudi Arabia
Link	https://chi.gov.sa/Rules/Documents/%D8%AA%D8%B9%D9%85%D9%8A%D9%85%20%D8%B3%D9%8A%D8%A7%D8%B3%D8%A9%20%D8%A7%D9%84%D8%B7%D8%A8%20%D8%A7%D9%84%D8%A7%D8%AA%D8%B5%D8%A7%D9%84%D9%8A%20.pdf
Aim/purpose	Include telemedicine in EBP
Record #	970
**Inclusion/Exclusion Criteria**	
Population	Private Health Insurance in Saudi Arabia
Concept	Transformation/Reforms of Saudi Arabia Private Health Insurance Scheme
Context	GCC Health System
**Review framework domains studied in literature on private health insurance transformation**	
Health System Framework (e.g. eligibility, sector size, user charges)	
Institutional environment (e.g. legal framework, regulation)	
Private Health Insurance policy framework (e.g. objective, population coverage, system description, types of coverage	Telemedicine services to be covered
Market structure, conduct and performance (e.g. market concentration, barriers to entry, local vs. international, vertical integration, value-based health care)	
**Key findings related to scoping review question**	
Health System framework	
Eligibility	

Sector size	
User charges	
Institutional environment	
Legal framework	
Material regulation	
Prudential regulation	
Private Health Insurance policy framework	
Objective	
Population coverage	
System description	
Coverage type	Telemedicine coverage
Market structure	
Concentration	
Barriers to entry	
Local vs international	
Vertical Integration	
Value Based Health Care	

Reviewer	Husein Reka
Date	20 January 2024
**Document information:**	
Name	Double insurance coverage 34
Author(s)	Council of Health Insurance
Year of publication	1 Aug 2023
Country/Emirate	Saudi Arabia
Link	https://chi.gov.sa/Rules/Documents/%D8%AA%D8%B9%D9%85%D9%8A%D9%85%20%D8%A7%D8%B2%D8%AF%D9%88%D8%A7%D8%AC%D9%8A%D8%A9%20%D8%A7%D9%84%D8%AA%D8%A3%D9%85%D9%8A%D9%86.pdf
Aim/purpose	Double coverage prohibited
Record #	971
**Inclusion/Exclusion Criteria**	
Population	Private Health Insurance in Saudi Arabia
Concept	Transformation/Reforms of Saudi Arabia Private Health Insurance Scheme
Context	GCC Health System
**Review framework domains studied in literature on private health insurance transformation**	
Health System Framework (e.g. eligibility, sector size, user charges)	
Institutional environment (e.g. legal framework, regulation)	
Private Health Insurance policy framework (e.g. objective, population coverage, system description, types of coverage	Double coverage
Market structure, conduct and performance (e.g. market concentration, barriers to entry, local vs. international, vertical integration, value-based health care)	
**Key findings related to scoping review question**	
Health System framework	
Eligibility	

Sector size	
User charges	
Institutional environment	
Legal framework	
Material regulation	
Prudential regulation	
Private Health Insurance policy framework	
Objective	
Population coverage	
System description	
Coverage type	Double health Insurance coverage stopped effective 1 August 2023
Market structure	
Concentration	
Barriers to entry	
Local vs international	
Vertical Integration	
Value Based Health Care	

Reviewer	Husein Reka
Date	20 January 2024
**Document information:**	
Name	Referral from a government health service provider to a specialized health center 1168
Author(s)	Council of Health Insurance
Year of publication	1 Aug 2023
Country/Emirate	Saudi Arabia
Link	https://chi.gov.sa/Rules/Documents/%D8%AA%D8%B9%D9%85%D9%8A%D9%85%20%D8%A7%D9%84%D8%A5%D8%AD%D8%A7%D9%84%D8%A9%20%D9%85%D9%86%20%D9%85%D9%82%D8%AF%D9%85%20%D8%AE%D8%AF%D9%85%D8%A9%20%D8%B5%D8%AD%D9%8A%20%D8%AD%D9%83%D9%88%D9%85%D9%8A%20%D8%A5%D9%84%D9%89%20%D9%85%D8%B1%D9%83%D8%B2%20%D8%B5%D8%AD%D9%8A%20%D9%85%D8%AA%D8%AE%D8%B5%D8%B5.pdf
Aim/purpose	Referral under Article 11 (clarification)
Record #	972
**Inclusion/Exclusion Criteria**	
Population	Private Health Insurance in Saudi Arabia
Concept	Transformation/Reforms of Saudi Arabia Private Health Insurance Scheme
Context	GCC Health System
**Review framework domains studied in literature on private health insurance transformation**	
Health System Framework (e.g. eligibility, sector size, user charges)	Referral
Institutional environment (e.g. legal framework, regulation)	
Private Health Insurance policy framework (e.g. objective, population coverage, system description, types of coverage	

Market structure, conduct and performance (e.g. market concentration, barriers to entry, local vs. international, vertical integration, value-based health care)	
**Key findings related to scoping review question**	
Health System framework	
Eligibility	Clarification on referral and prior authorization from Government provider as part of Article 11 mandate
Sector size	
User charges	
Institutional environment	
Legal framework	
Material regulation	
Prudential regulation	
Private Health Insurance policy framework	
Objective	
Population coverage	
System description	
Coverage type	
Market structure	
Concentration	
Barriers to entry	
Local vs international	
Vertical Integration	
Value Based Health Care	

Reviewer	Husein Reka
Date	20 January 2024
**Document information:**	
Name	Telemedicine Accreditation requirement 723
Author(s)	Council of Health Insurance
Year of publication	1 May 2023
Country/Emirate	Saudi Arabia
Link	https://chi.gov.sa/Rules/Documents/%D8%AA%D8%B9%D9%85%D9%8A%D9%85%20%D8%A7%D9%84%D8%A7%D8%A8%20%D8%A7%D9%84%D8%A7%D8%AA%D8%B5%D8%A7%D9%84%D9%8A%20%20.pdf
Aim/purpose	Telemedicine accreditation
Record #	973
**Inclusion/Exclusion Criteria**	
Population	Private Health Insurance in Saudi Arabia
Concept	Transformation/Reforms of Saudi Arabia Private Health Insurance Scheme
Context	GCC Health System
**Review framework domains studied in literature on private health insurance transformation**	
Health System Framework (e.g. eligibility, sector size, user charges)	
Institutional environment (e.g. legal framework, regulation)	Accreditation
Private Health Insurance policy framework (e.g. objective, population coverage, system description, types of coverage	
Market structure, conduct and performance (e.g. market concentration, barriers to entry, local vs. international, vertical integration, value-based health care)	
**Key findings related to scoping review question**	
Health System framework	
Eligibility	
Sector size	

User charges	
Institutional environment	
Legal framework	
Material regulation	Telemedicine providers must be accredited with CHI
Prudential regulation	
Private Health Insurance policy framework	
Objective	
Population coverage	
System description	
Coverage type	
Market structure	
Concentration	
Barriers to entry	
Local vs international	
Vertical Integration	
Value Based Health Care	

Reviewer	Husein Reka
Date	20 January 2024
**Document information:**	
Name	Ambulance services price list 882
Author(s)	Council of Health Insurance
Year of publication	15 Feb 2023
Country/Emirate	Saudi Arabia
Link	https://chi.gov.sa/Rules/Documents/%D8%AA%D8%B9%D9%85%D9%8A%D9%85%20%D8%AA%D8%AD%D8%AF%D9%8A%D8%AB%20%D8%A7%D9%84%D9%82%D8%A7%D8%A6%D9%85%D8%A9%20%D8%A7%D9%84%D8%B3%D8%B9%D8%B1%D9%8A%D8%A9%20%D9%88%D9%82%D8%A7%D8%A6%D9%85%D8%A9%20%D8%A7%D9%84%D9%85%D9%82%D8%A7%D8%A8%D9%84%20%D8%A7%D9%84%D9%85%D8%A7%D9%84%D9%8A%20%D9%84%D9%84%D8%AE%D8%AF%D9%85%D8%A7%D8%AA%20%D8%A7%D9%84%D8%A7%D8%B3%D8%B9%D8%A7%D9%81%D9%8A%D8%A9%20%20.pdf
Aim/purpose	Ambulance Services piece list
Record #	974
**Inclusion/Exclusion Criteria**	
Population	Private Health Insurance in Saudi Arabia
Concept	Transformation/Reforms of Saudi Arabia Private Health Insurance Scheme
Context	GCC Health System
**Review framework domains studied in literature on private health insurance transformation**	
Health System Framework (e.g. eligibility, sector size, user charges)	
Institutional environment (e.g. legal framework, regulation)	Accreditation

Private Health Insurance policy framework (e.g. objective, population coverage, system description, types of coverage	
Market structure, conduct and performance (e.g. market concentration, barriers to entry, local vs. international, vertical integration, value-based health care)	Price list
**Key findings related to scoping review question**	
Health System framework	
Eligibility	
Sector size	
User charges	
Institutional environment	
Legal framework	
Material regulation	
Prudential regulation	
Private Health Insurance policy framework	
Objective	
Population coverage	
System description	
Coverage type	
Market structure	
Concentration	
Barriers to entry	
Local vs international	
Vertical Integration	
Value Based Health Care	Price list mandated for Private Health Insurance Companies through Saudi Billing System for Saudi Red Crescent ambulance services

Reviewer	Husein Reka
Date	20 January 2024
**Document information:**	
Name	Generalizing coverage of the telemedicine service as one of the basic benefits in the Health Insurance Council document 500
Author(s)	Council of Health Insurance
Year of publication	20 Nov 2022
Country/Emirate	Saudi Arabia
Link	https://chi.gov.sa/Rules/Documents/%D8%AA%D8%B9%D9%85%D9%8A%D9%85%20%D8%AA%D8%BA%D8%B7%D9%8A%D8%A9%20%D8%AE%D8%AF%D9%85%D8%A9%20%D8%A7%D9%84%D8%B7%D8%A8%20%D8%A7%D9%84%D8%A7%D8%AA%D8%B5%D8%A7%D9%84%D9%8A%20%D9%83%D8%A3%D8%AD%D8%AF%20%D8%A7%D9%84%D9%85%D9%86%D8%A7%D9%81%D8%B9%20%D8%A7%D9%84%D8%A3%D8%A7%D8%B3%D9%8A%D8%A9%20%D9%81%D9%8A%20%D9%88%D8%AB%D9%8A%D9%82%D8%A9%20%D9%85%D8%AC%D9%84%D8%B3%20%D8%A7%D9%84%D8%B6%D9%85%D8%A7%D9%86%20%D8%A7%D9%84%D8%B5%D8%AD%D9%8A.PDF
Aim/purpose	Telemedicine coverage
Record #	975
**Inclusion/Exclusion Criteria**	
Population	Private Health Insurance in Saudi Arabia
Concept	Transformation/Reforms of Saudi Arabia Private Health Insurance Scheme
Context	GCC Health System
**Review framework domains studied in literature on private health insurance transformation**	
Health System Framework (e.g. eligibility, sector size, user charges)	

Institutional environment (e.g. legal framework, regulation)	
Private Health Insurance policy framework (e.g. objective, population coverage, system description, types of coverage	Telemedicine coverage
Market structure, conduct and performance (e.g. market concentration, barriers to entry, local vs. international, vertical integration, value-based health care)	
**Key findings related to scoping review question**	
Health System framework	
Eligibility	
Sector size	
User charges	
Institutional environment	
Legal framework	
Material regulation	
Prudential regulation	
Private Health Insurance policy framework	
Objective	
Population coverage	
System description	
Coverage type	Coverage of telemedicine until new Essential Benefit Package comes into effect
Market structure	
Concentration	
Barriers to entry	
Local vs international	
Vertical Integration	
Value Based Health Care	

Reviewer	Husein Reka
Date	20 January 2024
**Document information:**	
Name	Insurance Drug Formulary Implementation Guide for the updated Essential Benefit Package 289
Author(s)	Council of Health Insurance
Year of publication	28 Sep 2022
Country/Emirate	Saudi Arabia
Link	https://chi.gov.sa/Rules/Documents/%D8%AA%D8%B9%D9%85%D9%8A%D9%85%20%D8%A2%D9%84%D9%8A%D8%A9%20%D8%AA%D8%B7%D8%A8%D9%8A%D9%82%20%D8%AF%D9%84%D9%8A%D9%84%20%D8%A7%D9%84%D8%A3%D8%AF%D9%88%D9%8A%D8%A9%20%D8%A7%D9%84%D8%AA%D8%A3%D9%85%D9%8A%D9%86%D9%8A%D8%A9.pdf
Aim/purpose	Drug formulary introduction
Record #	976
**Inclusion/Exclusion Criteria**	
Population	Private Health Insurance in Saudi Arabia
Concept	Transformation/Reforms of Saudi Arabia Private Health Insurance Scheme
Context	GCC Health System
**Review framework domains studied in literature on private health insurance transformation**	
Health System Framework (e.g. eligibility, sector size, user charges)	
Institutional environment (e.g. legal framework, regulation)	
Private Health Insurance policy framework (e.g. objective, population coverage, system description, types of coverage	

Market structure, conduct and performance (e.g. market concentration, barriers to entry, local vs. international, vertical integration, value-based health care)	Drug formulary and generic prescription
**Key findings related to scoping review question**	
Health System framework	
Eligibility	
Sector size	
User charges	
Institutional environment	
Legal framework	
Material regulation	
Prudential regulation	
Private Health Insurance policy framework	
Objective	
Population coverage	
System description	
Coverage type	
Market structure	
Concentration	
Barriers to entry	
Local vs international	
Vertical Integration	
Value Based Health Care	Guidance to market how to implement drug formulary and generic dispensing/substitutions

Reviewer	Husein Reka
Date	20 January 2024
**Document information:**	
Name	Unified contract 818
Author(s)	Council of Health Insurance
Year of publication	31 Jan 2022
Country/Emirate	Saudi Arabia
Link	https://chi.gov.sa/Rules/Documents/%D9%85%D8%B2%D9%88%D8%AF%D9%8A%20%D8%A7%D9%84%D8%AE%D8%AF%D9%85%D8%A7%D8%AA%20%D8%A7%D9%84%D8%B5%D8%AD%D9%8A%D8%A9%20%D9%88%20%D8%B4%D8%B1%D9%83%D8%A7%D8%AA%20%D8%A7%D9%84%D8%AA%D8%A3%D9%85%D9%8A%D9%86%20%D8%A7%D9%84%D8%B5%D8%AD%D9%8A%20%D8%A7%D9%84%D9%85%D8%A4%D9%87%D9%84%D8%A9.pdf
Aim/purpose	Unified contract
Record #	982
**Inclusion/Exclusion Criteria**	
Population	Private Health Insurance in Saudi Arabia
Concept	Transformation/Reforms of Saudi Arabia Private Health Insurance Scheme
Context	GCC Health System
**Review framework domains studied in literature on private health insurance transformation**	
Health System Framework (e.g. eligibility, sector size, user charges)	
Institutional environment (e.g. legal framework, regulation)	
Private Health Insurance policy framework (e.g. objective, population coverage, system description, types of coverage	

Market structure, conduct and performance (e.g. market concentration, barriers to entry, local vs. international, vertical integration, value-based health care)	
**Key findings related to scoping review question**	
Health System framework	
Eligibility	
Sector size	
User charges	
Institutional environment	
Legal framework	
Material regulation	Unified contract
Prudential regulation	
Private Health Insurance policy framework	
Objective	
Population coverage	
System description	
Coverage type	
Market structure	
Concentration	
Barriers to entry	
Local vs international	
Vertical Integration	
Value Based Health Care	Reinforcement of Ministerial Decision no. 1143 19/02/2021 on provisions of unified contract in full

Reviewer	Husein Reka
Date	20 January 2024
**Document information:**	
Name	Unified contract announcement 1197
Author(s)	Council of Health Insurance
Year of publication	27 Jan 2021
Country/Emirate	Saudi Arabia
Link	https://chi.gov.sa/Rules/Documents/%D8%A7%D9%84%D8%B9%D9%82%D8%AF%20%D8%A7%D9%84%D9%85%D9%88%D8%AD%D8%AF.pdf
Aim/purpose	Unified contract announcement
Record #	983
**Inclusion/Exclusion Criteria**	
Population	Private Health Insurance in Saudi Arabia
Concept	Transformation/Reforms of Saudi Arabia Private Health Insurance Scheme
Context	GCC Health System
**Review framework domains studied in literature on private health insurance transformation**	
Health System Framework (e.g. eligibility, sector size, user charges)	
Institutional environment (e.g. legal framework, regulation)	
Private Health Insurance policy framework (e.g. objective, population coverage, system description, types of coverage	
Market structure, conduct and performance (e.g. market concentration, barriers to entry, local vs. international, vertical integration, value-based health care)	
**Key findings related to scoping review question**	
Health System framework	
Eligibility	
Sector size	
User charges	

Institutional environment	
Legal framework	
Material regulation	Unified contract introduced
Prudential regulation	
Private Health Insurance policy framework	
Objective	
Population coverage	
System description	
Coverage type	
Market structure	
Concentration	
Barriers to entry	
Local vs international	
Vertical Integration	
Value Based Health Care	Ministerial Decision no. 1143 19/02/2021 on provisions of unified contract to be implemented on all new or renewed contracts between insurance companies and health-care providers in the private sector shall start from 1/2/2021

Reviewer	Husein Reka
Date	20 January 2024
**Document information:**	
Name	Medical Prescription 1616
Author(s)	Council of Health Insurance
Year of publication	25 Jun 2020
Country/Emirate	Saudi Arabia
Link	https://chi.gov.sa/Rules/Documents/%D8%A7%D9%84%D9%88%D8%B5%D9%81%D8%A9%20%D8%A7%D9%84%D8%B7%D8%A8%D9%8A%D8%A9.pdf
Aim/purpose	Generic prescription mandate
Record #	985
**Inclusion/Exclusion Criteria**	
Population	Private Health Insurance in Saudi Arabia
Concept	Transformation/Reforms of Saudi Arabia Private Health Insurance Scheme
Context	GCC Health System
**Review framework domains studied in literature on private health insurance transformation**	
Health System Framework (e.g. eligibility, sector size, user charges)	
Institutional environment (e.g. legal framework, regulation)	
Private Health Insurance policy framework (e.g. objective, population coverage, system description, types of coverage	
Market structure, conduct and performance (e.g. market concentration, barriers to entry, local vs. international, vertical integration, value-based health care)	Generic prescription
**Key findings related to scoping review question**	
Health System framework	
Eligibility	
Sector size	
User charges	

Institutional environment	
Legal framework	
Material regulation	
Prudential regulation	
Private Health Insurance policy framework	
Objective	
Population coverage	
System description	
Coverage type	
Market structure	
Concentration	
Barriers to entry	
Local vs international	
Vertical Integration	
Value Based Health Care	Requirement to prescribe generic medicines as per Ministerial Decision No. (1438-1170075) dated 09/24/1438 AH

Reviewer	Husein Reka
Date	20 January 2024
**Document information:**	
Name	Uploading Insurance Data Document (HIDP) 1401
Author(s)	Council of Health Insurance
Year of publication	31 Mar 2020
Country/Emirate	Saudi Arabia
Link	https://chi.gov.sa/Rules/Documents/%D8%B1%D9%81%D8%B9%20%D8%A8%D9%8A%D8%A7%D9%86%D8%A7%D8%AA%20%D9%88%D8%AB%D8%A7%D8%A6%D9%82%20%D8%A7%D9%84%D8%AA%D8%A3%D9%85%D9%8A%D9%86%20.pdf
Aim/purpose	Mandatory beneficiary registration
Record #	988
**Inclusion/Exclusion Criteria**	
Population	Private Health Insurance in Saudi Arabia
Concept	Transformation/Reforms of Saudi Arabia Private Health Insurance Scheme
Context	GCC Health System
**Review framework domains studied in literature on private health insurance transformation**	
Health System Framework (e.g. eligibility, sector size, user charges)	Beneficiary registration
Institutional environment (e.g. legal framework, regulation)	
Private Health Insurance policy framework (e.g. objective, population coverage, system description, types of coverage	
Market structure, conduct and performance (e.g. market concentration, barriers to entry, local vs. international, vertical integration, value-based health care)	
**Key findings related to scoping review question**	
Health System framework	

Eligibility	Compliance with requirements to upload beneficiaries' data into Health Insurance Data Policy Document on assuring that the national ID of citizen and residence card of resident upon visiting the service provider is the sole identifier for all insured persons
Sector size	
User charges	
Institutional environment	
Legal framework	
Material regulation	
Prudential regulation	
Private Health Insurance policy framework	
Objective	
Population coverage	
System description	
Coverage type	
Market structure	
Concentration	
Barriers to entry	
Local vs international	
Vertical Integration	
Value Based Health Care	

Reviewer	Husein Reka
Date	20 January 2024
**Document information:**	
Name	Application of health insurance for pilgrims 873
Author(s)	Council of Health Insurance
Year of publication	31 Mar 2020
Country/Emirate	Saudi Arabia
Link	https://chi.gov.sa/Rules/Documents/%D8%AA%D8%B7%D8%A8%D9%8A%D9%82%20%D8%A7%D9%84%D8%AA%D8%A3%D9%85%D9%8A%D9%86%20%D8%A7%D9%84%D8%B5%D8%AD%D9%8A%20%D8%B9%D9%84%D9%89%20%D8%A7%D9%84%D9%85%D8%B9%D9%85%D8%AA%D8%B1%D9%8A%D9%86.pdf
Aim/purpose	Umrah health insurance
Record #	994
**Inclusion/Exclusion Criteria**	
Population	Private Health Insurance in Saudi Arabia
Concept	Transformation/Reforms of Saudi Arabia Private Health Insurance Scheme
Context	GCC Health System
**Review framework domains studied in literature on private health insurance transformation**	
Health System Framework (e.g. eligibility, sector size, user charges)	Pilgrims health insurance
Institutional environment (e.g. legal framework, regulation)	
Private Health Insurance policy framework (e.g. objective, population coverage, system description, types of coverage	
Market structure, conduct and performance (e.g. market concentration, barriers to entry, local vs. international, vertical integration, value-based health care)	
**Key findings related to scoping review question**	
Health System framework	

Eligibility	Pilgrim insurance coverage for those going for umrah
Sector size	
User charges	
Institutional environment	
Legal framework	
Material regulation	
Prudential regulation	
Private Health Insurance policy framework	
Objective	
Population coverage	
System description	
Coverage type	
Market structure	
Concentration	
Barriers to entry	
Local vs international	
Vertical Integration	
Value Based Health Care	

Reviewer	Husein Reka
Date	20 January 2024
**Document information:**	
Name	Cash payments 701
Author(s)	Council of Health Insurance
Year of publication	15 Dec 2019
Country/Emirate	Saudi Arabia
Link	https://chi.gov.sa/Rules/Documents/%20%D8%A7%D9%84%D8%AF%D9%81%D8%B9%20%D8%A7%D9%84%D9%86%D9%82%D8%AF%D9%8A.pdf
Aim/purpose	Prohibition of cash payments pending insurance approval
Record #	996
**Inclusion/Exclusion Criteria**	
Population	Private Health Insurance in Saudi Arabia
Concept	Transformation/Reforms of Saudi Arabia Private Health Insurance Scheme
Context	GCC Health System
**Review framework domains studied in literature on private health insurance transformation**	
Health System Framework (e.g. eligibility, sector size, user charges)	
Institutional environment (e.g. legal framework, regulation)	Cash payment prohibition
Private Health Insurance policy framework (e.g. objective, population coverage, system description, types of coverage	
Market structure, conduct and performance (e.g. market concentration, barriers to entry, local vs. international, vertical integration, value-based health care)	
**Key findings related to scoping review question**	
Health System framework	
Eligibility	
Sector size	
User charges	

Institutional environment	
Legal framework	
Material regulation	Requesting Service Providers to stop practice of insured parties to pay therapeutic services' costs in cash pending Insurance Company approval
Prudential regulation	
Private Health Insurance policy framework	
Objective	
Population coverage	
System description	
Coverage type	
Market structure	
Concentration	
Barriers to entry	
Local vs international	
Vertical Integration	
Value Based Health Care	

Reviewer	Husein Reka
Date	20 January 2024
**Document information:**	
Name	Collecting financial compensation for Red Crescent services 534
Author(s)	Council of Health Insurance
Year of publication	15 Dec 2019
Country/Emirate	Saudi Arabia
Link	https://chi.gov.sa/Rules/Documents/%D8%AA%D8%AD%D8%B5%D9%8A%D9%84%20%D8%A7%D9%84%D9%85%D9%82%D8%A7%D8%A8%D9%84%20%D8%A7%D9%84%D9%85%D8%A7%D9%84%D9%8A%20%D9%84%D8%AE%D8%AF%D9%85%D8%A7%D8%AA%20%D8%A7%D9%84%D9%87%D9%84%D8%A7%D9%84%20%D8%A7%D9%84%D8%A7%D8%AD%D9%85%D8%B1.pdf
Aim/purpose	Prohibition of cash payments pending insurance approval
Record #	997
**Inclusion/Exclusion Criteria**	
Population	Private Health Insurance in Saudi Arabia
Concept	Transformation/Reforms of Saudi Arabia Private Health Insurance Scheme
Context	GCC Health System
**Review framework domains studied in literature on private health insurance transformation**	
Health System Framework (e.g. eligibility, sector size, user charges)	
Institutional environment (e.g. legal framework, regulation)	Reimbursement for emergency ambulatory services
Private Health Insurance policy framework (e.g. objective, population coverage, system description, types of coverage	
Market structure, conduct and performance (e.g. market concentration, barriers to entry, local vs. international, vertical integration, value-based health care)	

**Key findings related to scoping review question**	
Health System framework	
Eligibility	
Sector size	
User charges	
Institutional environment	
Legal framework	
Material regulation	Initiating reimbursement of rendered services by Saudi Red Crescent
Prudential regulation	
Private Health Insurance policy framework	
Objective	
Population coverage	
System description	
Coverage type	
Market structure	
Concentration	
Barriers to entry	
Local vs international	
Vertical Integration	
Value Based Health Care	

Reviewer	Husein Reka
Date	20 January 2024
**Document information:**	
Name	Utilizing ID as an identifier for beneficiaries 535
Author(s)	Council of Health Insurance
Year of publication	21 Nov 2019
Country/Emirate	Saudi Arabia
Link	https://chi.gov.sa/Rules/Documents/%D8%AA%D8%AD%D8%B5%D9%8A%D9%84%20%D8%A7%D9%84%D9%85%D9%82%D8%A7%D8%A8%D9%84%20%D8%A7%D9%84%D9%85%D8%A7%D9%84%D9%8A%20%D9%84%D8%AE%D8%AF%D9%85%D8%A7%D8%AA%20%D8%A7%D9%84%D9%87%D9%84%D8%A7%D9%84%20%D8%A7%D9%84%D8%A7%D8%AD%D9%85%D8%B1.pdf
Aim/purpose	Introduction of National ID as identifier
Record #	998
**Inclusion/Exclusion Criteria**	
Population	Private Health Insurance in Saudi Arabia
Concept	Transformation/Reforms of Saudi Arabia Private Health Insurance Scheme
Context	GCC Health System
**Review framework domains studied in literature on private health insurance transformation**	
Health System Framework (e.g. eligibility, sector size, user charges)	National ID to identify eligibility
Institutional environment (e.g. legal framework, regulation)	
Private Health Insurance policy framework (e.g. objective, population coverage, system description, types of coverage	
Market structure, conduct and performance (e.g. market concentration, barriers to entry, local vs. international, vertical integration, value-based health care)	
**Key findings related to scoping review question**	

Health System framework	
Eligibility	National and Resident ID as unique identifier to access cooperative health insurance services
Sector size	
User charges	
Institutional environment	
Legal framework	
Material regulation	
Prudential regulation	
Private Health Insurance policy framework	
Objective	
Population coverage	
System description	
Coverage type	
Market structure	
Concentration	
Barriers to entry	
Local vs international	
Vertical Integration	
Value Based Health Care	

Reviewer	Husein Reka
Date	20 January 2024
**Document information:**	
Name	Unified Claims Form (UCAF)
Author(s)	Council of Health Insurance
Year of publication	1 Nov 2022
Country/Emirate	Saudi Arabia
Link	https://chi.gov.sa/Pages/PageNotFoundError.aspx?requestUrl=http://chi.gov.sa/InsuranceCompanies/Decisions/Documents/UCAF2.0.pdf
Aim/purpose	Introduction of UCAF
Record #	1003
**Inclusion/Exclusion Criteria**	
Population	Private Health Insurance in Saudi Arabia
Concept	Transformation/Reforms of Saudi Arabia Private Health Insurance Scheme
Context	GCC Health System
**Review framework domains studied in literature on private health insurance transformation**	
Health System Framework (e.g. eligibility, sector size, user charges)	
Institutional environment (e.g. legal framework, regulation)	Claims standardization
Private Health Insurance policy framework (e.g. objective, population coverage, system description, types of coverage	
Market structure, conduct and performance (e.g. market concentration, barriers to entry, local vs. international, vertical integration, value-based health care)	
**Key findings related to scoping review question**	
Health System framework	
Eligibility	
Sector size	
User charges	

Institutional environment	
Legal framework	
Material regulation	Standardized Unified Claims Form issued for the market
Prudential regulation	
Private Health Insurance policy framework	
Objective	
Population coverage	
System description	
Coverage type	
Market structure	
Concentration	
Barriers to entry	
Local vs international	
Vertical Integration	
Value Based Health Care	

Reviewer	Husein Reka
Date	20 January 2024
**Document information:**	
Name	Unified Dental Claims Form (DCAF)
Author(s)	Council of Health Insurance
Year of publication	1 Nov 2022
Country/Emirate	Saudi Arabia
Link	https://chi.gov.sa/Pages/PageNotFoundError.aspx?requestUrl=http://chi.gov.sa/InsuranceCompanies/Decisions/Documents/DCAF%202.0.pdf
Aim/purpose	Introduction of DCAF
Record #	1004
**Inclusion/Exclusion Criteria**	
Population	Private Health Insurance in Saudi Arabia
Concept	Transformation/Reforms of Saudi Arabia Private Health Insurance Scheme
Context	GCC Health System
**Review framework domains studied in literature on private health insurance transformation**	
Health System Framework (e.g. eligibility, sector size, user charges)	
Institutional environment (e.g. legal framework, regulation)	Claims standardization
Private Health Insurance policy framework (e.g. objective, population coverage, system description, types of coverage	
Market structure, conduct and performance (e.g. market concentration, barriers to entry, local vs. international, vertical integration, value-based health care)	
**Key findings related to scoping review question**	
Health System framework	
Eligibility	
Sector size	
User charges	

Institutional environment	
Legal framework	
Material regulation	Standardized Dental Unified Claims Form issued for the market
Prudential regulation	
Private Health Insurance policy framework	
Objective	
Population coverage	
System description	
Coverage type	
Market structure	
Concentration	
Barriers to entry	
Local vs international	
Vertical Integration	
Value Based Health Care	

Reviewer	Husein Reka
Date	20 January 2024
**Document information:**	
Name	Umrah Pilgrims Health Insurance Policy
Author(s)	Council of Health Insurance
Year of publication	1 Jan 2020
Country/Emirate	Saudi Arabia
Link	https://chi.gov.sa/Rules/DocLib/Umrah%20Pilgrims%E2%80%99%20Health%20Insurance%20Policy.pdf
Aim/purpose	Communication Umrah Pilgrims Health Insurance Policy
Record #	1006
**Inclusion/Exclusion Criteria**	
Population	Private Health Insurance in Saudi Arabia
Concept	Transformation/Reforms of Saudi Arabia Private Health Insurance Scheme
Context	GCC Health System
**Review framework domains studied in literature on private health insurance transformation**	
Health System Framework (e.g. eligibility, sector size, user charges)	Communication on policy
Institutional environment (e.g. legal framework, regulation)	
Private Health Insurance policy framework (e.g. objective, population coverage, system description, types of coverage	
Market structure, conduct and performance (e.g. market concentration, barriers to entry, local vs. international, vertical integration, value-based health care)	
**Key findings related to scoping review question**	
Health System framework	
Eligibility	Communication document on Pilgrims health insurance coverage
Sector size	
User charges	

Institutional environment	
Legal framework	
Material regulation	
Prudential regulation	
Private Health Insurance policy framework	
Objective	
Population coverage	
System description	
Coverage type	
Market structure	
Concentration	
Barriers to entry	
Local vs international	
Vertical Integration	
Value Based Health Care	

Reviewer	Husein Reka
Date	20 January 2024
**Document information:**	
Name	Health Insurance Coverage Policy for Privileged Residency Holder CHI-07-PO-01/1/0
Author(s)	Council of Health Insurance
Year of publication	19 May 2022
Country/Emirate	Saudi Arabia
Link	https://chi.gov.sa/Rules/DocLib3/%D8%B3%D9%8A%D8%A7%D8%B3%D8%A9%20%D8%A7%D9%84%D8%A7%D9%95%D9%82%D8%A7%D9%85%D8%A9%20%D8%A7%D9%84%D9%85%D9%85%D9%8A%D8%B2%D8%A9.pdf
Aim/purpose	Premium residence health insurance policy
Record #	1008
**Inclusion/Exclusion Criteria**	
Population	Private Health Insurance in Saudi Arabia
Concept	Transformation/Reforms of Saudi Arabia Private Health Insurance Scheme
Context	GCC Health System
**Review framework domains studied in literature on private health insurance transformation**	
Health System Framework (e.g. eligibility, sector size, user charges)	Premium resident
Institutional environment (e.g. legal framework, regulation)	
Private Health Insurance policy framework (e.g. objective, population coverage, system description, types of coverage	
Market structure, conduct and performance (e.g. market concentration, barriers to entry, local vs. international, vertical integration, value-based health care)	
**Key findings related to scoping review question**	
Health System framework	

Eligibility	Premium Residence program health insurance coverage
Sector size	
User charges	
Institutional environment	
Legal framework	
Material regulation	
Prudential regulation	
Private Health Insurance policy framework	
Objective	
Population coverage	
System description	
Coverage type	
Market structure	
Concentration	
Barriers to entry	
Local vs international	
Vertical Integration	
Value Based Health Care	

Reviewer	Husein Reka
Date	20 January 2024
**Document information:**	
Name	Procedures regulating the relationship between government health facilities and insurance companies Decision No. 676731
Author(s)	Council of Health Insurance
Year of publication	19 May 2022
Country/Emirate	Saudi Arabia
Link	https://chi.gov.sa/Rules/article11/Documents/%D8%A7%D9%84%D8%A7%D8%AC%D8%B1%D8%A7%D8%A1%D8%A7%D8%AA%20%D8%A7%D9%84%D9%85%D9%86%D8%B8%D9%85%D8%A9%20%D9%84%D9%84%D8%B9%D9%84%D8%A7%D9%82%D9%87%20%D8%A8%D9%8A%D9%86%20%D8%A7%D9%84%D9%85%D8%B1%D8%A7%D9%81%D9%82%20%D8%A7%D9%84%D8%B5%D8%AD%D9%8A%D8%A9%20%D8%A7%D9%84%D8%AD%D9%83%D9%88%D9%85%D9%8A%D8%A9%20%D9%88%D8%B4%D8%B1%D9%83%D8%A7%D8%AA%20%D8%A7%D9%84%D8%AA%D8%A7%D9%85%D9%8A%D9%86.pdf
Aim/purpose	Article 11 procedures
Record #	1010
**Inclusion/Exclusion Criteria**	
Population	Private Health Insurance in Saudi Arabia
Concept	Transformation/Reforms of Saudi Arabia Private Health Insurance Scheme
Context	GCC Health System
**Review framework domains studied in literature on private health insurance transformation**	
Health System Framework (e.g. eligibility, sector size, user charges)	
Institutional environment (e.g. legal framework, regulation)	Article 11 regulations

Private Health Insurance policy framework (e.g. objective, population coverage, system description, types of coverage	
Market structure, conduct and performance (e.g. market concentration, barriers to entry, local vs. international, vertical integration, value-based health care)	
**Key findings related to scoping review question**	
Health System framework	
Eligibility	
Sector size	
User charges	
Institutional environment	
Legal framework	Rules and procedures on applying Article 11 of Cooperative Health Insurance Law for Private Health Insurance Companies to pay to Public Health Care providers claims for rendered services
Material regulation	
Prudential regulation	
Private Health Insurance policy framework	
Objective	
Population coverage	
System description	
Coverage type	
Market structure	
Concentration	
Barriers to entry	
Local vs international	
Vertical Integration	
Value Based Health Care	

Reviewer	Husein Reka
Date	20 January 2024
**Document information:**	
Name	Nphies Onboarding Public Providers Billing
Author(s)	Council of Health Insurance
Year of publication	1 Jan 2020
Country/Emirate	Saudi Arabia
Link	https://chi.gov.sa/Rules/article11/Documents/%D8%AF%D9%84%D9%8A%D9%84%20%D8%AA%D9%87%D9%8A%D8%A6%D8%A9%20%D8%A7%D9%84%D9%82%D8%B7%D8%A7%D8%B9%20%D8%A7%D9%84%D8%B9%D8%A7%D9%85.pdf
Aim/purpose	NPHIES onboarding for public providers billing
Record #	1011
**Inclusion/Exclusion Criteria**	
Population	Private Health Insurance in Saudi Arabia
Concept	Transformation/Reforms of Saudi Arabia Private Health Insurance Scheme
Context	GCC Health System
**Review framework domains studied in literature on private health insurance transformation**	
Health System Framework (e.g. eligibility, sector size, user charges)	
Institutional environment (e.g. legal framework, regulation)	
Private Health Insurance policy framework (e.g. objective, population coverage, system description, types of coverage	
Market structure, conduct and performance (e.g. market concentration, barriers to entry, local vs. international, vertical integration, value-based health care)	NPHIES onboarding
**Key findings related to scoping review question**	
Health System framework	
Eligibility	

Sector size	
User charges	
Institutional environment	
Legal framework	
Material regulation	
Prudential regulation	
Private Health Insurance policy framework	
Objective	
Population coverage	
System description	
Coverage type	
Market structure	
Concentration	
Barriers to entry	
Local vs international	
Vertical Integration	
Value Based Health Care	Guidelines on applying a national health information exchange and health insurance bus (National Platform for Health Insurance Exchange, NPHIES) onboarding for public providers as part of Article 11

Reviewer	Husein Reka
Date	20 January 2024
**Document information:**	
Name	Appendix No. 4 Minimum Data Requirements
Author(s)	Council of Health Insurance
Year of publication	1 Jan 2022
Country/Emirate	Saudi Arabia
Link	https://chi.gov.sa/Rules/unitedc/Documents/%D8%A7%D9%84%D9%85%D9%84%D8%AD%D9%82%20%D8%B1%D9%82%D9%85%203_OCAF%202.0.pdf
Aim/purpose	Optical claims form
Record #	1016
**Inclusion/Exclusion Criteria**	
Population	Private Health Insurance in Saudi Arabia
Concept	Transformation/Reforms of Saudi Arabia Private Health Insurance Scheme
Context	GCC Health System
**Review framework domains studied in literature on private health insurance transformation**	
Health System Framework (e.g. eligibility, sector size, user charges)	
Institutional environment (e.g. legal framework, regulation)	Minimum Data Set mandate
Private Health Insurance policy framework (e.g. objective, population coverage, system description, types of coverage	
Market structure, conduct and performance (e.g. market concentration, barriers to entry, local vs. international, vertical integration, value-based health care)	
**Key findings related to scoping review question**	
Health System framework	
Eligibility	
Sector size	
User charges	

Institutional environment	
Legal framework	
Material regulation	Minimum Data Set mandated for cooperative health insurance scheme
Prudential regulation	
Private Health Insurance policy framework	
Objective	
Population coverage	
System description	
Coverage type	
Market structure	
Concentration	
Barriers to entry	
Local vs international	
Vertical Integration	
Value Based Health Care	

Reviewer	Husein Reka
Date	20 January 2024
**Document information:**	
Name	Appendix No.5 Provider reconciliation
Author(s)	Council of Health Insurance
Year of publication	1 Nov 2022
Country/Emirate	Saudi Arabia
Link	https://chi.gov.sa/Rules/unitedc/Documents/%D8%A7%D9%84%D9%85%D9%84%D8%AD%D9%82%20%D8%B1%D9%82%D9%85%203_OCAF%202.0.pdf
Aim/purpose	Optical claims form
Record #	1017
**Inclusion/Exclusion Criteria**	
Population	Private Health Insurance in Saudi Arabia
Concept	Transformation/Reforms of Saudi Arabia Private Health Insurance Scheme
Context	GCC Health System
**Review framework domains studied in literature on private health insurance transformation**	
Health System Framework (e.g. eligibility, sector size, user charges)	
Institutional environment (e.g. legal framework, regulation)	Provider reconciliation form
Private Health Insurance policy framework (e.g. objective, population coverage, system description, types of coverage	
Market structure, conduct and performance (e.g. market concentration, barriers to entry, local vs. international, vertical integration, value-based health care)	
**Key findings related to scoping review question**	
Health System framework	
Eligibility	
Sector size	
User charges	

Institutional environment	
Legal framework	
Material regulation	Provider reconciliation form
Prudential regulation	
Private Health Insurance policy framework	
Objective	
Population coverage	
System description	
Coverage type	
Market structure	
Concentration	
Barriers to entry	
Local vs international	
Vertical Integration	
Value Based Health Care	

Reviewer	Husein Reka
Date	20 January 2024
**Document information:**	
Name	Appendix No.6 Saudi Billing System
Author(s)	Council of Health Insurance
Year of publication	1 Nov 2022
Country/Emirate	Saudi Arabia
Link	https://chi.gov.sa/Rules/unitedc/Documents/%D9%85%D9%84%D8%AD%D9%82%206%20%D8%A7%D8%B3%D8%B9%D8%A7%D8%B1%20%D8%A7%D9%84%D8%AE%D8%AF%D9%85%D8%A7%D8%AA%20%D8%A7%D9%84%D8%B5%D8%AD%D9%8A%D8%A9.pdf
Aim/purpose	New billing system mandated
Record #	1018
**Inclusion/Exclusion Criteria**	
Population	Private Health Insurance in Saudi Arabia
Concept	Transformation/Reforms of Saudi Arabia Private Health Insurance Scheme
Context	GCC Health System
**Review framework domains studied in literature on private health insurance transformation**	
Health System Framework (e.g. eligibility, sector size, user charges)	
Institutional environment (e.g. legal framework, regulation)	
Private Health Insurance policy framework (e.g. objective, population coverage, system description, types of coverage	
Market structure, conduct and performance (e.g. market concentration, barriers to entry, local vs. international, vertical integration, value-based health care)	Novel billing system
**Key findings related to scoping review question**	
Health System framework	
Eligibility	

Sector size	
User charges	
Institutional environment	
Legal framework	
Material regulation	
Prudential regulation	
Private Health Insurance policy framework	
Objective	
Population coverage	
System description	
Coverage type	
Market structure	
Concentration	
Barriers to entry	
Local vs international	
Vertical Integration	
Value Based Health Care	Saudi Billing System schedule

Reviewer	Husein Reka
Date	20 January 2024
**Document information:**	
Name	The Unified Contract between Insurance Company and Health Service Provider in private sector
Author(s)	Council of Health Insurance
Year of publication	1 Nov 2022
Country/Emirate	Saudi Arabia
Link	https://chi.gov.sa/Rules/unitedc/Documents/.%D8%A7%D9%84%D8%B9%D9%82%D8%AF%20%D8%A7%D9%84%D9%85%D9%88%D8%AD%D8%AF.pdf
Aim/purpose	Unified contract
Record #	1019
**Inclusion/Exclusion Criteria**	
Population	Private Health Insurance in Saudi Arabia
Concept	Transformation/Reforms of Saudi Arabia Private Health Insurance Scheme
Context	GCC Health System
**Review framework domains studied in literature on private health insurance transformation**	
Health System Framework (e.g. eligibility, sector size, user charges)	
Institutional environment (e.g. legal framework, regulation)	
Private Health Insurance policy framework (e.g. objective, population coverage, system description, types of coverage	
Market structure, conduct and performance (e.g. market concentration, barriers to entry, local vs. international, vertical integration, value-based health care)	Unified contract
**Key findings related to scoping review question**	
Health System framework	
Eligibility	
Sector size	

User charges	
Institutional environment	
Legal framework	
Material regulation	Unified Contract template
Prudential regulation	
Private Health Insurance policy framework	
Objective	
Population coverage	
System description	
Coverage type	
Market structure	
Concentration	
Barriers to entry	
Local vs international	
Vertical Integration	
Value Based Health Care	

Reviewer	Husein Reka
Date	20 January 2024
**Document information:**	
Name	The Unified Contract between Insurance Company and Health Service Provider in private sector
Author(s)	Council of Health Insurance
Year of publication	1 Nov 2022
Country/Emirate	Saudi Arabia
Link	https://chi.gov.sa/Rules/unitedc/Documents/.%D8%A7%D9%84%D8%B9%D9%82%D8%AF%20%D8%A7%D9%84%D9%85%D9%88%D8%AD%D8%AF.pdf
Aim/purpose	Unified contract
Record #	1019
**Inclusion/Exclusion Criteria**	
Population	Private Health Insurance in Saudi Arabia
Concept	Transformation/Reforms of Saudi Arabia Private Health Insurance Scheme
Context	GCC Health System
**Review framework domains studied in literature on private health insurance transformation**	
Health System Framework (e.g. eligibility, sector size, user charges)	
Institutional environment (e.g. legal framework, regulation)	
Private Health Insurance policy framework (e.g. objective, population coverage, system description, types of coverage	
Market structure, conduct and performance (e.g. market concentration, barriers to entry, local vs. international, vertical integration, value-based health care)	Unified contract
**Key findings related to scoping review question**	
Health System framework	
Eligibility	
Sector size	

User charges	
Institutional environment	
Legal framework	
Material regulation	Unified Contract template
Prudential regulation	
Private Health Insurance policy framework	
Objective	
Population coverage	
System description	
Coverage type	
Market structure	
Concentration	
Barriers to entry	
Local vs international	
Vertical Integration	
Value Based Health Care	

Reviewer	Husein Reka
Date	20 January 2024
**Document information:**	
Name	Dhaman fund treatment eligibility
Author(s)	Council of Health Insurance
Year of publication	18 Aug 2022
Country/Emirate	Saudi Arabia
Link	https://chi.gov.sa/DocLib/%D9%86%D9%85%D9%88%D8%B0%D8%AC%20%D8%A7%D9%84%D8%A7%D9%86%D8%B6%D9%85%D8%A7%D9%85%20%D9%84%D8%A8%D8%B1%D9%86%D8%A7%D9%85%D8%AC%20%D8%AA%D8%BA%D8%B7%D9%8A%D8%A9%20%D9%85%D8%B3%D8%AA%D9%81%D9%8A%D8%AF%D9%8A%20%D8%A7%D9%84%D8%B6%D9%85%D8%A7%D9%86%20%D8%A7%D9%84%D8%B5%D8%AD%D9%8A.pdf
Aim/purpose	Dhaman Fund
Record #	1020
**Inclusion/Exclusion Criteria**	
Population	Private Health Insurance in Saudi Arabia
Concept	Transformation/Reforms of Saudi Arabia Private Health Insurance Scheme
Context	GCC Health System
**Review framework domains studied in literature on private health insurance transformation**	
Health System Framework (e.g. eligibility, sector size, user charges)	Dhaman fund treatment eligibility
Institutional environment (e.g. legal framework, regulation)	
Private Health Insurance policy framework (e.g. objective, population coverage, system description, types of coverage	

Market structure, conduct and performance (e.g. market concentration, barriers to entry, local vs. international, vertical integration, value-based health care)	
**Key findings related to scoping review question**	
Health System framework	
Eligibility	Dhaman fund treatment eligibility
Sector size	
User charges	
Institutional environment	
Legal framework	
Material regulation	
Prudential regulation	
Private Health Insurance policy framework	
Objective	
Population coverage	
System description	
Coverage type	
Market structure	
Concentration	
Barriers to entry	
Local vs international	
Vertical Integration	
Value Based Health Care	

Reviewer	Husein Reka
Date	20 January 2024
**Document information:**	
Name	Dhaman Fund Project
Author(s)	Council of Health Insurance
Year of publication	13 Jan 2022
Country/Emirate	Saudi Arabia
Link	https://chi.gov.sa/Pages/PageNotFoundError.aspx?requestUrl=http://chi.gov.sa/AboutCCHI/CCHIprograms/Pages/prog017.aspx
Aim/purpose	Dhaman Fund Project
Record #	1021
**Inclusion/Exclusion Criteria**	
Population	Private Health Insurance in Saudi Arabia
Concept	Transformation/Reforms of Saudi Arabia Private Health Insurance Scheme
Context	GCC Health System
**Review framework domains studied in literature on private health insurance transformation**	
Health System Framework (e.g. eligibility, sector size, user charges)	Dhaman fund
Institutional environment (e.g. legal framework, regulation)	
Private Health Insurance policy framework (e.g. objective, population coverage, system description, types of coverage	
Market structure, conduct and performance (e.g. market concentration, barriers to entry, local vs. international, vertical integration, value-based health care)	
**Key findings related to scoping review question**	
Health System framework	
Eligibility	Dhaman Fund explanation (covering cases that exhaust financial limits through existing coverage limits)
Sector size	

User charges	
Institutional environment	
Legal framework	
Material regulation	
Prudential regulation	
Private Health Insurance policy framework	
Objective	
Population coverage	
System description	
Coverage type	
Market structure	
Concentration	
Barriers to entry	
Local vs international	
Vertical Integration	
Value Based Health Care	

Reviewer	Husein Reka
Date	20 January 2024
**Document information:**	
Name	Insurance Authority Law COM Decision no. 85 of H. 28/01/1445
Author(s)	Council of Ministers
Year of publication	15 Aug 2023
Country/Emirate	Saudi Arabia
Link	https://laws.boe.gov.sa/BoeLaws/Laws/Viewer/cb6e9f52-08ed-4aad-9cb7-b08000dccc8b?lawId=6994e763-5a47-4c4e-b796-b08000dccc7d
Aim/purpose	Insurance Authority establishment
Record #	1029
**Inclusion/Exclusion Criteria**	
Population	Private Health Insurance in Saudi Arabia
Concept	Transformation/Reforms of Saudi Arabia Private Health Insurance Scheme
Context	GCC Health System
**Review framework domains studied in literature on private health insurance transformation**	
Health System Framework (e.g. eligibility, sector size, user charges)	
Institutional environment (e.g. legal framework, regulation)	Insurance Authority establishment
Private Health Insurance policy framework (e.g. objective, population coverage, system description, types of coverage	
Market structure, conduct and performance (e.g. market concentration, barriers to entry, local vs. international, vertical integration, value-based health care)	
**Key findings related to scoping review question**	
Health System framework	
Eligibility	
Sector size	

User charges	
Institutional environment	
Legal framework	Council of Ministers established Insurance Authority giving all powers and responsibilities related to insurance sector, including health insurance with transition within three years from the date of this law from Council of Health Insurance
Material regulation	
Prudential regulation	
Private Health Insurance policy framework	
Objective	
Population coverage	
System description	
Coverage type	
Market structure	
Concentration	
Barriers to entry	
Local vs international	
Vertical Integration	
Value Based Health Care	

Reviewer	Husein Reka
Date	20 January 2024
**Document information:**	
Name	Circular Circular no. 5650/ran to market to enforce CoM decision No. 206
Author(s)	Council of Ministers
Year of publication	7 Jan 2009
Country/Emirate	Saudi Arabia
Link	
Aim/purpose	Private health insurance coverage for national
Record #	1033
**Inclusion/Exclusion Criteria**	
Population	Private Health Insurance in Saudi Arabia
Concept	Transformation/Reforms of Saudi Arabia Private Health Insurance Scheme
Context	GCC Health System
**Review framework domains studied in literature on private health insurance transformation**	
Health System Framework (e.g. eligibility, sector size, user charges)	
Institutional environment (e.g. legal framework, regulation)	Enforcement of mandate
Private Health Insurance policy framework (e.g. objective, population coverage, system description, types of coverage	
Market structure, conduct and performance (e.g. market concentration, barriers to entry, local vs. international, vertical integration, value-based health care)	
**Key findings related to scoping review question**	
Health System framework	
Eligibility	
Sector size	
User charges	
Institutional environment	

Legal framework	Circular to market to enforce CoM decision No. 206 on Saudis in private sector coverage with PHI
Material regulation	
Prudential regulation	
Private Health Insurance policy framework	
Objective	
Population coverage	
System description	
Coverage type	
Market structure	
Concentration	
Barriers to entry	
Local vs international	
Vertical Integration	
Value Based Health Care	

Reviewer	Husein Reka
Date	20 January 2024
**Document information:**	
Name	Decision no. 586 of 2010
Author(s)	Council of Ministers
Year of publication	1 May 2010
Country/Emirate	Kuwait
Link	
Aim/purpose	Establishing Dhaman Health Assurance Company
Record #	1040
**Inclusion/Exclusion Criteria**	
Population	Private Health Insurance in Kuwait
Concept	Transformation/Reforms of Kuwait Private Health Insurance Scheme
Context	GCC Health System
**Review framework domains studied in literature on private health insurance transformation**	
Health System Framework (e.g. eligibility, sector size, user charges)	
Institutional environment (e.g. legal framework, regulation)	Establishment of health assurance company
Private Health Insurance policy framework (e.g. objective, population coverage, system description, types of coverage	
Market structure, conduct and performance (e.g. market concentration, barriers to entry, local vs. international, vertical integration, value-based health care)	
**Key findings related to scoping review question**	
Health System framework	
Eligibility	
Sector size	
User charges	
Institutional environment	
Legal framework	Decision to establish Dhaman Kuwait

Material regulation	
Prudential regulation	
Private Health Insurance policy framework	
Objective	
Population coverage	
System description	
Coverage type	
Market structure	
Concentration	
Barriers to entry	
Local vs international	
Vertical Integration	
Value Based Health Care	

Reviewer	Husein Reka
Date	20 January 2024
**Document information:**	
Name	Extension of double coverage prohibition for Qatari Nationals by one year MASHS/8/2015
Author(s)	Supreme Council of Health
Year of publication	30 Apr 2015
Country/Emirate	Qatar
Link	https://www.facebook.com/photo/?fbid=970690319617268&set=a.1059963000689999
Aim/purpose	Extend double coverage prohibition for nationals
Record #	1041
**Inclusion/Exclusion Criteria**	
Population	Private Health Insurance in Qatar
Concept	Transformation/Reforms of Qatar Private Health Insurance Scheme
Context	GCC Health System
**Review framework domains studied in literature on private health insurance transformation**	
Health System Framework (e.g. eligibility, sector size, user charges)	
Institutional environment (e.g. legal framework, regulation)	
Private Health Insurance policy framework (e.g. objective, population coverage, system description, types of coverage	Double coverage
Market structure, conduct and performance (e.g. market concentration, barriers to entry, local vs. international, vertical integration, value-based health care)	
**Key findings related to scoping review question**	
Health System framework	
Eligibility	
Sector size	
User charges	

Institutional environment	
Legal framework	
Material regulation	
Prudential regulation	
Private Health Insurance policy framework	
Objective	
Population coverage	
System description	
Coverage type	Extension of double coverage prohibition for Qatari Nationals by one year
Market structure	
Concentration	
Barriers to entry	
Local vs international	
Vertical Integration	
Value Based Health Care	

Reviewer	Husein Reka
Date	20 January 2024
**Document information:**	
Name	The Health Insurance Council launches the “Dhaman Fund” Board Decision
Author(s)	Council of Health Insurance
Year of publication	18 Oct 2021
Country/Emirate	Saudi Arabia
Link	https://chi.gov.sa/en/mediacenter/news/pages/news-10-18-2021.aspx
Aim/purpose	Dhaman Fund launch announcement
Record #	1042
**Inclusion/Exclusion Criteria**	
Population	Private Health Insurance in Saudi Arabia
Concept	Transformation/Reforms of Saudi Arabia Private Health Insurance Scheme
Context	GCC Health System
**Review framework domains studied in literature on private health insurance transformation**	
Health System Framework (e.g. eligibility, sector size, user charges)	Dhaman Fund
Institutional environment (e.g. legal framework, regulation)	
Private Health Insurance policy framework (e.g. objective, population coverage, system description, types of coverage	
Market structure, conduct and performance (e.g. market concentration, barriers to entry, local vs. international, vertical integration, value-based health care)	
**Key findings related to scoping review question**	
Health System framework	
Eligibility	Announcement of Dhaman Fund launch
Sector size	
User charges	

Institutional environment	
Legal framework	Circular to market to enforce CoM decision No. 206 on Saudis in private sector coverage with PHI
Material regulation	
Prudential regulation	
Private Health Insurance policy framework	
Objective	
Population coverage	
System description	
Coverage type	
Market structure	
Concentration	
Barriers to entry	
Local vs international	
Vertical Integration	
Value Based Health Care	

Reviewer	Husein Reka
Date	20 January 2024
**Document information:**	
Name	Rules of Forming and Managing Health Insurance Risk Pools through Brokers
Author(s)	Saudi Central Bank SAMA
Year of publication	18 Oct 2021
Country/Emirate	Saudi Arabia
Link	https://www.ia.gov.sa/Documents/Rules/en/Rules_of_Forming_and_Managing_Health_Insurance_Risk_Pools_through_Brokers.pdf
Aim/purpose	Brokers managed health insurance pools
Record #	1043
**Inclusion/Exclusion Criteria**	
Population	Private Health Insurance in Saudi Arabia
Concept	Transformation/Reforms of Saudi Arabia Private Health Insurance Scheme
Context	GCC Health System
**Review framework domains studied in literature on private health insurance transformation**	
Health System Framework (e.g. eligibility, sector size, user charges)	Dhaman Fund
Institutional environment (e.g. legal framework, regulation)	
Private Health Insurance policy framework (e.g. objective, population coverage, system description, types of coverage	Fund pooling
Market structure, conduct and performance (e.g. market concentration, barriers to entry, local vs. international, vertical integration, value-based health care)	
**Key findings related to scoping review question**	
Health System framework	
Eligibility	
Sector size	

User charges	
Institutional environment	
Legal framework	
Material regulation	
Prudential regulation	
Private Health Insurance policy framework	
Objective	
Population coverage	Regulations and rules for brokers to form and manage health insurance pools
System description	
Coverage type	
Market structure	
Concentration	
Barriers to entry	
Local vs international	
Vertical Integration	
Value Based Health Care	
